# Proton trap engineered electric swing adsorption for scalable and cost-effective direct air capture

**DOI:** 10.1038/s41467-026-75916-7

**Published:** 2026-07-21

**Authors:** Yao Shen, Kai Pang, Weichen Zhao, Liang Chen, Jingkai Zhao, Jiexu Ye, Beini Zhang, Sujing Li, Wei Li, Zhen Xu, Jing Meng, Xiang Gao, Shihan Zhang

**Affiliations:** 1 Zhejiang Key Laboratory of Clean Energy Conversion and Utilization, Science and Education Integration College of Energy and Carbon Neutralization, Zhejiang University of Technology, Hangzhou, China; 2 State Key Laboratory of Advanced Separation Membrane Materials, Zhejiang University of Technology, Hangzhou, China; 3 Baima Lake Laboratory, Hangzhou, China; 4 MOE Key Laboratory of Macromolecular Synthesis and Functionalization, International Research Center for X Polymers, Department of Polymer Science and Engineering, Zhejiang University, Hangzhou, China; 5 The Bartlett School of Sustainable Construction, University College London, London, UK; 6 College of Material, Chemistry and Chemical Engineering, Key Laboratory of Organosilicon Chemistry and Material Technology, Ministry of Education, Hangzhou Normal University, Hangzhou, China; 7 College of Chemical and Biological Engineering, Zhejiang University, Hangzhou, China

**Keywords:** Carbon capture and storage, Chemical engineering, Porous materials, Graphene

## Abstract

Direct air capture (DAC) is critical to achieve carbon neutrality, yet current technologies face significant barriers to widespread, cost-effective deployment. Amine-based electric swing adsorption (ESA) offers a promising low-energy, steam-free pathway, but its efficiency is fundamentally limited by an inherent 2:1 amine-to-CO_2_ stoichiometric penalty. Here, we overcome this bottleneck by engineering a point defect-mediated proton trapping network into ESA sorbents, enabling a 1:1 amine-CO_2_ stoichiometry. Our engineered sorbent achieves a CO_2_ uptake of 6.57 mmol g^−1^ from 400 ppm CO_2_, a 28.8% improvement over the state-of-the-art sorbents. Regeneration is achieved with a low energy input of 3.4 GJ t^−1^ and exhibits a CO_2_ release rate 48% faster than conventional thermal methods. *N*5-*d*GA remains stable under 0-80% relative humidity fluctuations and at a gas velocity of 1 m s^−1^. Techno-economic analysis projects DAC operating costs of $48-62 t^−1^ using renewable electricity, up to 78% lower than temperature swing adsorption DAC and below the $100 t^−1^ CO_2_ target. This work presents a sorbent design and ESA process, establishing a scientifically rigorous and economically viable pathway towards gigaton-scale DAC deployment.

## Abstract (web summary)

Direct air capture is essential for reducing atmospheric CO2 but is limited by efficiency and cost. Here, authors engineer a proton-trap sorbent that enables faster, lower-cost CO2 removal using electric swing adsorption, achieving high uptake and stability.

## Introduction

The imperative to limit global warming necessitates large-scale removal of atmospheric carbon dioxide (CO_2_), positioning Direct air capture (DAC) as a critical technology for achieving net-zero emissions^1–3^. While several DAC approaches are under development, their widespread deployment is hindered by high energy consumption, complex steam systems, and prohibitive costs, which fundamentally challenge their scalability and economic viability^4,5^. The International Energy Agency projects an urgent need for a nearly 2,000-fold expansion in DAC capacity by 2030, demanding immediate breakthroughs in both materials science and process engineering^6^.

**Fig. 1:**
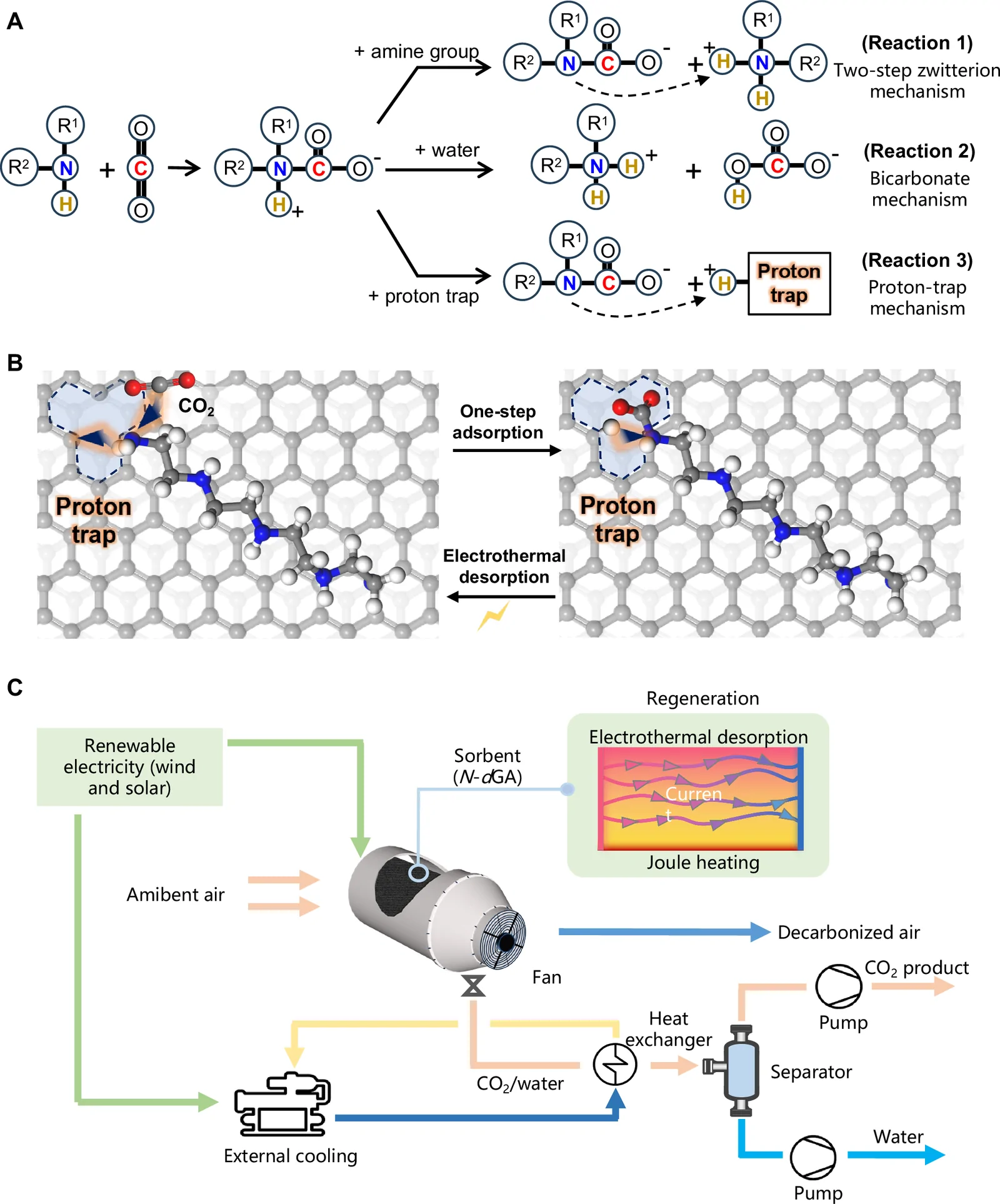
Schematic illustration of the proton trap-based process for atmospheric CO_2_. **A** Two-step zwitterion, bicarbonate and proton-trap mechanisms. **B** CO_2_ capture and release of *N*-*d*GA via a proton-trap mechanism. **C** Complete process for stable and efficient atmospheric CO_2_ capture.

Electric swing adsorption (ESA) has emerged as a promising DAC strategy, leveraging renewable electricity for sorbent regeneration via Joule heating, thereby eliminating the need for steam^7,8^. This offers a pathway to simpler, more compact DAC systems with potentially lower energy footprints than conventional Temperature Swing Adsorption (TSA), which typically require 7.2 to 9.5 GJ t^−1^ CO_2_^9,10^. The sorbents for ESA are primarily carbon-based materials with high electrothermal conversion efficiency, such as graphene^11^, carbon nanotubes^12^, and carbon fibers^13–15^. While the CO_2_ adsorption capacity is mainly determined by amine-functionalized molecules modified on the surface or in the pores. It is now widely recognized that increasing the CO_2_ capacity of ESA sorbent is the most direct route to enhancing the energy efficiency for DAC^16^. However, current ESA sorbents, typically amine-functionalized carbon materials, are fundamentally constrained by the conventional zwitterion mechanism for CO_2_ capture^17–19^. This mechanism (Fig. 1A, Reaction 1) dictates that two amine groups are required to capture one CO_2_ molecule – one to form a carbamate or zwitterion, and another to accept a proton^20–22^. This inherent 2:1 amine:CO_2_ stoichiometry not only halves the theoretical CO_2_ uptake capacity but also presents thermodynamic and kinetic hurdles for efficient capture from dilute air (400 ppm CO_2_) and for rapid sorbent regeneration^23^. While humid conditions can promote bicarbonate formation (Fig. 1A, Reaction 2), appearing to improve stoichiometry, this often involves undesirable water management^24^ and can negatively impact material lifetime and energy efficiency^25^. The inherent flaw of the conventional mechanism constricts the adsorption efficiency of current ESA sorbents, rendering them incapable of overcoming the thermodynamic limitation to CO_2_ capture from dilute gas mixtures. This fundamental limitation has significantly hampered the progress of ESA-DAC towards practical, large-scale application.

Here, we present a design method for ESA sorbent via engineering a point defect-mediated proton trapping network. It redirects proton management during CO_2_ capture effectively, enabling all amine functional groups to directly bind CO_2_ in a 1:1 stoichiometric ratio (Fig. 1A, Reaction 3). Point defects introduced into carbon frameworks can create three-dimensional unpaired electrons via *sp*^3^ hybridization, according to recent reports^26,27^. This enhances the ability of the materials to anchor protons vertically through electrostatic attraction^28^. Thus, point defects can serve as effective proton traps theoretically. Based on this, we designed and fabricated an amine-functionalized point defect-consisting graphene aerogel, named *N*-*d*GA (Fig. 1B). Thorough density functional theory (DFT) and ab initio born-oppenheimer molecular dynamics (AIMD) calculations elucidated the mechanistic transition from the conventional zwitterionic mechanism to a proton-trap mechanism mediated by point defects, while simultaneously investigating the influence of water molecules on the proton-trap mechanism. To further validate this mechanism, we determined the amine-CO_2_ binding stoichiometry via quantitative experiments. The dynamic variations of amine groups and point defects during CO_2_ adsorption-desorption processes were elucidated by Raman spectroscopy. The DAC capacity and stability of *N*-*d*GA were measured in simulated real-world operating environments. Finally, the industrial scalability and cost of the ESA-DAC system using *N*-*d*GA were examined in detail through techno-economic analysis (Fig. 1C).

## Results

### Engineering a point defect-mediated proton-trap mechanism

Building on the theoretical insights from material synthesis^29^, we computationally designed the atomic structure of point defects on *N*-*d*GA, and successful fabricated materials with proton traps. Reduced density gradient analysis reveals a significant electrostatic attraction between the point defects and protons (Supplementary Fig. 1). The gradual decrease in the distance between point defects and protons has led to a 33.3% increase in the attractive force between them, and this attraction reaches its peak when the modified amine is configured as *penta*-amine (*N5*-*d*GA). Electrons transfer from the C atom at the point defects to the H proton of the amine group (Supplementary Fig. 2), enabling point defects to act as proton trap and accept protons from zwitterions. Consistent with theoretical predictions, the experimental results confirmed that *N*5-*d*GA exhibited the highest CO_2_ adsorption capacity under a pure CO_2_ atmosphere compared with all other samples (Supplementary Fig. 3). The precise location of *penta*-amine molecules at the point defects allowed the efficient controlling of amine loaded on *N*5-*d*GA to optimize CO_2_ adsorption (Supplementary Fig. 4). The maximum CO_2_ adsorption capacity under a pure CO_2_ atmosphere reached 12.6 mmol g^−1^ with an amine loading of 66.6 wt.% (Supplementary Fig. 5), which implies that amine groups of *N*5-*d*GA can adsorb CO_2_ in a stoichiometry of 1.1:1, approximately twice the capacity of the aqueous amine counterpart (Supplementary Figs. 6 and 7).

**Fig. 2:**
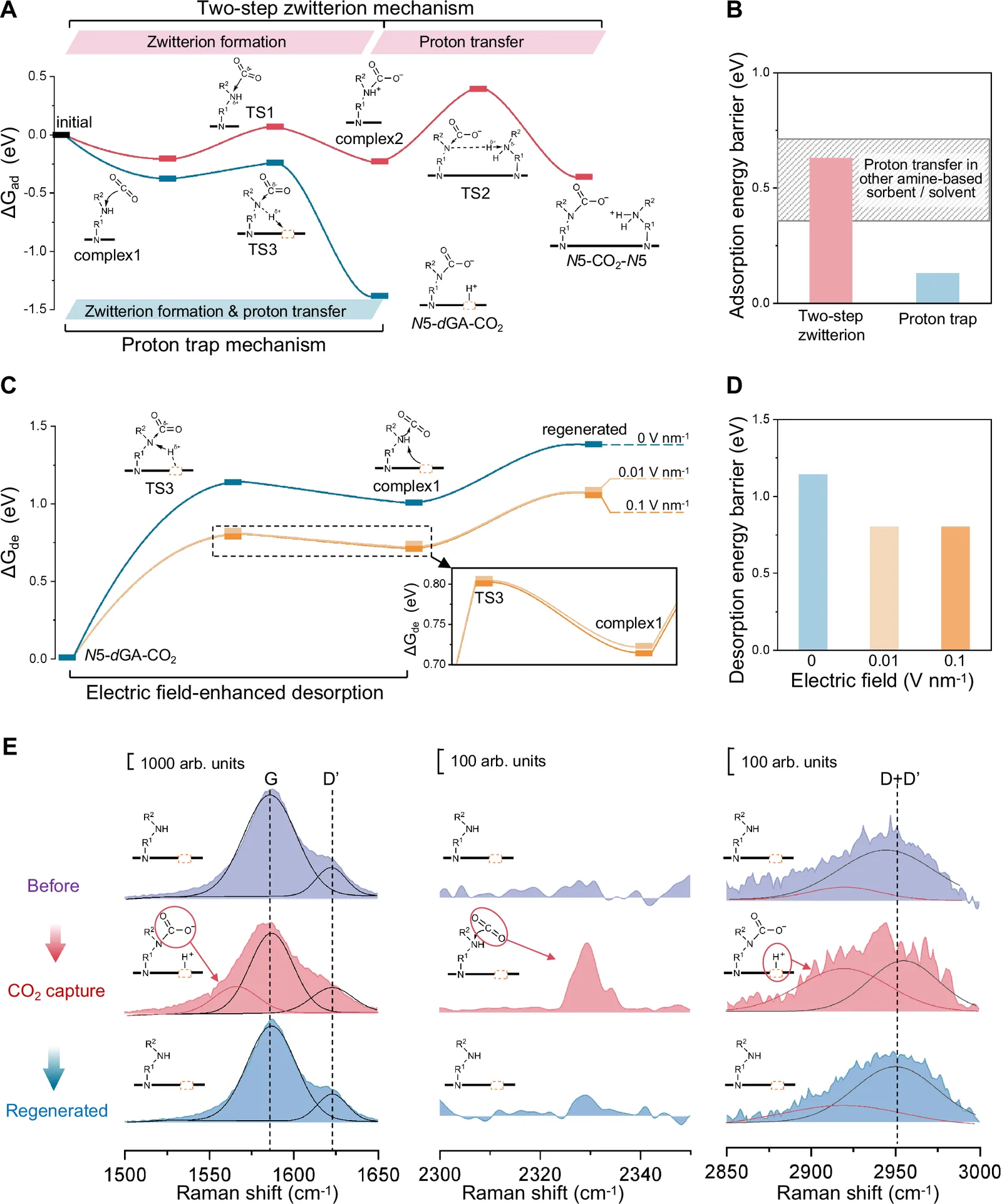
CO_2_ adsorption and desorption pathways of *N*5-*d*GA. **A** The CO_2_ adsorption pathway and (**B**) adsorption energy barrier of *N*5-*d*GA via two-step zwitterion mechanism and proton trap mechanism. **C** The CO_2_ desorption pathway and (**D**) desorption energy barrier of *N*5-*d*GA under different electric field strengths. Inset image of (**C**): An enlarged view of TS3 and complex 1. **E** Amine-defect synergistic enhancement of proton transfer on *N*5-*d*GA revealed using the Raman spectra. Source data for (**A**–**D**) are provided as a Source Data file.

The one-step point defect-based proton-trap mechanism has thermodynamic and kinetic advantages over the conventional two-step zwitterion mechanism. To illustrate the enhancing effect of proton traps on the adsorption reaction, the the process in which CO_2_ undergoes nucleophilic attack by amine groups of *N*5-*d*GA was simulated using DFT calculations. Proton trap-based CO_2_ adsorption proceeds via a one-step mechanism, distinct from the conventional two-step zwitterion mechanism. The proton transfer from the zwitterion to another amine group is the rate-limiting step of the two-step zwitterion mechanism, with an energy barrier of 0.63 eV, consistent with the reported values of 0.3–0.7 eV^30,31^ (Fig. 2A). Alternately, the energy barrier is reduced by 79% when point defects act as the proton acceptor instead of the amine group (Fig. 2B). The potential energy of the adsorbed product (*N*5-*d*GA-CO_2_, −1.38 eV) is ~1 eV lower than that of the conventional product (*N*5-CO_2_-*N*5, −0.37 eV).

The proton traps exhibit rapid regeneration under the influence of an electric field, demonstrating a high suitability for the ESA process. Figure 2C shows that the presence of an electric field can effectively reduce the energy barrier of the dissociation of protons attached to point defects. The energy barrier of CO_2_ desorption is reduced by 28.9% under an electric field intensity of 0.01 V nm^−1^ (Fig. 2D). Further increase in the electric field strength to 0.1 V nm^−1^ reduces the energy barrier by 1.2%. Thus, in this case, a reduction of 22.6% of regeneration potential energy was achieved compare with that without an electric field. According to the proton-coupled electron transfer effect^32^, electrons from the electric field render the fast proton dissociation from point defects to amine group, accompanied by the easier release of CO_2_ and regeneration of point defects.

The proton-trap mechanism was experimentally validated by confocal Raman spectroscopy (Fig. 2E). Although the symmetric stretching vibration of COO^-^ (～1380 cm^−1^) is obscured by the D band of graphene (1270 ~ 1450 cm^−1^), several diagnostic spectral features are observed upon CO_2_ adsorption. Specifically, the new peaks at 1570 cm^−1^ and 2348 cm^−1^ are assigned to CO_2_-amine interaction products^33^. Additionally, a distinct C-H stretching peak develops at 2920 cm^−1^, indicating protonation of the point defect site^34,35^. Notably, the CO_2_-derived species and the C-H bond exhibit concerted emergence during adsorption and synchronous disappearance during regeneration, respectively. Supplementary Fig. 8 shows the DFT-based Raman simulations for *N*5-*d*GA before and after CO_2_ adsorption. CO_2_ adsorption leads to an enhanced C-H stretching feature in the ~2900 cm^−1^ region. This result is consistent with the experimentally observed Raman peaks, supporting the assignment of this feature to protonation of the point defect sites during CO_2_ adsorption. In addition, the result of electron paramagnetic resonance (EPR) strongly supported the proton-trap mechanism. After CO_2_ capture, the EPR signal at g ≈ 2.003 is attenuated (Supplementary Fig. 9), indicating a decrease in point defect-related spin density^36^. After Joule heating regeneration, the EPR signal recovers to nearly its initial intensity. This attenuation and subsequent recovery phenomenon confirms the reversible activation of point defect-based proton traps.

### CO_2_ capture from dilute air via proton trapping

**Fig. 3:**
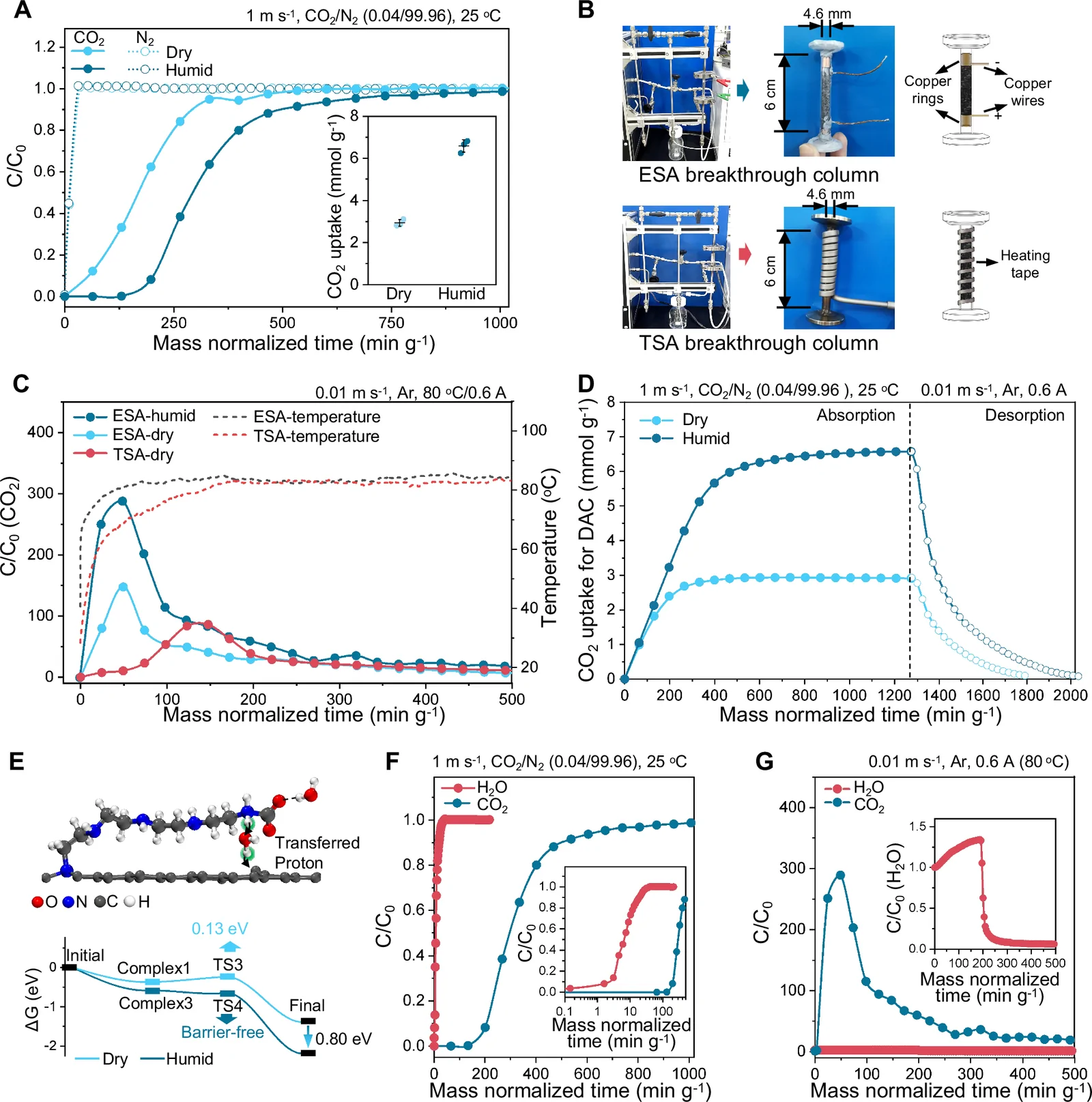
ESA performance of *N-d*GA for DAC. **A** Breakthrough curves and CO_2_ uptakes adsorbed directly from the air of *N*5-*d*GA under dry and humid conditions. Inset image of (A): CO_2_ uptakes of *N*5-*d*GA under dry and humid conditions. **B** The breakthrough columns for ESA and TSA. **C** CO_2_ desorption and temperature curves of *N*5-*d*GA via ESA and TSA. **D** CO_2_ adsorption and desorption process of *N*5-*d*GA under dry and humid conditions. **E** Schematic diagram and the ΔG plot of *N*5-*d*GA for CO_2_ adsorption under humid conditions. **F** Breakthrough adsorption and **G** electrothermal desorption curves of CO_2_ and water. Inset images of (**F**) and (**G**): Enlarged views of the water breakthrough adsorption curve in (**F**) and the water electrothermal desorption curve in (**G**), respectively. Dry and humid conditions correspond to 0% RH and 80% RH, respectively. The error bars in (**A**) correspond to the standard deviation of three independent measurements. Data are presented as mean values ± s.d. Source data for (**A**, **C**–**G**) are provided as a Source Data file.

*N*5-*d*GA demonstrated high adsorption capacity in DAC experiments under real conditions benefiting from the one-step adsorption mechanism. All DAC experiments were conducted under real conditions with an industrial-level gas velocity of 1 m s^−1^. *N*5-*d*GA exhibited 2.93 mmol g^−1^ CO_2_ uptake for DAC under dry conditions (Fig. 3A). The presence of water vapor enhanced the CO_2_ adsorption capacity of amine-based sorbents; CO_2_ retaining time was 212% longer at 80% relative humidity (RH) than under dry conditions (0% RH). Consequently, CO_2_ adsorbed directly from the air by *N*5-*d*GA under humid conditions increased to 6.57 mmol g^−1^, which was higher than that of the current state-of-the-art sorbents in Supplementary Table 1, and 28.8% higher than the current record^3^. It should be noted that the ultrahigh porosity of the graphene aerogel (>98%) results in a reduced CO_2_ capacity per unit volume of 52.6 mol m^−3^ for *N*5-*d*GA. The nitrogen (N_2_) retention time by *N*5-*d*GA is shorter than that of CO_2_, demonstrating high adsorption selectivity under dry and humid conditions. The kinetic studies can be found in Supplementary Fig. 10, and the high correlation coefficients (R^2^ > 0.99) indicate a good fit of the model. The results demonstrate that the rate constant is 12% lower in humid air than in dry air. This behavior is consistent with the mass transfer process of water-mediated CO_2_ adsorption on amine-functionalized sorbents^37^.

The high electrical conductivity ensures the rapid electrothermal desorption of CO_2_ in *N*5-*d*GA. Although the conductivity of graphene aerogel is reduced owing to the presence of point defects and amines, *N*5-*d*GA packed into the breakthrough column still exhibits a conductivity of 409 S m^−1^ (Supplementary Fig. 11). The electrical conductivity of graphene aerogels increases significantly upon compression due to the formation of additional contact points between graphene sheets (Supplementary Fig. 12). As shown in Supplementary Fig. 13, the electrothermal temperature of *N*5-*d*GA can be rising from 60 ^o^C to 129 ^o^C, by control the current changes from 0.5 A to 0.8 A. Actually, a current of 0.6 A is sufficient to consistently heat *N*5-*d*GA to the desired desorption temperature of 80 °C. More detailed thermal analysis was conducted on the bulk samples using thermal imaging technology, which shows an overall uniform heating under stable electrical power (Supplementary Fig. 14-16).

The electrothermal desorption process of *N*5-*d*GA outperforms conventional the externally induced thermal process, requiring significantly less time to release CO_2_ at the same temperature. The photos of test devices were shown in Fig. 3B. Figure 3C shows that the time required for releasing 95% ESA-based CO_2_ is 48% less than that of TSA-based CO_2_. 70% of ESA-based CO_2_ ( < 200 min g^−1^) is rapidly desorbed, while 25% of the residual CO_2_ is slowly released later. The desorption peak of CO_2_ adsorbed under humid conditions is asymptotically distributed with a long tail owing to the desorption of water and CO_2_, as reported in earlier studies^38^. *N*5-*d*GA desorbs ESA-based CO_2_ completely (Fig. 3D). CO_2_ uptake returns to the initial value under both dry and humid conditions after adsorption–desorption cycles.

*N*5-*d*GA transfers protons faster through water hydrogen bond networks without directly involving water in the chemical reaction, demonstrating good water resistance under humid conditions. Based on the DFT calculation, water molecules accelerate proton transfer between amine groups and point defects. The energy barrier of proton transfer reduces from 0.13 eV to 0 eV (Fig. 3E). Water molecules capable of forming hydrogen bonds with the adsorbed CO_2_ reduce the potential energy of adsorption product by 0.80 eV, resulting in the better adsorption behavior^39^. Water improves the intrinsic CO_2_ adsorption reaction by facilitating proton transfer and stabilizing the adsorbed state, but also increases macroscopic mass-transfer resistance within the fixed bed. The competitive adsorption of CO_2_ and water vapor on *N*5-*d*GA was investigated. CO_2_ and water vapor were simultaneously introduced into a dry sorbent at 80% RH. The breakthrough adsorption curves of water and CO_2_ were obtained in Fig. 3F. The breakthrough time of water is significantly shorter than that of CO_2_. Complete electrothermal desorption of water was achieved within 200 min g^−1^ (Fig. 3G), indicating that *N*5-*d*GA has a weak retention capacity for adsorbed water.

### Stability under industrially relevant operational conditions

**Fig. 4:**
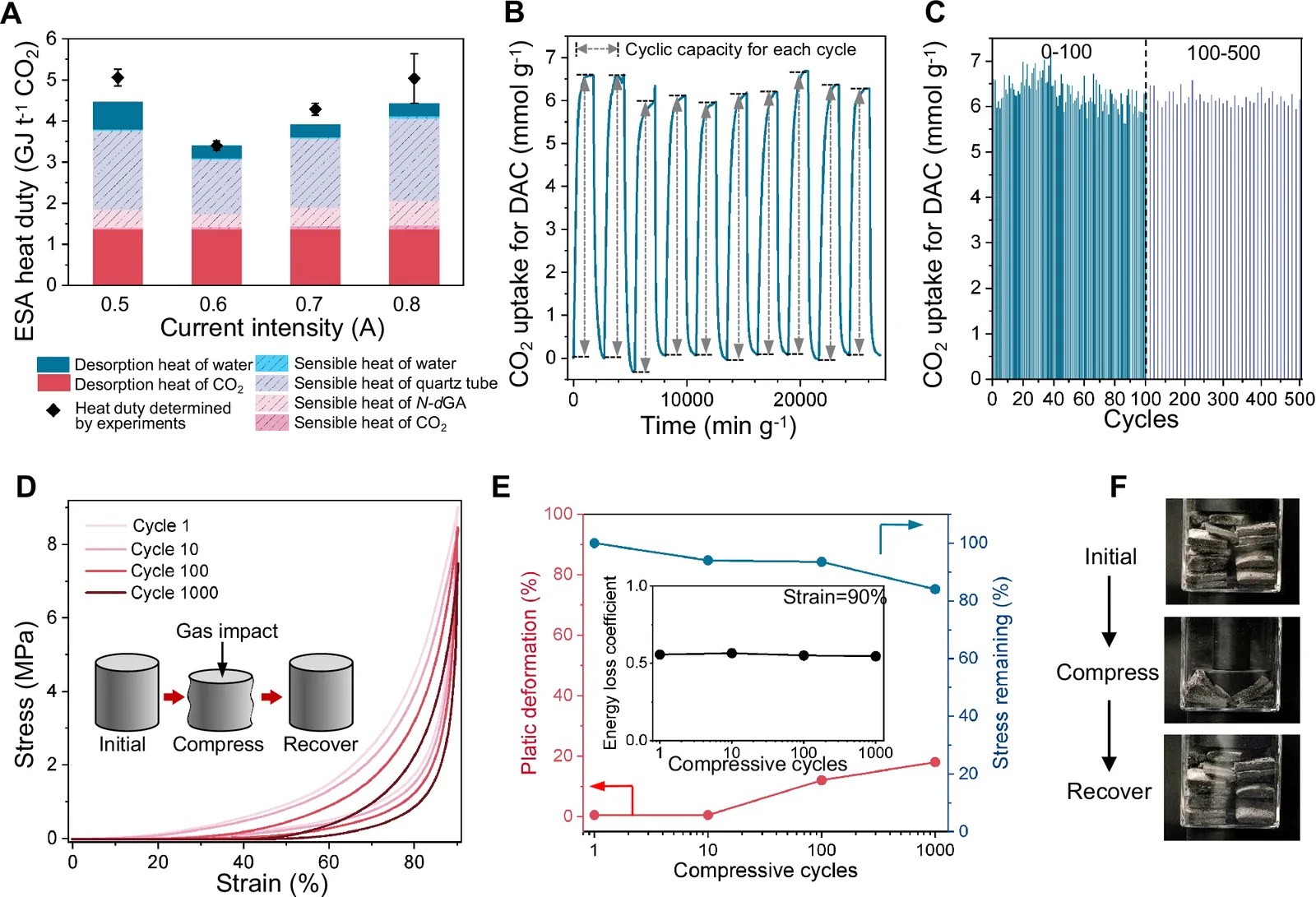
Chemical and mechanical stability of *N*-*d*GA. **A** Comparison of calculated and experimental ESA heat duties for CO_2_ desorption using *N*5-*d*GA. The *N*5-*d*GA adsorbs CO_2_ to saturation in humid air. **B** 10 cycles of CO_2_ adsorption–electrothermal desorption. **C** CO_2_ uptake of *N*5-*d*GA during 500 adsorption-electrothermal desorption cycles. CO_2_ uptake values were recorded for each cycle from 0 to 100 cycles and at 10-cycle intervals from 100 to 500 cycles. **D** The compressive curves, (**E**) plastic deformation and stress remaining of *N*5-*d*GA after 1000^th^ cycle at 90% strain. The inset is the energy loss coefficient. **F** Digital photos of *N*5-*d*GA during 1000^th^ cycle of compression. The error bars in (b) correspond to the standard deviation of three independent measurements. Data are presented as mean values ± s.d. Source data for (**A**–**E**) are provided as a Source Data file.

*N*5-*d*GA shows pronounced industrial viability, thanks to its low energy consumption and high cyclic stability. The ESA heat duty of *N-d*GA was quantitatively assessed across varying current densities and experimentally validated (Fig. 4A). While elevating the current intensity from 0.6 A to 0.8 A sharply increases sensible heat, reducing it to 0.5 A paradoxically raises both sorbent sensible heat and water desorption heat. This discrepancy arises from a 50% reduction in the amount of CO_2_ desorbed at 0.5 A (Supplementary Tables 2 and 3), necessitating a larger sorbent mass to maintain equivalent CO_2_ cyclic capacity. Notably, the minimum ESA heat duty of 3.4 GJ·t^−1^ CO_2_ occurs at 0.6 A, which is lower than the widely recognized energy consumption for DAC^40^ (Supplementary Table 1). This reduced heat duty can be attributed to direct Joule heating within the conductive adsorbent bed and electric-field-assisted CO_2_ desorption during the ESA process. Cyclic adsorption–desorption experiments were conducted to verify the cyclic stability of *N*5-*d*GA, which were repeated with adsorption at 25 °C and 80% RH, and electrothermal desorption at 0.6 A and 0% RH. Each cycle consisted of saturated adsorption with 1700 min g^−1^ followed by regeneration with 1000 min g^−1^. The fluctuation range of CO_2_ absorption is within ±3% in 10 consecutive cycles, indicating stable adsorption-regeneration behavior (Fig. 4B). The results of 100 adsorption–desorption cycles are shown in Fig. 4C. The CO_2_ uptake of the as-prepared sorbent marginally decreases by 3.7% after the 100^th^ cycle compared to the initial uptake, accompanied by virtually unchanged amine loading. More importantly, the CO_2_ uptake remains essentially stable from 100 to 500 cycles, with no further measurable decline within experimental fluctuation. This indicates that the sorbent reaches a stable cyclic working state after the initial conditioning period. The small initial decrease is mainly attributed to bed-level conditioning during the early adsorption-desorption cycles. Under repeated gas flow and Joule heating regeneration, the packed bed may undergo minor redistribution before reaching a stable packed configuration. Post-cycling characterization further supports the material’s robustness. TG analysis shows that the change in amine loading is less than 2%, while XPS N1s spectra and Raman spectroscopy confirm that the covalent grafting between amine groups and *d*GA remains intact, indicating no significant degradation during repeated electrothermal regeneration (Supplementary Figs. 17–19).

The high-velocity gas flow for DAC demands substantial material strength of sorbents. The above results indicate that *N*5-*d*GA exhibits the chemical and mechanical stability. The micron-level cavity of the material retains its original hyperboloid structure, indicating strong robustness^41^. *N*5-*d*GA exhibits strong elasticity when subjected to a 90% compression strain for 1000 cycles (Fig. 4D). It exhibits a high degree of recovery (84% remaining stress) with minimal relaxation (18% plastic deformation) and maintains a slight variation of the energy loss coefficient (0.05) (Fig. 4E). No fragment is detached from the glass exterior (Fig. 4F), rendering *N*5-*d*GA capable of withstanding the formidable impact of the high-velocity gas flow. The appearance of the sample before and after undergoing 1000 compression tests shows imperceptible differences.

### Pathway to economic viability

**Fig. 5:**
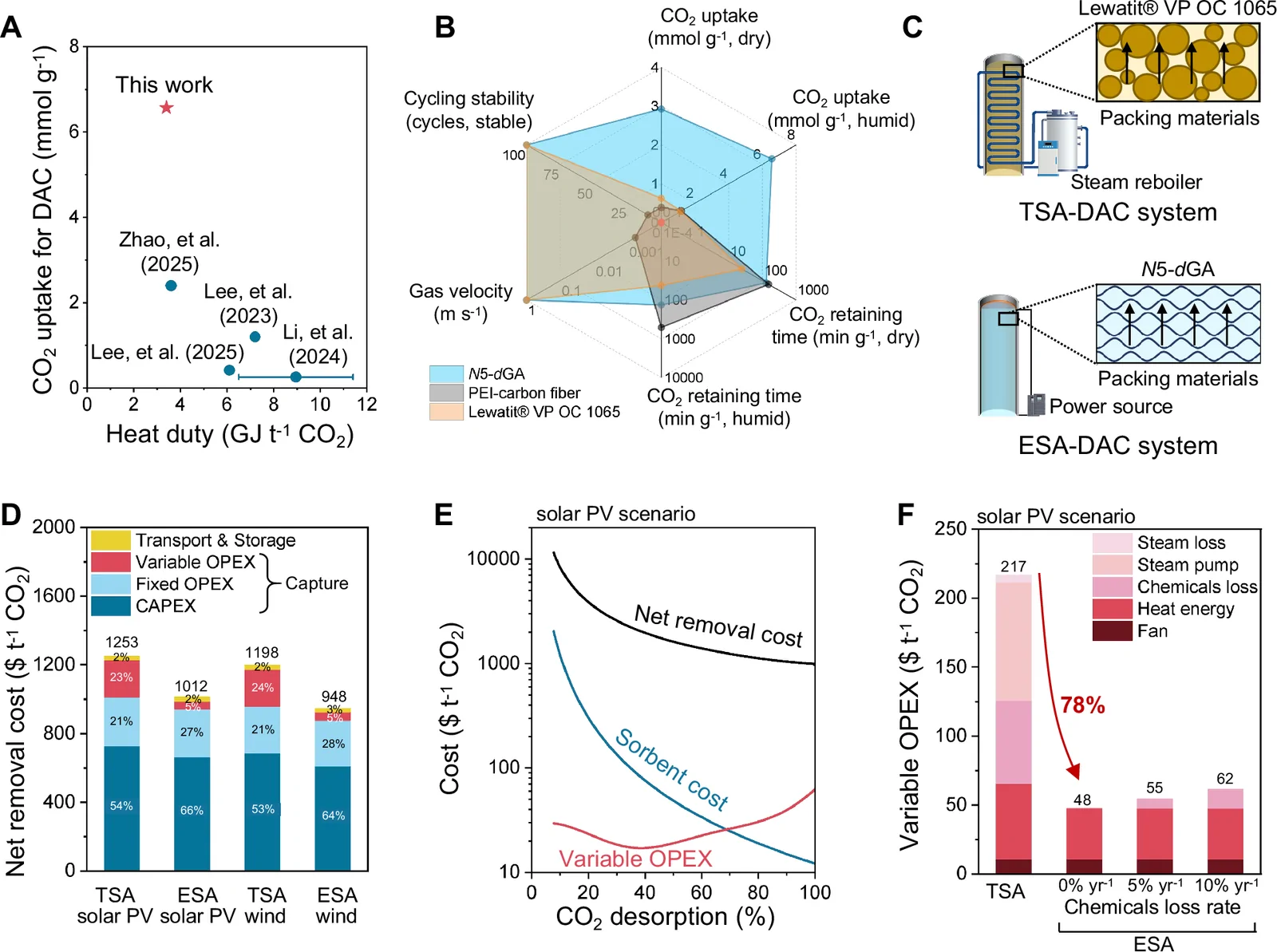
Techno-economic analysis of ESA-DAC system. **A** A comparison of CO_2_ uptake versus regeneration energy for *N*5-*d*GA and reported Joule heating-enabled sorbents, all DAC tested under ambient conditions (400 ppm CO_2_, 25 ^o^C, 1 atm). The corresponding data can be found in Supplementary Table 1. **B** The CO_2_ uptake, CO_2_ retaining time, cycling stability and gas velocity of different sorbents at 25 ^o^C. Dry and humid conditions correspond to 0% RH and 80% RH, respectively. **C** The schematic diagram of TSA- and ESA-DAC systems. **D** Cost for TSA- and ESA-DAC systems categorized by their electricity sources of a 4000 t a^-1^ scale. The stacked bars represent the CO_2_ net removal cost. The amount of CO_2_ desorbed is 95%. **E** Relationship between DAC cost by solar PV and amount of CO_2_ desorbed in the ESA-DAC system. **F** Detailed variable OPEX for CO_2_ capture using TSA- and ESA-DAC systems by solar PV of a 4000 t a^-1^ scale. The amount of CO_2_ desorbed is 95%. The data of TSA-DAC system was reported by Sievert et al.^44^. Source data for (**A**, **B**, **D**–**F**) are provided as a Source Data file.

A comparative performance assessment (Fig. 5A) underscores *N*5-*d*GA’s clear advantages over reported Joule heating-enabled sorbents for DAC. Figure 5B shows the gap between *N*5-*d*GA and two typical sorbents (Lewatit® VP OC 1065 and PEI-carbon fiber)^42,43^ using 6 key indicators, including CO_2_ uptake (under dry and humid conditions), CO_2_ retention time (under dry and humid conditions), cycling stability, and gas velocity. *N*5-*d*GA outperforms the PEI-carbon fiber in 4 indicators—CO_2_ uptake under dry and humid conditions, cycling stability, and gas velocity—although it lags slightly in CO_2_ retention times. Compared to the commercial DAC sorbent Lewatit® VP OC 1065, *N*5-*d*GA is more competitive in all indicators.

The ESA-DAC system using *N*5-*d*GA exhibits a lower projected CO_2_ capture cost than conventional TSA-DAC systems based on techno-economic analysis. The technology cost forecast of TSA- and ESA-DAC systems of a 4000 t a^−1^ scale, using various renewable electricity sources, is based on an empirical analysis reported by Sievert et al^44^. The CO_2_ net removal cost includes the capital expenditure (CAPEX), such as the cost of initial equipment and sorbents, fixed operational expenditure (OPEX), variable OPEX and the cost of CO_2_ transport & storage. The packing materials used in TSA- and ESA-DAC systems are Lewatit® VP OC 1065 and *N*5-*d*GA, respectively. The derivation of each term can be found in Supplementary Information, section techno-economic analysis and Supplementary Tables 4-8. Compared to TSA-DAC, the ESA-DAC system reduces the CO_2_ net removal cost by 19-21%. This substantial cost advantage is primarily driven by significant reductions in initial equipment and pump operating costs, as shown in Fig. 5C, due to the elimination of steam systems required in traditional TSA systems. Specifically, Fig. 5D demonstrates a pronounced decrease in the proportion of variable OPEX for CO_2_ capture, from 23-24% in TSA-DAC down to only 5% in ESA-DAC. In comparison, capital expenditures (CAPEX) exhibit a slight decrease. Although the unit cost of the sorbent *N*5-*d*GA is somewhat higher (approximately 1.12 times that of Lewatit® VP OC 1065), overall equipment investment decreases modestly due to the reduced sorbent dosage and system complexity.

It is noted that the reduction in net removal costs of ESA-DAC system mainly comes from the decrease in variable OPEX for CO_2_ capture. This is a direct result of the ESA system’s ability to operate without steam handling, which significantly lowers energy consumption and associated maintenance costs. Under the solar PV scenario, the variable OPEX for ESA-DAC CO_2_ capture follows a U-shaped trend with increasing desorption capacity, reaching a minimum of $17 t^−1^ CO_2_ at the desorption amount of 40% (Fig. 5E). However, at this point, the initial sorbent cost and net removal cost are $79 t^−1^ CO_2_ and $2006 t^−1^ CO_2_, respectively. Therefore, considering both CO_2_ uptake and heat duty, setting the CO_2_ desorption amount to 95% is more favorable for achieving an optimal balance between efficiency and cost for ESA-DAC. As shown in Fig. 5F and Supplementary Fig. 20, the variable OPEX for CO_2_ capture using ESA-DAC system in optimal conditions is $48-62 t^−1^ CO_2_ using various renewable electricity sources, representing a maximum reduction of 78% compared to TSA-DAC system. Furthermore, the high CO_2_ uptake capacity of *N*5-*d*GA combined with its low ESA heat duty and stable cycling performance under humidity conditions ranging from 0 to 80% and at industrial gas flow velocities, further strengthens the economic viability of the ESA-DAC system.

## Discussion

This work presents an integrated study of Direct Air Capture, connecting fundamental sorbent design with process-level techno-economic analysis of an ESA system. We developed an efficient sorbent, *N*5-*d*GA, based on the mechanistic understanding of point defect-based proton traps. Unlike the conventional two-step zwitterion mechanism, which imposes a limiting 2:1 amine:CO_2_ stoichiometry, CO_2_ adsorption in *N*5-*d*GA proceeds via a proton-trap pathway. Here, engineered point defects, rather than secondary amine groups, act as the primary proton acceptors during zwitterion deprotonation. It unlocks the full potential of amine functionalities, enabling them to capture CO_2_ in a highly efficient 1:1 stoichiometry. The practical outcome in this work is a CO_2_ uptake of 6.57 mmol g^−1^ from dilute 400 ppm CO_2_ streams, coupled with a low regeneration heat requirement of 3.4 GJ t^−1^ CO_2_ (for 95% release), thereby addressing two major bottlenecks that have historically hindered amine-based DAC technologies. More broadly, the implications of the proton-trap strategy are not restricted to intrinsically conductive carbon sorbents. Defect-engineered porous materials with limited electrical conductivity could also be used into the ESA process by coupling them with conductive additives. In such hybrid sorbents, point defects may serve as proton-acceptors to promote assist amine-based CO_2_ chemisorption, while the conductive component provides electrical heatability for Joule heating regeneration. This approach opens a broader materials-design space for next-generation amine-functionalized sorbents.

The implications of this mechanistic finding extend directly to economic feasibility. Our techno-economic analysis forecasts that an ESA-DAC system utilizing *N*5-*d*GA can achieve variable OPEX for CO_2_ capture of $48-62 t^−1^, a transformative 78% reduction compared to established TSA-DAC benchmarks for a 4000 t a^−1^ facility. This dramatic cost improvement is a direct consequence of the proton-trap mechanism, which synergistically enhances CO_2_ adsorption capacity while minimizing regeneration energy demands—the two most critical levers for DAC cost-effectiveness. The high stability of *N*5-*d*GA under industrially relevant thermal, chemical, and mechanical stresses further bolsters its practical applicability and long-term economic performance.

These findings highlight the potential of proton-trap engineered ESA-DAC for large-scale atmospheric CO_2_ removal. The ability to achieve high capture efficiencies with significantly lower energy inputs and projected costs, all while operating without steam system, offers a compelling pathway towards gigaton-scale DAC deployment, particularly in regions with abundant renewable electricity but scarce water resources. Currently, the global energy structure is accelerating the transformation towards wind and solar renewable energy. In China, curtailed wind and solar electricity reached 67.4 TWh in 2024, according to the National Energy Administration. Furthermore, the International Energy Agency predicts that the total amount of wind and solar curtailment may reach 2000 TWh in 2030^45^. Wind and solar power are often curtailed in regions with abundant renewable energy, such as deserts and plateaus. The amount of electricity generated can exceed the capacity of the local grid or transmission system. As an electrically driven process, ESA-DAC can direct utilize these curtailed renewable electricity for sorbent regeneration. As an electrically driven process, ESA-DAC can directly utilize these curtailed renewable electricity for sorbent regeneration. Its modular design, low water demand, and lack of steam or hot-water infrastructure further support distributed deployment in these regions.

These curtailments often occur in areas such as deserts and plateaus, which are rich in wind and solar energy but lack infrastructure. ESA technology offers several advantages for DAC, including the ability to rapidly and precisely control temperature, a compact module design, the elimination of the need for steam or hot water infrastructure, and the capacity to dynamically respond to intermittent renewable power. These features enable ESA-DAC to achieve distributed deployment and to flexibly offset green power fluctuations.

This work not only provides a material solution but also establishes a design approach for CO_2_ sorbents that may facilitate further exploration of gas separation and catalysis where proton management is key. Future research should focus on scaling up *N*5-*d*GA production, optimizing contactor designs for ESA, and conducting extended pilot-scale demonstrations to validate long-term performance under real-world atmospheric variations, thereby accelerating the transition of this technology towards industrial implementation and its crucial role in global carbon neutrality strategies.

## Methods

### Chemicals

Chemicals, including *dual*-amine (ethylenediamine, gas chromatography grade), *tetra*-amine (triethylenetetramine, analytical reagent grade), *penta*-amine (tetraethylenepentamine, technical grade), *hex*-amine (pentaethylenehexamine, technical grade), etc., were purchased from Aladdin. The aqueous GO dispersions were obtained from Hangzhou Gaoxi Technology Co., Ltd. The deionized water (18.2 MΩ·cm) was used throughout all experiments.

### Preparation of samples

GA is prepared by hydroplastic foaming method by aqueous GO dispersions (12.55 mg g^−1^) and ultrapure water at a volume ratio of 1:1 and stirring for 10 min to prepare a homogeneous dilution of 6.275 mg g^−1^ ^41^. The graphene oxide flakes are obtained by taking 5 g of the dilution solution and spreading it on a 33 mm diameter plastic Petri dish and drying it in an oven at 60 ^o^C for 6 h. Take 10 mL of 80 vol% hydrazine hydrate solution and add 16.67 mL of water to configure 30 % hydrazine hydrate solution. The graphene oxide flakes are immersed in the 30 vol% hydrazine hydrate solution at room temperature and foamed for 3 h. The flakes are gradually fluffed to a foamy state. The foamed sample is completely submerged in excess of anhydrous ethanol and soaked for three hours, with this process repeated three times using fresh anhydrous ethanol each time. Then replace anhydrous ethanol with n-hexane and repeat the above steps. Finally, the samples are dried overnight in an oven at 60 ^o^C to produce the GA samples for subsequent thermal reduction treatment.

Thermal reduction treatment of GA: Take 20 mg of GA sample into corundum porcelain boat and load it into the corundum tube of tube furnace. Oxygen is completely removed from the tube by argon (Ar)-vacuum cycle operation for 3 times, and the temperature is increased to 1600 °C at a rate of 10 ^o^C min^−1^ under the protection of 20 mL min^−1^ Ar gas and maintained for 2 h. Subsequently, the temperature is naturally cooled down to produce the GA sample for subsequent plasma treatment. The density of GA is 8 ( ± 2) mg cm^−1^.

Ar plasma treatment of *d*GA: Take 20 mg of thermal reduction GA sample into corundum porcelain boat and load it into the plasma vapor deposition tube. The tube is thoroughly excluded from oxygen by 3 Ar-vacuum cycles and ramped up to 700 °C at a heating rate of 10 ^o^C min^−1^ under 20 mL min^−1^ Ar protection, with the plasma power set to 200 W, the GA samples are subjected to Ar low-pressure capacitively coupled plasma treatment under vacuum conditions at an absolute pressure of less than 30 kPa for various durations (1 ~ 180 min), and the GA surface is bombarded by Ar plasma to create defective sites to produce *d*GA carriers for subsequent polyamino functionalization. The *d*GA samples were transferred to a sealed vessel under an Ar-protective atmosphere at room temperature after Ar plasma treatment.

Amine-functionalization of *N*-*d*GA: The synthesis of *N*-*d*GA is depicted in Supplementary Fig. 21. Pour 25 mL of undiluted amine into 100 mL of Teflon hydrothermal kettle liner and add 20 mg of *d*GA carrier so that the *penta-*amine is thoroughly submerged in the *d*GA carrier, seal it with a stainless steel shell. The sealed reactor was heated at 90 °C for 10 h in a blast oven. The samples are then soaked in 170 mL of anhydrous ethanol for 20 min at 40 ^o^C (without stirring) and finally dried overnight in a blast oven at 80 ^o^C to produce *N*-*d*GA. The resulting *N*-*d*GA is obtained by removing excess amine molecules through ethanol washing, ensuring direct contact between the vast majority of grafted amine groups and CO_2_ during adsorption. Notably, amine loading of *N*-*d*GA was modulated exclusively by varying Ar plasma treatment duration (0–180 min) to regulate defect density of *d*GA.

### Characterization

Point defects were observed via aberration-corrected transmission electron microscopy (AC-TEM) (Supplementary Fig. 22). The rapid variation of the D band to G band intensity ratio (I_D_/I_G_) obtained via Raman spectroscopy indicates that the modulation of Ar plasma treatment duration can precisely tune the defect density of *d*GA (Supplementary Figs. 23–25). Supplementary Fig. 26 shows that the wall thickness of GAs remains intact after amine functionalisation, with no effect on porosity or the macroporous structure. As indicated via field emission scanning electron microscopy (FE-SEM), energy-dispersive X-ray spectroscopy (EDS) and X-ray photoelectron spectroscopy analyses results (Supplementary Figs. 27–30). The amines of *N*-*d*GA are distributed uniformly in *d*GA by forming C–N covalent bonds with point defects. *N*5-*d*GA displays a new peak at ～400.7 eV, characteristic of graphitic nitrogen, accompanied by a marked decrease in its pyrrolic component. This result indicates that the terminal -NH_2_ groups of *penta*-amine undergo covalent bonding with point defects in *d*GA to form stable C-N linkages and generate graphitic nitrogen moieties. Correspondingly, the defect density is reduced as demonstrated by the decrease of the overall I_D_/I_G_ value from 0.6 to 0.55 (Supplementary Fig. 31).

PXRD patterns are obtained in a PANalytical X’Pert PRO diffractometer, using Cu Kα radiation (λ = 1.5406 Å, 40 kV). In order to avoid the effects of preferred crystal orientation, the samples are grounded. The data are recorded from 5 to 60 ° (2θ) with a resolution of 0.01°. X-ray photoelectron spectroscopy (XPS) is tested using an ESCALAB 250 Xi system equipped with a microfocusing monochromatic Al Kα source and high region resolution up to 0.45 eV.

FE-SEM images are obtained using a Hitachi SU8010 field-emission scanning electron microscope. A secondary electron (Everhart-Thornley) detector is used for the image acquisition. An electron acceleration voltage of 3 kV and the resolution of 1.3 nm@1 kV are chosen for the measurements. EDS data were collected using an Oxford Instruments EDS system. The graphene aerogel samples were cut to expose the internal cross-section and directly fixed on a conductive sample holder without conductive coating.

TEM images are obtained using a HT-7700 microscope. An electron acceleration voltage of 120 kV and the point resolution of 0.33 nm are chosen for the measurements.

AC-TEM images were acquired using an aberration-corrected FEI Titan G2 60-300 transmission electron microscope operated at 80 kV. The as-prepared samples were gently dispersed in ethanol, drop-cast onto an ultrathin carbon film TEM grid, and dried before observation.

TGA and DSC experiments and quantification of amine loading are carried out by SDT Q600 instrument. The sample temperature is increased from room temperature to 800 °C at a heating rate of 10 °C min^−1^ under a continuous N_2_ flow of 100 mL min^−1^.

The Raman spectrum is acquired by LabRAM HR Evolution spectrometer with a spectral resolution of 0.65 cm^−1^, a measurement range of 1000 ~ 3000 cm^−1^, and a spatial resolution of 1 μm. The Raman imaging (mapping) used a 10×10 grid to take points, the mapping spatial resolution is 2 μm, and the size of the mapping square area is 20 μm×20 μm. Raman peak fitting was performed using the Gaussian function.

The EPR spectrum is acquired by Bruker EMXplus-6/1 spectrometer at room temperature. CO_2_ adsorption and Joule heating regeneration were performed ex situ on the same sample.

The compressive test is performed on a microcomputer-controlled electronic universal testing machine (RGWT-4000-20, REGER) at a strain rate of 0.25 mm min^−1^.

The time-dependence temperature curves of *N*5-*d*GA are carried out with a homemade four-electrode conductive stage and a thermal imaging camera. The current intensity (0.5 A, 0.6 A, 0.7 A and 0.8 A) is set on the CS2350H electrochemical workstation and the corresponding voltage is recorded. Thermal imaging temperature data processed by FLIR ResearchIR software. The size of tested bulk sample is 3 cm × 1.5 cm × 0.5 cm. The electrical conductivity is determined using the formula σ = l/(R×S), where l represents the length, S the cross-sectional area, and R the electrical resistance of the GAs.

The porosity of *N*5*-d*GA is conventionally calculated from bulk and skeletal densities using the formula: (1-ρ_bulk_/ρ_skeletal_)×100%. The ρ_bulk_ of *N*5*-d*GA was measured to be 27 mg cm^−3^. The skeletal density, accounting for the composite density of graphene and *penta*-amine, was estimated as 1420 mg cm^−3^ based on its mass ratio. Therefore, the porosity of *N*5*-d*GA is 98.1%.

### Calculation

The calculation methods and details of DFT, AIMD, CO_2_ uptake, heat duty and techno-economic analysis are listed in Supplementary Information. Using AIMD and DFT calculations, we evaluated thermodynamically stable structures of point-defective GAs functionalized using diamine (*N*2-*d*GA), tetraamine (*N*4-*d*GA), *N*5-*d*GA, and hexaamine (*N*6-*d*GA).

Conformational analyses (Supplementary Fig. 32-39) revealed the presence of point defects thermodynamically stabilizes amine adsorption, reducing the system potential energy by 0.16-0.27 eV. Furthermore, during functionalization, amines selectively react with reactive moieties adjacent to defects form C-N bonds, corresponding to a Gibbs free energy reduction of −0.77 to −2.30 eV. AIMD results (Supplementary Fig. 40-43) revealed significant bending of amine molecules within 2 ps owing to the presence of multiple flexible C–C and C–N bonds. Reduced density gradient analysis manifests that the interaction between the point defects and protons of the H of amine groups depends strongly on their distance (referred to as the defect distance). The decreasing defect distances of *N*6-*d*GA, *N*4-*d*GA, *N*2-*d*GA, and *N*5-*d*GA are 11.16, 5.50, 5.23, and 3.94 Å, respectively (Supplementary Fig. 44).

Raman spectra were simulated using the PAW-PBE method with Tkatchenko-Scheffler dispersion correction. Phonon calculations and Raman tensor analysis were performed using the finite displacement method in Phonopy. A plane-wave cutoff of 520 eV and a force convergence of 0.01 eV Å^−1^ were used.

## Supplementary information

Supplementary Information

Transparent Peer Review file

## Source data

Source Data file

## Data Availability

The data supporting the findings of this study are available in the Main text, Supplementary Information, and Source data files provided with this paper. Source data for this paper are provided as a Source Data file. Source data are provided with this paper.
