# Supplementary material for: Proton trap engineered electric swing adsorption for scalable and cost-effective direct air capture
Source: Nat Commun. 2026 Jul 21;17:8885. doi: 10.1038/s41467-026-75916-7 (PMC13500661; doi:10.1038/s41467-026-75916-7)
Supplement: Supplementary file 1 [file 41467_2026_75916_MOESM1_ESM.pdf]

Supplementary Information

Proton Trap Engineered Electric Swing Adsorption for Scalable and Cost-Effective Direct Air Capture

Yao Shen, Kai Pang, Weichen Zhao, Liang Chen, Jingkai Zhao, Jiexu Ye, Beini Zhang, Sujing Li, Wei Li, Zhen Xu, Jing Meng, Xiang Gao*, and Shihan Zhang**

1. Supplementary Notes 2
2. Supplementary Figures 10
3. Supplementary Tables 54
4. Supplementary References 62

1. Supplementary Notes

1.1 Theoretical calculation

Density functional theory (DFT) simulation

The geometry optimization and electronic properties of the *N-d*GA structures were performed using the projector augmented wave (PAW) method and Generalized Gradient Approximation-Perdew-Burke-Ernzerhof (GGA-PBE) exchange-correlation functional via Vienna Ab-initio Simulation Package (VASP) software (version 6.1.1 with full LICENSE) [1]. A vacuum region was set to be 20 Å. The electron wave function was expanded using plane waves with an energy cutoff of 400 eV. 3×3×1 Gamma centered Monkhorst-Pack K-point were adopted in the calculation. The convergence standards of energy and force were selected as 10-4 eV and 0.05 eV Å-1, respectively. Electronic isosurfaces and reduced density gradient (RDG) were calculated by Multiwfn software (version 3.6) [2]. Images were processed with VMD software (version 1.9.3) [3].

Ab initio Born-Oppenheimer molecular dynamics (AIMD) simulation

AIMD was performed using the CP2K package (version 2022.1) in Gaussian and plane waves (GPW) method of quickstep module [4]. The electron structure was calculated using the PBE functional together with the Grimme's D3 dispersion correction [5]. The kind of DZVP-MOLOPT-SR basis set was used, and the pseudopotentials within the GTH-PBE were employed [6]. The plane wave cutoff was set to 350 Ry. The temperature was set constant using canonical sampling through velocity rescaling (CSVR) thermostat with a time constant of 100 fs. The integration time step was set to 1 fs in all cases.

Nomenclature: *d*GA: defective graphene aerogel (Ar-plasma treated); *N-d*GA: amine-functionalized *d*GA; *N*5-*d*GA: dGA grafted with *penta*-amine. Other variants (e.g., *N*4-*d*GA, *N*6-*d*GA) follow analogous naming (amine type indicated by numeral).

1.2 CO_2 adsorption measurements.

Static CO_2 adsorption

Due to the extremely low density of aerogel materials, the error of the conventional TGA test method is too large, so a test device was copied from the literature of Dawson et al to determine the mass adsorption capacity of aerogel materials [7]. A 20 mg sample was placed in a Teflon box, sealed around with sealing film, and the initial mass (mg) was recorded. Fill a balloon with 1 L of pure CO_2 gas with valves and fittings (internal pressure about 1 bar). The balloon will be connected to the Teflon box, open the valve and record the time in 15 s, 30 s, 1 min 30 s, 3 min, 6 min, 11 min, 21 min and 31 min were recorded after the change in weight for m_t (mg). The weighing test was completed in a glove box filled with Ar gas throughout, and the water vapor content in the box was less than 0.1 ppm to exclude the possibility of water absorption and weight gain. To confirm reproducibility, each sample was independently tested three times using this method and the mean value was reported. The static CO_2 uptake (q_{static}, mmol g^{-1}) per unit mass of sorbent was calculated as follows:

$$q_{static}=\frac{m_t\text{-}m_{t=0}}{44\times m_{t=0}}\times 1000 \qquad \text{(Equation S1)}$$

CO_2 uptake for DAC

A homemade adsorption breakthrough device was used for the experiment. A quartz tube with an inner diameter of 4.6 mm, a length of 60 mm, and a smooth inner wall without burrs was selected for the breakthrough column. The tested sample was configured into regular cylinders with a diameter of 5 mm for packing into a simulated fixed bed (0.032 g for *d*GA, 0.065 g for *N*5-*d*GA). The fixed bed of the breakthrough column was activated at 80°C under flowing 100 mL min^{-1} dry Ar for at least 12 h. Then, the heat was turned off, and the system was cooled to room temperature. Subsequently, simulated air containing CO_2/N_2 (0.04/99.96) was introduced at a gas flow rate of 1000 mL min^{-1} under dry (0% RH) and humid (80% RH)

conditions, with an outlet flow rate of 999 mL min^{-1}. The water-containing adsorption breakthrough experiment was performed by setting a small bubble bottle with ultrapure water at the front end of the breakthrough column through the CO_2/N_2 gas mixture, and the relative humidity of inlet and outlet gas were measured by Testo 625 hygrometer at room temperature. The CO_2 concentrations in inlet and outlet gas were measured using a GC9790II gas chromatograph with a calibrated CO_2 standard curve. The CO_2 standard curve is updated quarterly. Whether dry or humidified via water bubbler, the gas stream is adjusted and verified to contain exactly 400 ppm CO_2 prior to exposure to the sorbent in every DAC test. The CO_2 uptake (q_{DAC}, mmol g^{-1}) per unit mass of sorbent was calculated as follows:

$$q_{\mathrm{DAC}} = \frac{C_0 Q}{V_{\mathrm{m}} m_{\mathrm{sample}}} \int_0^t (1-\frac{C}{C_0}) dt \qquad \text{(Equation S2)}$$

where Q was the gas flow rate, mL [STP] min^{-1}; t was the adsorption time, min; V_{m} was the the molar volume, mL [STP] mol^{-1}; m_{sample} was the sample weight, g; C and C_0 were the the gas outlet and inlet CO_2 concentration respectively, mmol L^{-1}; and $\frac{C}{C_0}$ was used for gas concentration profiles of adsorption and desorption for plotting all gas species in the same range. The breakthrough and pseudo-equilibrium CO_2 uptake were calculated from the integrated areas of the breakthrough curves until the normalized CO_2 concentration reached 0.05 (C/C_0=0.05) and 0.95 (C/C_0=0.95).

1.3 CO_2 desorption measurements

Externally driven thermal desorption

The temperature swing adsorption (TSA) breakthrough column was covered by heating tape. The TSA breakthrough column was heated to 80°C, while 10 mL min^{-1} Ar gas flowed across the internal *N*5-*d*GA. When the CO_2 concentration from the column decreased to the baseline level, the thermal desorption was terminated.

Electrothermal desorption

The quartz tube was used as electric swing adsorption (ESA) breakthrough column to achieve insulation. Copper rings and wires were used to connect the *N*5-*d*GA to a DC power supply. A 0.6 A current that was based on electrothermal characteristics to achieve 80°C sample temperature was applied to the *N*5-*d*GA along with 10 mL min^{-1} Ar flow. When the CO_2 concentration from the column decreased to the baseline level, the electrothermal desorption was terminated.

1.4 Thermodynamic analysis

Calculated ESA heat duty

The calculated ESA heat duty (E_{cal}, GJ t^{-1} CO_2) based on the methodology outlined in a previously reported reference[8,9]. Specifically, E_{cal} followed the framework comprising desorption heats of CO_2 and water, sensible heats of CO_2, water, sorbent and quartz tube column, as detailed below. The calculation does not account for heat recovery during the cooling step.

$$\text{desorption heat of CO}_2 = \frac{\Delta H_{\text{ads}}}{M_{\text{CO}_2}} \qquad \text{(Equations S3)}$$

$$\text{desorption heat of water} = \frac{r_{\text{H}_2\text{O/CO}_2}\Delta H_{\text{H}_2\text{O}}}{M_{\text{H}_2\text{O}}} \qquad \text{(Equations S4)}$$

where ΔH_{ads} and ΔH_{H2O} were the CO_2 and water adsorption heats respectively, kJ mol^{-1}; M_{CO2} and M_{H2O} were the molar mass of CO_2 and H_2O, g mol^{-1}; $r_{H2O/CO2}$ was the mass ratio of adsorbed water to adsorbed CO_2.

$$\text{sensible heat of CO}_2 = C_{\text{p,CO}_2}\,\Delta T_{\text{sample}} \qquad \text{(Equations S5)}$$

$$\text{sensible heat of water} = r_{\text{H}_2\text{O/CO}_2} C_{\text{p,H}_2\text{O}} \Delta T_{\text{sample}} \qquad \text{(Equations S6)}$$

$$\text{sensible heat of sample} = \frac{\dfrac{m_{\text{graphene}} C_{\text{p,graphene}} + m_{\text{amine}} C_{\text{p,amine}}}{m_{\text{sample}}} \Delta T_{\text{sample}}}{0.001 \times q_{\text{DAC}} M_{\text{CO}_2}} \qquad \text{(Equations S7)}$$

$$\text{sensible heat of column} = \frac{r_{\text{column/sample}} C_{\text{p,quartz}} \Delta T_{\text{column}}}{0.001 \times q_{\text{DAC}} M_{\text{CO}_2}} \qquad \text{(Equations S8)}$$

where $C_{p,i}$ was the heat capacity of i component at constant pressure, kJ kg^{-1} K^{-1}; m_i was the mass of i component, g; ΔT_i was the temperature rise of i component during electrothermal desorption, K; $r_{column/sample}$ was the mass ratio of quartz tube column to sample.

Experimental ESA heat duty

The experimental ESA heat duty (E_{exp} , GJ t^{-1} CO_2) of electrothermal desorption was measured using a custom-built evaluation setup, which consists of a power supply, a four-electrode conductive stage, a thermocouple for monitoring the internal temperature of sorbents, and a FLIR T660 infrared thermal imaging camera for measuring the surface temperature of the quartz tube and the ambient air temperature:

$$E_{\mathrm{exp}} = \frac{W_{\mathrm{ele}} - W_{\mathrm{loss}}}{1000 \times m_{\mathrm{CO_2,de}}} \qquad \text{(Equation S9)}$$

where $m_{\mathrm{CO_2,de}}$ was the CO_2 emissions during desorption, g; W_{ele} and W_{loss} were the electric work and heat loss, J, which can be calculated as:

$$W_{\mathrm{ele}} = \int P\,dt = \int UI\,dt = \overline{P}t \qquad \text{(Equation S10)}$$

$$W_{\mathrm{loss}} = \int \Phi\,dt \qquad \text{(Equation S11)}$$

where P and $\overline{P}$ were electrical power and its average value, W; I was the constant output current, A; U was the voltage, V; The rate of heat flow (Φ , W) can be deduced by the Fourier's law [10]:

$$\Phi = \lambda_{\mathrm{air}} A_{\mathrm{sample}} \frac{T_{\delta_{\mathrm{sample}}}}{\delta_{\mathrm{sample}}} + \lambda_{\mathrm{quartz}} A_{\mathrm{column}} \frac{T_{\delta_{\mathrm{column}}}}{\delta_{\mathrm{column}}} \qquad \text{(Equation S12)}$$

where λ_{air} and $\lambda_{\mathrm{quartz}}$ were the thermal conductivity of air and quartz tube respectively, W m^{-1} $^{o}C^{-1}$; A_{sample} was the contact area between tested sample and quartz tube, m^2; A_{column} was the contact area between the quartz tube column and air, m^2; T_{δ} was the temperature difference between the surface and distance δ, ^{o}C; δ was the distance between two temperature points in one thermal conductive medium, m. All the parameters used in heat duty calculation of ESA are listed in Supplementary Table 2.

1.5 Kinetic analysis

To analyze the kinetics of breakthrough adsorption curves, the Thomas model was applied to fit the experimental data, which accounts for both external mass transfer and intraparticle diffusion in fixed-bed column[11].

$$\ln(\frac{C_0}{C} - 1) = \frac{k_\mathrm{T} q_0 m_\mathrm{sample}}{Q} - k_\mathrm{T} C_0 t_\mathrm{r} \qquad \text{(Equation S13)}$$

Where k_T was the Thomas rate constant, L mmol^{-1} s^{-1}; q_0 was the sorption capacity per unit mass of adsorbent via Thomas model, mmol g^{-1}; t_r was the CO_2 retaining time, s.

1.6 Techno-economic analysis

The cost of DAC represents the average net cost of removing one ton of CO_2 for a given DAC technology including transport and storage, accounting for energy-related greenhouse gas (GHG) emissions and the capacity factor. This approach ensures a comprehensive assessment of the true cost of CO_2 removed, considering only energy-related emissions as they dominate in life cycle analysis of DAC.

DAC cost in \$ t^{-1} CO_2 (in US\$2022 terms) is the sum of levelized capital expenditure (CAPEX), annual fixed operational expenditure (fixed OPEX), annual variable OPEX and and the cost of CO_2 transport & storage where:

$$CAPEX = \frac{C_{toc} * CRF}{plant\ size * cf * (1 - X)} \qquad \text{(Equation S14)}$$

CAPEX, in \$ t^{-1} CO_2 removed, is calculated by determining the total overnight cost (C_{toc})60 (Equation S15) which is subsequently annualized and levelized taking into account the capital recovery factor (CRF) (Equation S16), the plant size (plant size) and energy related emissions (x), and the capacity factor (cf).

Total overnight cost C_{toc} are calculated from direct materials cost of components (C_d), installation cost (C_i), and engineering and procurement cost (C_{ep}), process and project contingencies (C_c), owner's spare parts (C_o), and startup cost (C_s) (see Supplementary Table 4).

$$C_{toc} = C_d + C_i + C_{ep} + C_c + C_o + C_s \qquad \text{(Equation S15)}$$

CRF is derived from a 7% discount rate , and a 25-year plant life (T).

$$CRF = \frac{r(1+r)^T}{(1+r)^T - 1} \qquad \text{(Equation S16)}$$

Energy related GHG emissions (Equation S8, represented as x) are calculated based on the carbon intensity of the energy sources and their respective energy consumption, and subsequently deducted from the plant size (refer to for details on the carbon intensities of different energy sources).

Fixed OPEX are calculated in \$ t^{-1} CO_2 removed by considering direct and indirect labor, maintenance, insurance, local taxes and fees, and adjusting for plant capacity, and employee salaries (Supplementary Table 6). Variable OPEX are calculated in \$ t^{-1} CO_2 by accounting for electricity, natural gas, low-grade heat, water, chemicals and sorbent replacement (Tables S7).

2. Supplementary Figures

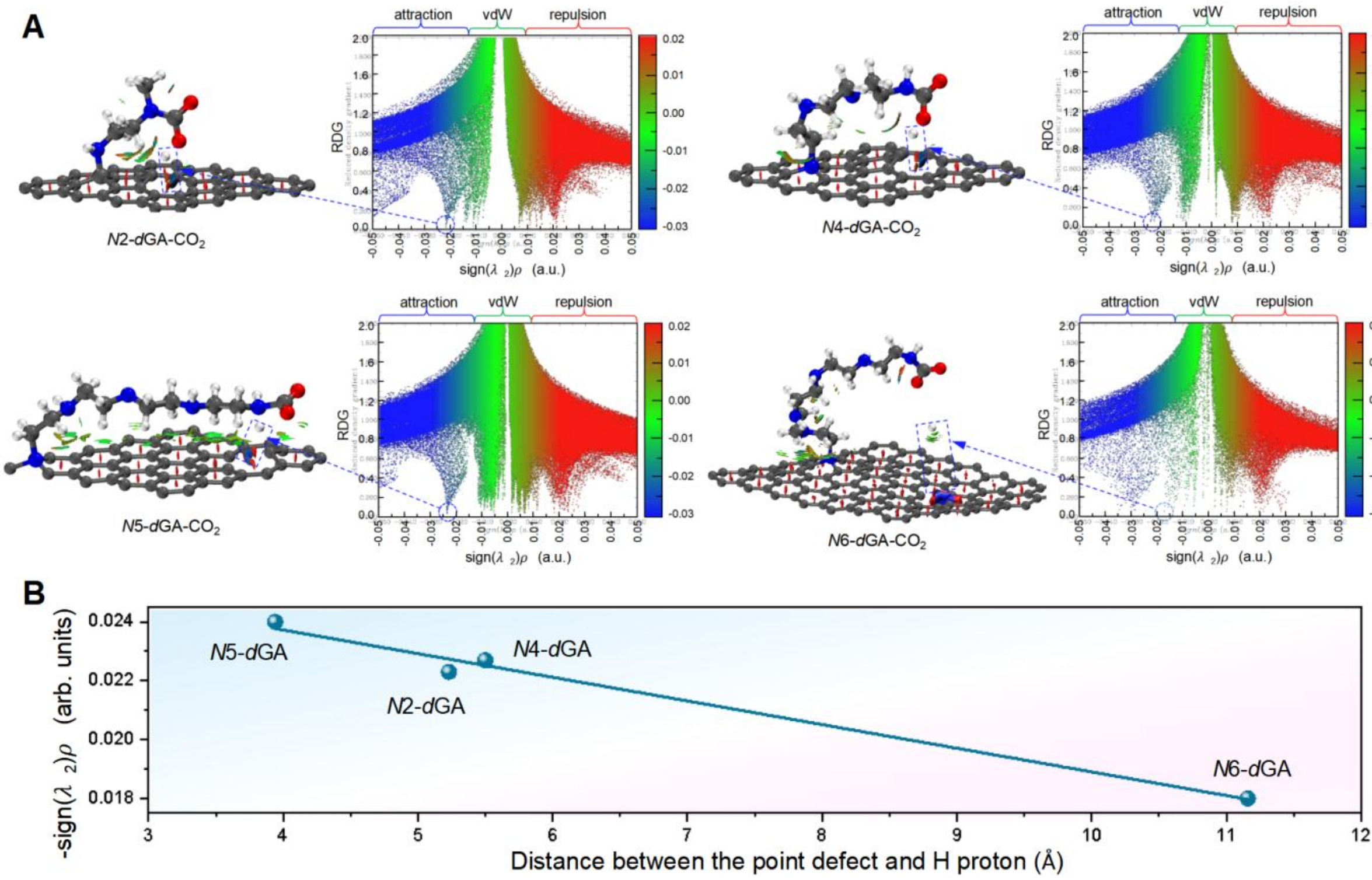

Supplementary Figure 1: (A) 3D image and scatter plot of RDG in *N-d*GA-CO_2. (B) Relationship between the RDG value of proton transfer and the distance between the point defect of *N-d*GA and its H proton. Source data for Supplementary Figure 1 are provided as a Source Data file.

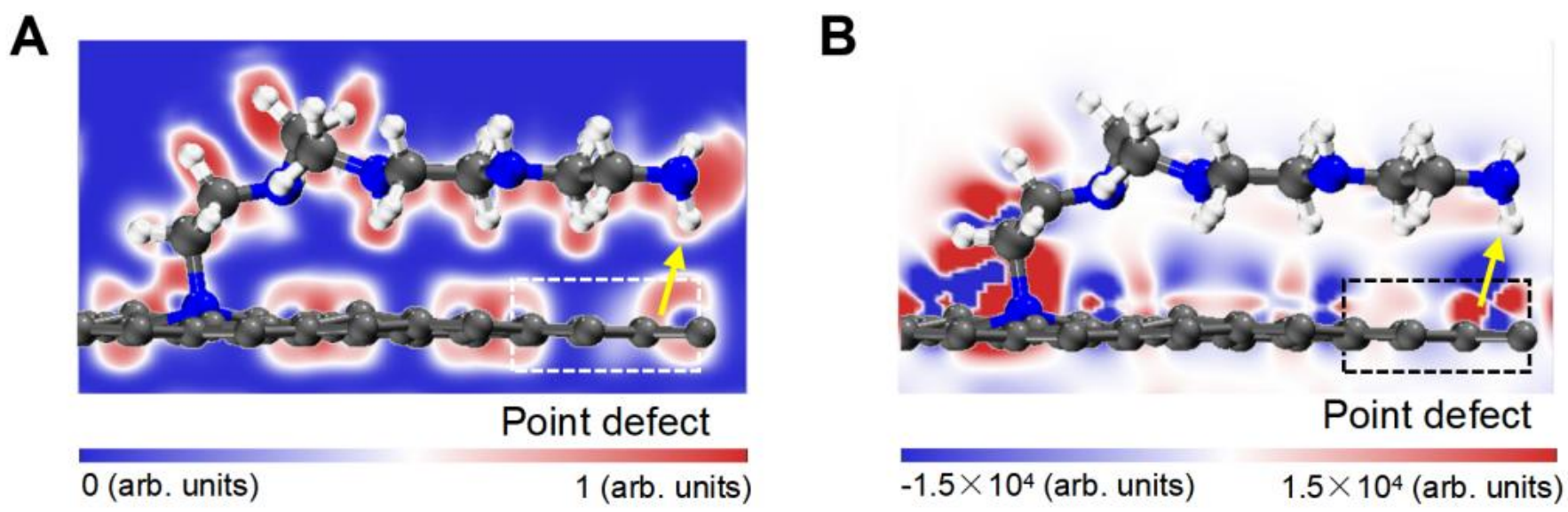

Supplementary Figure 2: (A) Electronic localization function plot and **(B)** isosurfaces of two-dimensional charge difference density distribution for *N*5-*d*GA. The yellow arrow represents the tendency of electron transfer from the carbon atom hanging on the point defect to the hydrogen proton of the amine group.

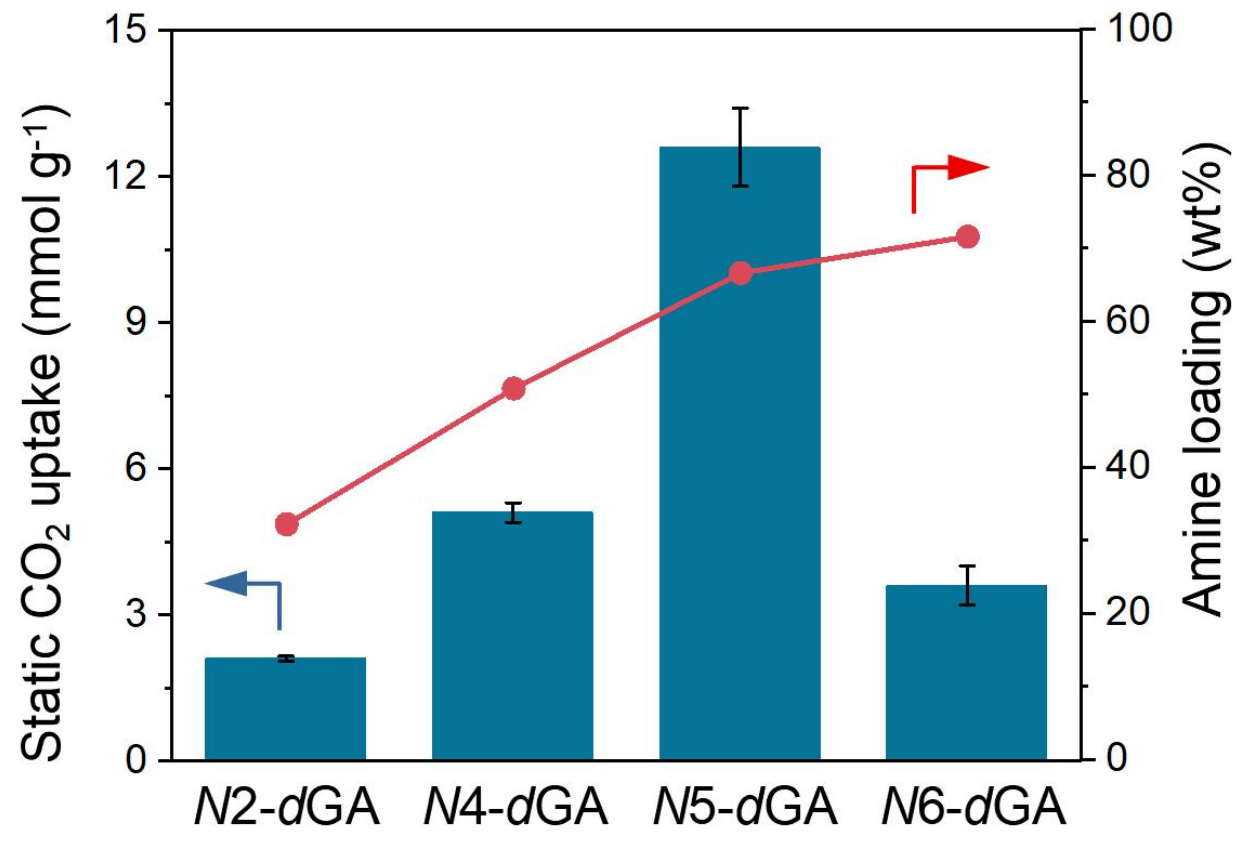

Supplementary Figure 3: CO_2 uptakes and amine loadings of *N*-*d*GA under a pure CO_2 atmosphere (25 °C, 1.0 bar). The error bars correspond to the standard deviation of three independent measurements. Data are presented as mean values ± s.d. Source data for Figure S3 are provided as a Source Data file.

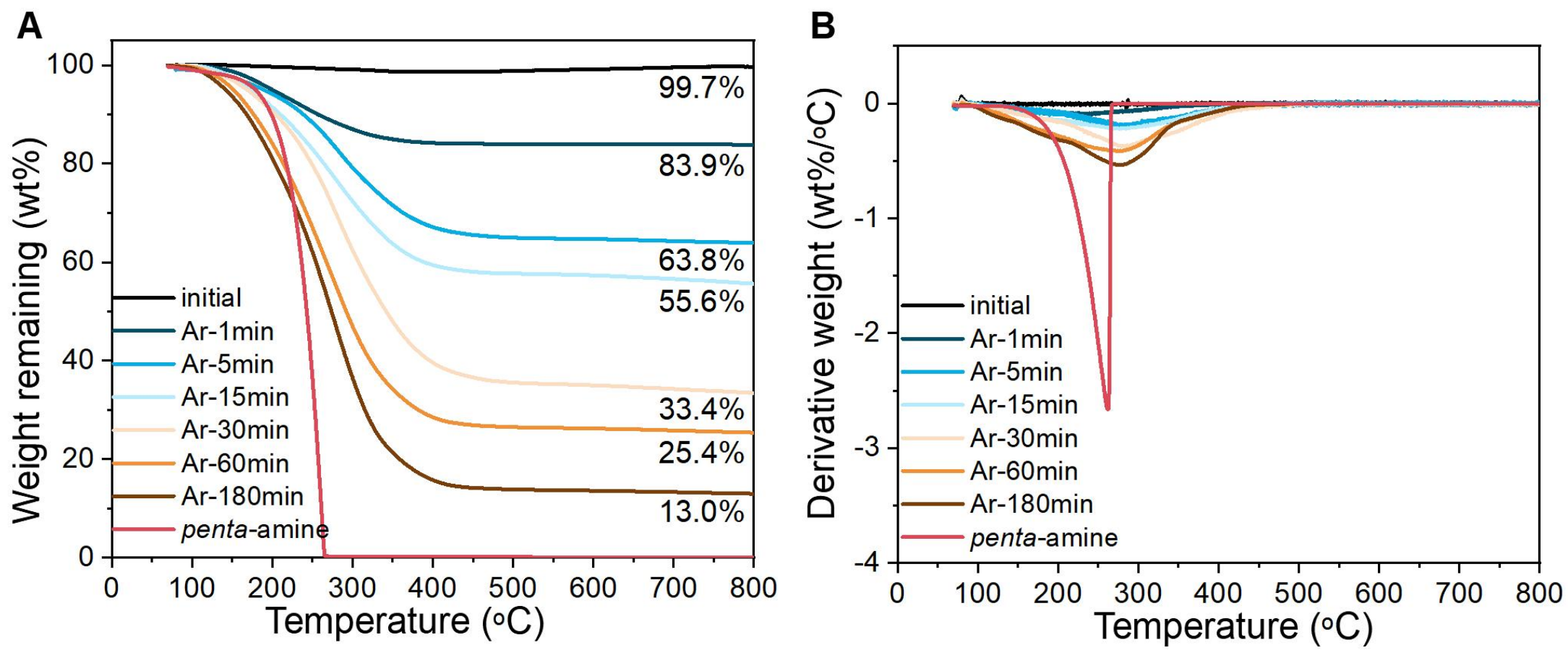

Supplementary Figure 4: (A) TG curves and (B) derivative of thermal gravity curves of *N*5-*d*GA after different durations of Ar plasma treatment. The heating rate was set to 10°C min^{-1}. Source data for Supplementary Figure 4 are provided as a Source Data file.

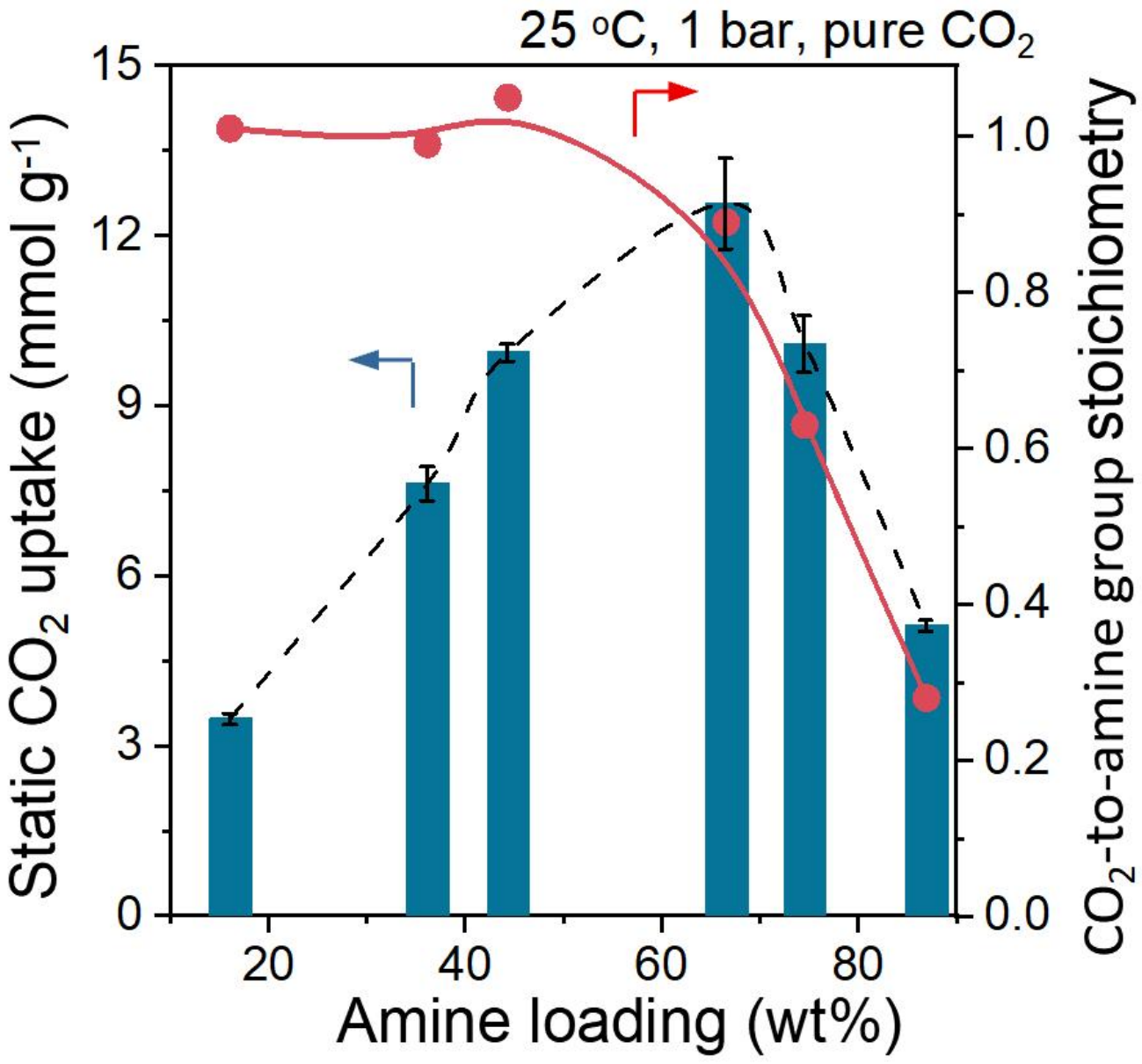

Supplementary Figure 5: CO_2 uptakes and CO_2-to-amine group stoichiometry of *N*5-*d*GA with different amine loadings. The error bars correspond to the standard deviation of three independent measurements. Data are presented as mean values ± s.d. Source data for Supplementary Figure 5 are provided as a Source Data file.

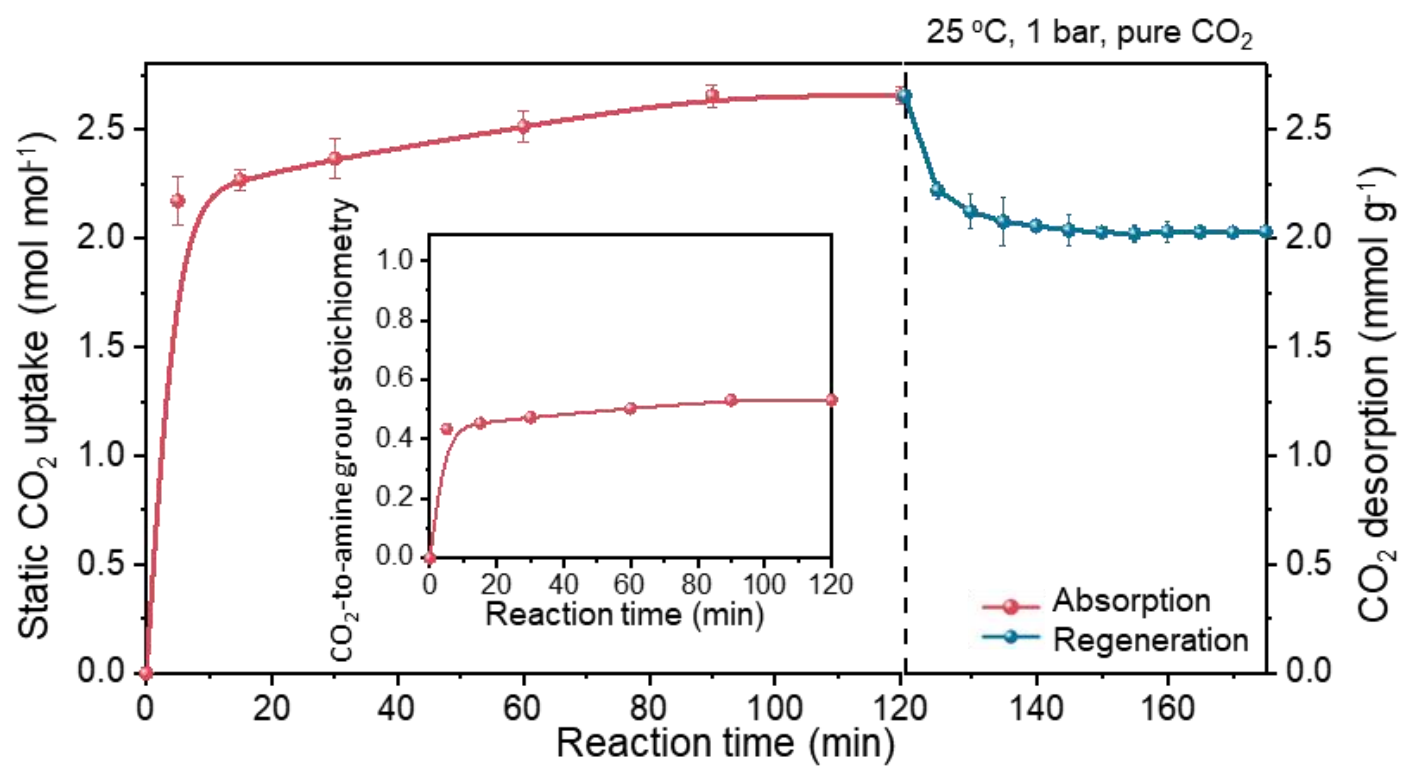

Supplementary Figure 6: CO_2 absorption and regeneration curves of 30 mL aqueous *penta*-amine solution at the same molar concentration with *N*5-*d*GA. The inset shows the CO_2-to-amine group stoichiometry of pentaamine in the solution during the CO_2 absorption. The regeneration process was 20 mL min^{-1} pure CO_2 gas flow, 25 °C, 1.0 bar. The regeneration process was conducted at 80 °C, 1.0 bar. The error bars correspond to the standard deviation of three independent measurements. Data are presented as mean values ± s.d. Source data for Supplementary Figure 6 are provided as a Source Data file.

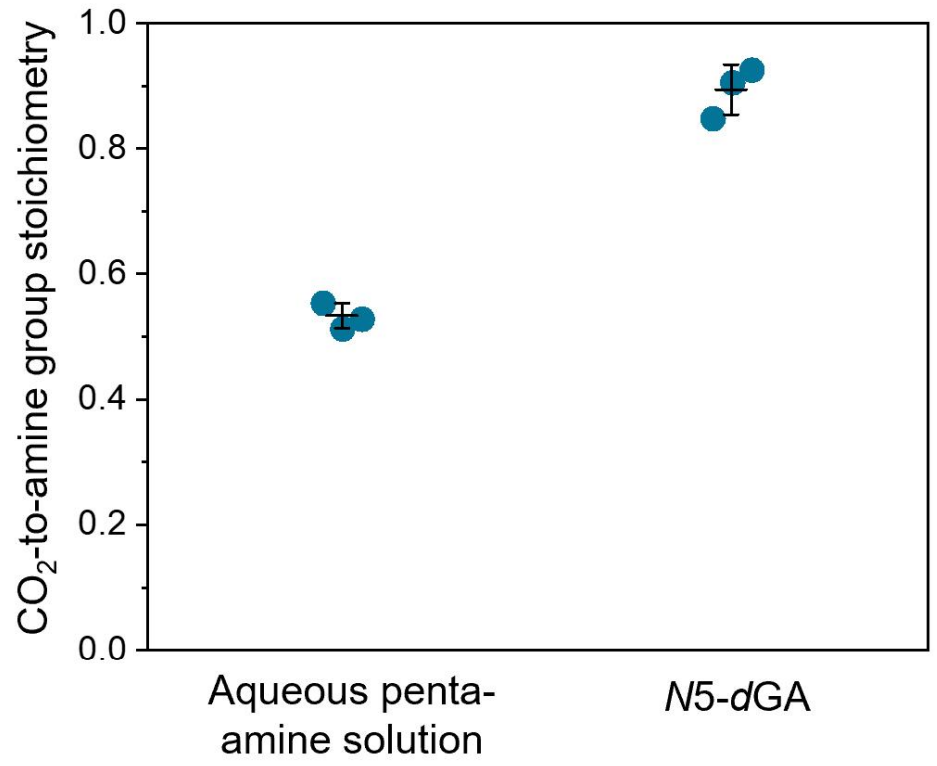

Supplementary Figure 7: Comparison of the CO_2-to-amine group stoichiometry for the aqueous pentaamine solution and *N*5-*d*GA. The error bars correspond to the standard deviation of three independent measurements. Data are presented as mean values ± s.d. Source data for Supplementary Figure 7 are provided as a Source Data file.

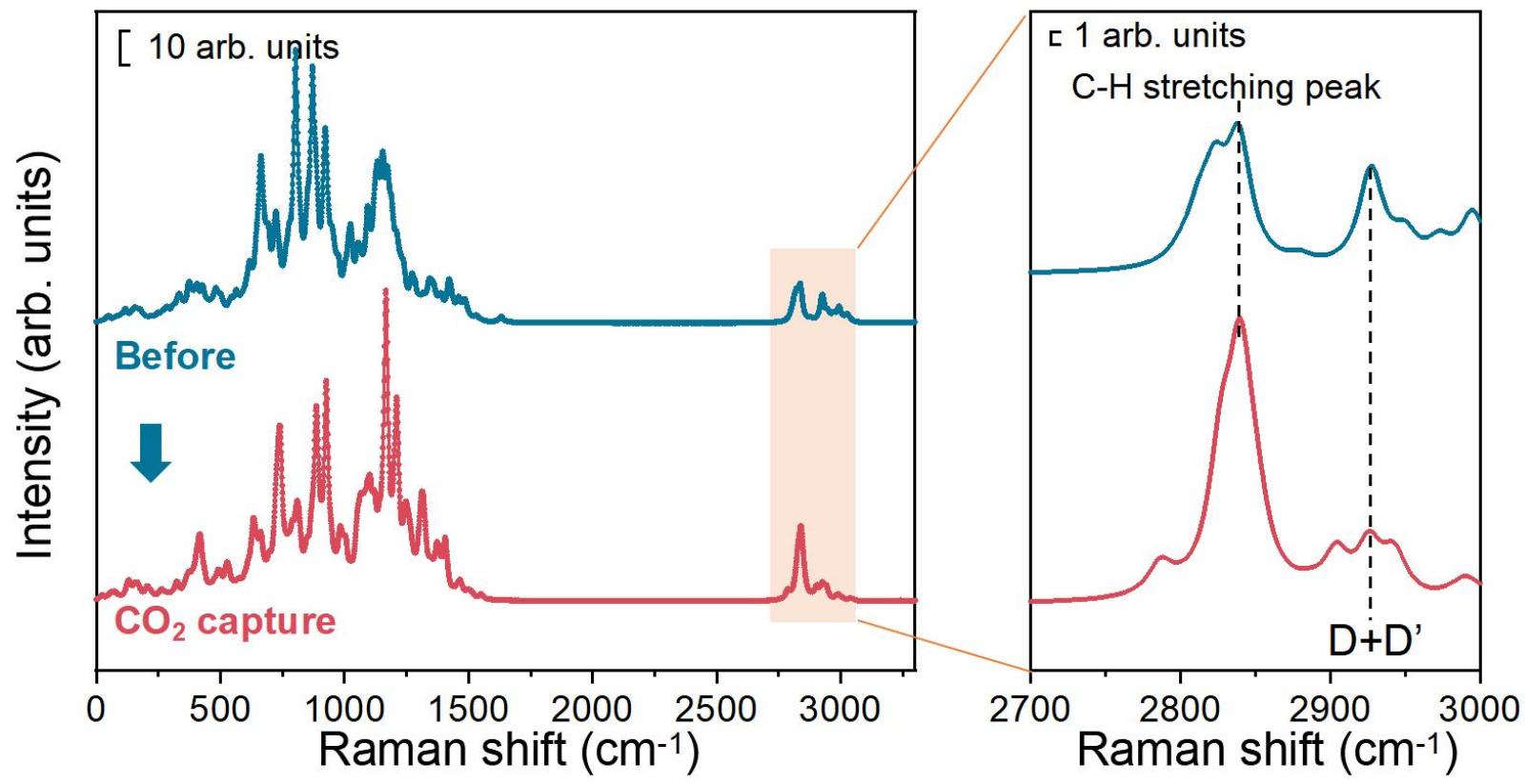

Supplementary Figure 8: DFT-simulated Raman spectra of *N*5-*d*GA before and after CO_2 capture. Source data for Supplementary Figure 8 are provided as a Source Data file.

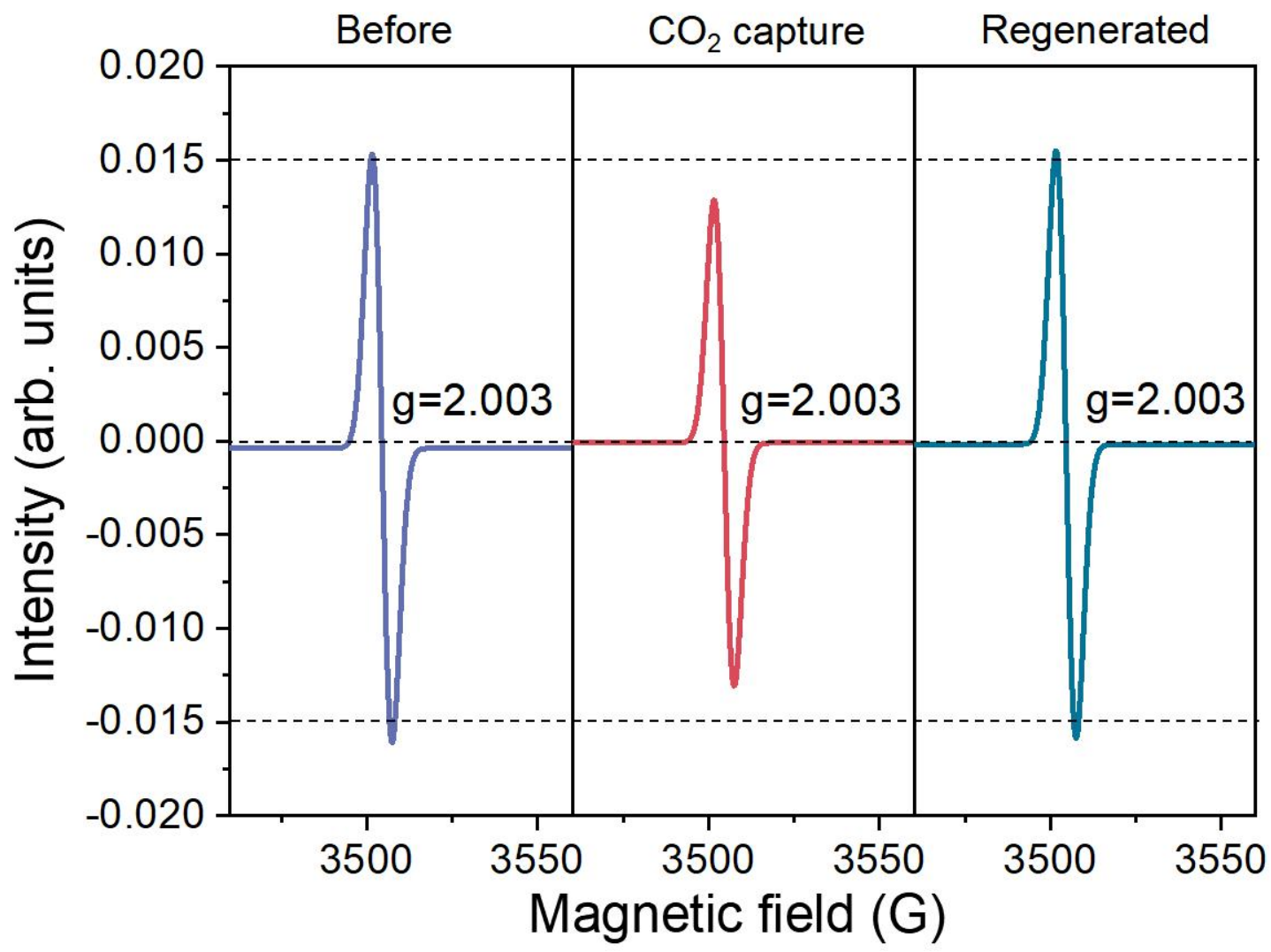

Supplementary Figure 9: EPR spectra of *N*5-*d*GA before and after CO_2 capture, and after Joule heating regeneration. Source data for Supplementary Figure 9 are provided as a Source Data file.

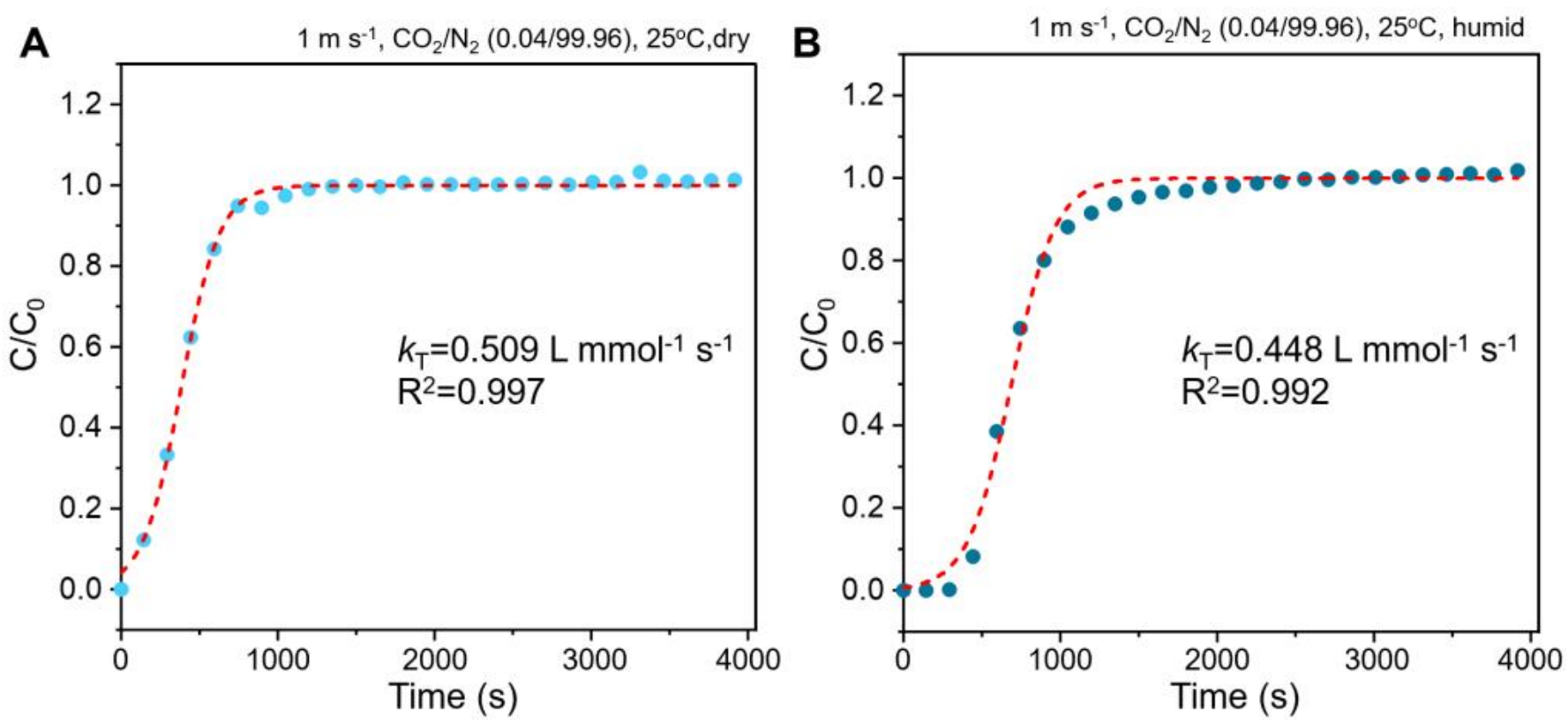

Supplementary Figure 10: CO_2 adsorption rate constant of *N*5-*d*GA under (A) dry and (B) humid conditions. Dashed lines represent the breakthrough curves fitted using the Thomas model. Source data for Supplementary Figure 10 are provided as a Source Data file.

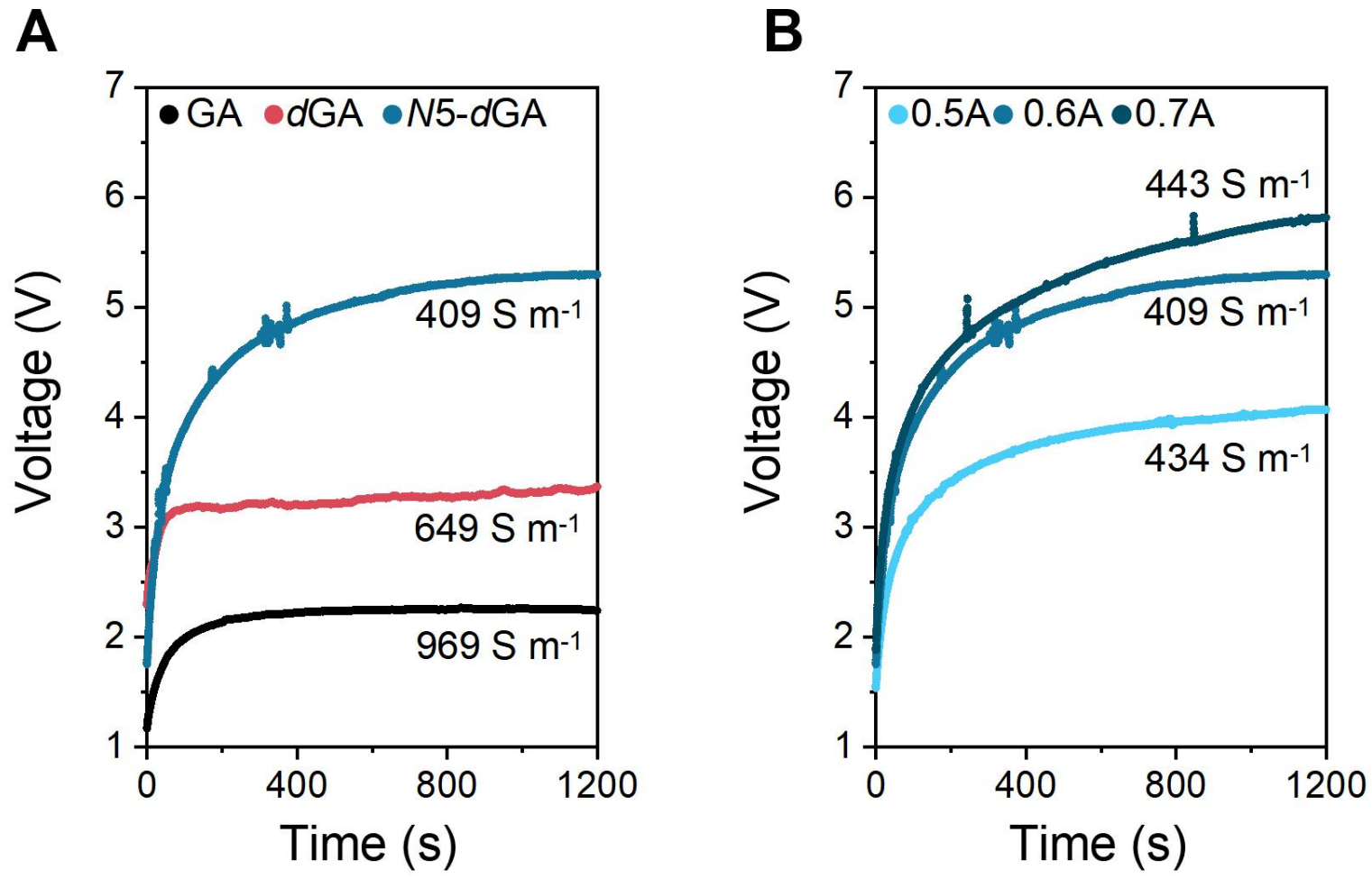

Supplementary Figure 11: (A) The electrical conductivity of GA, *d*GA and *N*5-*d*GA packed into a quartz breakthrough column. I=0.6 A. (B) The electrical conductivity of *N*5-*d*GA packed into a quartz breakthrough column at different current intensities. Source data for Supplementary Figure 11 are provided as a Source Data file.

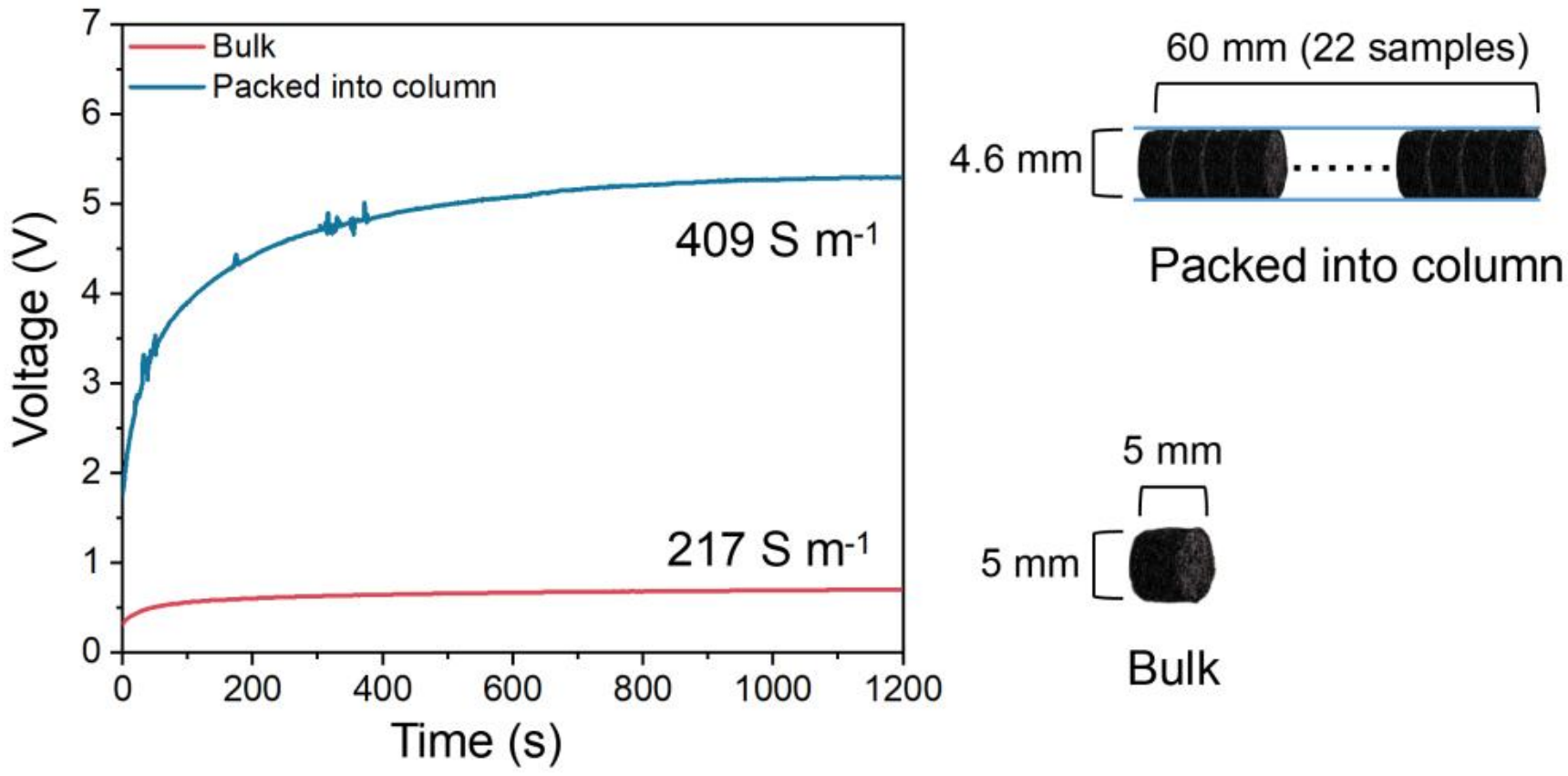

Supplementary Figure 12. The electrical conductivity of single bulk *N*5-*d*GA and *N*5-*d*GA packed into a quartz breakthrough column. I=0.6 A. Source data for Supplementary Figure 12 are provided as a Source Data file.

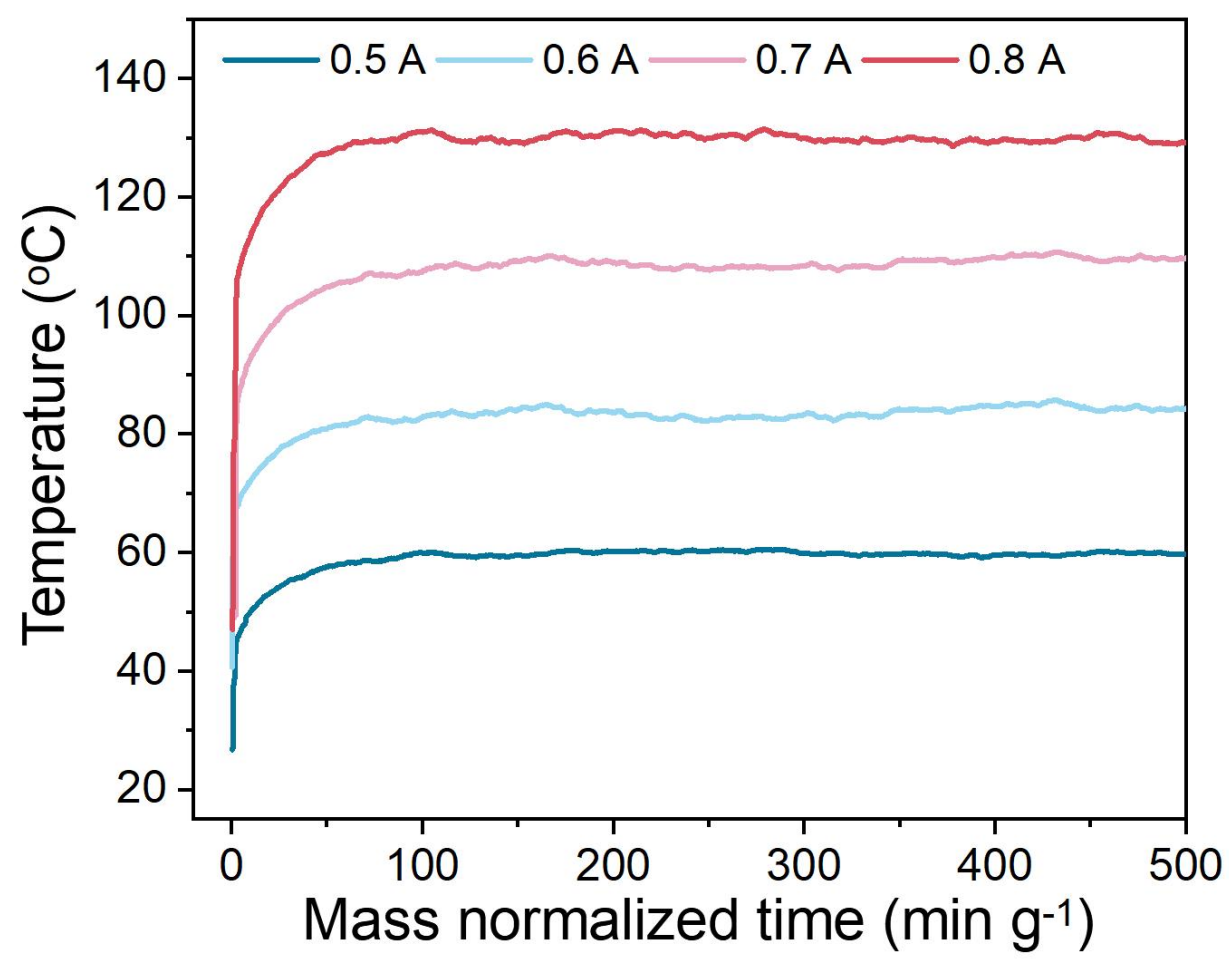

Supplementary Figure 13: Time-dependence temperature curves of *N*5-*d*GA packed into a quartz breakthrough column at different current intensities. Source data for Supplementary Figure 13 are provided as a Source Data file.

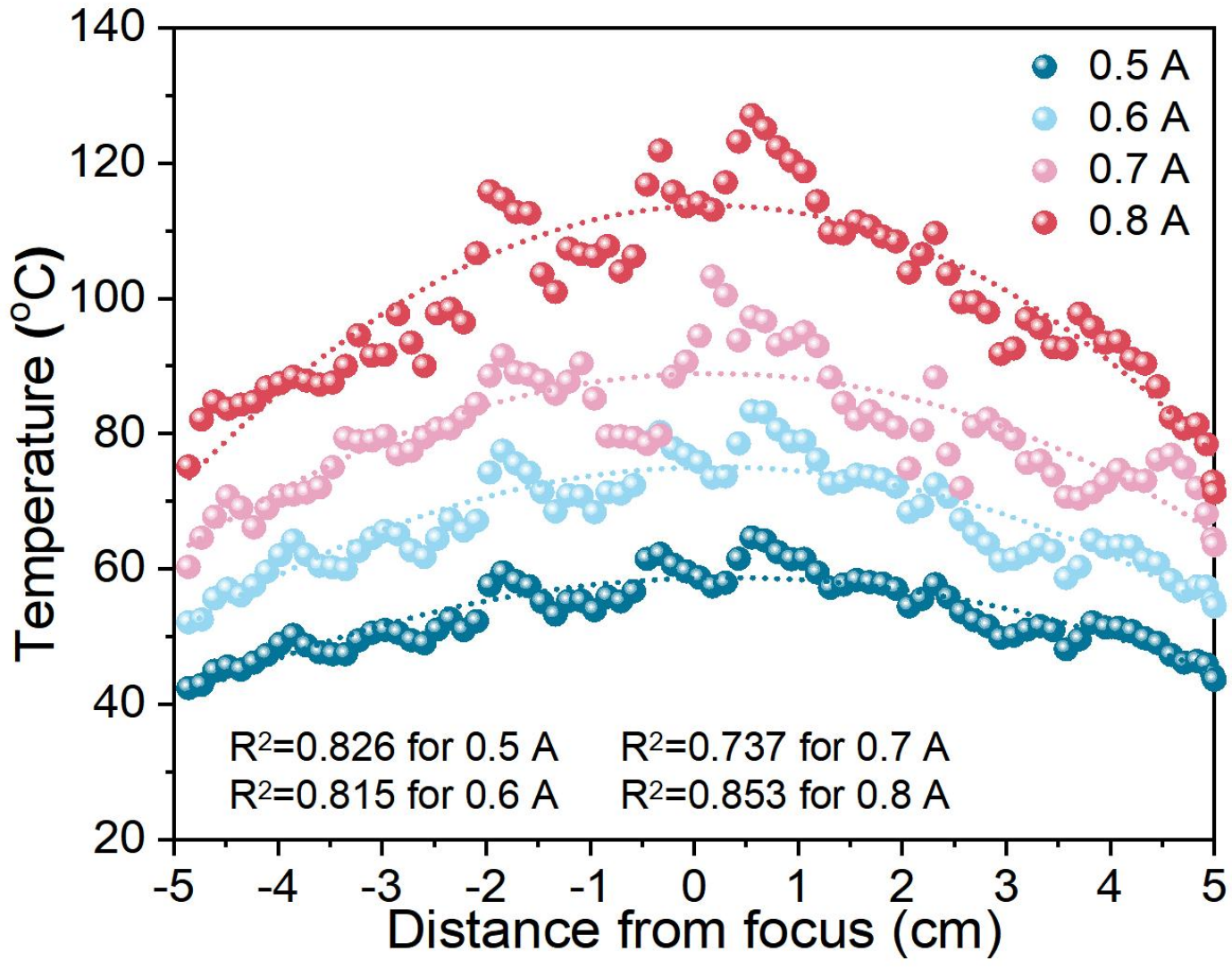

Supplementary Figure 14: Distance-dependence temperature of focus point on bulk *N*5-*d*GA surface at different current intensities. The temperature distributions were fitted using a second-order polynomial function, and the corresponding R^2 values are provided. Source data for Supplementary Figure 14 are provided as a Source Data file.

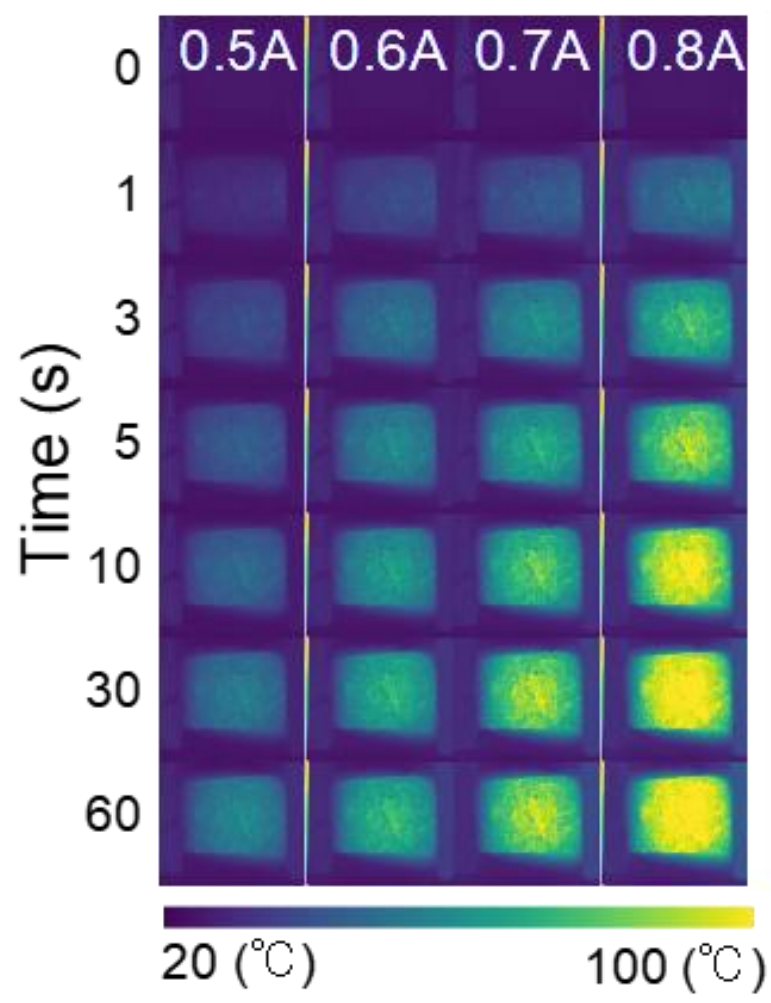

Supplementary Figure 15: The thermal images of bulk *N*5-*d*GA at different current intensities.

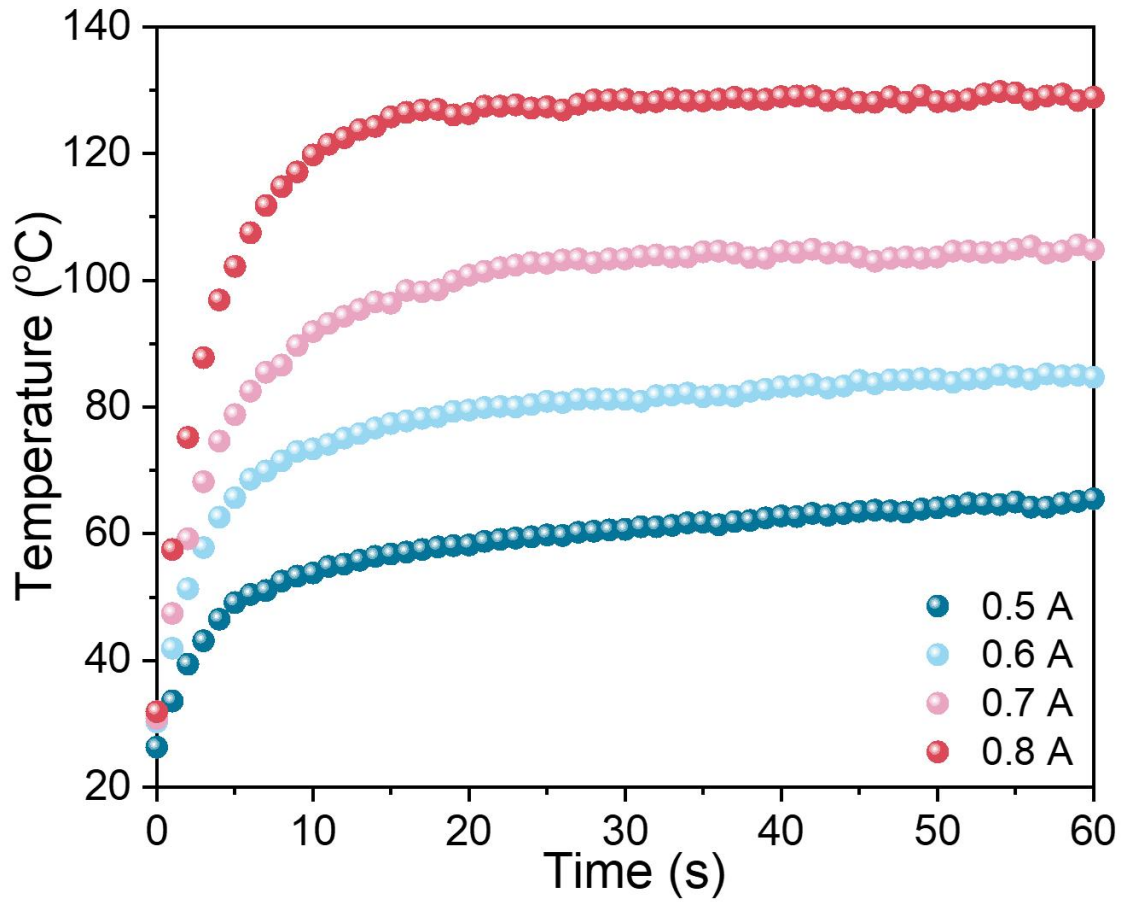

Supplementary Figure 16: Time-dependence temperature curves of the bulk *N*5-*d*GA at different current intensities. Source data for Supplementary Figure 16 are provided as a Source Data file.

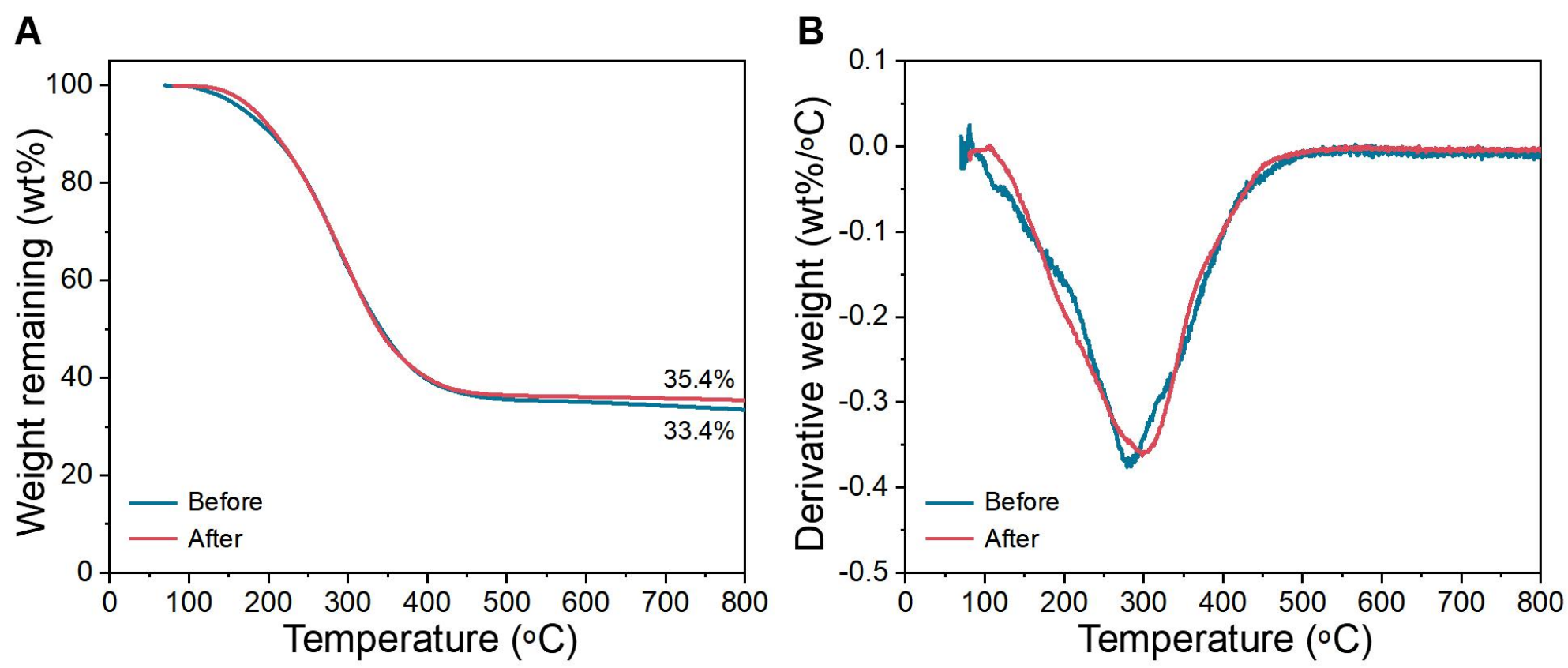

Supplementary Figure 17: (A) TG curves and (B) derivative of thermal gravity curves of *N5-d*GA before and after 100 adsorption-desorption cycles. Source data for Supplementary Figure 17 are provided as a Source Data file.

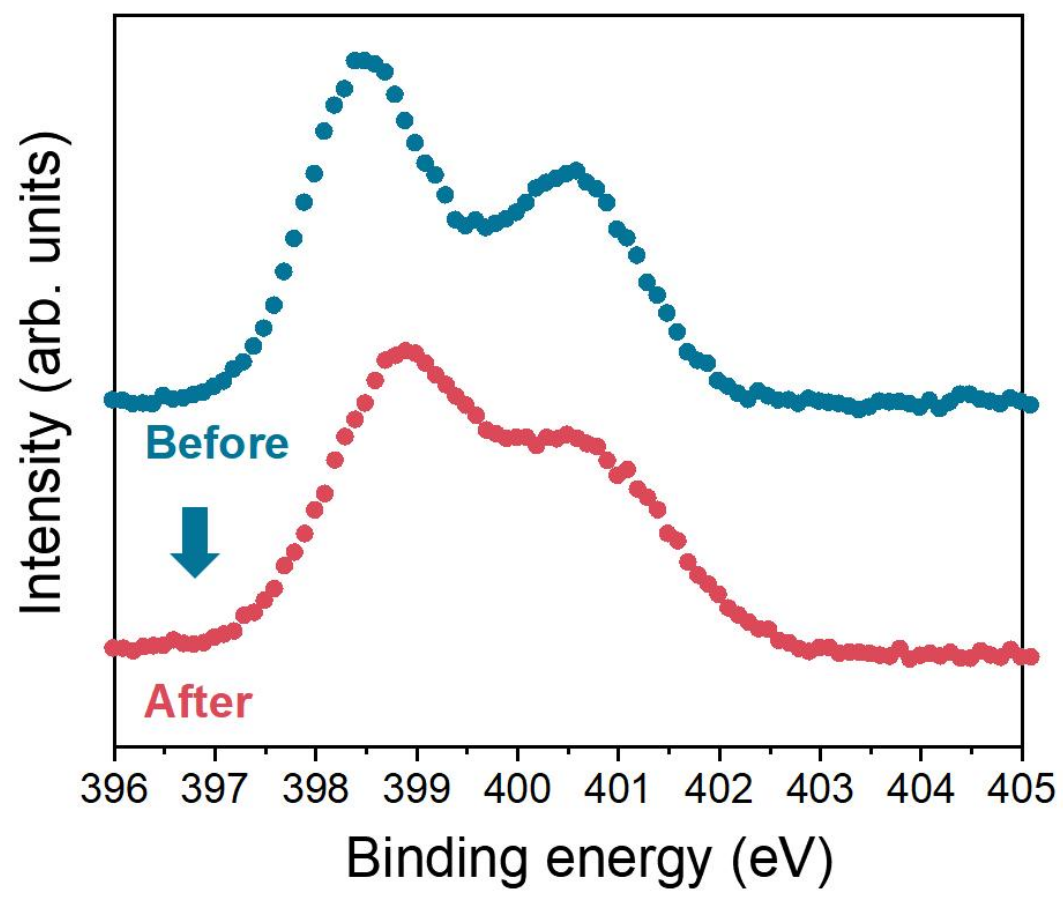

Supplementary Figure 18: High-resolution XPS N *1s* spectra of *N*5-*d*GA before and after 100 adsorption-desorption cycles. Source data for Supplementary Figure 18 are provided as a Source Data file.

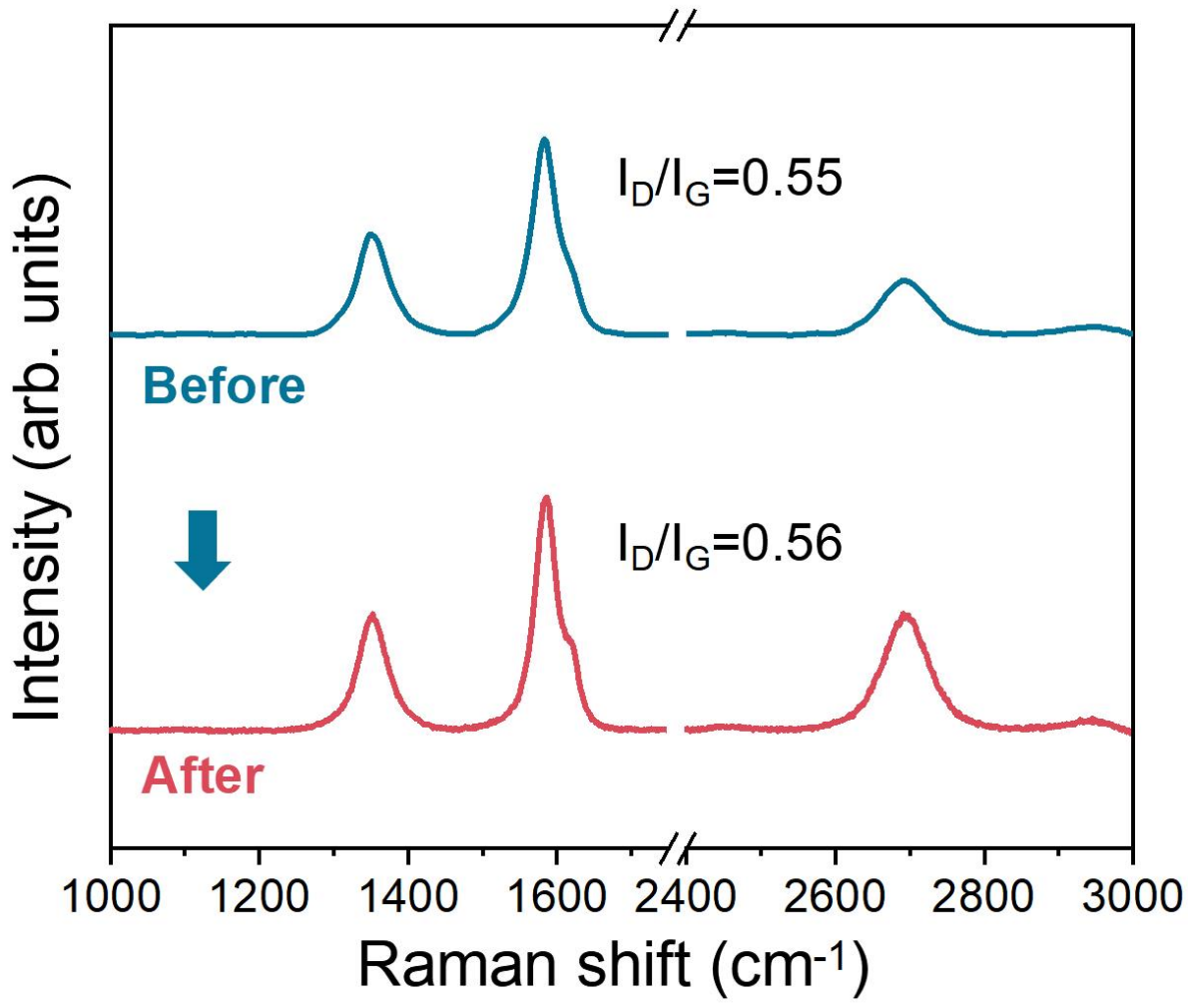

Supplementary Figure 19: Raman spectroscopy of *N*5-*d*GA before and after 100 adsorption- desorption cycles. Source data for Supplementary Figure 19 are provided as a Source Data file.

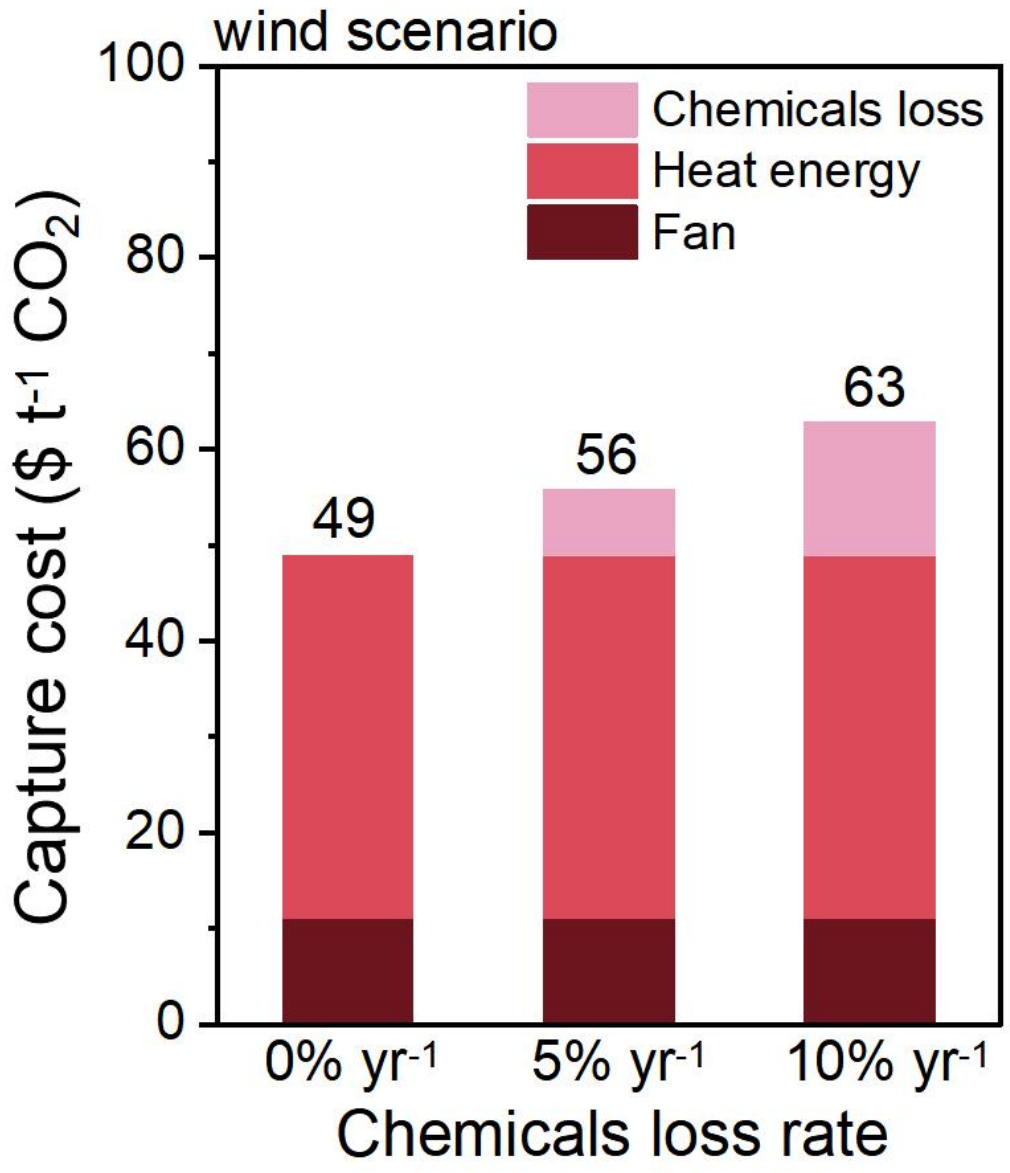

Supplementary Figure 20: Detailed capture cost for ESA-DAC systems by wind of a 4000 t a^{-1} scale. The amount of CO_2 desorbed is 95%. Source data for Supplementary Figure 20 are provided as a Source Data file.

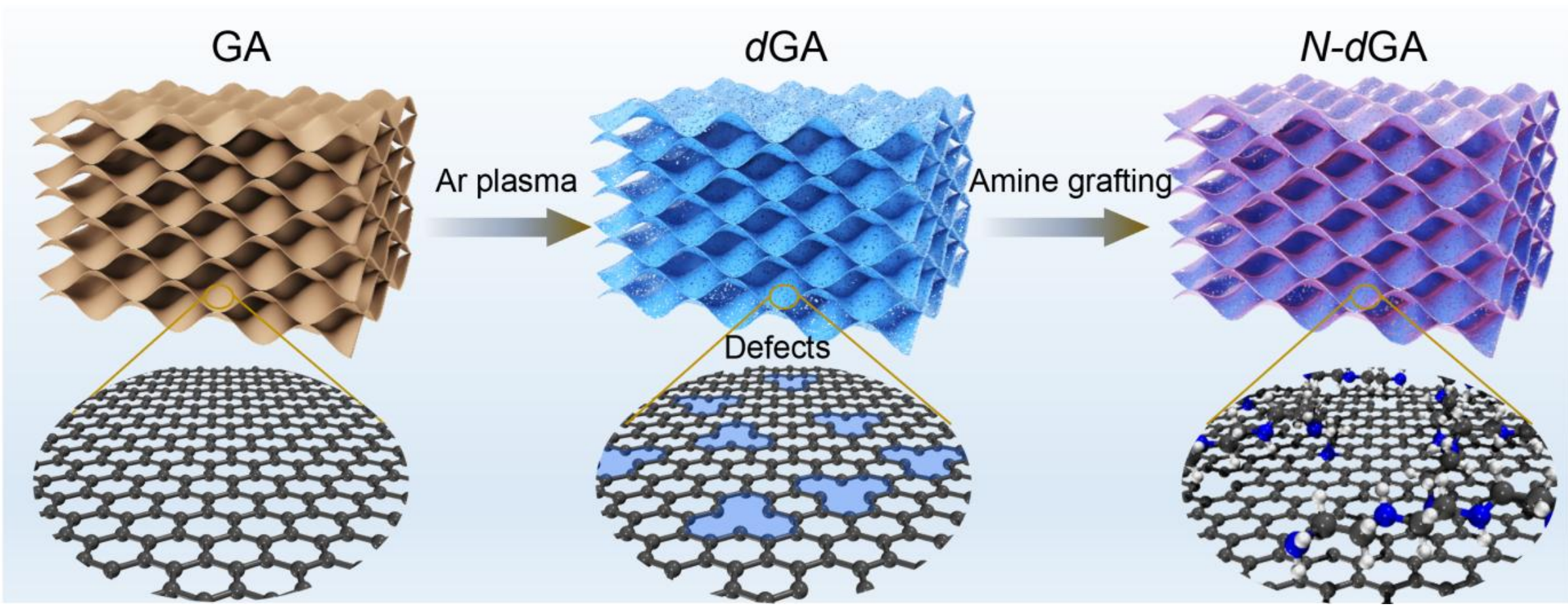

Supplementary Figure 21: Schematic illustration of *N*-*d*GA.

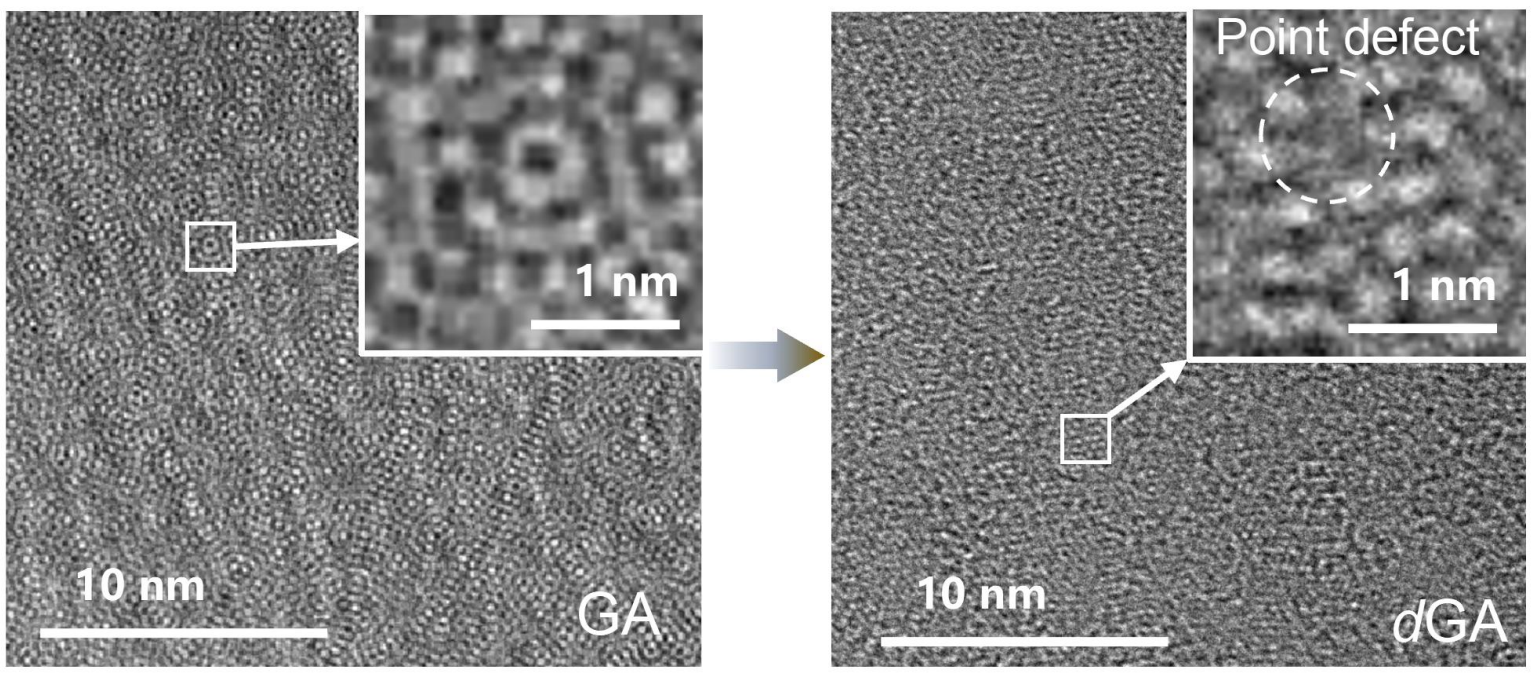

Supplementary Figure 22: AC-TEM images of GA and *d*GA after 30 min of Ar plasma treatment. Inset images show enlarged atomic-scale views of the selected regions, indicating the generation of point defects on the graphene after Ar plasma treatment.

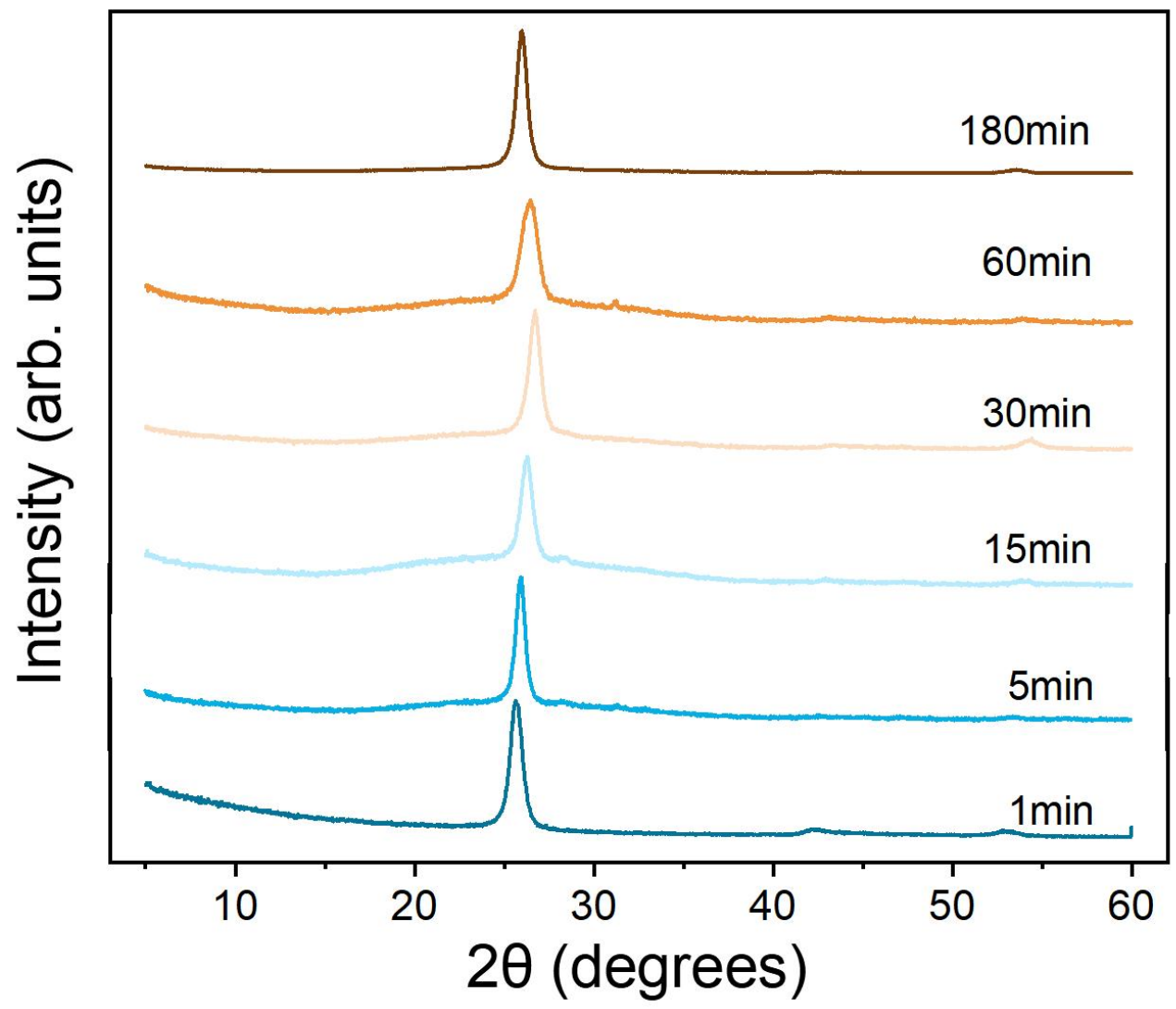

Supplementary Figure 23: XRD patterns of *d*GA after different durations of Ar plasma treatment. Source data for Supplementary Figure 23 are provided as a Source Data file.

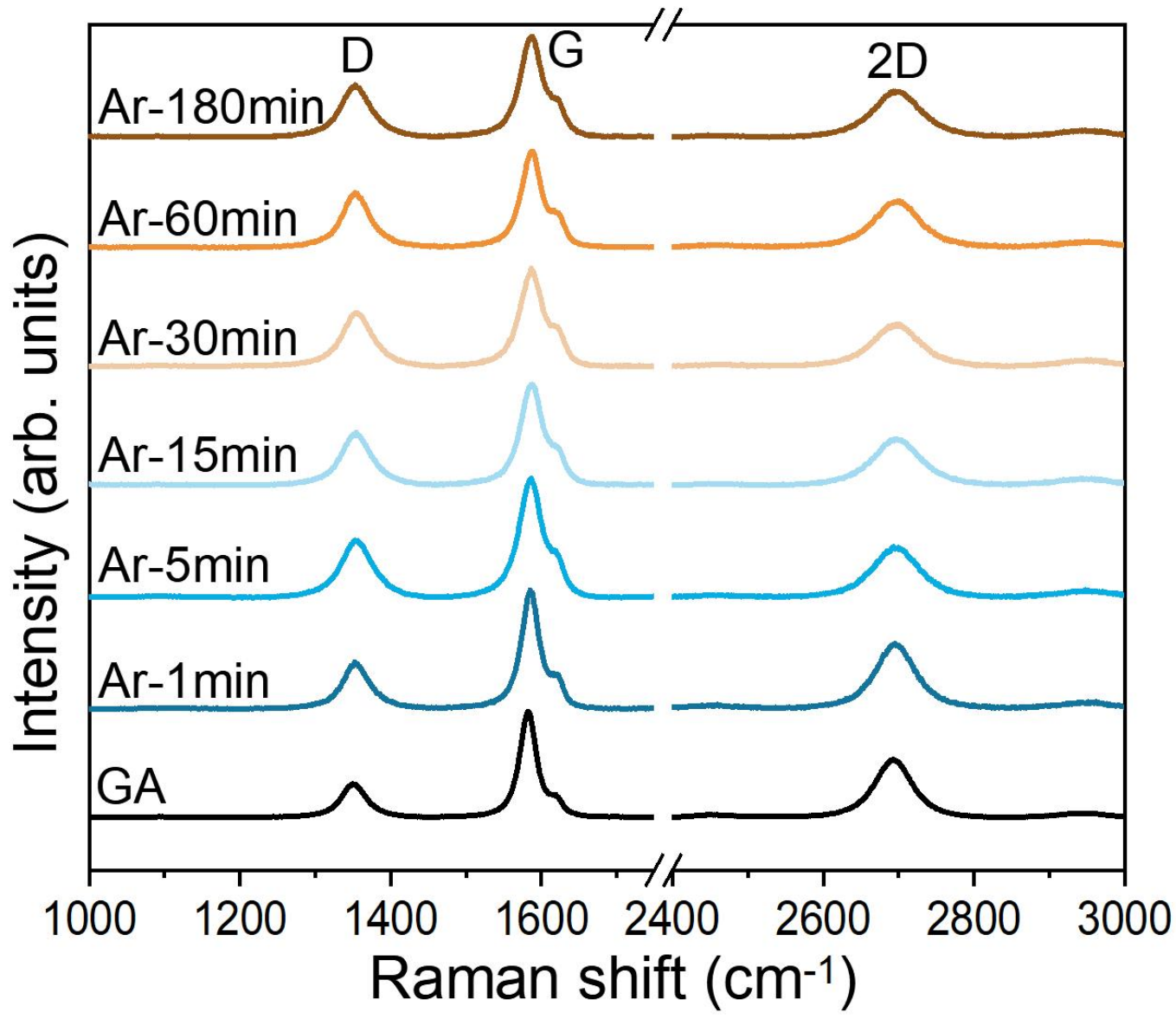

Supplementary Figure 24: Raman spectroscopy of *d*GA after different durations of Ar plasma treatment. With the processing time increasing to 180 minutes, all Raman characteristic peaks (D, G and 2D) of *d*GA slightly widened, indicating a decrease in overall crystallinity. Source data for Supplementary Figure 24 are provided as a Source Data file.

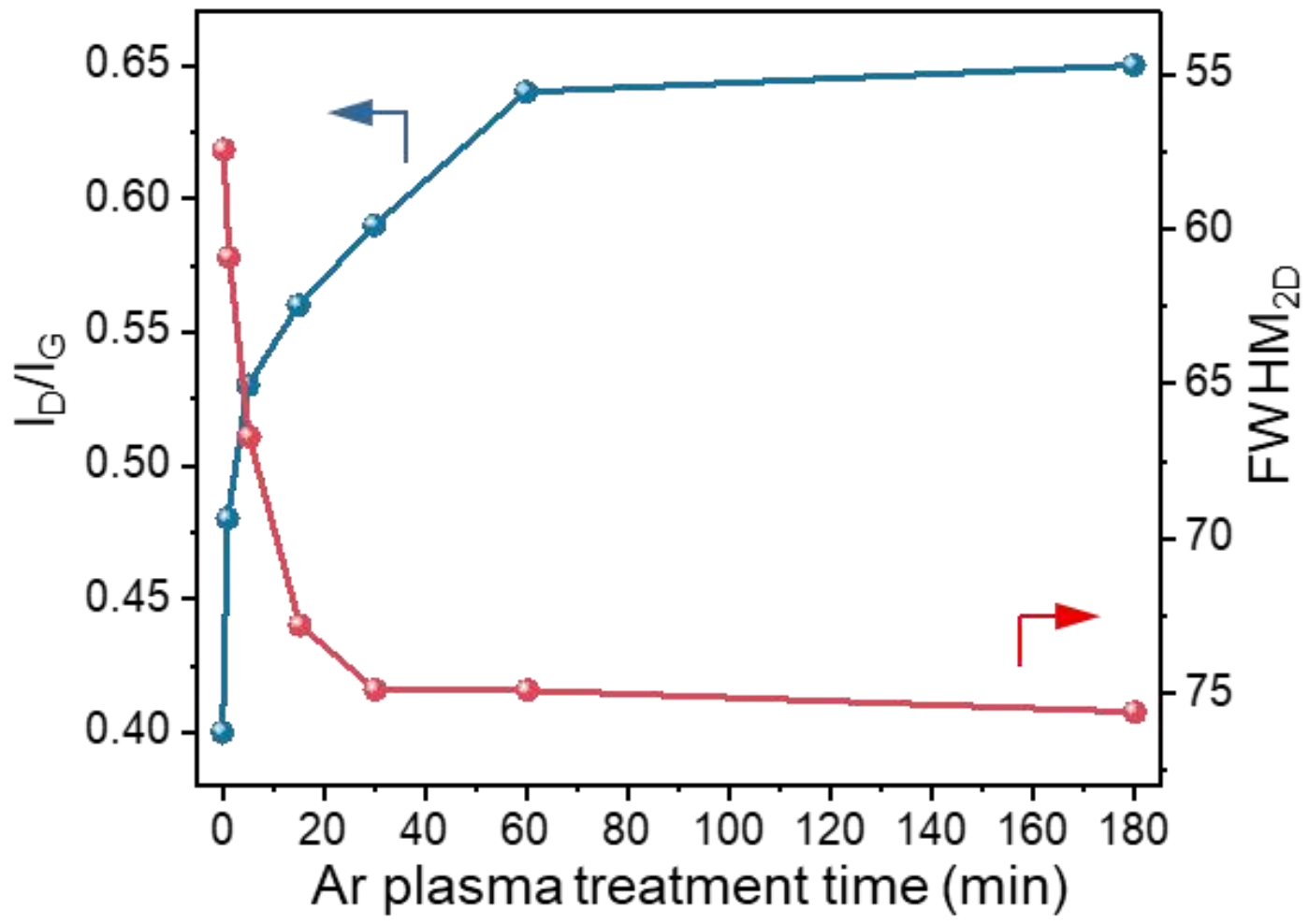

Supplementary Figure 25: I_D/I_G and $FWHM_{2D}$ of *d*GA after different durations of Ar plasma treatment. Source data for Supplementary Figure 25 are provided as a Source Data file.

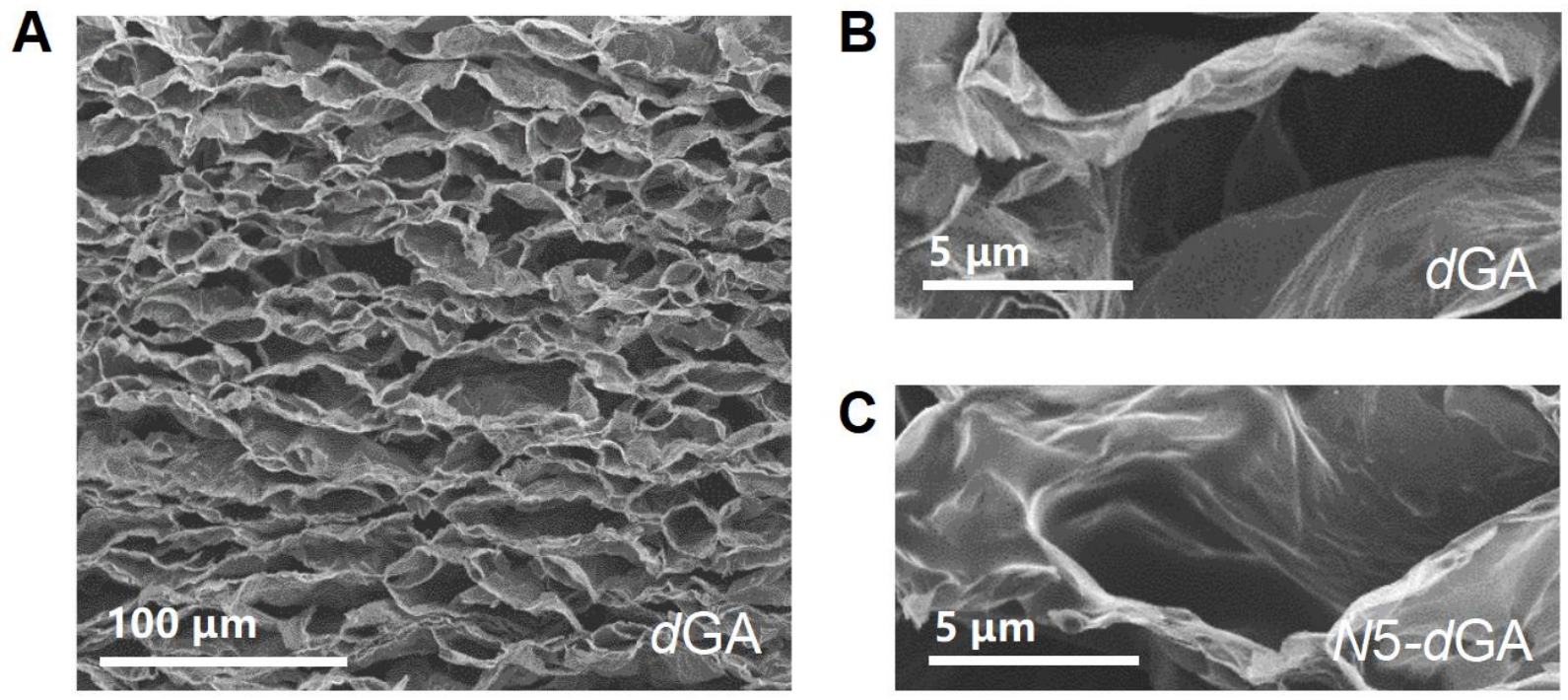

Supplementary Figure 26: (A,B) FE-SEM images of *d*GA at different magnifications, showing showing the high porosity and hyperboloid structure. (C) FE-SEM image of *N*5-*d*GA, showing the retained porosity and hyperboloid structure after penta-amine grafting.

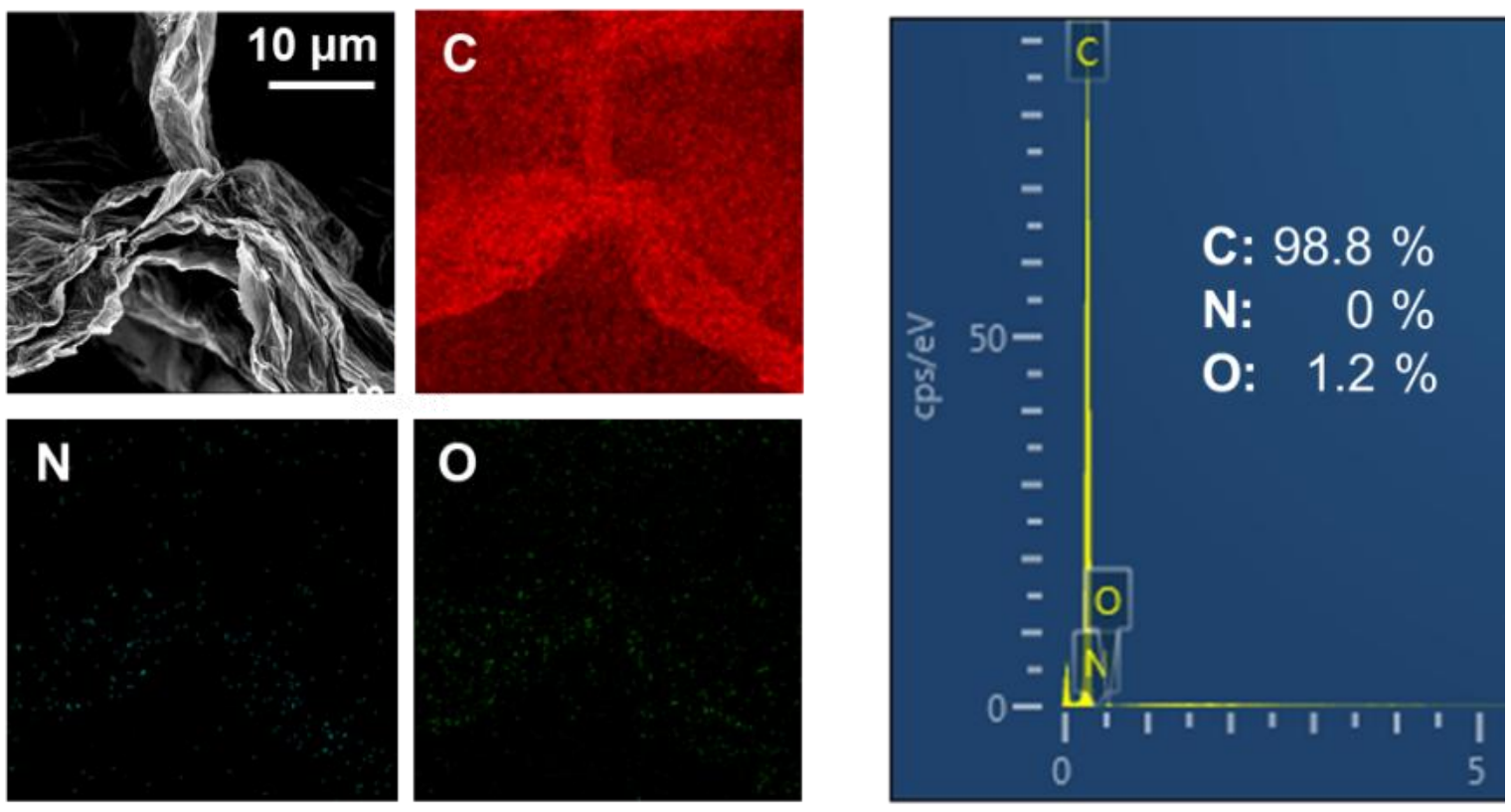

Supplementary Figure 27: The EDS images and result of *d*GA.

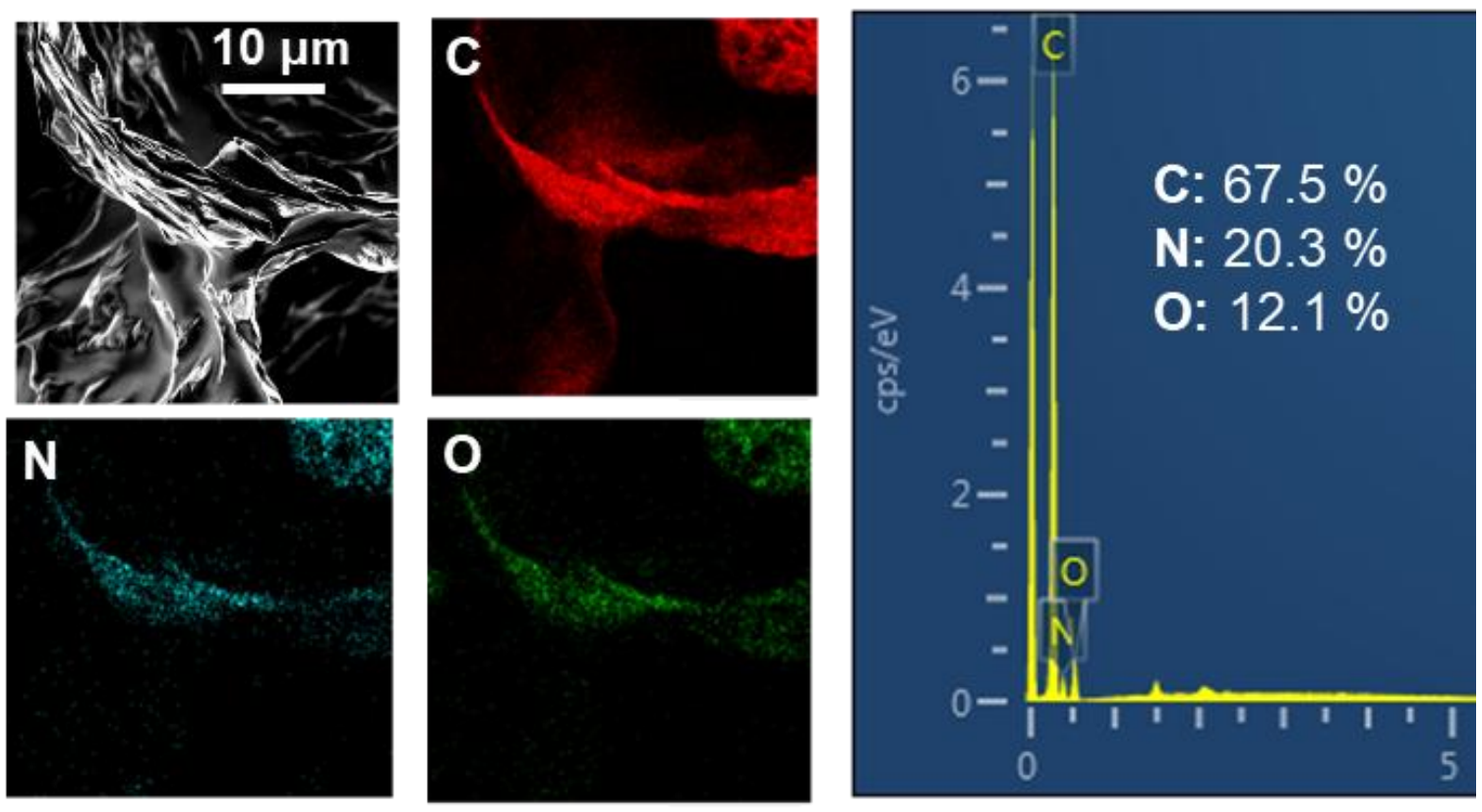

Supplementary Figure 28: The EDS images and result of *N*5-*d*GA.

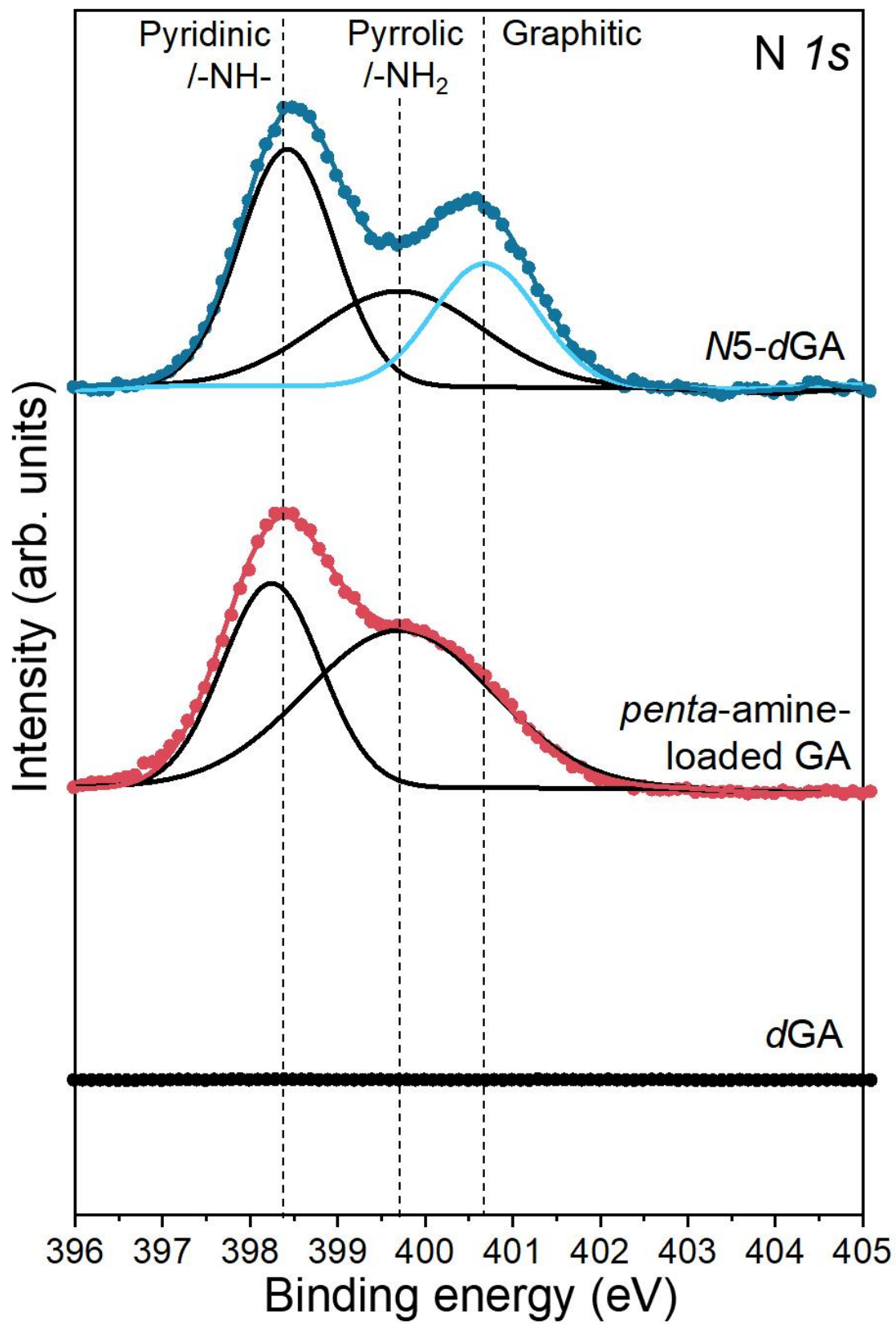

Supplementary Figure 29: High-resolution XPS N *1s* spectra of *N*5-*d*GA, *penta*-amine-loaded GA and *d*GA. Source data for Supplementary Figure 29 are provided as a Source Data file.

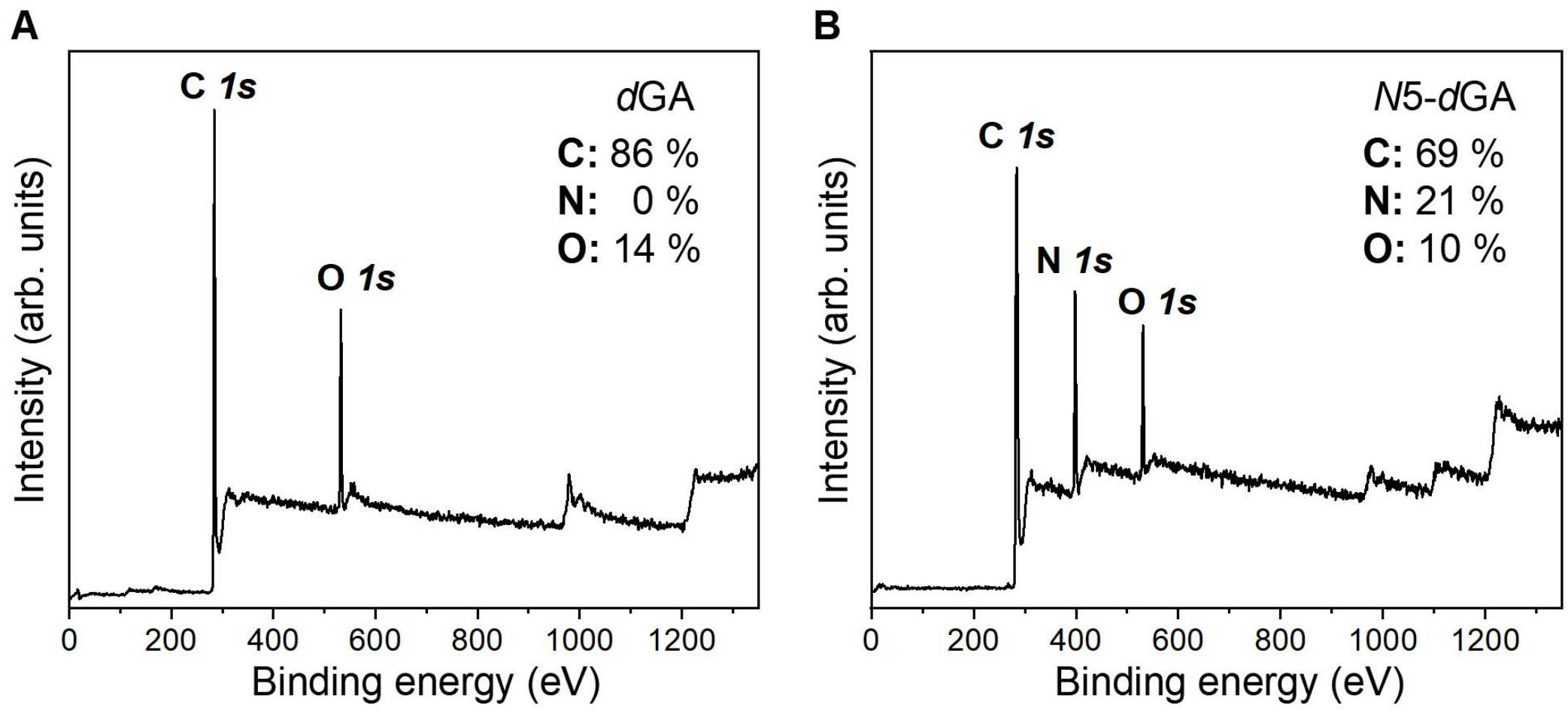

Supplementary Figure 30: The XPS survey of (A) *d*GA and (B) *N*5-*d*GA. Source data for Supplementary Figure 30 are provided as a Source Data file.

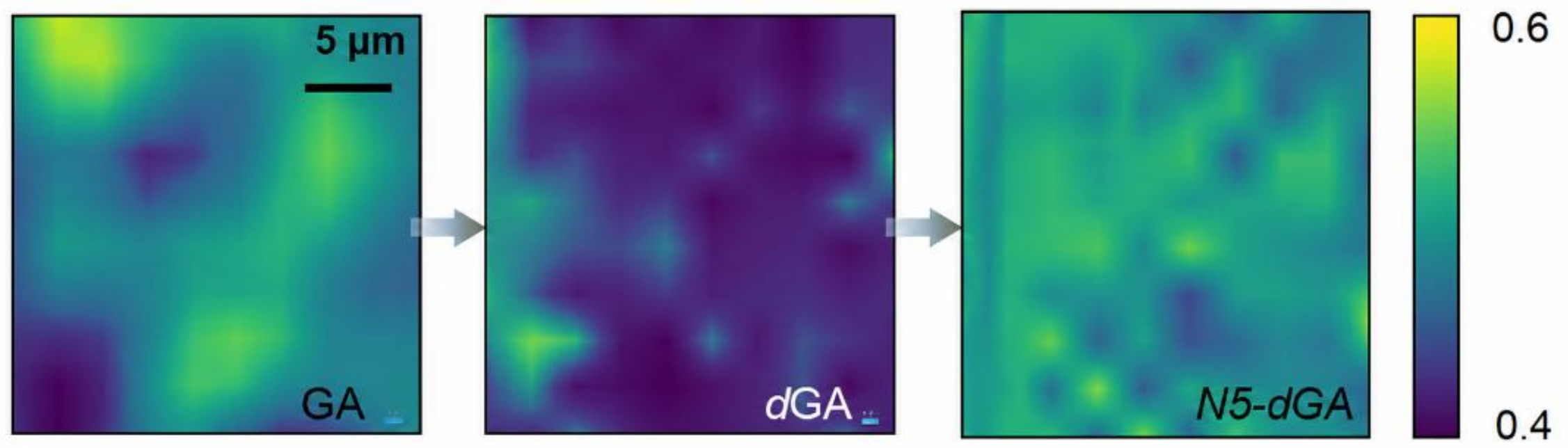

Supplementary Figure 31: Raman mappings of overall I_D/I_G for samples (scanning area: 20 μm×20 μm).

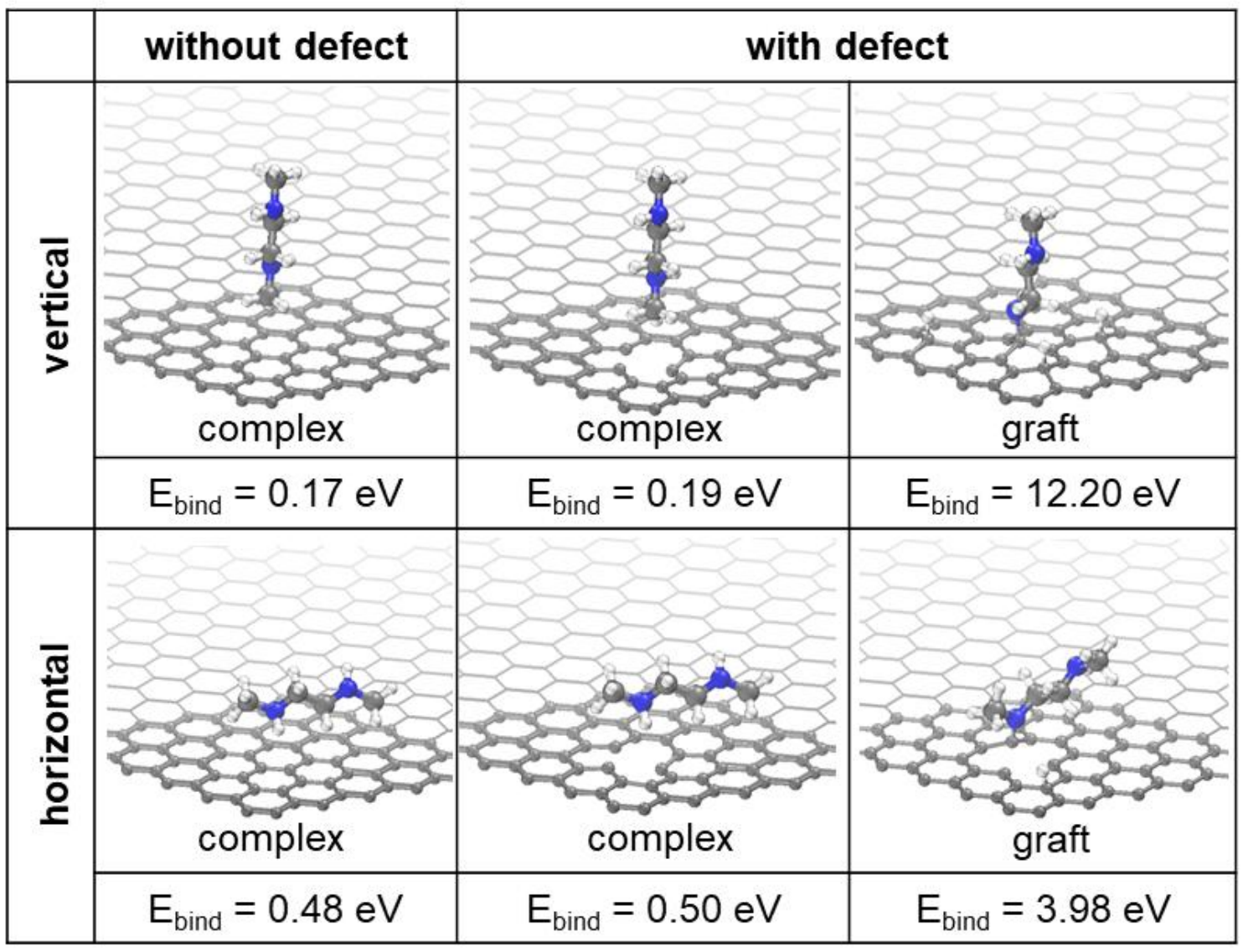

Supplementary Figure 32: The spatial structures and E_{bind} of *dual*-amine adsorbed or bound on the *d*GA surface.

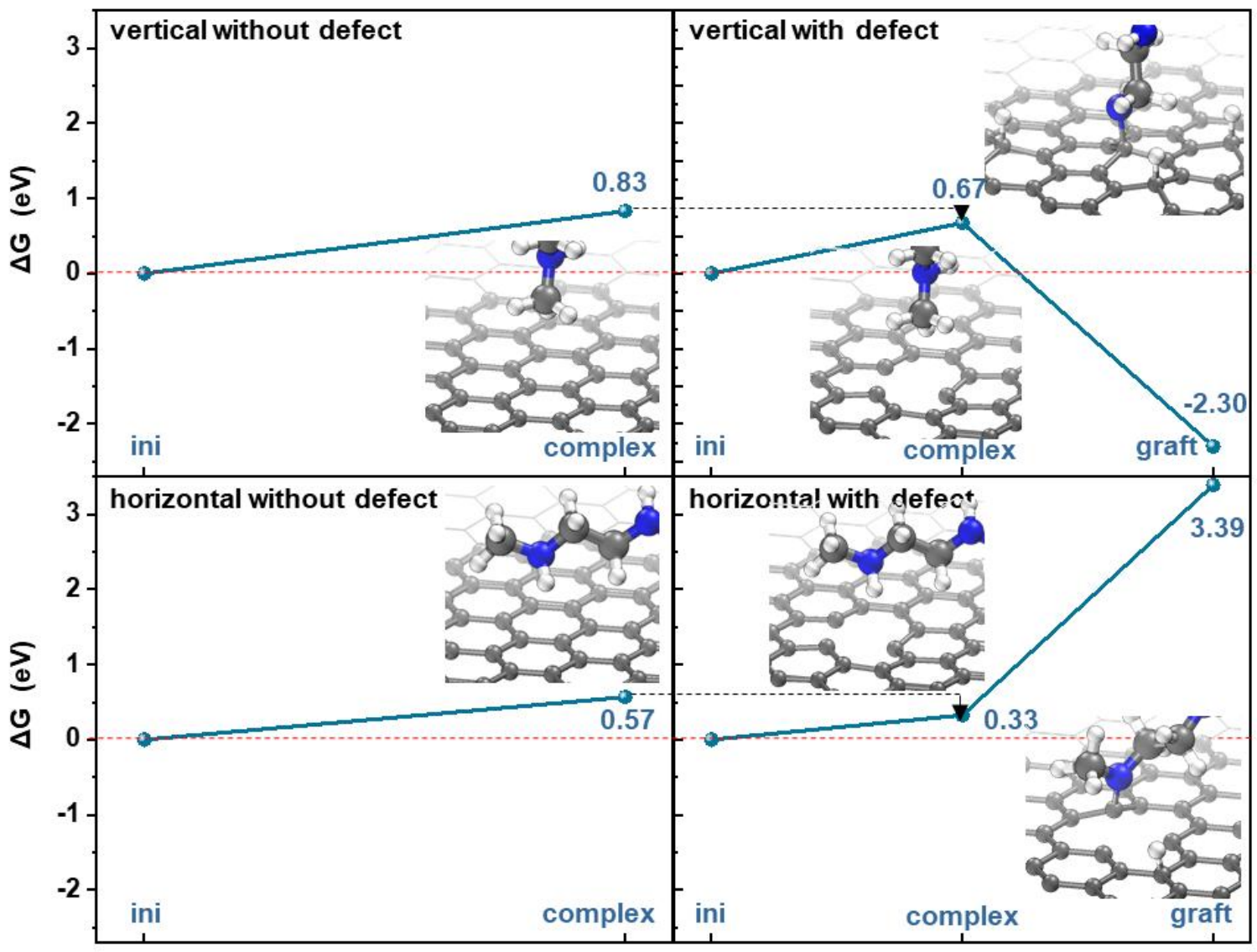

Supplementary Figure 33: The ΔG of *dual*-amine adsorbed or bound on the *d*GA surface.

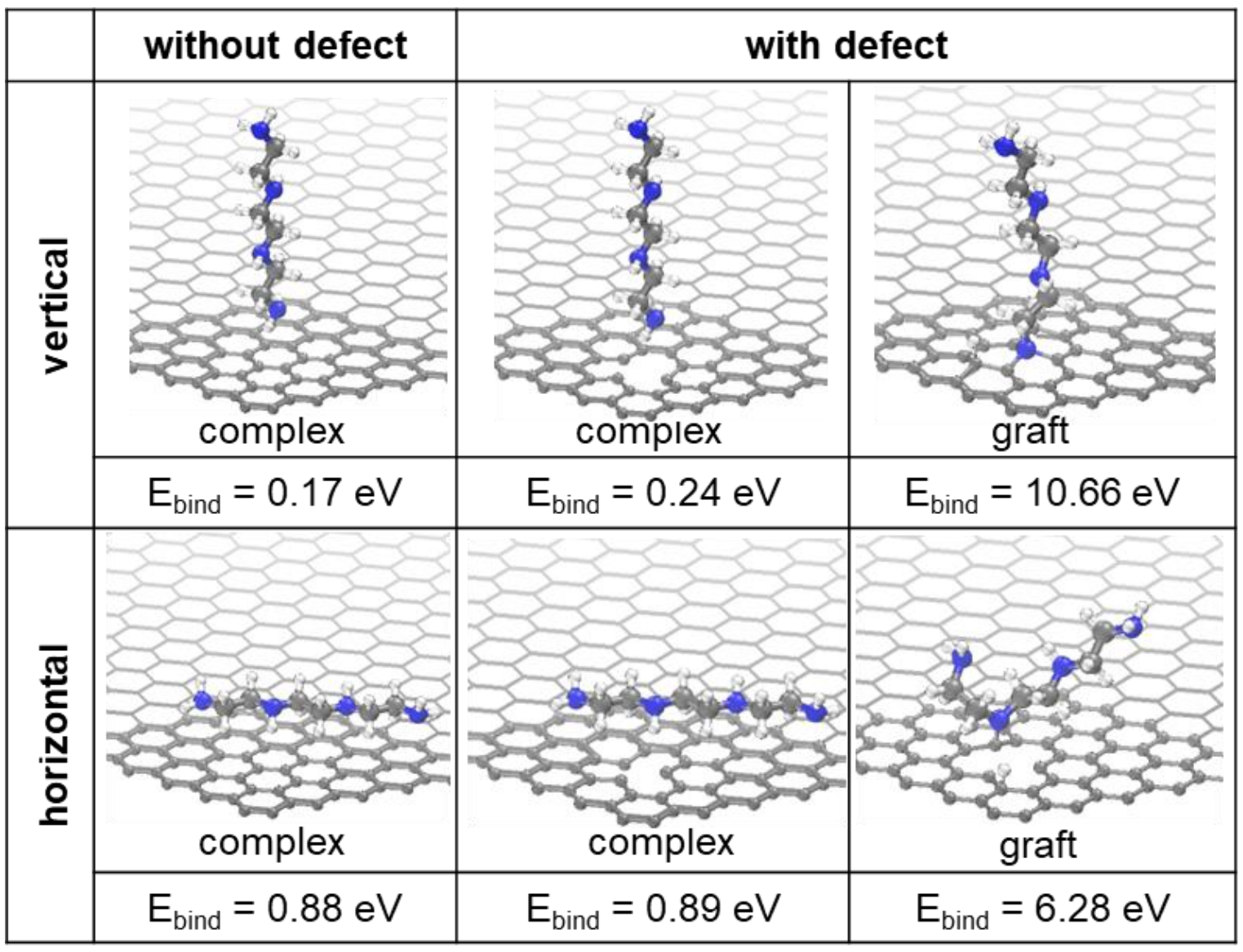

Supplementary Figure 34: The spatial structures and E_{bind} of *tetra*-amine adsorbed or bound on the *d*GA surface.

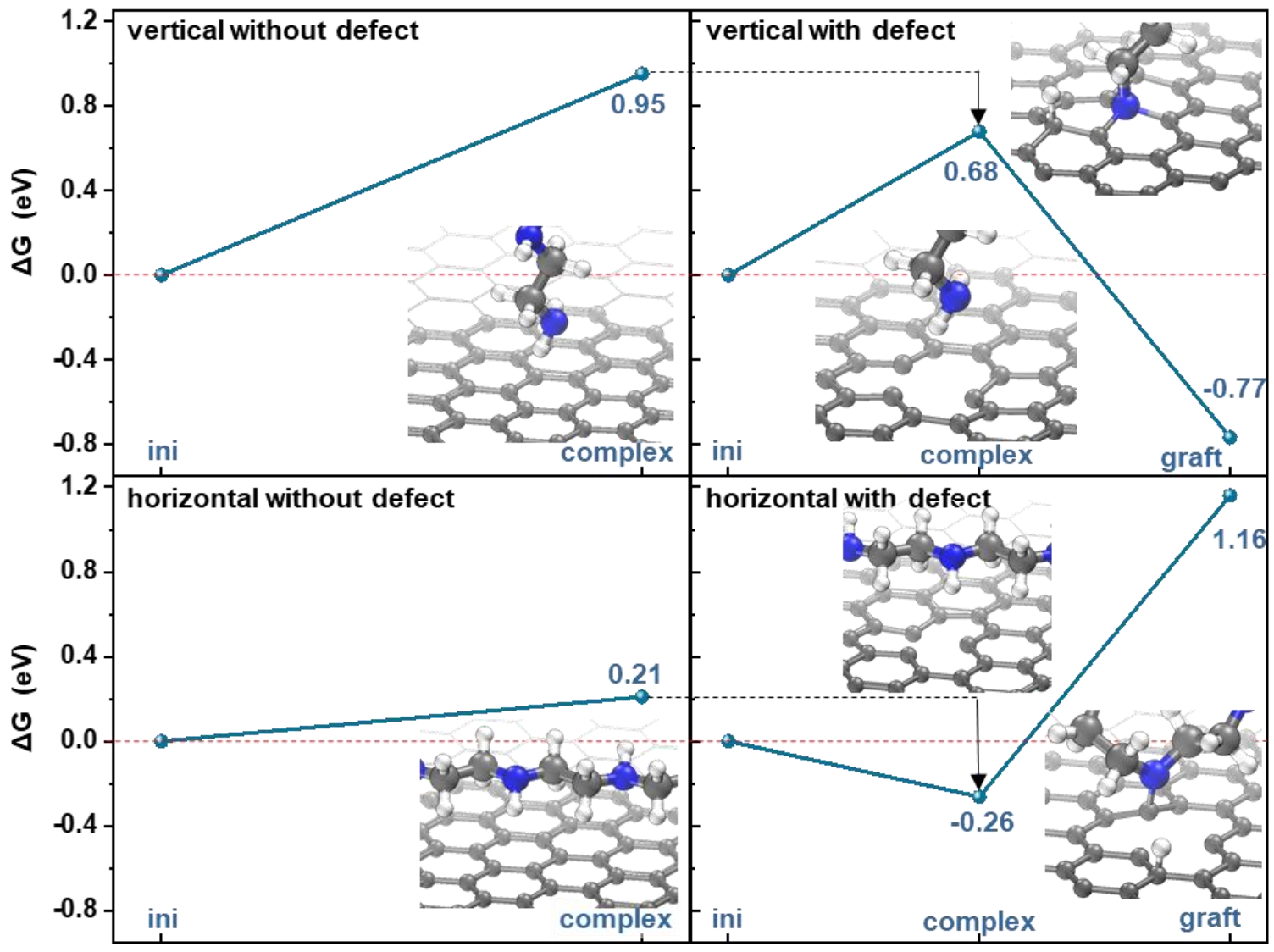

Supplementary Figure 35: The ΔG of *tetra*-amine adsorbed or bound on the *d*GA surface.

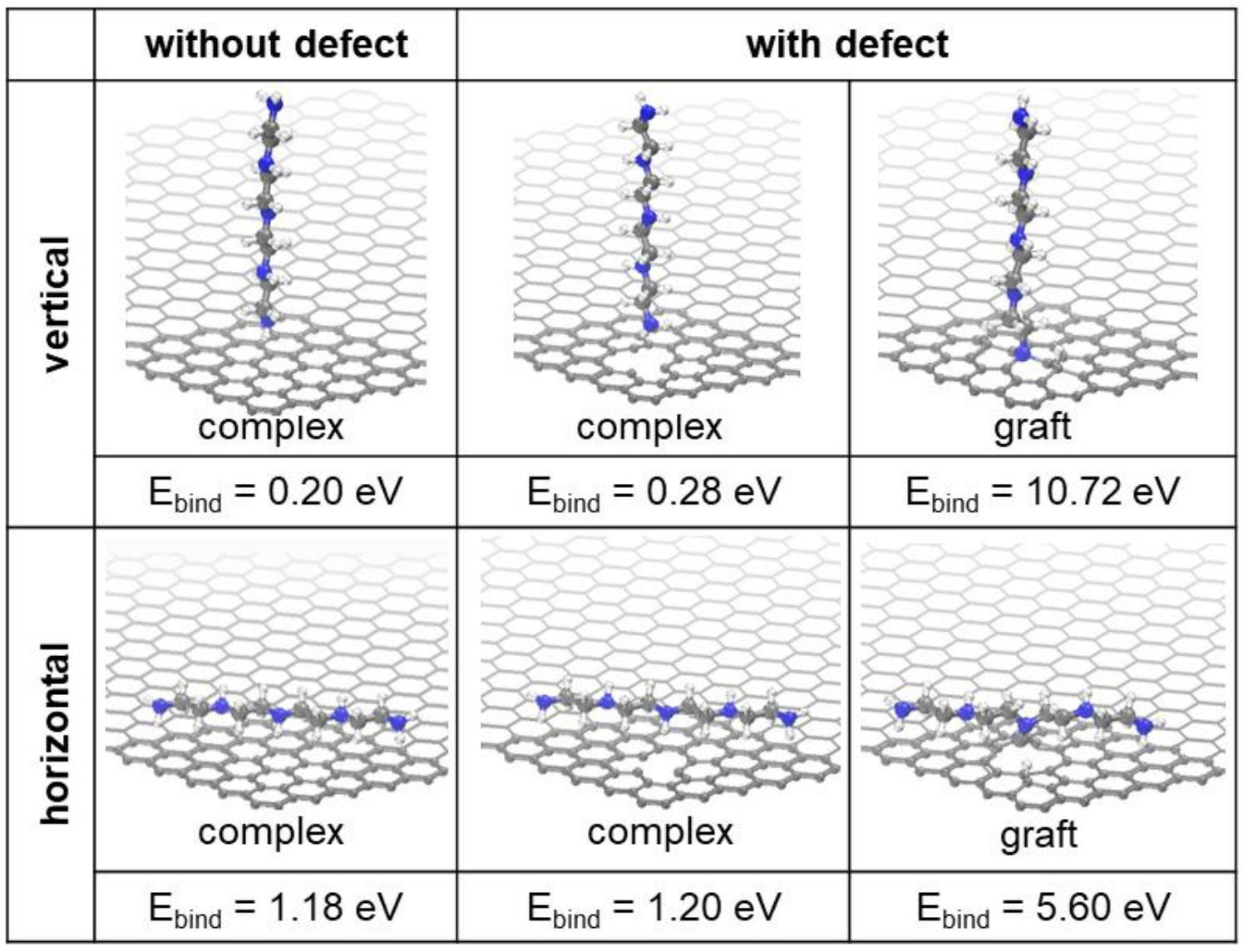

Supplementary Figure 36: The spatial structures and E_{bind} of *penta*-amine adsorbed or bound on the *d*GA surface.

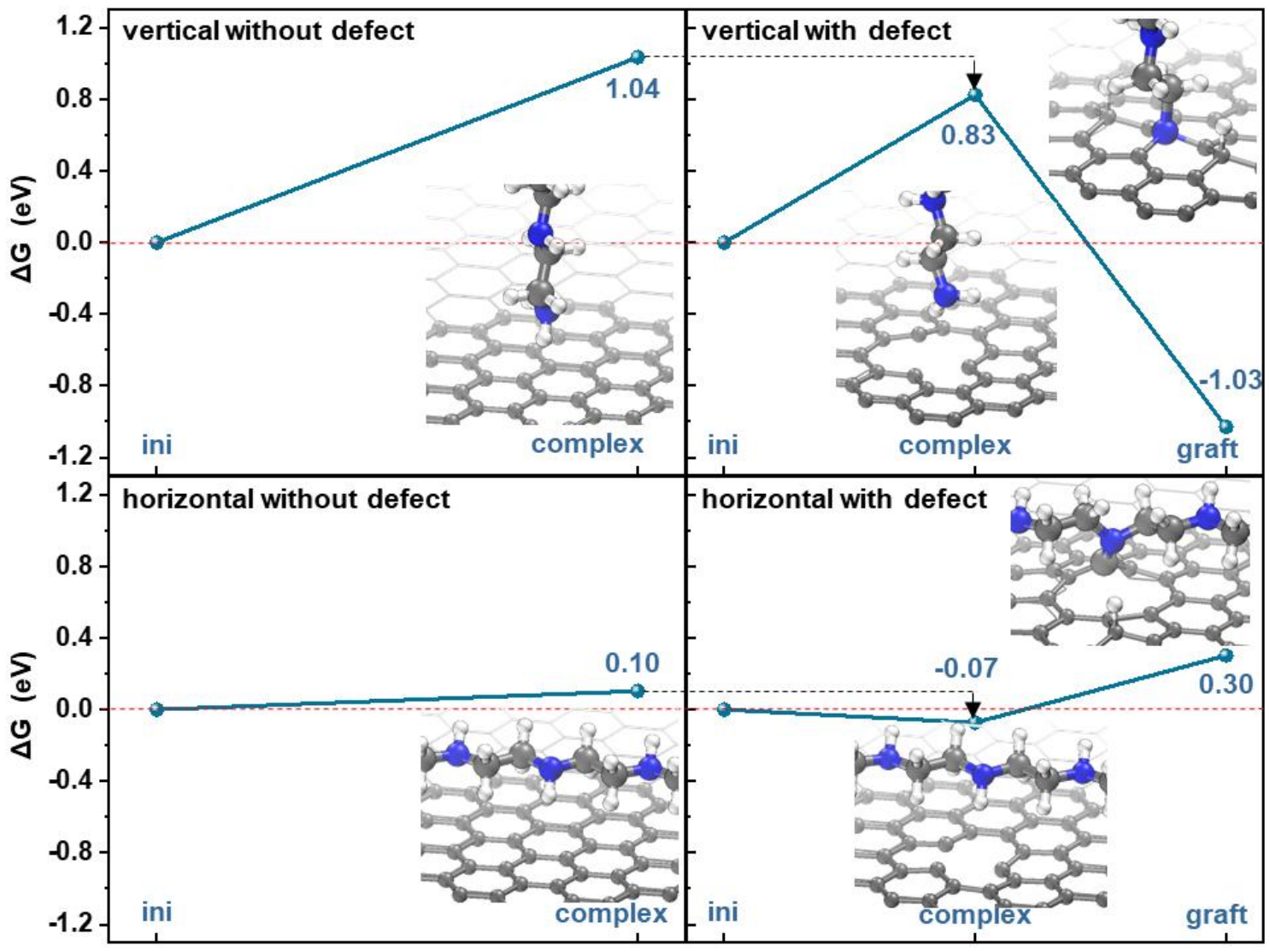

Supplementary Figure 37: The ΔG of *penta*-amine adsorbed or bound on the *d*GA surface.

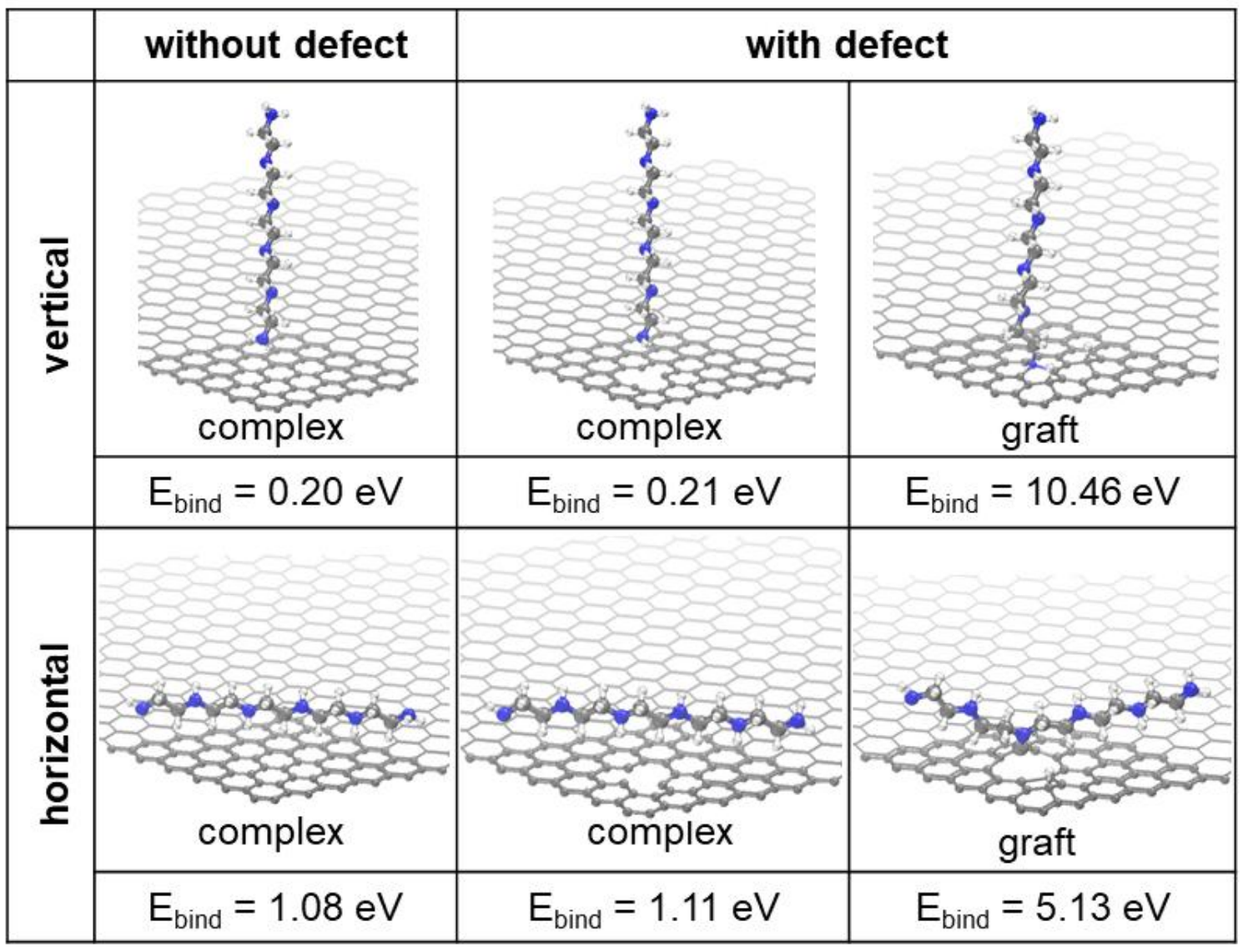

Supplementary Figure 38: The spatial structures and E_{bind} of *hex*-amine adsorbed or bound on the *d*GA surface.

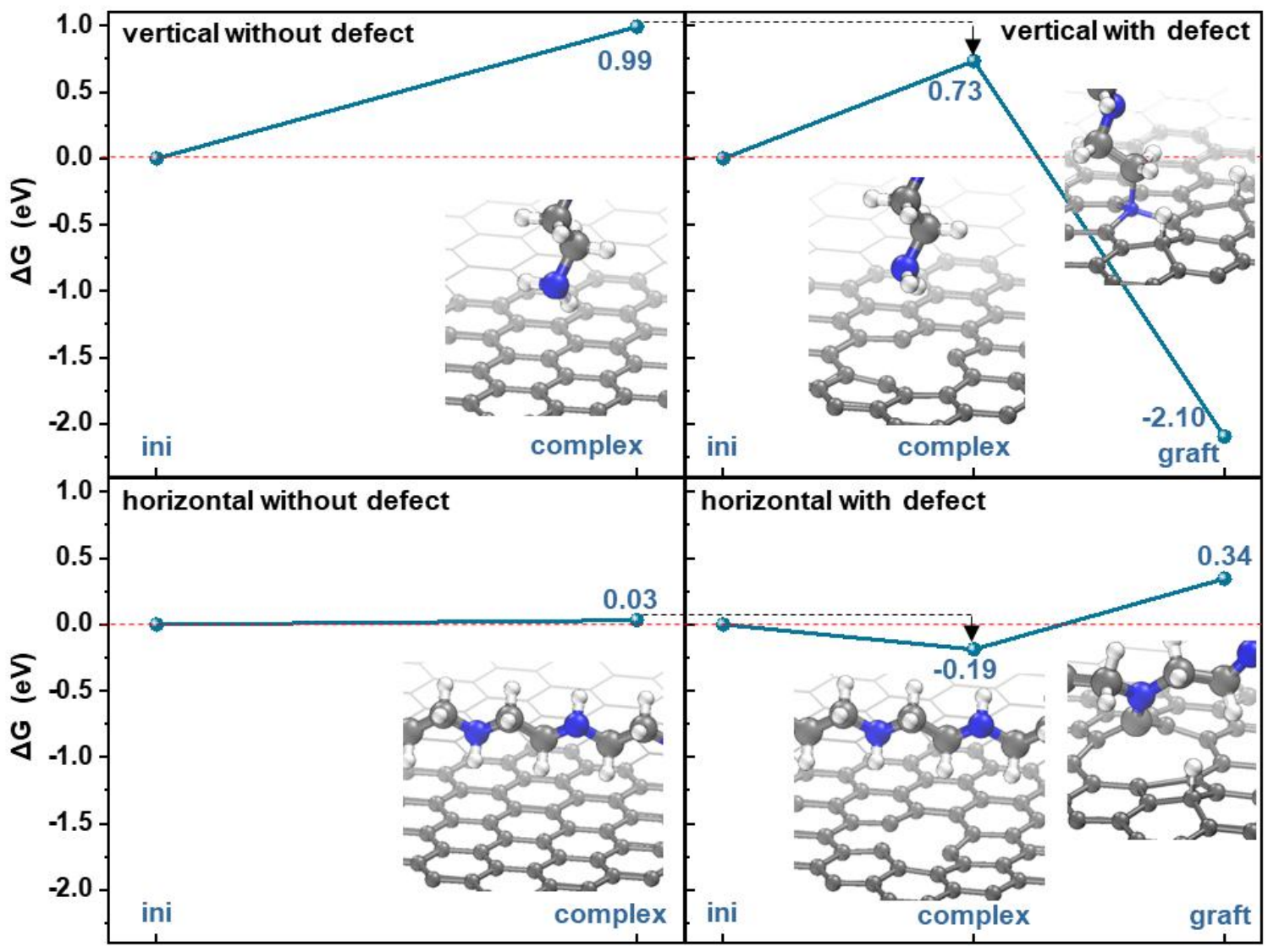

Supplementary Figure 39: The ΔG of *hex*-amine adsorbed or bound on the *d*GA surface.

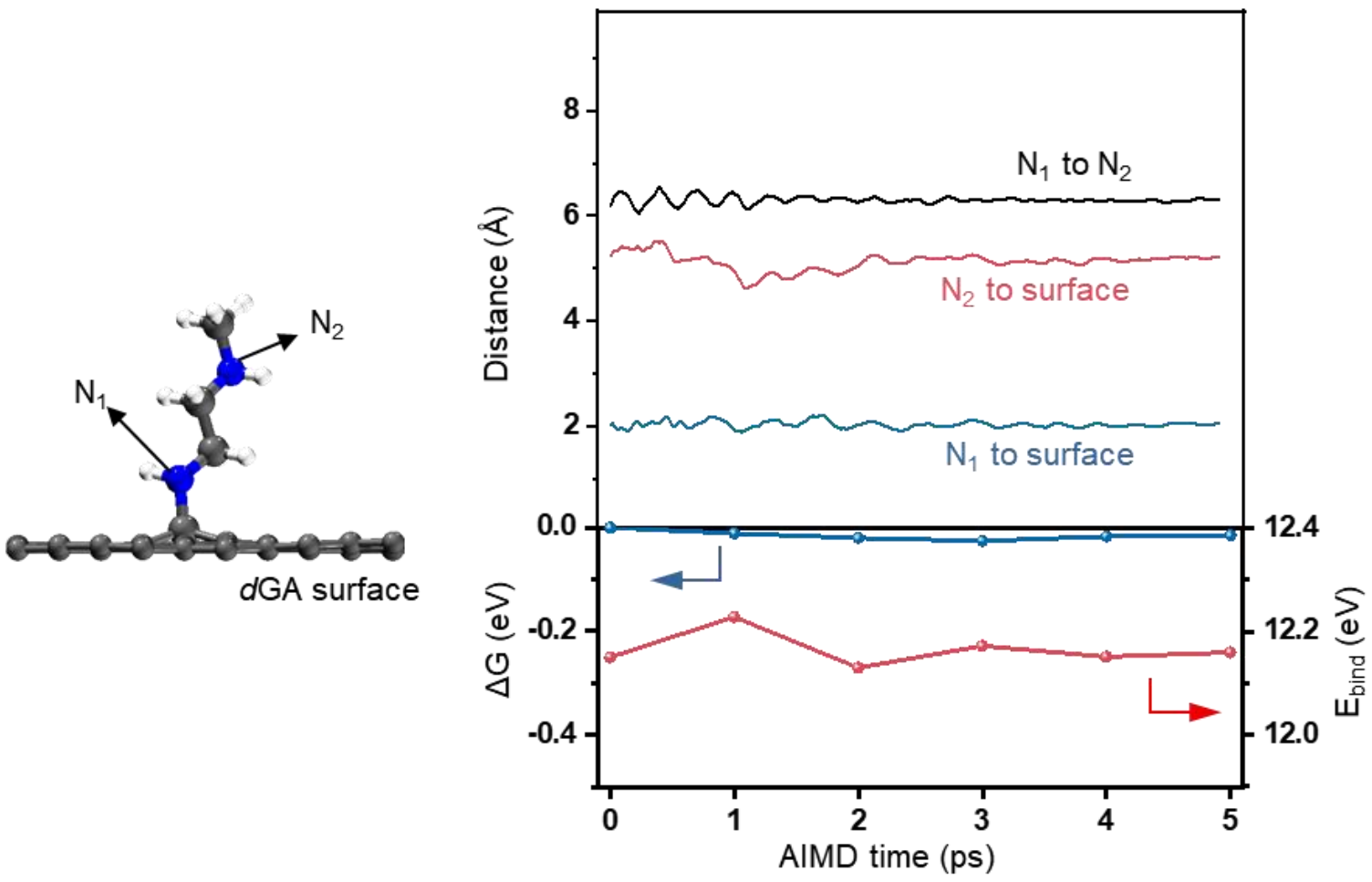

Supplementary Figure 40: Trajectory and energy changes of *N*2-*d*GA during the AIMD. Source data for Supplementary Figure 40 are provided as a Source Data file.

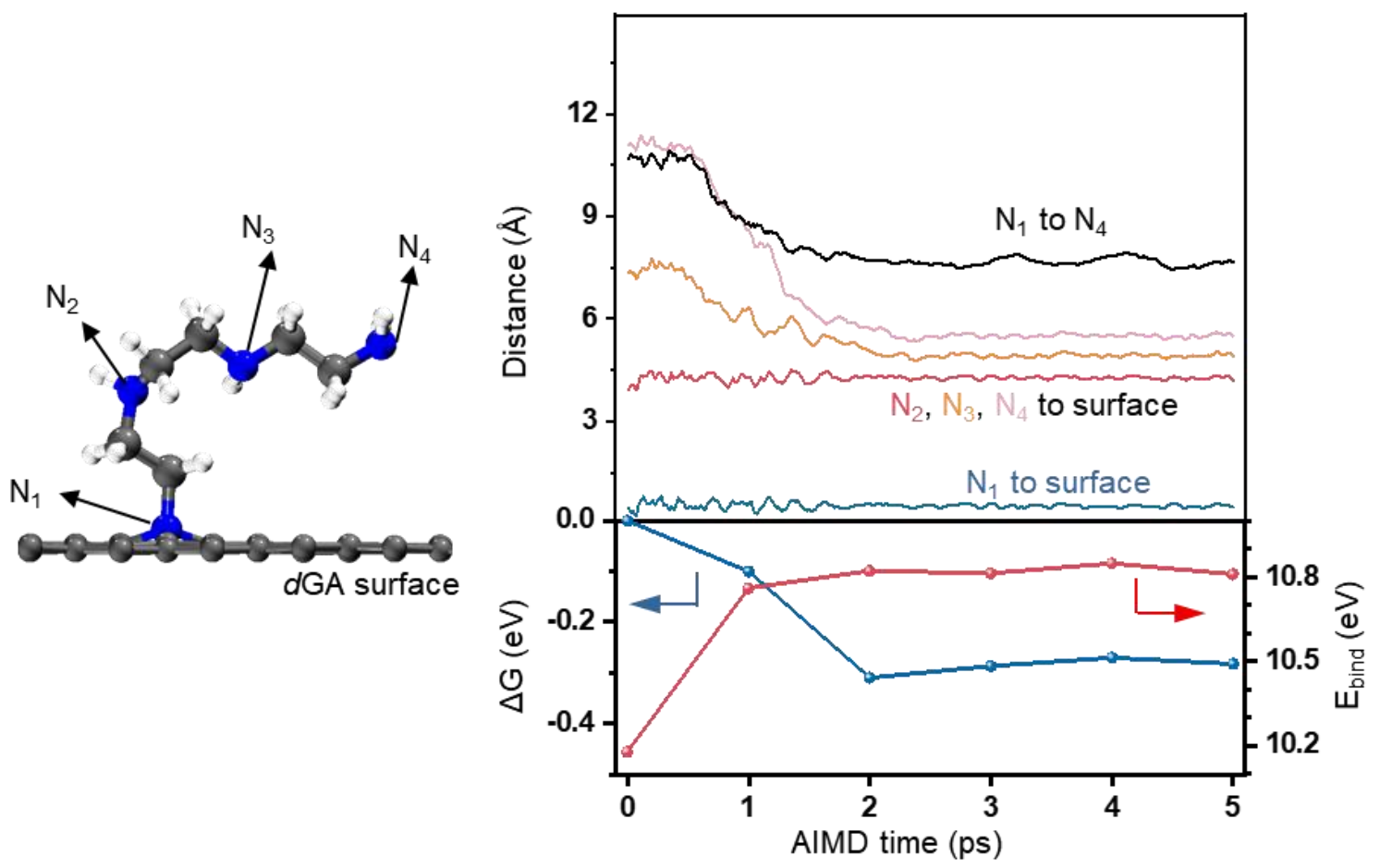

Supplementary Figure 41: Trajectory and energy changes of *N*4-*d*GA during the AIMD.

Source data for Supplementary Figure 41 are provided as a Source Data file.

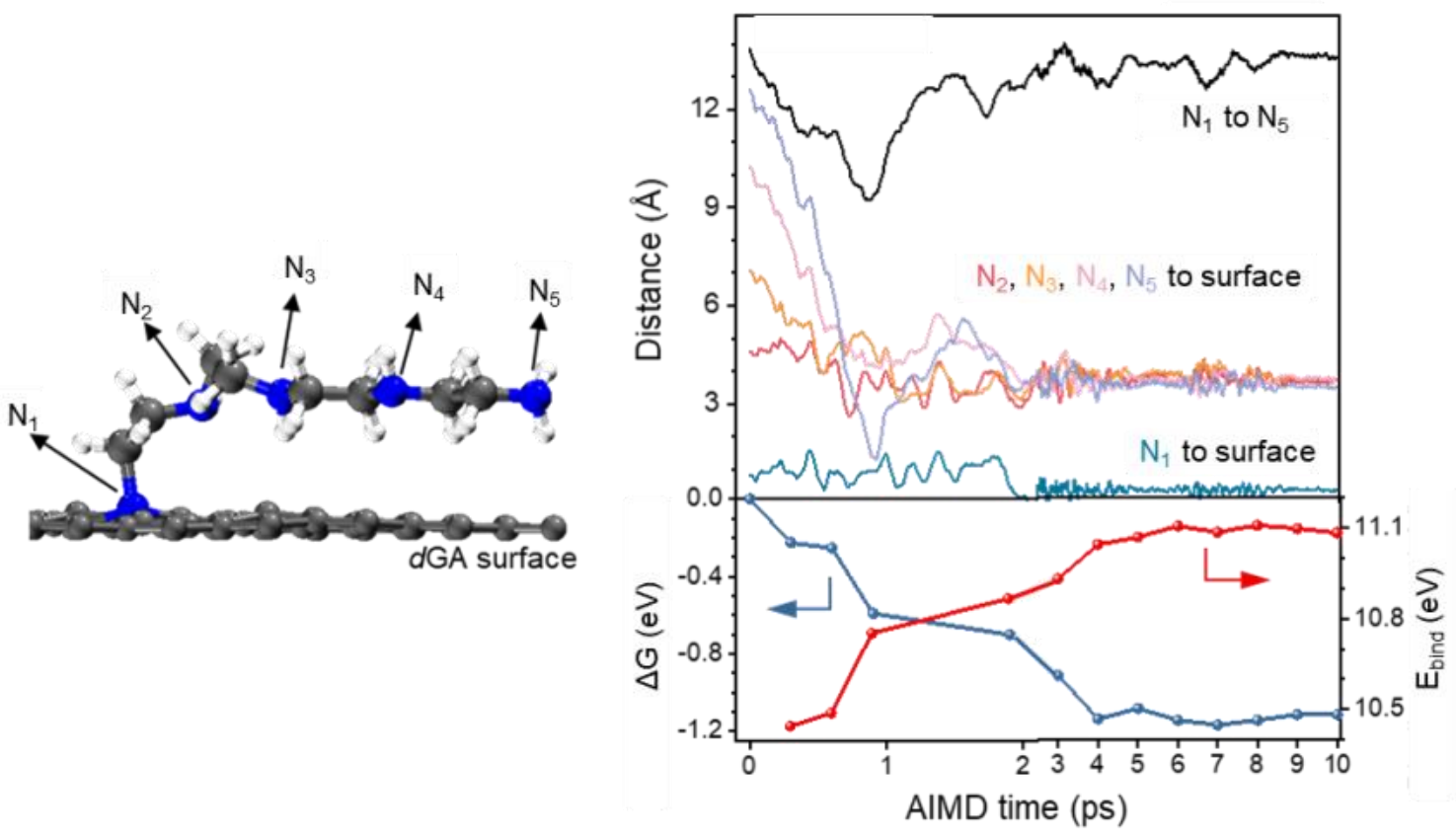

Supplementary Figure 42: Trajectory and energy changes of *N*5-*d*GA during the AIMD. Source data for Supplementary Figure 42 are provided as a Source Data file.

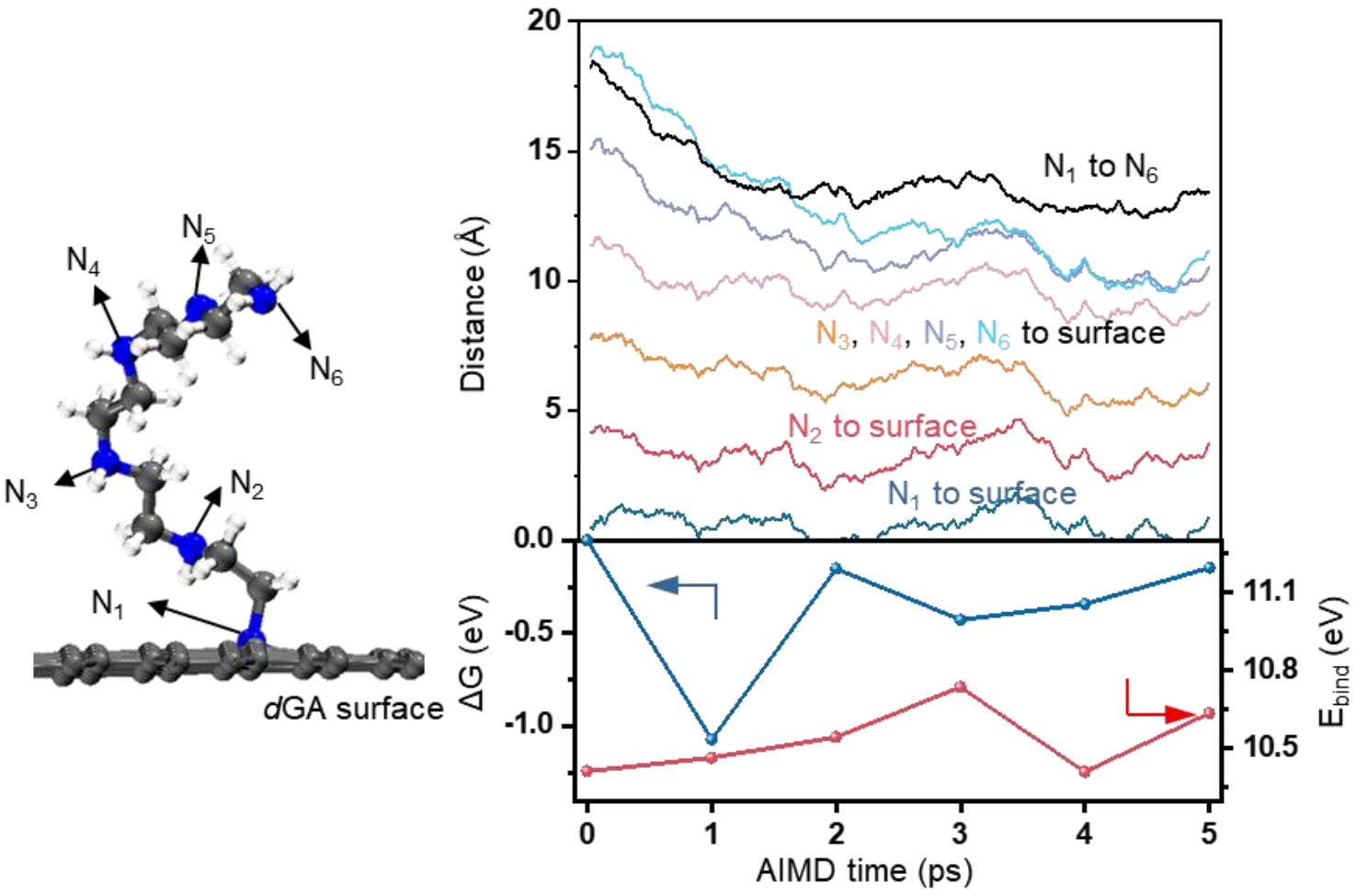

Supplementary Figure 43: Trajectory and energy changes of *N*6-*d*GA during the AIMD. Source data for Supplementary Figure 43 are provided as a Source Data file.

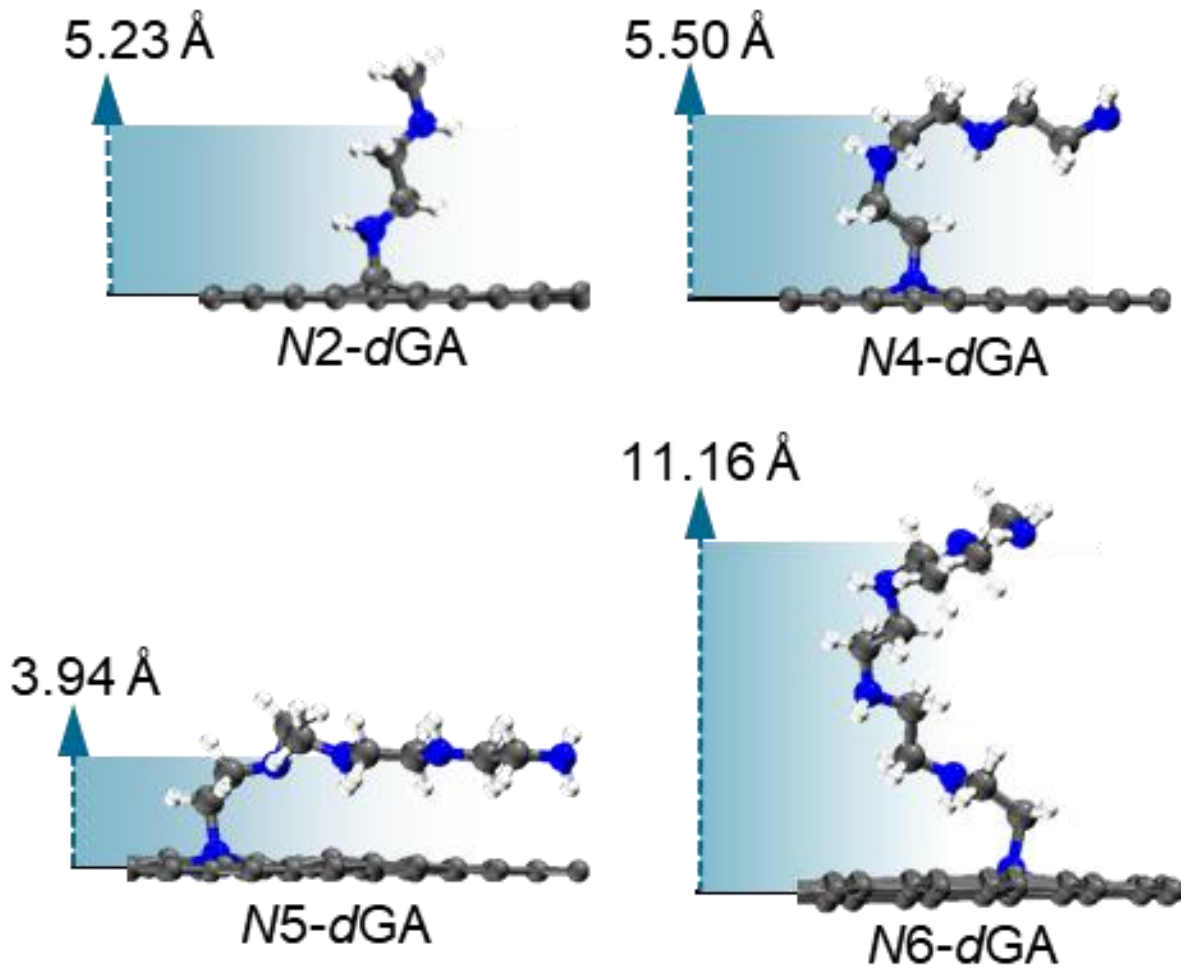

Supplementary Figure 44: Atomic structures and defect-distances of *N*-*d*GA.

3. Supplementary Tables

Supplementary Table 1: CO_2 uptake for DAC of various reported sorbents.

Type	Name	Adsorption temperature (°C)	Gas flow (mL min^{-1})	Gas velocity (m s^{-1})	q (mmol g^{-1}, dry)	q (mmol g^{-1}, humid)	Desorption temperature (°C)	Heat duty for regeneration (GJ t^{-1})	Volumetric Capacity (mol m^{-3})	Ref.
TSA/ TVSA	MIL-101(Cr) _50 wt % TEPA	-20	50	-	0.71^{a}	0.95^{a}	90	110 (kJ mol^{-1})^{c}	408.5	12
	Developed by Oy Hydrocell Ltd.	25	1000	2.62×10^{-2}	0.54	0.89	90	67.9 (kJ mol^{-1})^{c}	-	13
	2-UIO-EDA	25	10	5.89×10^{-3}	0.04	0.44	140	58.13 (kJ mol^{-1})^{c}	-	14
	MOR-type zeolites	30	50	-	1.05	1.02	100	-	997.5	
	13X-type zeolites	30	50	-	0.39	0.49	200~300	30 (kJ mol^{-1})^{c}	-	15
	SAPO-34+MOR	30	50	-	0.9	0.94	100	-	-	
	PEI-ePTFE/silica	35	1000	5.31×10^{-2}	1.08^{a}	1.5^{a}	110	67 (kJ mol^{-1})^{c}	-	16
	Polyam-N-Cu^{2+}	25	1500	5.1×10^{-2}	-	5.1	80	51.1 (kJ mol^{-1})^{c}	-	17
	COF-999	25	50	4.42×10^{-2}	0.96	2.05	60	53 (kJ mol^{-1})^{c}	-	18
	Lewatit® VP OC 1065	25	1000		0.63	1.16		54.9 (kJ mol^{-1})^{c}	777.2	19
ESA/ Joule heating	PEI-carbon fiber	25	100	5.89×10^{-4}	0.39^{a}	1.2^{a}	90~110<br>0.8~1.0 (A)^{b}	7.2	-	8
	Ag-NC coated fiber	25	30	4.19×10^{-2}	0.42	-	110<br>1.9 (A)^{b}	6.1	-	9
	PCS-OH	25	-	-	-	0.26	100	6.5~11.4	-	20
	Ad-0.48PE/0.32HP/0.20CB	25	400	0.13	-	2.4	90	3.5~3.7	-	21
	PEI-CNF	25	8(dry)<br>20(humid)	0.20(dry)<br>0.60(humid)	1.5	4.0	80	-	-	22
	*N*5-*d*GA	25	1000	1.00	2.93<br>2.91^{a}	6.57<br>6.41^{a}	80<br>0.6 (A)^{b}	3.4<br>60.7 (kJ mol^{-1})^{c}	52.6	This work

^{a}The pseudo-equilibrium CO_2 uptake for DAC (C/C_0=0.95).
^{b} The current intensity for CO_2 desorption, A.
^{c} Isosteric adsorption heat or adsorption enthalpy change, kJ mol^{-1}.

Supplementary Table 2: Parameters used for calculated ESA heat duty.

**Parameters**	**Symbol**	**Value**				**Unit**
CO_2 adsorption enthalpy changes	ΔH_{ads}	60.7				kJ mol^{-1}
Water adsorption enthalpy changes	ΔH_{H2O}	42				kJ mol^{-1}
Mass ratio of adsorbed water to CO_2	$r_{H2O/CO2}$	0.13				-
Mass ratio of column to sample	$r_{column/sample}$	10.42				-
Heat capacity of CO_2	$C_{p,CO2}$	0.85				kJ kg^{-1} K^{-1}
Heat capacity of H_2O	$C_{p,H2O}$	4.18				kJ kg^{-1} K^{-1}
Heat capacity of graphene	$C_{p,graphene}$	0.75				kJ kg^{-1} K^{-1}
Heat capacity of *penta*-amine	$C_{p,amine}$	2.0				kJ kg^{-1} K^{-1}
Heat capacity of quartz	$C_{p,quartz}$	0.8				kJ kg^{-1} K^{-1}
Current intensity of electrothermal desorption		**0.5**	**0.6**	**0.7**	**0.8**	A
Electrothermal temperature		60	80	105	129	℃
The amount of CO_2 desorbed		45	95	95	95	%
Temperature rise of sample	ΔT_{sample}	35	55	80	104	K
Temperature rise of column	ΔT_{column}	28.7	45	56.7	68	K

Supplementary Table 3: Parameters used for experimental ESA heat duty.

Parameters	Symbol	Value	Unit
Electrical power average value	$\overline{P}$	0.64	W
Thermal conductivity of air	λ_{air}	0.027	W m^{-1} $^{o}C^{-1}$
Thermal conductivity of quartz	$\lambda_{\mathrm{quartz}}$	1.40	W m^{-1} $^{o}C^{-1}$
Contact area between tested sample and quartz tube	A_{sample}	4.71×10^{-4}	m^2
Contact area between the column and air	A_{column}	9.97×10^{-5}	m^2
Temperature difference between tested sample and quartz tube surface	$T_{\delta_{\mathrm{sample}}}$	10	^{o}C
Temperature difference between column surface and air	$T_{\delta_{\mathrm{column}}}$	45	^{o}C
Distance from tested sample to quartz tube surface	δ_{sample}	0.0025	m
Distance from column surface to air	δ_{column}	0.01	m

Supplementary Table 4: TSA-DAC total equipment cost, equal to engineering, procurement, and construction as EPC cost, obtained by summing direct materials, installation, and engineering & procurement costs. Solid sorbent values were adapted from Young et al.[23] and scaled to 4000 tCO_2/year. All values are in M$US 2022. Note that values are rounded up to M$.

Component	Cost/M$	Sources
Contactor	3.7	Scaled from [23] and adjusted for inflation
Air blowers	0.1	Cost per blower derived from interview and scaled to 4000 tCO_2/year.
Vacuum pump	0.2	Scaled from [23] and adjusted for inflation
Vacuum pump	0.1	Scaled from [23] and adjusted for inflation
Gas storage balloon	0	Scaled from [23] and adjusted for inflation
Condensers	0.4	Scaled from [23] and adjusted for inflation
Switching valves	3.4	Scaled from [23] and adjusted for inflation
Initial sorbent	0.5	Scaled from [23] and adjusted for inflation
Buildings and land	1	Based on expert interview
Compressor	2.6	Scaled from [23] and adjusted for inflation
Installation cost	1.5	[23] difference between total installed and total direct materials cost. Adjusted for inflation. Air source heat pump cost included.
Engineering and Procurement cost	2	Sum direct materials and installed cost multiplied with epc-factor
**Sum**	**15.5**	

Supplementary Table 5: Cost components included in total overnight cost (TOC) including EPC, contingencies, owner's costs, spare parts, and start-up expenses.

Cost component	Value	Source
Direct materials cost	See Supplementary Table 3	
Installation cost	See Supplementary Table 3	
EPC factor	15% of direct materials and installation costs	23-26
= EPC cost	Total direct materials and installation costs multiplied by (1 + EPC factor)	-
Project contingencies	35% of total EPC cost	23,24,27
Process contingencies	Process-dependent % of total EPC cost.	
**= TPC as sum of EPC and contingencies**		
Owners' cost	7% of TPC	23-25
Spare parts	0.5% of TPC	23-25
Start-up capital	2% of TPC	23-25
Start-up labour	0.25 years of direct and indirect labour	23-25
Start-up fuel/energy	0.02 years of fuel/energy	23-25
Start-up chemicals/minerals	0.08 years of chemicals/minderals	23-25
**= TOC as sum of TPC, owners' cost, spart parts and start-up cost**		

Supplementary Table 6: Fixed OPEX in $ t^{-1} CO_2 per year including direct and indirect labor, maintenance, insurance, local taxes and fees, and adjusting for plant size, location-specific productivity factors, and employee salaries. All costs and factors were taken from sources indicated and converted to $US 2022.

Cost component	Value	Source
Operator salary	72186 $2022/year/employee	[23-25]; adjusted for inflation
Direct labour cost	Scaled linearly based on 278 employees at a 1 Mt-CO_2 year-1 plant in the United States.	[23,24,28]
Maintenance cost	1.5% of TPC	[23-25]
Indirect labour cost	30% of direct labour and maintenance	[23-25]
Insurance	0.5% of TPC	[23-25]
Local taxes and fees	0.5% of TPC	[23-25]

Supplementary Table 7: Variable OPEX in $/tCO_2$ per year including cost of energy, chemicals/minerals, CO_2 transport and CO_2 storage. All costs were taken from sources indicated and converted to $US 2022.

Cost component	Value	Source
Energy (electricity, and heat)	See Supplementary Table 5	-
Water	$0.21 m^{-3} of water pumped. See requirements in Supplementary Table 5.	[23-25]
Unit cost of cooling water	0.25 $ m^{-3}	[23,24]
Chemicals/minerals	See Supplementary Table 5.	-
CO_2 transport distance	Transport distance assumed to be 50km.	[23-25]
CO_2 transport cost	0.09 ($ t^{-1} CO_2) km^{-1}	$5-12 t^{-1} CO_2 (for distances from 64 km up to 112 km)[29], which is 0.075 – 0.1 ($ t^{-1} CO_2) km^{-1}. Inflation adjusted mean chosen.
CO_2 storage cost	Triangular distribution: min: 11 $2022 t^{-1} CO_2, mode: 19 $2022 t^{-1} CO_2, max: 27 $2022 t^{-1} CO_2	Range of $9.74- $23.20 $2019 t^{-1} CO_2[30]. Inflation adjusted and used as triangular distribution in input data.

Supplementary Table 8: Process specific parameters of ESA-DAC.

Parameters	Value	Unit
Initial plant capacity	4000	t CO_2 a^{-1}
adsorption capacity	6.57	mmol g^{-1}
adsorption time	6.00	h
desorption time	2.00	h
cooling time	4.00	h
Capacity factor	0.98	
Regenerative energy consumption	3.40	GJ t^{-1} CO_2
Cost of graphene aerogel	0.16	M$ t^{-1}
Cost of *penta*-amine	0.004148	M$ t^{-1}
Cost of synthesis and post-processing[a]	0.003	M$ t^{-1}
Regeneration temperature	80.00	°C
Mass fraction of *penta*-amine	66.7	%
Mass fraction of graphene aeroge	33.3	%
Mass of initial sorbents	9.48	t
Cost of initial sorbents[b]	0.56	M$
CO_2 release ratio	95	%
Electricity cost of solar PV	39	$ MWh^{-1}
Electricity cost of wind	37	$ MWh^{-1}
Carbon intensity (x)	0.0176	
Additional electrical energy (like air blower, etc).	0.99	GJ t^{-1} CO_2

[a]The synthesis and post-processing cost was attributed to thermal reduction (26%), plasma treatment (22%), amine functionalization (12%), washing (20%), drying (10%), and auxiliary handling (10%).

[b]The resulting sorbent cost ($C_{sorbent}$) was calculated to be 0.059 M$ t^{-1}: $C_{sorbent}$=33.3 wt%×Cost of graphene aerogel+66.7 wt%×Cost of *penta*-amine+Cost of synthesis and post-processing. For an initial sorbent mass of 9.48 t, this gives an initial sorbent cost of approximately 0.56 M$.

4. Supplementary References

1 Kresse, G. & Furthmuller, J. Efficiency of ab-initio total energy calculations for metals and semiconductors using a plane-wave basis set. *Comput. Mater. Sci.* **6**, 15-50, (1996).

2 Lu, T. & Chen, Q. van der Waals potential: An important complement to molecular electrostatic potential in studying intermolecular interactions. *J. Mol. Model.* **26**, 315, (2020).

3 Humphrey, W., Dalke, A. & Schulten, K. VMD: Visual molecular dynamics. *J. Mol. Graph. Model.* **14**, 33-38, (1996).

4 Luo, C., Zheng, Y., Ding, N., Wu, Q. L. & Zheng, C. G. SGCS-made ultrafine CaO/Al_2O_3 sorbent for cyclic CO_2 capture. *Chin. Chem. Lett.* **22**, 615-618, (2011).

5 Perdew, J. P., Burke, K. & Ernzerhof, M. Generalized gradient approximation made simple. *Phys. Rev. Lett.* **78**, 1396, (1997).

6 VandeVondele, J. & Hutter, J. Gaussian basis sets for accurate calculations on molecular systems in gas and condensed phases. *J. Chem. Phys.* **127**, 114105, (2007).

7 Dawson, R. *et al.* 'Dry bases': Carbon dioxide capture using alkaline dry water. *Energy Environ. Sci.* **7**, 1786-1791, (2014).

8 Lee, W. H. *et al.* Sorbent-coated carbon fibers for direct air capture using electrically driven temperature swing adsorption. *Joule* **7**, 1241-1259, (2023).

9 Lee, Y. H. *et al.* Design of electrified fiber sorbents for direct air capture with electrically-driven temperature vacuum swing adsorption. *Adv. Mater.*, e04542, (2025).

10 Li, Q., Duan, J. & Jin, W. Efficient CO_2/N_2 separation by mixed matrix membrane with amide functionalized porous coordination polymer filler. *Chin. Chem. Lett.* **29**, 854-856, (2018).

11 Chu, K. H. Fixed bed sorption: Setting the record straight on the Bohart-Adams and Thomas models. *J. Hazard. Mater.* **177**, 1006-1012, (2010).

12 Rim, G. *et al.* Sub-ambient temperature direct air capture of CO_2 using amine-impregnated MIL-101(Cr) enables ambient temperature CO_2 recovery. *JACS Au* **2**, 380-393, (2022).

13 Elfving, J., Bajamundi, C., Kauppinen, J. & Sainio, T. Modelling of equilibrium working capacity of PSA, TSA and TVSA processes for CO_2 adsorption under direct air capture conditions. *J. CO_2 Util.* **22**, 270-277, (2017).

14 Dong, H. *et al.* Amine-functionalized quasi-MOF for direct air capture of CO_2. *Acs Materials Letters* **5**, 2656-2664, (2023).

15 Fu, D. & Davis, M. E. Toward the feasible direct air capture of carbon dioxide with molecular sieves by water management. *Cell Rep. Phys. Sci.* **4**, 101389, (2023).

16 Min, Y. J., Ganesan, A., Realff, M. J. & Jones, C. W. Direct air capture of CO_2 using poly(ethyleneimine)-functionalized expanded poly(tetrafluoroethylene)/silica composite structured sorbents. *ACS Appl. Mater. Interfaces* **14**, 40992-41002, (2022).

17 Chen, H., Dong, H., Shi, Z. & SenGupta, A. K. Direct air capture (DAC) and sequestration of CO_2: Dramatic effect of coordinated Cu(II) onto a chelating weak base ion exchanger. *Sci. Adv.* **9**, eadg1956, (2023).

18 Zhou, Z. *et al.* Carbon dioxide capture from open air using covalent organic frameworks. *Nature* **635**, 96-101, (2024).

19 Fu, Y. Experimental study of different sorbents for direct air capture. *KTH Royal Institute of Technology*, (2024).

20 Li, H. *et al.* Capturing carbon dioxide from air with charged-sorbents. *Nature* **630**, 654-659, (2024).

21 Zhao, S. *et al.* Electrically conductive amine-functionalized adsorbents driven by joule heating for direct air capture. *ACS Sustainable Chem. Eng.* **13**, 14949-14960, (2025).

22 Wu, R. *et al.* Distributed direct air capture by carbon nanofiber air filters. *Sci. Adv.* **11**, eadv6846, (2025).

23 Young, J. *et al.* The cost of direct air capture and storage can be reduced via strategic deployment but is unlikely to fall below stated cost targets. *One Earth* **6**, 899-917, (2023).

24 Sievert, K., Schmidt, T. S. & Steffen, B. Considering technology characteristics to project future costs of direct air capture. *Joule* **8**, 979–999, (2024).

25 IEAGHG. Techno-economic evaluation of smr based standalone (Merchant) hydrogen plant with CCS. *Technical Review* **2017-02**, 286, (2017).

26 NETL. Quality guidelines for energy systems studies: Cost estimation methodology for NETL assessments of power plant performance. *National Energy Technology Laboratory*, (2011).

27 International, A. Cost estimate classification system as applied in engineering, procurement, and construction for the process industries. (2020).

28 Larsen, J., Herndon, W. & Hiltbrand, G. The employment opportunities associated with scale-up of Direct Air Capture (DAC) technology in the US. (2020).
29 Psarras, P. C. *et al.* Carbon capture and utilization in the industrial sector. *Environ. Sci. Technol.* **51**, 11440-11449, (2017).
30 Smith, E. *et al.* The cost of CO_2 transport and storage in global integrated assessment modeling. *Int. J. Greenh. Gas Con.* **109**, 103367, (2021).
