# Supplementary material for: Proton trap engineered electric swing adsorption for scalable and cost-effective direct air capture
Source: Nat Commun. 2026 Jul 21;17:8885. doi: 10.1038/s41467-026-75916-7 (PMC13500661; doi:10.1038/s41467-026-75916-7)
Supplement: Supplementary file 2 [file 41467_2026_75916_MOESM2_ESM.pdf]

nature portfolio

Peer Review File

Proton Trap Engineered Electric Swing Adsorption for Scalable and Cost-Effective Direct Air Capture

Corresponding Author: Professor Shihan Zhang

This file contains all reviewer reports in order by version, followed by all author rebuttals in order by version.

Version 0:

Reviewer comments:

Reviewer #1

(Remarks to the Author)
An innovative amine-functionalized graphene aerogel (N-dGA) with point defect engineering achieves a record CO_2 uptake of 6.57 mmol g–1 at 400 ppm CO_2 and 1.0 m s–1 gas velocity, surpassing traditional DAC sorbents, and proposing a low-cost electro-thermal desorption technology below conventional DAC energy consumption levels. Point defects in N-dGA enable efficient dispersion of amines and serve as proton acceptors, could revolutionize ESA materials for DAC by facilitating simultaneous zwitterion formation and proton transfer within a single elementary reaction, enhancing CO_2 adsorption capacity, stability, and electrical desorption rates. As a whole this is a very nice proof of concept. But important points shall be addressed prior to publication in a journal such as Nature Communications :
1. The authors have employed diverse experimental methodologies and computational analyses to elucidate the interaction dynamics between amines and point defects. However, the mechanism underlying the attraction of amines towards point defects remains ambiguous. Why amines are so attracted towards defects shall be better explained. The inquiry seeks to discern the specific atoms or groups involved in the interaction and subsequent bond formation.
2. The synthetic procedure lacks details. Please specify the quantity ratio of graphene aerogels to amines, the solvent and synthetic conditions, and the washing and activation methods employed for the sample.
3. One shall calculate the Qst at different temperatures.
4. The record value mentioned by the authors is shown in mmol.g-1. However, in most cases performances are given in volume/volume values; here the graphene-based material has probably a very low bulk / packing density which would lead to a much lower v/v performance in comparison with denser materials such as porous silica / MOFs amine grafted materials. Thus, the referee recommends strongly that authors discuss this point and give an approximative v/v value and compare this value with the other benchmarks in this field. Accordingly a table such as the one figure 2d
5. Amine based materials suffer often from strong stability / cyclability issues. Here the ESA process is used to desorb the CO_2 from the amine grafted aerogel; have authors look at the longer term stability upon cycling of their material ? Here, if the authors have carried out 1,000 decompression cycles, the separation of CO_2 over N_2 has only been carried out for three cycles (without ESA) and the referee does not understand if multiple ESA cycling has been carried out. Note that any real CCS process needs a material that can still be efficient upon >100,000 cycles…This is here particularly important as overheating through ESA could lead to hot spots that, in the presence of oxygen, could degrade the grafted amines…
6. ESA processes are not limited to bare porous carboneous materials, for instance when dealing with insulating porous materials that are hybridised with electronically conductive carbon additives. In addition defective porous graphene oxides are not expected to exhibit a high electronic conductivity; thus one could expect the inorganic or hybrid carbon composites to exhibit a good conductivity suitable for ESA as shown before for Microwave swing adsorption. This point shall be discussed, probably in the outlooks, to determine if the new mechanism in play either is superior to other non carbon state-of-the-art ESA materials or could even be applied to other types of defective inorganic or hybrid adsorbents (of course being set conductive enough with carbon additives).
7. The plasma generation of defects is a rather expensive and poorly scalable technique. As DAC is a very large-scale process, it this amine grafted defective GO suitable for real applications (note that GO itself is not a cheap material, although being already commercialised). Authors shall discuss the cost and scalability of their materials.
Other points :
1. The authors presented the Isotherm Absorption capacity versus time at 1 bar. Please provide the isotherm illustrating absorption pressure ranging from 0 to 1 bar.
2. The authors shall carry out solid-state NMR analysis of CO_2 adsorbed onto N-dGA to elucidate the interaction between CO_2 and amine.

Reviewer #2

(Remarks to the Author)
This paper presents important findings on point defect engineering on graphene aerogel for direct air capture using electric swing adsorption (ESA). However, the paper's primary limitation is its lack of novelty. Please find detailed comments and suggestions below:

Novelty and Contribution to the Field: The paper provides valuable insights into CO2 capture in low concentration with relatively high velocity. However, it would benefit from a clearer articulation of its novel contributions compared to existing literature. Point defect engineering on graphene aerogel is not a new idea. It is already known. Specifically, how does this study advance our understanding beyond what is already known? Highlighting unique aspects or findings not previously explored would strengthen the paper's contribution to the field. No competitive adsorption study was conducted. Under high humid conditions, adsorption of water vapor onto a sorbent would likely change its operational performance. Likewise, molecular nitrogen could also compete with CO2, limiting the adsorption capacity of the material. No cost-benefit analysis was done to show the merits of using renewable electricity. Multiple adsorption-desorption cycles were not investigated to demonstrate the regeneration of the material and the retention of the adsorption performance with no loss of the integrity of the material. I do not find any kinetic studies i.e. the rate of uptake of CO2 under different operating conditions typically encountered in industrial flue gases.

Statistical Analysis and Presentation of Results: The paper could benefit from a more detailed explanation of the statistical methods used, including the rationale for choosing particular models and statistical tests. Additionally, providing a clearer interpretation of the statistical significance and practical implications of the findings would be beneficial.

Discussion of Limitations and Future Research: While the paper acknowledges certain limitations, a more comprehensive discussion of these, including potential biases and the generalizability of the results, would be beneficial. Additionally, suggesting specific avenues for future research based on the study's findings and limitations could provide valuable guidance for the field.

Practical Implications and Policy Recommendations: It would be helpful to discuss the key findings in more depth, particularly how they can be realistically implemented in urban environments for direct CO2 capture from ambient air or in specific power plants or industrial environments. This could include a discussion of the feasibility, potential challenges, and ways to overcome these challenges.

Enhancing the Literature Review: The literature review could be expanded to provide a more comprehensive background, particularly in terms of comparing and contrasting this study's approach and findings with previous research. This would help in positioning the paper within the broader context of the field.

In view of these shortcomings, the manuscript in its current form is not suitable for publication in Nature Communications.

Reviewer #3

(Remarks to the Author)
This paper is difficult to follow. The title discusses direct air capture, but the majority of the work concerns higher concentrations of CO2. Additionally, many different samples are discussed by their composition and synthesis are not described in all cases. This makes the reader have a very difficult time understanding the paper, and as a reader, I have doubts about the work that cannot be easily reconciled due to missing information. It appears that work that could easily fill 1-2 full papers was crammed into a short manuscript length for a specific journal. This is unwise, and I cannot recommend publication.

Specific comments:
1. The authors make many important claims. A new mechanism for CO2 capture. Unprecedented CO2 uptakes. Important impacts on DAC. However, there is no experimental evidence for the claimed mechanism that the readers will believe (in situ IR or NMR spectroscopy). Also, much of the sorption studies are done with 100%, 15% or 1% CO2, not 0.04% CO2 for DAC. I am not convinced by much of the presented work and discussion.

2. Given the low density, the sorbents here will have terrible volumetric productivity. Both gravimetric (you pay per kg sorbent) and volumetric (reactor and process size determined by volumetric productivity) productivity are important. The authors should compare the size of the contactor needed to obtain a fixed CO2 sorption amount with other state-of-the art sorbents.

3. Why are there references in the abstract? No journal does this?

4. The authors discuss poor conversion of renewable electricity to heat. But nobody would do that?! People would use other sources of heat, like geothermal or nuclear, with a very low or no carbon footprint. This appears to be a false motivation.

5. Authors report only one reference for ESA and DAC (ref 1). There are others – a better literature search should be conducted: https://doi.org/10.1016/j.joule.2023.05.016 This might be ref 3.

6. Page 2, lines 67-68, 0.4 to 0.65 is NOT a wide range of ratios, despite what is claimed.

7. It is claimed that XPS proves covalent attachment of amines to dGA. I do not understand this and it is not explained. The authors should be aware that technical grade TEPA (penta) has amine macrocycles – see https://papers.ssrn.com/sol3/papers.cfm?abstract_id=3814668

8. NdGA is not defined in the paper but is extensively discussed. How can a reader assess the paper is key samples are not described in the paper or the SI?

9. Fig 2, many cells in this figure use different gas compositions but that is not apparent from the caption. This makes the paper misleading and very hard to interpret.

10. p5, amines to not "attract" CO_2. Rather, Co2 has random collisions with different sites, and when it encounters an amine the amine can nucleophilicly attack the CO_2.

11. Figure 4, very large currents are required to desorb CO_2, much higher than the Joule reference above (ref 3).

12. There is little or no discussion of water. How much water is adsorbed by these materials? What is the energy penalty for water adsorption and desorption.

13. There are many steps to the synthesis of the sorbent. For DAC, we will need millions of tons of sorbents. Can this sorbent be synthesized at scale at low cost?

14. How are TGA experiments used to assess amine loading in dGA materials?

15. Reproducibility of the Dawson method of measuring CO_2 uptake should be presented.

16. Does the use of water bubblers change the CO_2 concentration in the gas phase?

17. What is dual-amine, tetra-amine?

18. There is no useful discussion of kinetic analysis.

19. The diameter of GO-aerogel shown in Fig. 2 is 5 mm. In this case, what is the penetration depth of Ar plasma into GO structure? What is the defect concentration distribution along the radial direction?

20. Introducing a defect into a graphene structure will increase the resistance of the graphene composite. What were the resistances of GO gel before and after defect/amine functionalization? How does the author rationalize the conductivity of the defective graphene? (see comment 11)

21. Lines 142-3: How did you measure porosity? What kind of porosity does it mean? Mesopore porosity or Macropore porosity?

22. If amines form layers in between graphene layers, then most of the defects will not interact with amines. What is the other evidence for one-step Zwitterion formation by defect-proton transfer to amine?

23. Is there any evidence supporting that the transient Raman peak at 2348 cm^{-1} indicates defect-proton interaction such as calculation from the first principles? Could it be just CO_2-amine interaction or by-products from the CO_2-amine reactions?

24. Overall, experimental information and support for ESA are very, very weak. It also seems like the sorbent for the high-flux system, and the ESA module look different. Why is that?

25. In the ESA part, the applied voltage is another important parameter, but the information cannot be found in the manuscript.

26. Supporting Figure 44 is too small to examine. Also, it seems like the author used a pile of rectangular-shaped sorbents for ESA. With this set up, ohmic contact can be poor and increase the resistance to very high values. The authors need to show the resistance of the single ESA sorbent and a pile of ESA sorbents along with the temperature profile of the whole ESA module during desorption.

27. With the rectangular shape sorbent, the gas flow rate of the outlet can be much lower than that of the inlet due to the significant pressure drop. The author needs to show the flow rates of the inlet and outlet.

Typos:

A. p1, line 36, "low" CO_2 concentration.

Version 1:

Reviewer comments:

Reviewer #4

(Remarks to the Author)
I was asked to assess the authors' response to Reviewer 1, who was unavailable at the time. Accordingly, my evaluation is based solely on the rebuttal document and the revised manuscript, and does not include an assessment of novelty or overall suitability for publication in Nature Communications.

Overall, the authors have addressed most of Reviewer 1's concerns. They have undertaken substantial additional work and provided a useful set of new data. However, several of Reviewer 1's questions (notably Q1, Q6, and Q9) remain insufficiently addressed and would benefit from further clarification.

Q1 and Q9:
The nature of the defect sites and the interactions between amine groups, CO_2, and defects -which are central to the proposed proton trap mechanism - remain insufficiently clear and are not strongly supported by experimental evidence. At present, the only experimental indication of defect structure appears to be a single AC TEM image, while evidence for proton transfer relies on noisy and largely inconclusive Raman data around 2900 cm^{-1} (Figure 2e, right panel). Additional clarification and experimental support would substantially strengthen the manuscript. While the challenges associated with solid state NMR are understandably outlined, some experimental evidence beyond simulations and the limited experimental data currently presented would be desirable.

Q2: The added details describing the experimental functionalisation procedures are generally adequate but remain somewhat sparse and could be further expanded to improve clarity and reproducibility. For example, it is unclear whether the plasma treated samples needed to be functionalised immediately or whether they could be stored (e.g., under a protective atmosphere) prior to amine functionalisation. In addition, the rationale for selecting the functionalisation conditions should be clarified, particularly given their implications for the TEA: a 10 hour treatment at 90 °C represents a relatively long and potentially energy intensive process.

Q3 and Q7: I am not an expert in techno economic analysis or Qst calculations. Nevertheless, the work appears to be reasonably executed and appropriately integrated into the manuscript. It would, however, be important to more clearly specify how the TEA parameters were sourced and, in particular, to explicitly state the assumptions underlying the estimated costs of the graphene aerogel and the amine functionalisation process.

Q4: The inclusion of volumetric capacity data is an important improvement, as is the comparison with competing materials. The reported volumetric capacity is relatively low and may limit commercial feasibility; however, I do not consider this a barrier to publication in Nature Communications. For transparency, the volumetric capacity value should also be explicitly stated in the main text.

Q5: The recycling experiments are a valuable addition and demonstrate reasonably good chemical stability. While many more cycles would be required in a practical application, the 100 cycles presented here appear appropriate in the context of a primarily fundamental scientific investigation.

Q6: In my view, the authors have partially misunderstood this comment. Their response focuses on reaffirming the novelty of the proton trap process, whereas the original comment concerned the broader applicability of electrically swing adsorption (ESA) to defective material systems, including those with limited conductivity (e.g., defective MOFs combined with carbon to induce electrical heatability). That said, this is a relatively minor point intended to broaden the implications of the work and is not critical for publication in my view.

Q8: This point has been fully addressed. Revising the main text to present data exclusively under ambient conditions (400 ppm CO_2) is a significant and necessary improvement and avoids potential misunderstandings, as previously noted by another reviewer.

Reviewer #5

(Remarks to the Author)
The comments given to authors are well addressed.

Reviewer #6

(Remarks to the Author)
The revised manuscript has been substantially improved compared to the previous version. The overall structure is now significantly clearer, allowing the central concept and mechanistic insights to be more readily appreciated. The newly incorporated operando/in situ characterizations and the quantitative assessment of Joule-heating-driven regeneration further strengthen the key scientific claims of the work. The manuscript is suitable for publication after the following concerns are clarified.

(1) From lines 174–177, the authors report that the kinetic rate constant decreases in the presence of water, indicating slower adsorption kinetics. In contrast, the calculations in lines 198–204 suggest that water enhances CO2 adsorption via hydrogen bonding. These observations appear to be inconsistent. The authors are encouraged to provide a more detailed discussion to explain the apparent discrepancy between kinetic and thermodynamic effects of water.

(2) Figure 4c shows a slight but noticeable decline in CO2 uptake over cycling. The authors are encouraged to clarify the underlying cause.

(3) The authors refer to a 95% CO2 release in both the experimental demonstration (line 192) and the techno-economic analysis (line 295). Do these two values have the same meaning, that 95% of the sorbent was regenerated? If regeneration is incomplete, does the remaining fraction of adsorbed CO2 accumulate over cycles, potentially leading to performance degradation? Further clarification on the definition and implications of this release ratio would be helpful.

(4) In response to Question 11, the authors state that the regeneration energy and temperature are lower than those reported in prior studies. However, a key claim of this work is the enhanced CO_2 binding under dilute conditions. From a thermodynamic perspective, stronger binding is typically associated with higher desorption energy barriers. Therefore, it is not clear how both enhanced capture and facile regeneration are simultaneously achieved.

Version 2:

Reviewer comments:

Reviewer #4

(Remarks to the Author)
The authors have fully addressed all my queries. I have no further comments.

Reviewer #6

(Remarks to the Author)
The authors have satisfactorily clarified the concerns raised in my previous review. I appreciate the additional explanation and revisions provided in the manuscript. In my opinion, the manuscript is now suitable for publication in Nature Communications.

Open Access This Peer Review File is licensed under a Creative Commons Attribution 4.0 International License, which permits use, sharing, adaptation, distribution and reproduction in any medium or format, as long as you give appropriate credit to the original author(s) and the source, provide a link to the Creative Commons license, and indicate if changes were made.
In cases where reviewers are anonymous, credit should be given to 'Anonymous Referee' and the source.
The images or other third party material in this Peer Review File are included in the article’s Creative Commons license,

unless indicated otherwise in a credit line to the material. If material is not included in the article's Creative Commons license and your intended use is not permitted by statutory regulation or exceeds the permitted use, you will need to obtain permission directly from the copyright holder.
To view a copy of this license, visit https://creativecommons.org/licenses/by/4.0/

Point-by-Point Response to Referees

Reviewer #1:

General comment: An innovative amine-functionalized graphene aerogel (N-dGA) with point defect engineering achieves a record CO_2 uptake of 6.57 mmol g^{-1} at 400 ppm CO_2 and 1.0 m s^{-1} gas velocity, surpassing traditional DAC sorbents, and proposing a low-cost electro-thermal desorption technology below conventional DAC energy consumption levels. Point defects in N-dGA enable efficient dispersion of amines and serve as proton acceptors, could revolutionize ESA materials for DAC by facilitating simultaneous zwitterion formation and proton transfer within a single elementary reaction, enhancing CO_2 adsorption capacity, stability, and electrical desorption rates. As a whole this is a very nice proof of concept.

Response: Thank you so much for your positive comments on our work.

1. The authors have employed diverse experimental methodologies and computational analyses to elucidate the interaction dynamics between amines and point defects. However, the mechanism underlying the attraction of amines towards point defects remains ambiguous. Why amines are so attracted towards defects shall be better explained. The inquiry seeks to discern the specific atoms or groups involved in the interaction and subsequent bond formation.

Response: The attraction originates from synergistic electronic and structural effects at the atomic scale. Point defects in carbon frameworks (e.g., vacancies, Stone-Wales defects) induce localized disruption of sp^2 hybridization symmetry, generating unpaired electrons via partial sp^3 reconfiguration (An et al. Stone-wales defects cause high proton permeability and isotope selectivity of single-layer graphene, Adv. Mater. 2020, 32, 2002442). These defect sites exhibit heightened chemical reactivity and a more positive electrostatic potential relative to pristine graphene, which collectively promote the adsorption and covalent binding of amine molecules on them.

Based on the computational data presented in **Figures** S30-S37 in SI, we evaluated

the Gibbs free energy changes during amine molecules adsorb or bind to *d*GA surfaces with and without point defects. Conformational analyses reveal that the presence of point defects thermodynamically stabilizes amine adsorption, reducing the system potential energy by 0.16-0.27 eV. Furthermore, during functionalization, amines selectively react with reactive moieties adjacent to defects form C-N bonds, corresponding to a Gibbs free energy reduction of −0.77 to −2.30 eV. The emergence of graphitic nitrogen species at ~400.7 eV in high-resolution XPS N1s spectra (**Figure** S27) provides direct experimental validation of covalent bond formation between amines and defect sites.(Wood et. al, Spectroscopic investigation of nitrogen-functionalized carbon materials, Surf. Interface Anal., 2016, 48, 283-292)

The relevant content has been updated on page 16 of the revised manuscript, and Figure S27 of the Supplementary Information.

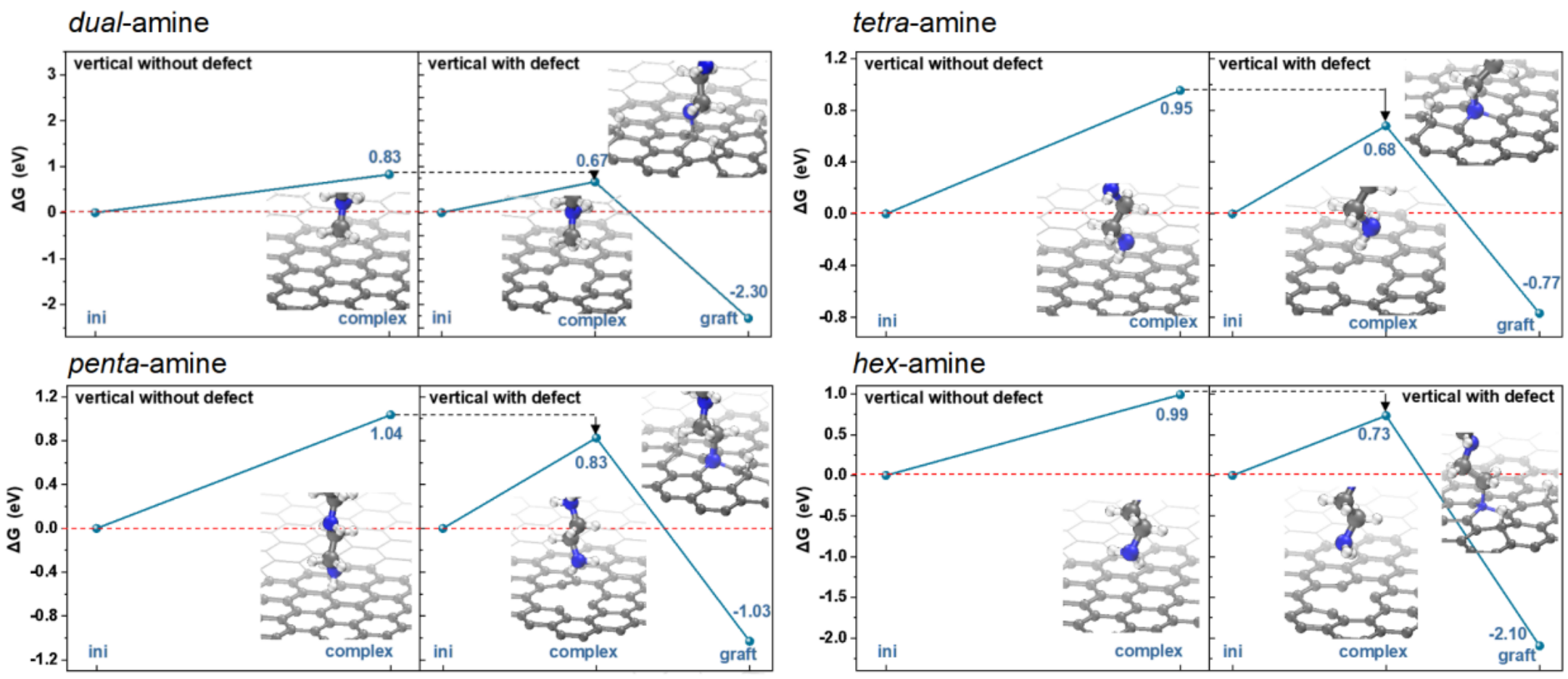

Organized from Figure S30-S37: The ΔG of amines adsorbed or bound on the *d*GA surface.

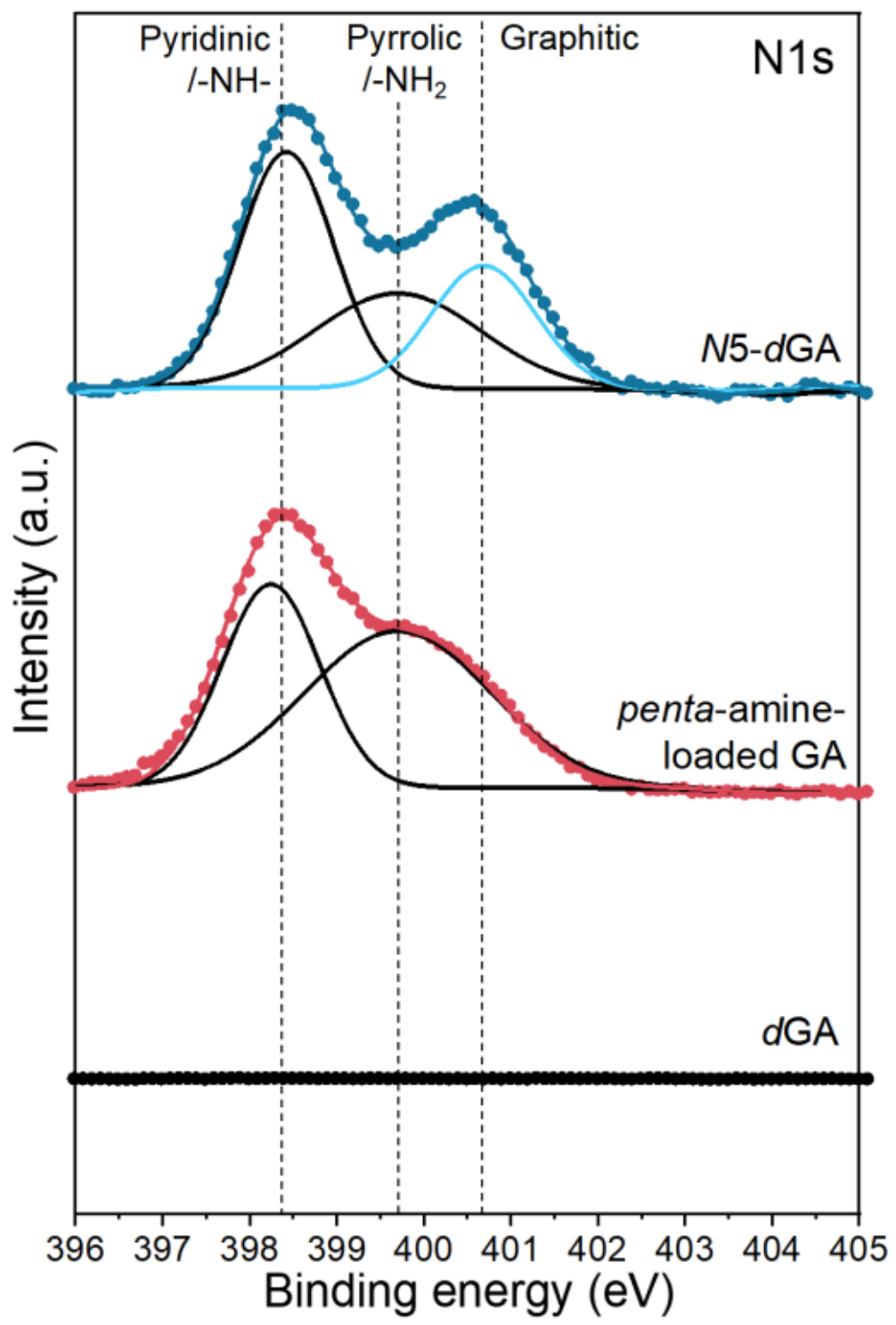

Figure S27: High-resolution XPS N1s spectra of *N5*-*d*GA, *penta*-amine-loaded GA and *d*GA.

2. The synthetic procedure lacks details. Please specify the quantity ratio of graphene aerogels to amines, the solvent and synthetic conditions, and the washing and activation methods employed for the sample.

Response: Sorry for your confusion. In our synthesis protocol, a fixed stoichiometric ratio was consistently maintained during the amine-functionalization of *N*-dGA. Amine loading was modulated exclusively by varying Ar plasma treatment duration (0–180 min) to regulate defect density (**Figure** S23). Due to the inherent chemical reactivity and positive electrostatic potential of point defects, amines were grafted with precision, achieving controlled loadings ranging from 0.3 to 87.0 wt% (**Figure** S4).

The specifically synthesis protocol can be found at page 16 in the manuscript:

"20 mg of *d*GA carrier was immersed in 25 mL of undiluted *penta*-amine (tetraethylenepentamine, technical grade, purity > 90%) within a Teflon-lined hydrothermal reactor. Amine loading was modulated exclusively by varying Ar plasma treatment duration (0–180 min) to regulate defect density. The sealed reactor was heated at 90°C for 10 h in a blast oven. Subsequently, samples were soaked in 170 mL of

anhydrous ethanol at 40°C for 20 min (without stirring), followed by overnight drying at 80°C in a blast oven."

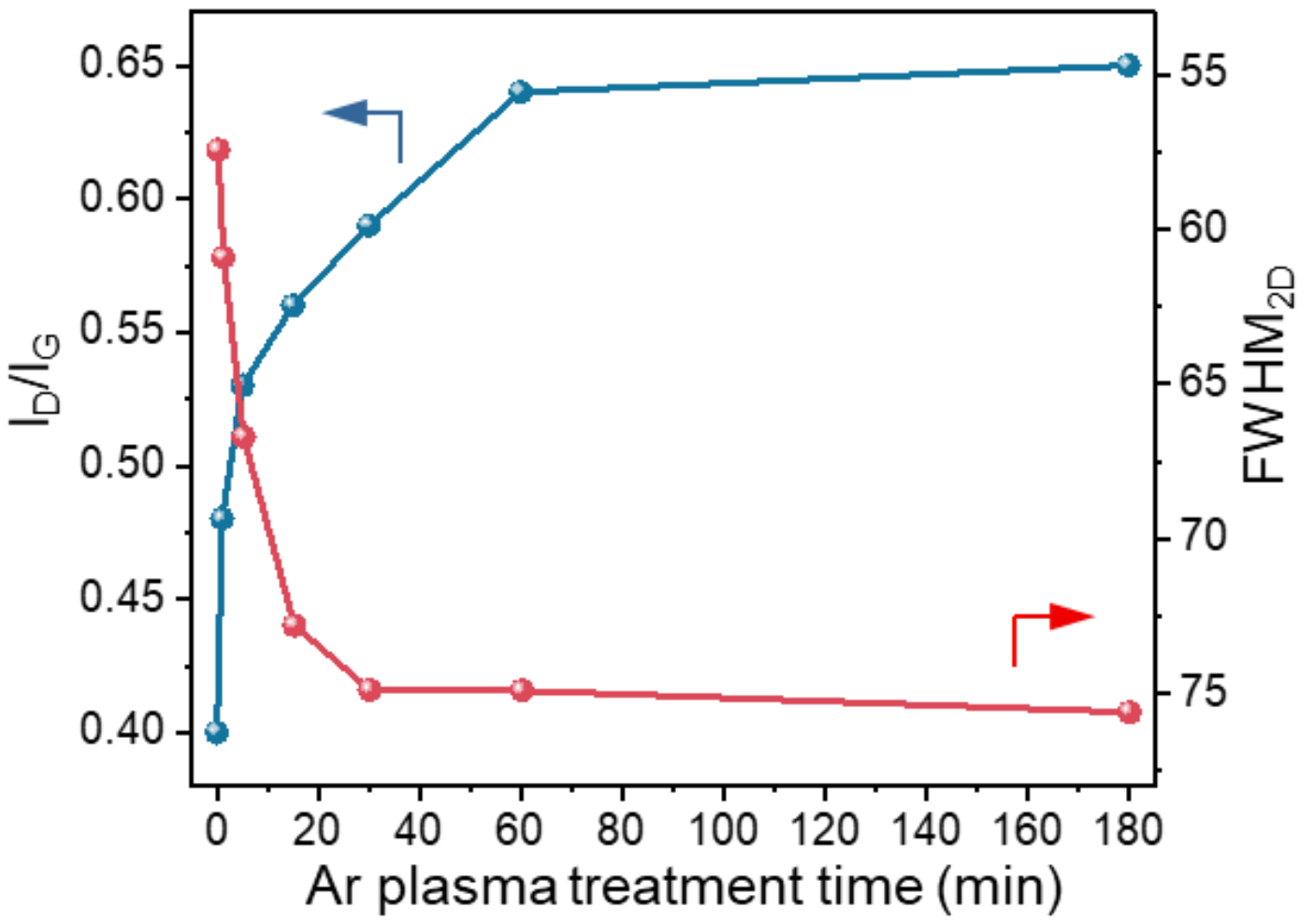

Figure S23: I_D/I_G and $FWHM_{2D}$ of *d*GA after different durations of Ar plasma treatment.

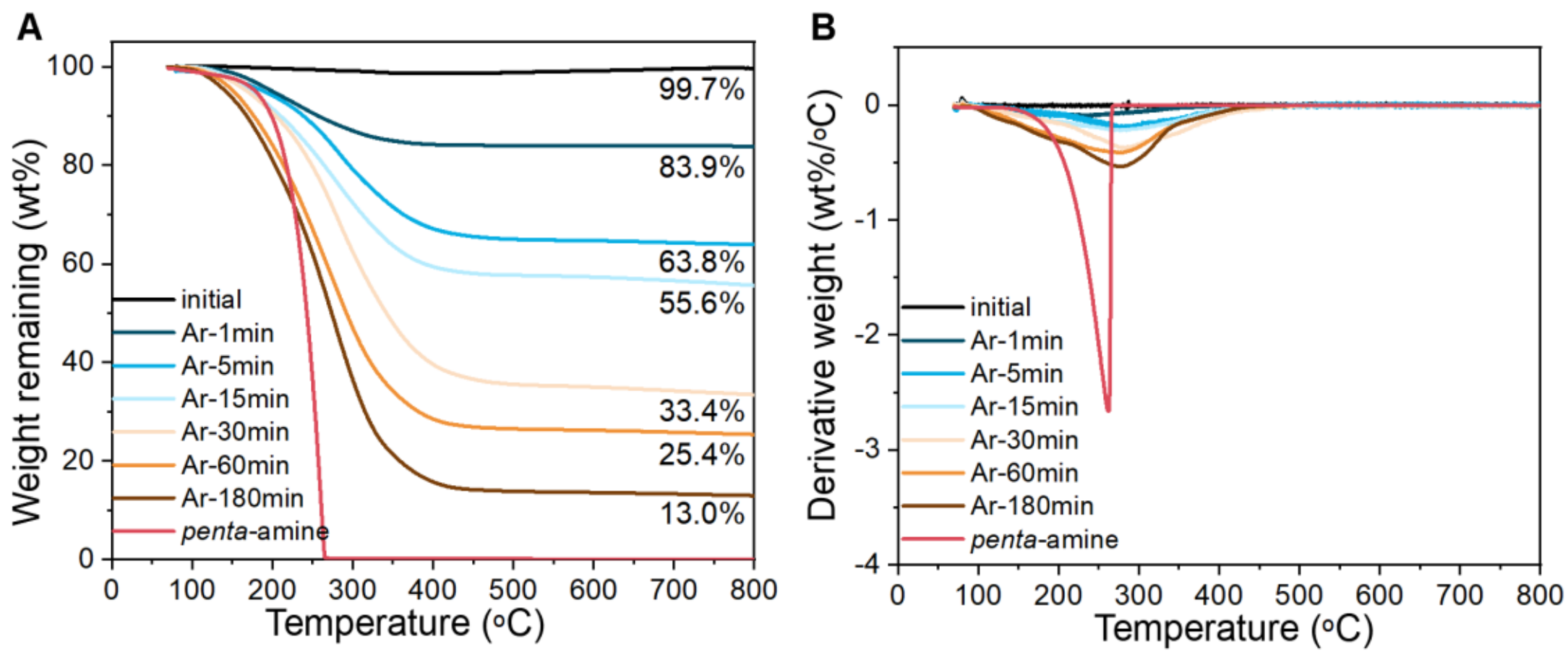

Figure S4: (A) TG curves and (B) derivative of thermal gravity curves of *N*5-*d*GA after different durations of Ar plasma treatment.

3. One shall calculate the Qst at different temperatures.

Response: We sincerely appreciate your suggestion. Indeed, the electrothermal temperature plays a critical role in determining the ESA heat duty required for sorbent regeneration. The total regeneration energy comprises desorption heats of CO_2 and water, and sensible heats of CO_2, water, sorbent and quartz tube column. In general, the isosteric heat of adsorption (Q_{st}) is a widely accepted parameter for evaluating adsorption thermodynamics using static equilibrium data at different temperature. However, for the dynamic adsorption process of DAC, the CO_2 desorption heat is

typically evaluated using the adsorption enthalpy change (ΔH_{ads}), which has been validated in recent studies. (Lee, W. H. et al. Sorbent-coated carbon fibers for direct air capture using electrically driven temperature swing adsorption. Joule 7, 1241-1259, (2023); Lee, Y. H. et al. Design of electrified fiber sorbents for direct air capture with electrically-driven temperature vacuum swing adsorption. Adv. Mater., e04542, (2025)) Detailed derivations and calculation methodologies are provided in the "Thermodynamic analysis" of Supplementary Information.

The ESA heat duty of *N*-*d*GA was quantitatively assessed across varying current densities and experimentally validated (**Figure** 4A). While elevating the current intensity from 0.6 A to 0.8 A sharply increases sensible heat, reducing it to 0.5 A paradoxically raises both sorbent sensible heat and water desorption heat. This discrepancy arises from a 50% reduction in the amount of CO_2 desorbed at 0.5 A (**Table** S2), necessitating a larger sorbent mass to maintain equivalent CO_2 cyclic capacity. Notably, the minimum ESA heat duty of 3.4 $GJ\cdot t^{-1}$ CO_2 occurs at 0.6 A, where experimental and calculated values show excellent agreement.

The relevant content has been updated on pages 9-10 and **Figure** 4 of the revised manuscript, as well as **Table** S2 of the Supplementary Information.

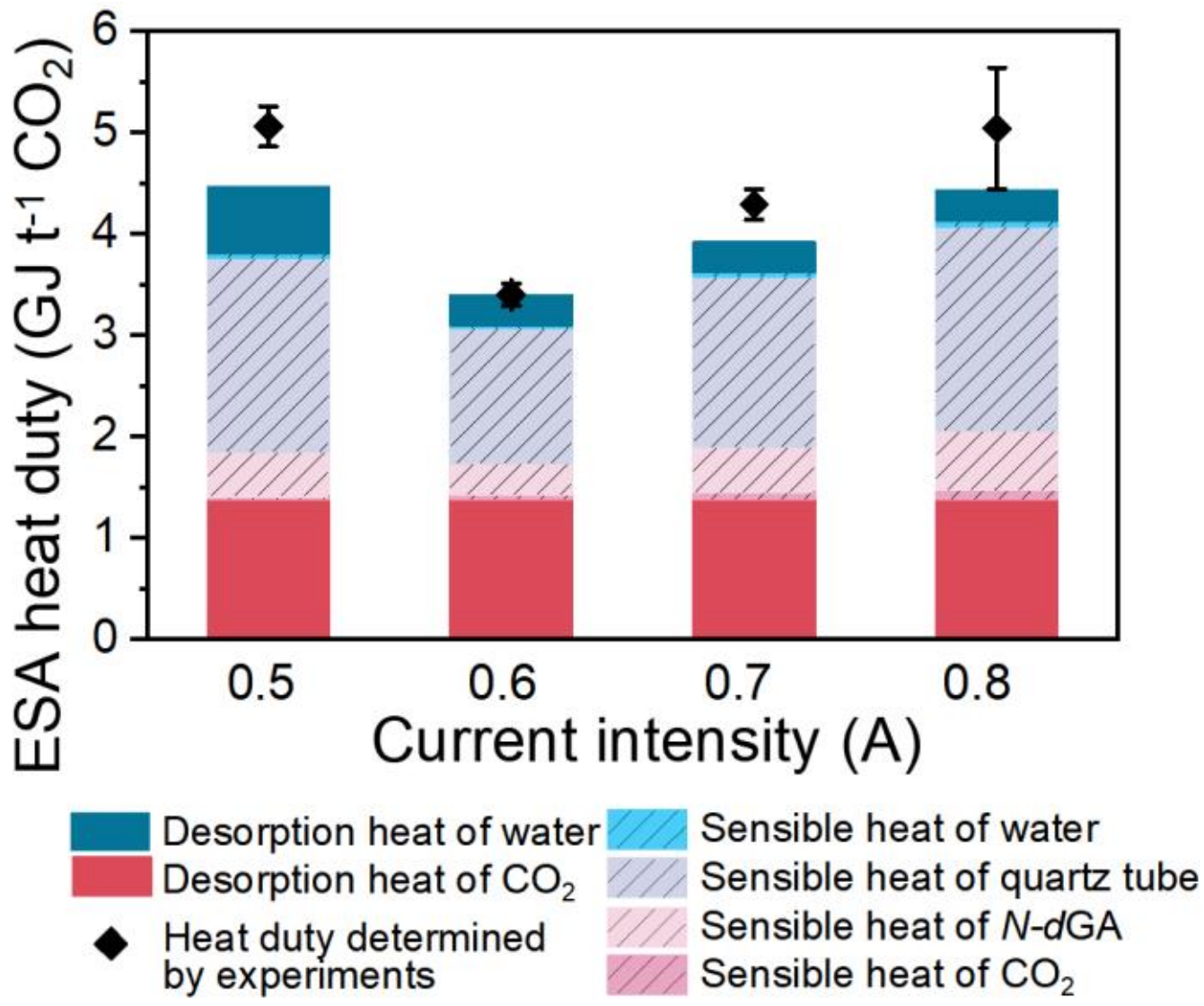

Figure 4A: Comparison of calculated and experimental ESA heat duties for CO_2 desorption using *N*5-*d*GA. The *N*5-*d*GA adsorbs CO_2 to saturation in humid air.

Table S2: Parameters used for calculated ESA heat duty.

Parameters	Symbol	Value				Unit
CO_2 adsorption enthalpy changes	ΔH_{ads}	60.7				kJ mol^{-1}
Water adsorption enthalpy changes	ΔH_{H2O}	42				kJ mol^{-1}
Mass ratio of adsorbed water to CO_2	$r_{H2O/CO2}$	0.13				-
Mass ratio of column to sample	$r_{column/sample}$	10.42				-
Heat capacity of CO_2	$C_{p,CO2}$	0.85				kJ kg^{-1} K^{-1}
Heat capacity of H_2O	$C_{p,H2O}$	4.18				kJ kg^{-1} K^{-1}
Heat capacity of graphene	$C_{p,graphene}$	0.75				kJ kg^{-1} K^{-1}
Heat capacity of *penta*-amine	$C_{p,amine}$	2.0				kJ kg^{-1} K^{-1}
Heat capacity of quartz	$C_{p,quartz}$	0.8				kJ kg^{-1} K^{-1}
Current intensity of electrothermal desorption		**0.5**	**0.6**	**0.7**	**0.8**	A
Electrothermal temperature		60	80	105	129	°C
The amount of CO_2 desorbed		45	95	95	95	wt%
Temperature rise of sample	ΔT_{sample}	35	55	80	104	K
Temperature rise of column	ΔT_{column}	28.7	45	56.7	68	K

4. The record value mentioned by the authors is shown in mmol g^{-1}. However, in most cases performances are given in volume/volume values; here the graphene-based material has probably a very low bulk / packing density which would lead to a much lower v/v performance in comparison with denser materials such as porous silica / MOFs amine grafted materials. Thus, the referee recommends strongly that authors discuss this point and give an approximative v/v value and compare this value with the other benchmarks in this field. Accordingly a table such as the one figure 2d.

Response: Thank you for your insightful suggestion. Unlike gravimetric capacity (mmol/g), the volumetric capacity is governed by sorbent bulk density and holds direct engineering relevance. As requested, we have estimated the approximate volumetric capacity of *N*5-*d*GA based on its gravimetric capacity and measured bulk density, and compared it with state-of-the art sorbents under related conditions. As shown below, the ultrahigh porosity of the graphene aerogel (>98%) results in a reduced CO_2 capacity per unit volume for *N*5-*d*GA. However, the high CO_2 uptake (6.73 mmol/g), exceptional

cyclic stability (>100 cycles with <5% decay), mechanical robustness, and low heat duty (3.4 GJ t^{-1} CO_2) present a compelling case for practical DAC deployment of *N*5-*d*GA.

The relevant content has been updated in **Table** S1 of the Supplementary Information.

**Name**	**CO_2 uptake ($mmol \cdot g^{-1}$)**	**Bulk density ($mg \cdot cm^{-3}$)**	**Volumetric capacity ($mol \cdot m^{-3}$)**
*N*5-dGA (this work)	6.57	35	52.6
MIL-101(Cr)@TEPA	0.95	430	408.5
Lewatit® VP OC 1065	1.16	670	777.2
MOR-type zeolites	1.05	950	997.5

5. Amine based materials suffer often from strong stability / cyclability issues. Here the ESA process is used to desorb the CO2 from the amine grafted aerogel; have authors look at the longer term stability upon cycling of their material ? Here, if the authors have carried out 1,000 decompression cycles, the separation of CO2 over N2 has only been carried out for three cycles (without ESA) and the referee does not understand if multiple ESA cycling has been carried out. Note that any real CCS process needs a material that can still be efficient upon >100,000 cycles…This is here particularly important as overheating through ESA could lead to hot spots that, in the presence of oxygen, could degrade the grafted amines.

Response: We agree with your rigorous perspective regarding process scalability. In response, we evaluated the sorbent's stability over 100 consecutive ESA cycles, observing only a 3.7% decline in CO_2 uptake capacity after 100 cycles (from 6.57 to 6.33 mmol g^{-1} in **Figure** 4C). Post-cycling characterization further supports the material's robustness. TGA analysis shows negligible change in amine loading, while XPS N1s spectra and Raman spectroscopy confirm that the covalent grafting between amine groups and *d*GA remains intact, indicating no significant degradation during repeated electrothermal regeneration (**Figures** S15-S17). Additionally, **Figure** S12

shows the distance-dependent temperature profile along the bulk *N*5-*d*GA surface under constant electrical power at varying current densities, demonstrating spatially uniform heating without detectable hot spots.

The relevant content has been updated in page 10 of the revised manuscript and **Figures** S15-17 of the Supplementary Information.

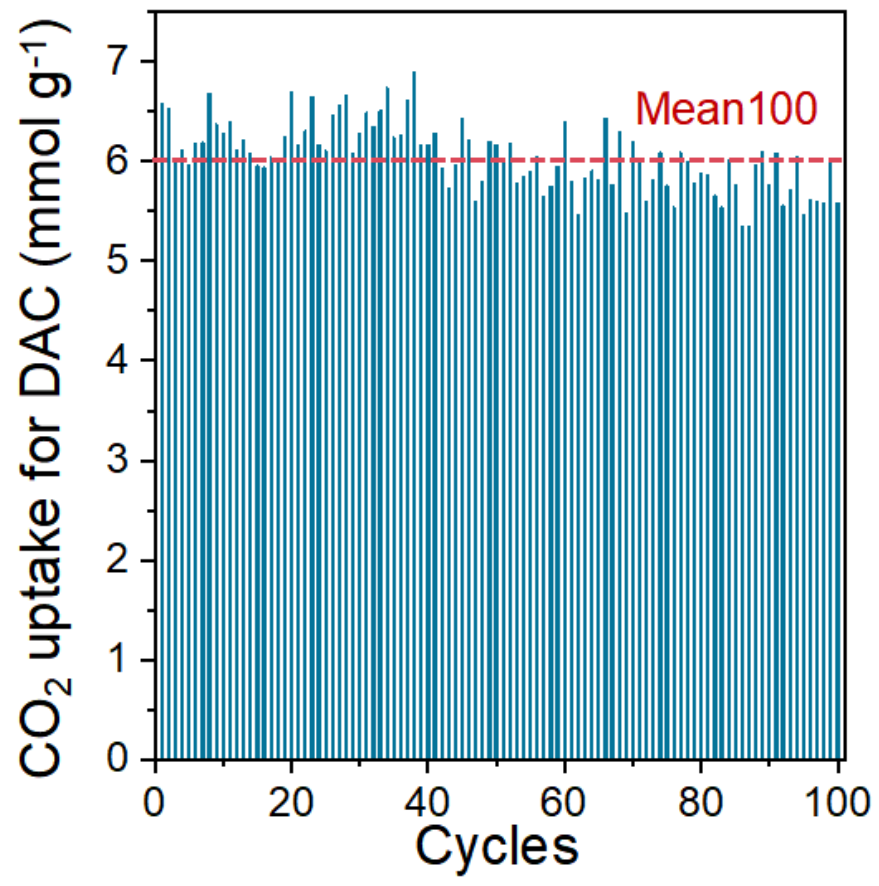

Figure 4C. Bar graph of 100 cycles of CO_2 adsorption–electrothermal desorption. The horizontal red dashed lines represent the average CO_2 uptake over the 100th cycle.

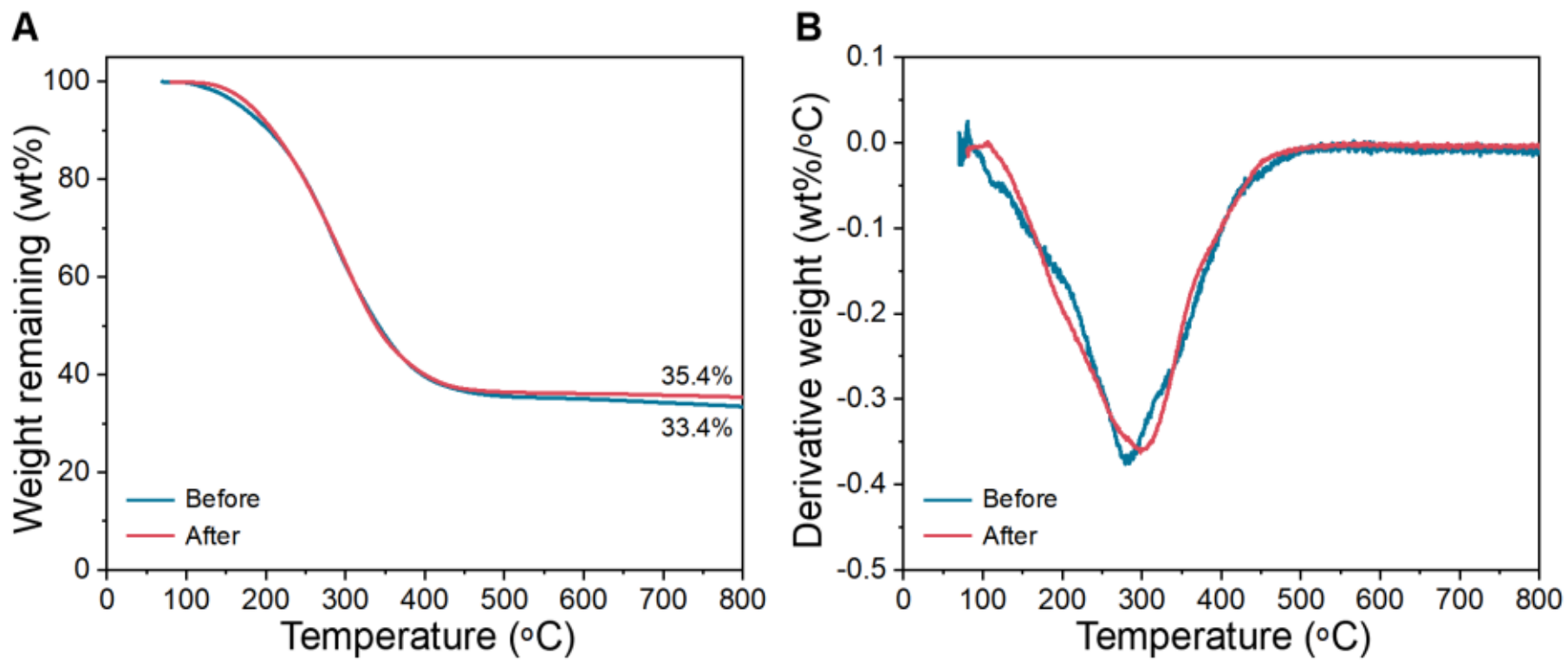

Figure S15: (A) TG curves and (B) derivative of thermal gravity curves of *N5-d*GA before and after 100 adsorption-desorption cycles.

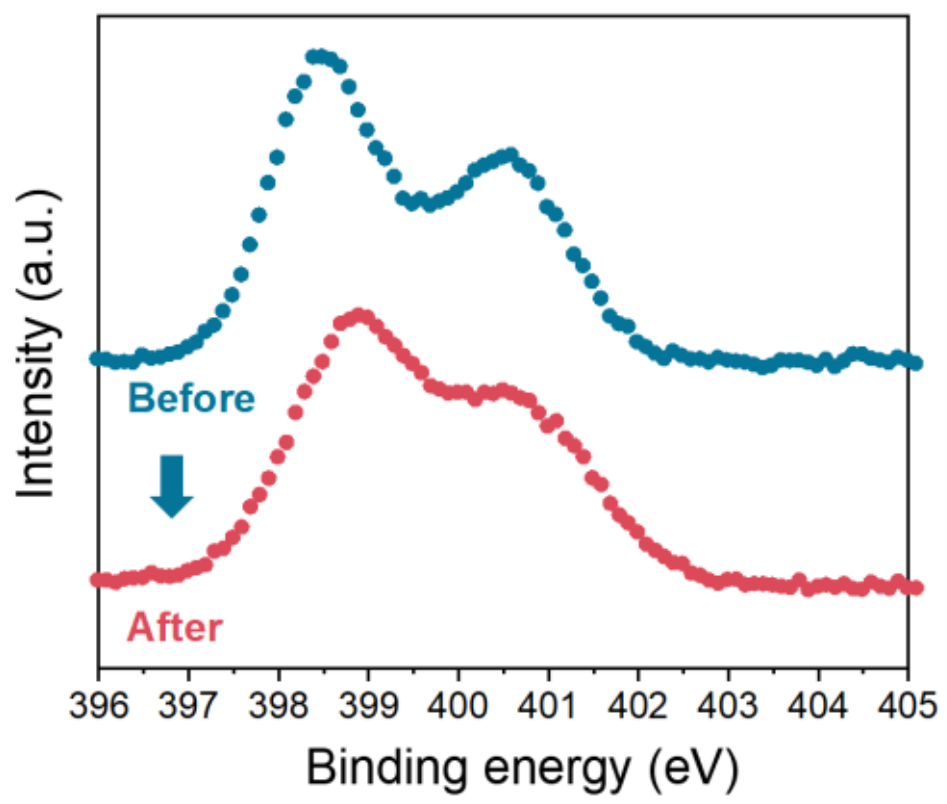

Figure S16: High-resolution XPS N1s spectra of *N5-d*GA before and after 100 adsorption-desorption cycles.

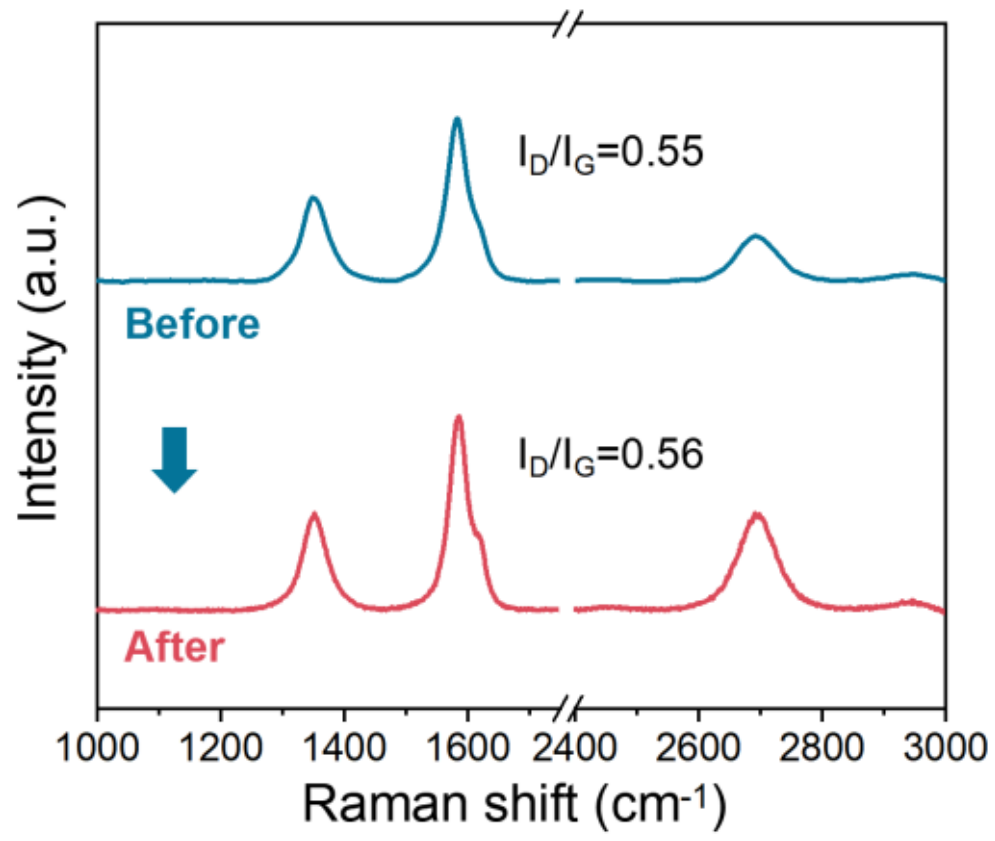

Figure S17: Raman spectroscopy of *N5-d*GA before and after 100 adsorption- desorption cycles.

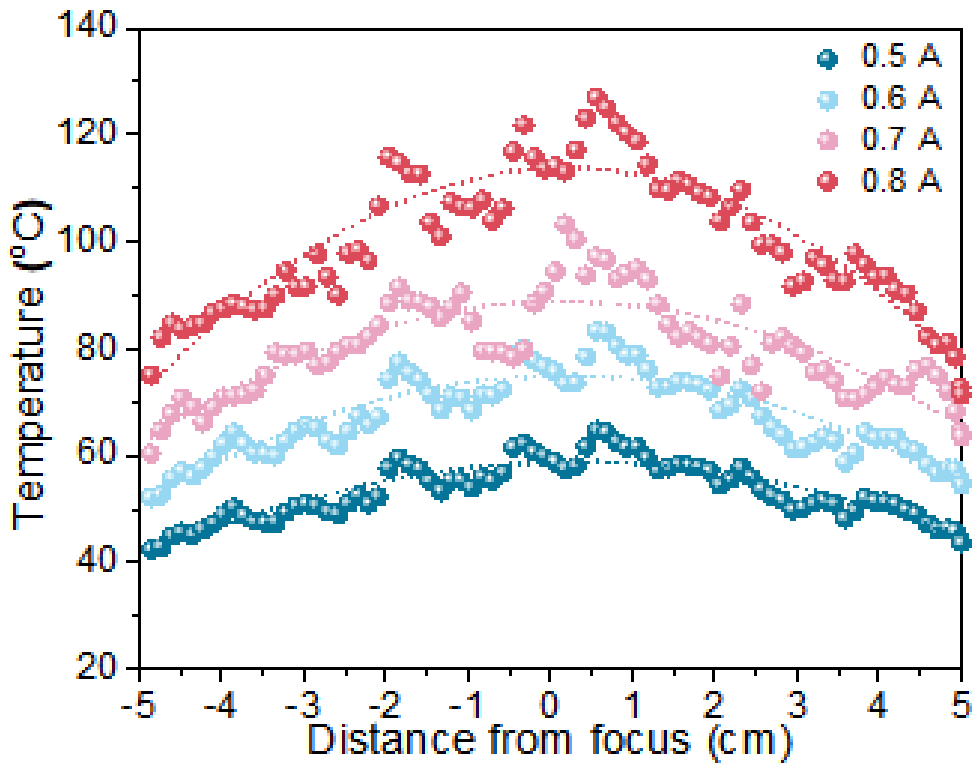

Figure S12: Distance-dependence temperature of focus point on bulk *N5-d*GA surface at different current intensities.

6. ESA processes are not limited to bare porous carboneous materials, for instance when dealing with insulating porous materials that are hybridised with electronically conductive carbon additives. In addition defective porous graphene oxides are not expected to exhibit a high electronic conductivity; thus one could expect the inorganic or hybrid carbon composites to exhibit a good conductivity suitable for ESA as shown before for Microwave swing adsorption. This point shall be discussed, probably in the outlooks, to determine if the new mechanism in play either is superior to other non carbon state-of-the-art ESA materials or could even be applied to other types of defective inorganic or hybrid adsorbents (of course being set conductive enough with carbon additives).

Response: Sorry for your confusion. The proton-trap mechanism is applicable only when the CO_2 capture mechanism inherently involves proton transfer, which was a requirement specific to amine-based systems. The conventional amine-based CO_2 mechanism requires a 2:1 amine:CO_2 stoichiometry (one amine forms a carbamate or zwitterion, and another accepts a proton). Our proton-trap mechanism breaks this limitation by introducing point defects as proton acceptors, enabling 1:1 stoichiometry. Critically, this mechanism only applies to amine-functionalized sorbents. Non-carbon materials (e.g., MOFs, zeolites) capture CO_2 via physical/chemical interactions with surface sites without proton transfer, making proton traps unnecessary for their established mechanisms.

7. The plasma generation of defects is a rather expensive and poorly scalable technique. As DAC is a very large-scale process, it this amine grafted defective GO suitable for real applications (note that GO itself is not a cheap material, although being already commercialised). Authors shall discuss the cost and scalability of their materials.

Response: Scalability and cost are critical considerations that cannot be overlooked for any emerging technology. While plasma processing has historically been regarded as a batch-limited technique, continuous-flow argon plasma treatment and the scale-up of plasma reactors have now been demonstrated as feasible (Mohotti, D., Hossain, M. M., Mukhtar, A. & Wu, S. Impact of discharge gas on a novel continuous flow in-liquid plasma discharge process for cobalt remediation and recovery from wastewater, Journal of Environmental Management, 384, 125570, (2025); Assadi, A. A., Bouzaza, A. & Wolbert, D. Comparative study between laboratory and large pilot scales for VOC's removal from gas streams in continuous flow surface discharge plasma, Chemical Engineering Research and Design, 106, 308-314 (2016)). More importantly, our techno-economic analysis shows that the cost of synthesis and post-processing (including plasma treatment, ethanol washing, and drying) accounts for only 5.3% of the total sorbent cost (**Table** S8, SI). Furthermore, at a deployment scale of 4000 t CO_2 per year, the material cost of *N*5-*d*GA in the ESA-DAC process is just 12% higher than that of the commercial sorbents Lewatit® VP OC 1065 used in conventional TSA-DAC.

As shown in **Figure** 5, when powered by renewable electricity, ESA-DAC achieves up to 78% lower operating costs compared to TSA-DAC and delivers a capture cost well below the \$100 t^{-1} CO_2 target, establishing a clear pathway to gigaton-scale deployment.

We have added a new section "Pathway to economic viability" to discuss scalability considerations and cost trade-offs in the revised manuscript. The relevant content has been found at pages 11-12 and **Figure** 5 of the revised manuscript, and **Table** S4-S8 of the Supplementary Information.

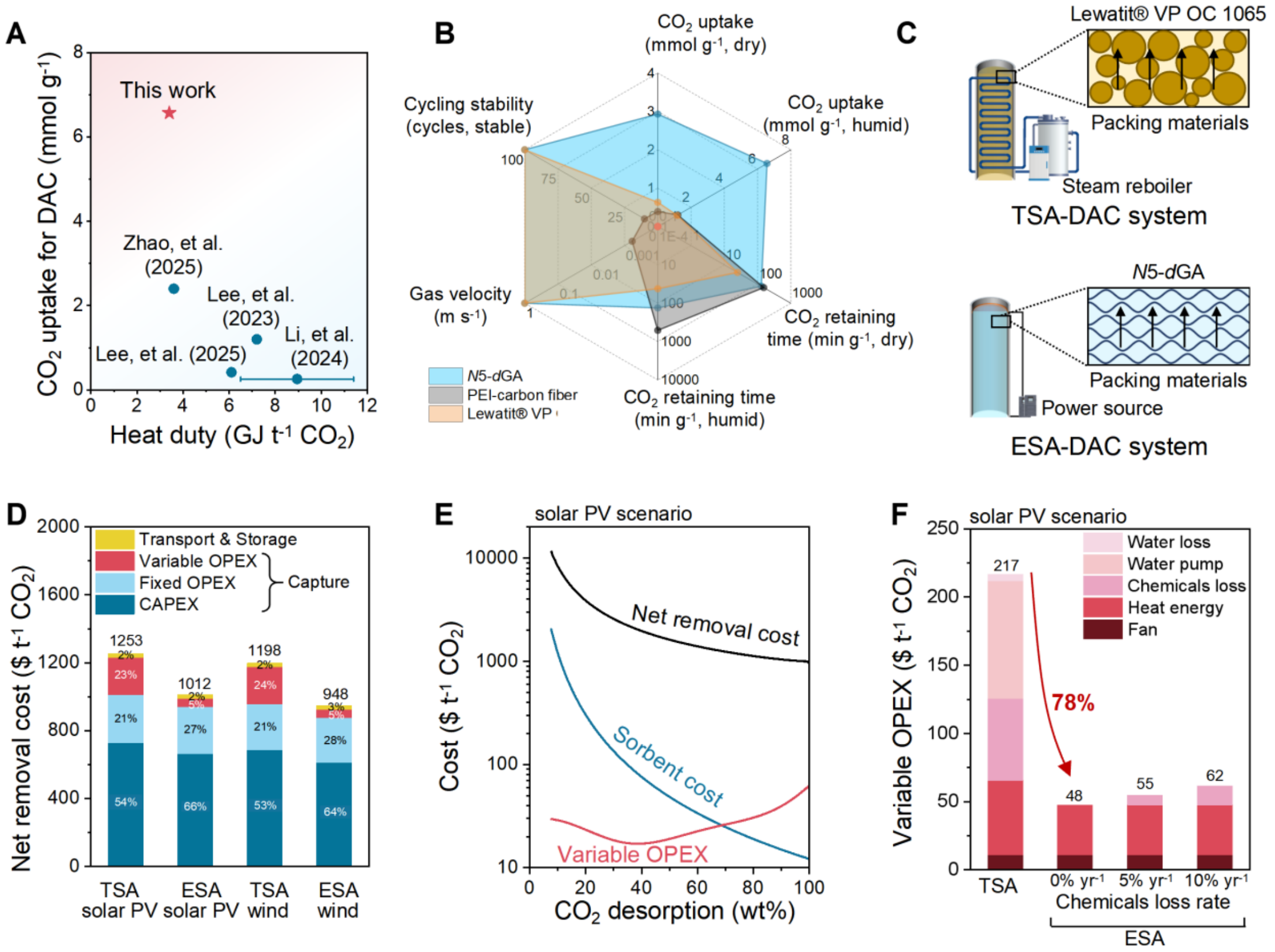

Figure 5. Techno-economic analysis of ESA-DAC system.

(A) A comparison of CO_2 uptake versus regeneration energy for *N*5-*d*GA and reported Joule heating-enabled sorbents, all DAC tested under ambient conditions (400 ppm CO_2, 25 °C, 1 atm). (B) The CO_2 uptake, CO_2 retaining time, cycling stability and gas velocity of different sorbents at 25 °C. (C) The schematic diagram of TSA- and ESA-DAC systems. (D) Cost for TSA- and ESA-DAC systems categorized by their electricity sources of a 4000 t a^{-1} scale. The stacked bars represent the CO_2 net removal cost. The amount of CO_2 desorbed is 95 wt%. (E) Relationship between DAC cost by solar PV and amount of CO_2 desorbed in the ESA-DAC system. (F) Detailed variable OPEX for CO_2 capture using TSA- and ESA-DAC systems by solar PV of a 4000 t a^{-1} scale. The amount of CO_2 desorbed is 95 wt%. The data of TSA-DAC system was reported by Sievert et al. (Sievert, K., Schmidt, T. S. & Steffen, B. Considering technology characteristics to project future costs of direct air capture. Joule 8, 979－999, (2024))

Table S8: Process specific parameters of ESA-DAC.

Parameters	Value	Unit
Initial plant capacity	4000	t CO_2/a
adsorption capacity	6.57	mmol/g
adsorption time	6.00	h
desorption time	2.00	h
cooling time	4.00	h
Capacity factor	0.98	
Regenerative energy consumption	3.40	GJ/tCO_2
Cost of graphene aerogel	0.16	M$/t
Cost of *penta*-amine	0.004148	M$/t
Cost of Synthesis and post-processing (e.g. plasma, washing, drying)	0.003	M$/t
Regeneration temperature	80.00	℃
Specific gravity of *penta*-amine	66.7	%
Graphene aeroge specific gravity	33.3	%
Initial sorbent	9.48	t
Initial sorbent costs	0.56	M$
Capacity factor	95	%
Electricity cost of solar PV	39	$//MWh
Electricity cost of wind	37	$//MWh
Carbon intensity (x)	0.0176	
Additional electrical energy (like air blower, etc).	0.99	GJ/t CO_2

8. The authors presented the Isotherm Absorption capacity versus time at 1 bar. Please provide the isotherm illustrating absorption pressure ranging from 0 to 1 bar.

Response: Sorry for your confusion. Now all CO_2 uptake values reported in the manuscript exclusively reflect 400 ppm CO_2 breakthrough experiments. The inlet gas was simulated air containing CO_2/N_2 (0.04/99.96) at a gas flow rate of 1000 mL min^{-1} under dry (0% RH) and humid (80% RH) conditions, with an outlet flow rate of 999 mL min^{-1}. To ensure methodological clarity and alignment with the DAC focus of this work, we have removed adsorption data under other CO_2 concentrations (CO_2/N_2=15/85 and 1/99) from the main text—these data were not representative of

realistic DAC conditions and created unnecessary ambiguity.

The relevant content has been updated in the "CO_2 capture from dilute air via proton trapping" section and **Figure** 3 of the revised manuscript, as well as the "CO_2 uptake for DAC" section of the Supplementary Information.

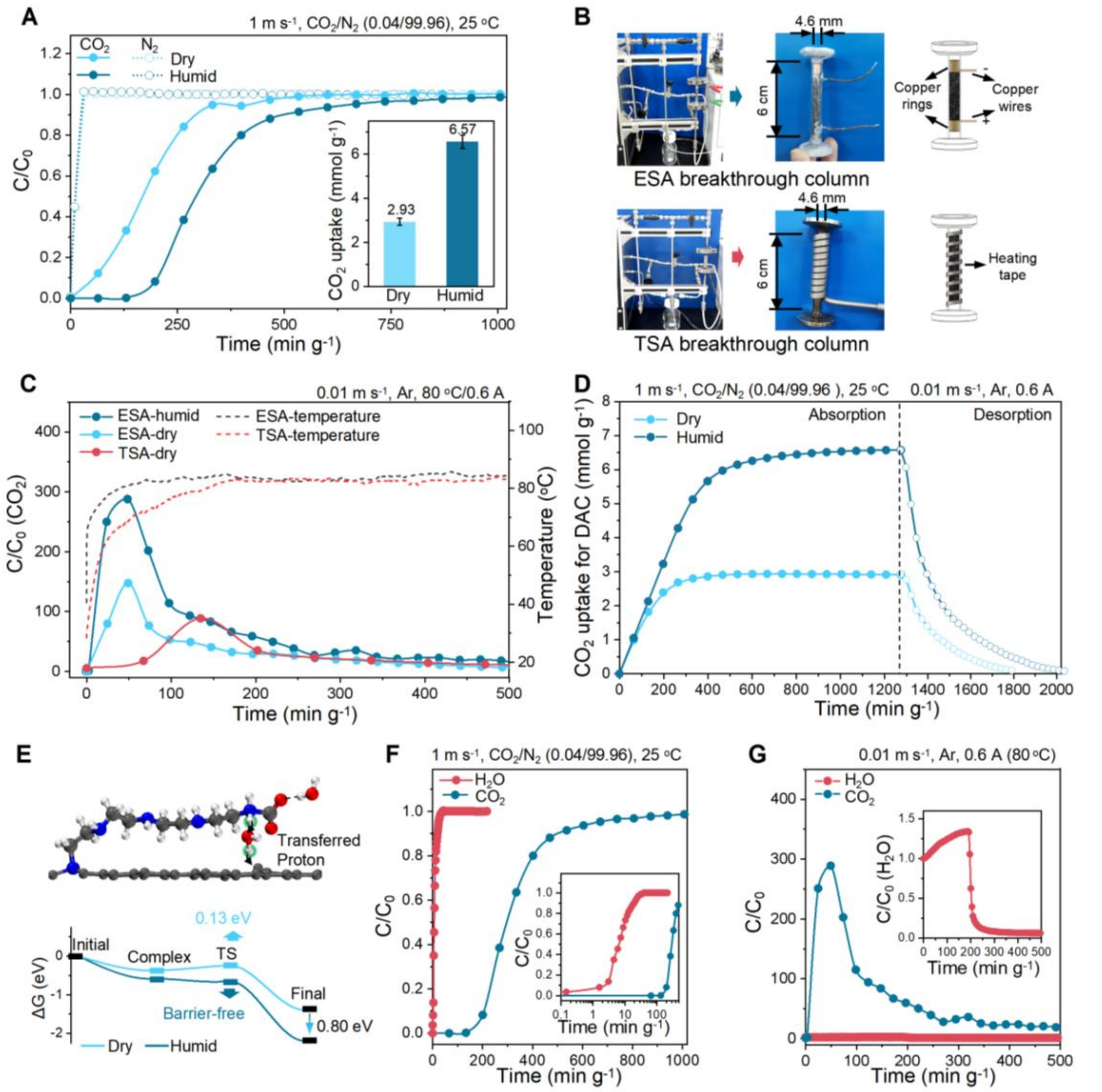

Figure 3. ESA performance of *N-d*GA for DAC.

(A) Breakthrough curves and CO_2 uptakes adsorbed directly from the air of *N*5-*d*GA under dry and humid conditions. (B) The breakthrough columns for ESA and TSA. (C) CO_2 desorption and temperature curves of *N*5-*d*GA via ESA and TSA. (D) CO_2 adsorption and desorption process of *N*5-*d*GA under dry and humid conditions. (E) Schematic diagram and the ΔG plot of *N*5-*d*GA for CO_2 adsorption under humid conditions. (F) Breakthrough adsorption and (G) electrothermal desorption curves of CO_2 and water.

9. The authors shall carry out solid-state NMR analysis of CO_2 adsorbed onto N-dGA

to elucidate the interaction between CO_2 and amine.

Response: We sincerely thanks for your suggesting. In general, the high conductivity and paramagnetism of the reduced graphene framework cause severe signal broadening (Souza, et al. NMR spectral parameters in graphene, graphite, and related materials: Ab Initio calculations and experimental results. J. Phys. Chem. C, 120, 27707-27716, (2016); Leskes, et al. Surface-sensitive nmr detection of the solid electrolyte interphase layer on reduced graphene oxide. J. Phys. Chem. Lett. 8, 1078-1085 (2017)). When combined with the low intrinsic sensitivity of ^{13}C NMR, these factors obscure the signals of adsorbed CO_2, making it difficult to resolve the specific amine interactions. We attempted to mitigate this issue by mixing it with KBr, but the results were unsatisfactory (**Figure** R1).

To robustly probe the binding mechanism, we employed alternative techniques that circumvent these limitations and provide complementary insight. DFT calculation results in **Figures** 2A and 2B revealed a 79% lower energy barrier for the proton-trap pathway compared to the conventional zwitterion mechanism. The proton traps exhibit rapid regeneration under the influence of an electric field, demonstrating a high suitability for the ESA process. **Figure** 2C shows that the presence of an electric field can effectively reduce the energy barrier of the dissociation of protons attached to point defects. The energy barrier of CO_2 desorption is reduced by 28.9% under an electric field intensity of 0.01 V nm^{-1} (**Figure** 2D). **Figure** 2E shows the Confocal Raman spectroscopy during CO_2 adsorption–desorption. The new peaks at 1570 cm^{-1} and 2348 cm^{-1} are assigned to CO_2-amine interaction products (Hadjiivanov, K. I., et al. Power of infrared and Raman spectroscopies to characterize metalorganic frameworks and investigate their interaction with guest molecules. *Chemical Reviews*, 121, 1286-1424, (2021)). Additionally, a distinct C-H stretching peak develops at 2920 cm^{-1}, indicating protonation of the point defect site (Cançado, L. G., et al. Quantifying defects in graphene via raman spectroscopy at different excitation energies. *Nano Letters*, 11, 3190-3196, (2011); Elias, D. C., et al. Control of graphene's properties by reversible hydrogenation. *Science*, 323, 610-613, (2009)). These observations align closely with the DFT-predicted reaction pathway (**Figure** 2A). Notably, the CO_2-derived species and

the C-H bond exhibit concerted emergence during adsorption and synchronous disappearance during regeneration, respectively. This coupled phenomenon confirms the reversible activation of point defect-based proton traps, providing the direct experimental evidence for the one-step mechanism in N5-dGA.

The relevant content has been updated in the pages 5-7 and **Figure** 2 of the revised manuscript.

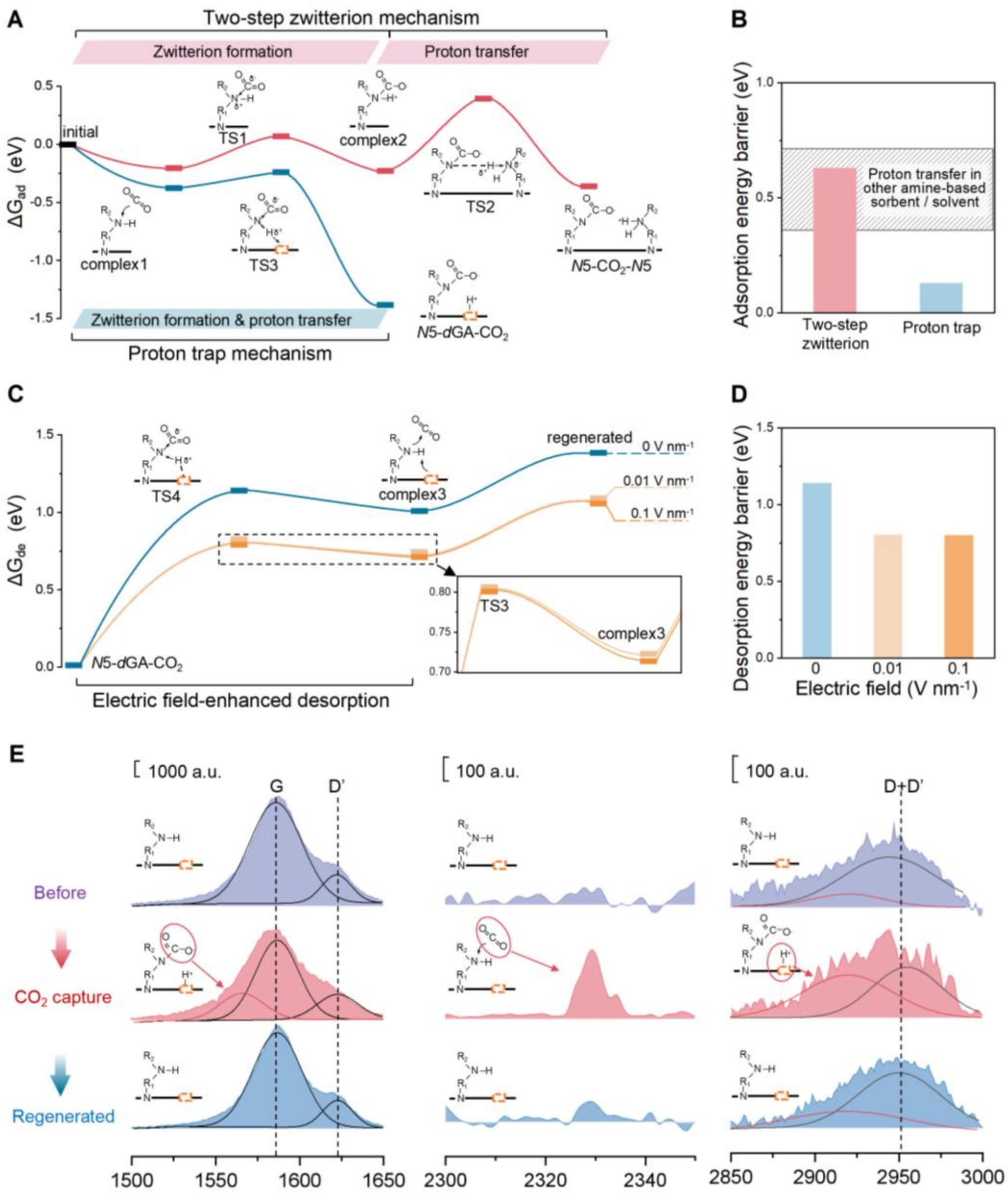

Figure 2. CO_2 adsorption and desorption pathways of *N*5-*d*GA.

(A) The CO_2 adsorption pathway and (B) adsorption energy barrier of *N*5-*d*GA via two-step zwitterion mechanism and proton strap mechanism. The orange dashed box represents a point defect on *d*GA. The gray solid box represents the reported value of proton transfer barriers for amine-based solvent or sorbent. (C) The CO_2 desorption pathway and (D) desorption energy barrier of *N*5-*d*GA

under different electric field strengths. (E) Amine-defect synergistic enhancement of proton transfer on *N*5-*d*GA revealed using the Raman spectra.

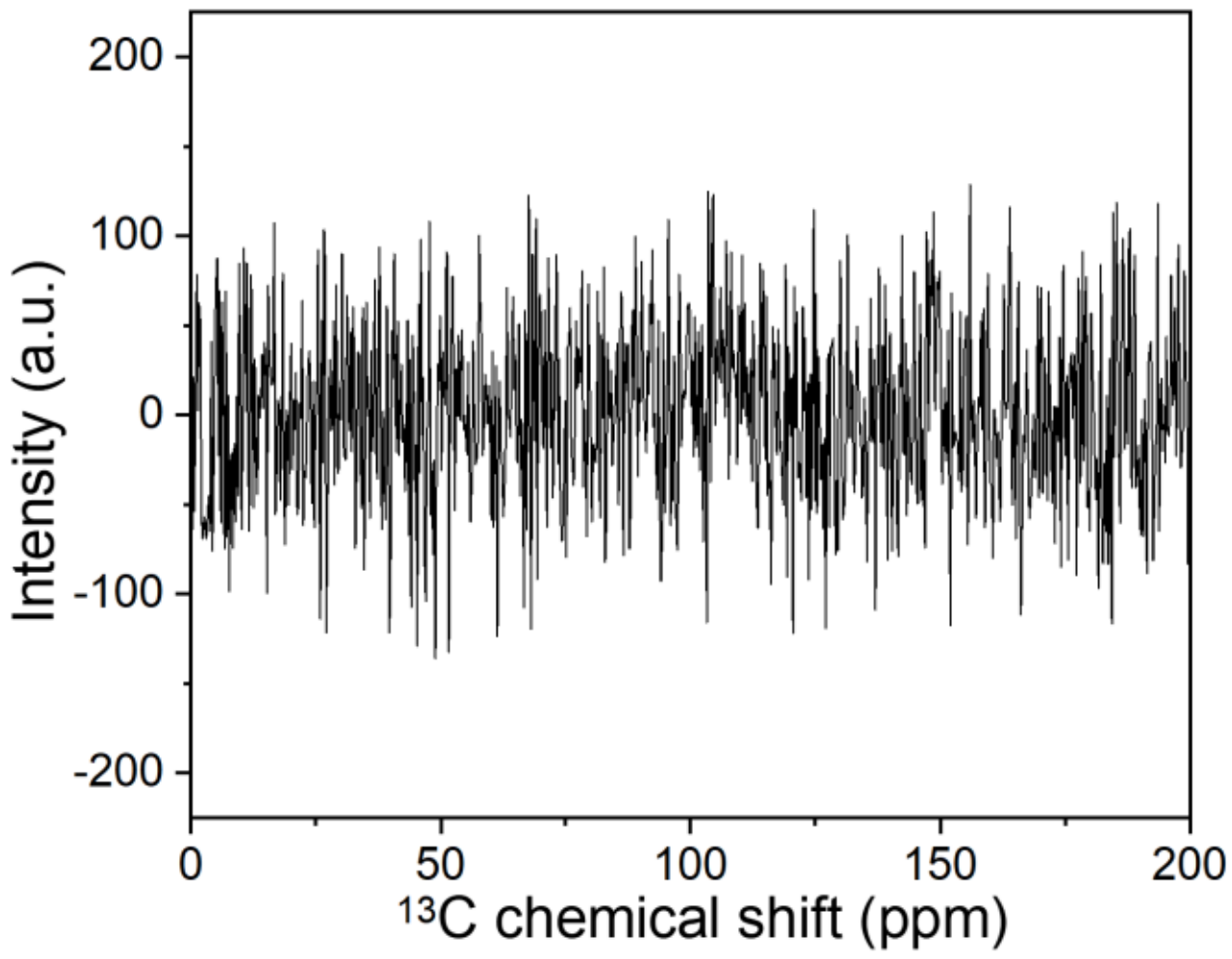

Figure R1. ^{13}C cross polarization magic angle spinning (CP-MAS) solid-state NMR spectra of *N*5-*d*GA.

Reviewer #2:

General comment: This paper presents important findings on point defect engineering on graphene aerogel for direct air capture using electric swing adsorption (ESA). However, the paper's primary limitation is its lack of novelty.

Response: Thanks for your constructive feedback. We have revised the manuscript to address the about novelty. Following your suggestion, we have added a competitive adsorption analysis, a cost-benefit analysis using renewable electricity, long-term cyclability, material stability, and kinetic performance under industrial conditions to clarify the unique contributions of this work. These additions elevate the work from a narrow focus on material synthesis to a material-to-system comprehensive assessment of ESA-DAC. Detailed responses to each point are provided below.

1. Novelty and Contribution to the Field: The paper provides valuable insights into CO2 capture in low concentration with relatively high velocity. However, it would benefit from a clearer articulation of its novel contributions compared to existing literature. Point defect engineering on graphene aerogel is not a new idea. It is already known. Specifically, how does this study advance our understanding beyond what is already known? Highlighting unique aspects or findings not previously explored would strengthen the paper's contribution to the field.

Response: Electric swing adsorption (ESA) offers a promising steam-free DAC pathway that has attracted attention (Lee, W. H. et al. Sorbent-coated carbon fibers for direct air capture using electrically driven temperature swing adsorption. Joule 7, 1241-1259, (2023); Lee, Y. H. et al. Design of electrified fiber sorbents for direct air capture with electrically-driven temperature vacuum swing adsorption. Adv. Mater., e04542, (2025); Wu, R. et al. Distributed direct air capture by carbon nanofiber air filters. Sci. Adv. 11, eadv6846, (2025)), but its efficiency has been historically capped by the 2:1 amine:CO_2 binding ratio inherent to the conventional two-step zwitterion mechanism.

Herein, this work introduces a paradigm shift. Our work introduces a previously unreported proton-trapping mechanism enabled by point defects in graphene aerogels to redefine the amine-CO_2 coordination. Engineered point defects, rather than

secondary amine groups, act as the primary proton acceptors during zwitterion deprotonation. This crucial innovation unlocks the full potential of amine functionalities, enabling them to capture CO_2 in a highly efficient 1:1 stoichiometry. In contrast to prior studies that utilized defects primarily to enhance adsorption selectivity (Hsu, Kuang-Jung et al. Graphene membranes with pyridinic nitrogen at pore edges for high-performance CO_2 capture. Nature Energy, 9, 964-974 (2024)) or create physisorption sites (Pepa Cabrera-Sanfelix, Adsorption and reactivity of CO_2 on defective graphene sheets. J. Phys. Chem. A, 113, 493-498 (2009)), our strategy uniquely repurposes defects as proton traps to maximize amine utilization. This approach fundamentally redefines CO_2 capture chemistry in amine-based sorbents. The role of point defects in accepting and releasing protons is confirmed through quantum mechanical simulations and Raman spectroscopy. Therefore, *N*5-*d*GA achieves a record CO_2 capacity of 6.57 mmol·g^{-1} from 400 ppm CO_2, which a 28.8% improvement over current benchmarks for ESA sorbents.

Additionally, a comprehensive techno-economic analysis projects operating costs for DAC of \$48-62 t^{-1} CO_2 for a 4000 t a^{-1} facility using renewable electricity. This represents a maximum cost reduction of 78% compared to traditional TSA-DAC and significantly surpasses the \$100 t^{-1} CO_2 target, establishing a clear pathway to gigaton-scale deployment. We believe these findings bridge fundamental science and scalable process engineering, representing a significant leap forward in DAC technology with broad appeal to the chemical engineering community.

The relevant content has been updated in the "Engineering a point defect-mediated proton-trap mechanism" and "Pathway to economic viability" sections of the revised manuscript.

2. No competitive adsorption study was conducted. Under high humid conditions, adsorption of water vapor onto a sorbent would likely change its operational performance. Likewise, molecular nitrogen could also compete with CO2, limiting the adsorption capacity of the material.

Response: We fully agree that competitive adsorption of water vapor and molecular

nitrogen is critical under real-world operating environments. To directly address this concern, we have conducted comprehensive competitive adsorption studies under simulated ambient air conditions (400 ppm CO_2, 80% RH, balance N_2), as shown in **Figures** 3A, 3G, and 3H.

Figure 3A demonstrates that even under humid conditions, CO_2 breakthrough occurs significantly later than N_2, confirming high selectivity for CO_2 over N_2. The CO_2 uptake remains high at 6.57 mmol g^{-1}, which is 2.9 times higher than under dry conditions (2.93 mmol g^{-1}), indicating that moisture does not hinder but may even enhance CO_2 capture. Additionally, Based on the DFT calculation, water molecules accelerate proton transfer between amine groups and point defects. The energy barrier of proton transfer reduces from 0.13 eV to 0 eV (**Figure** 3E). Water molecules capable of forming hydrogen bonds with the adsorbed CO_2 reduce the potential energy of adsorption product by 0.80 eV, resulting in the better adsorption behavior

The competitive adsorption of CO_2 and water vapor on *N*5-*d*GA was investigated. CO_2 and water vapor were simultaneously introduced into a dry sorbent at 80% RH. The breakthrough adsorption curves of water and CO_2 were obtained in **Figure** 3F. The breakthrough time of water is significantly shorter than that of CO_2. Complete electrothermal desorption of water was achieved within 200 min g^{-1} (**Figure** 3G), indicating that *N*5-*d*GA has a weak retention capacity for adsorbed water.

In summary, our material exhibits excellent selectivity for CO_2 over both N_2 and H_2O, robust performance under high humidity, and facile regeneration, all validated by experimental and computational evidence. These results demonstrate that N5-dGA is well-suited for real-world DAC applications.

The relevant content has been updated in the page 8 and **Figure** 3 of the revised manuscript.

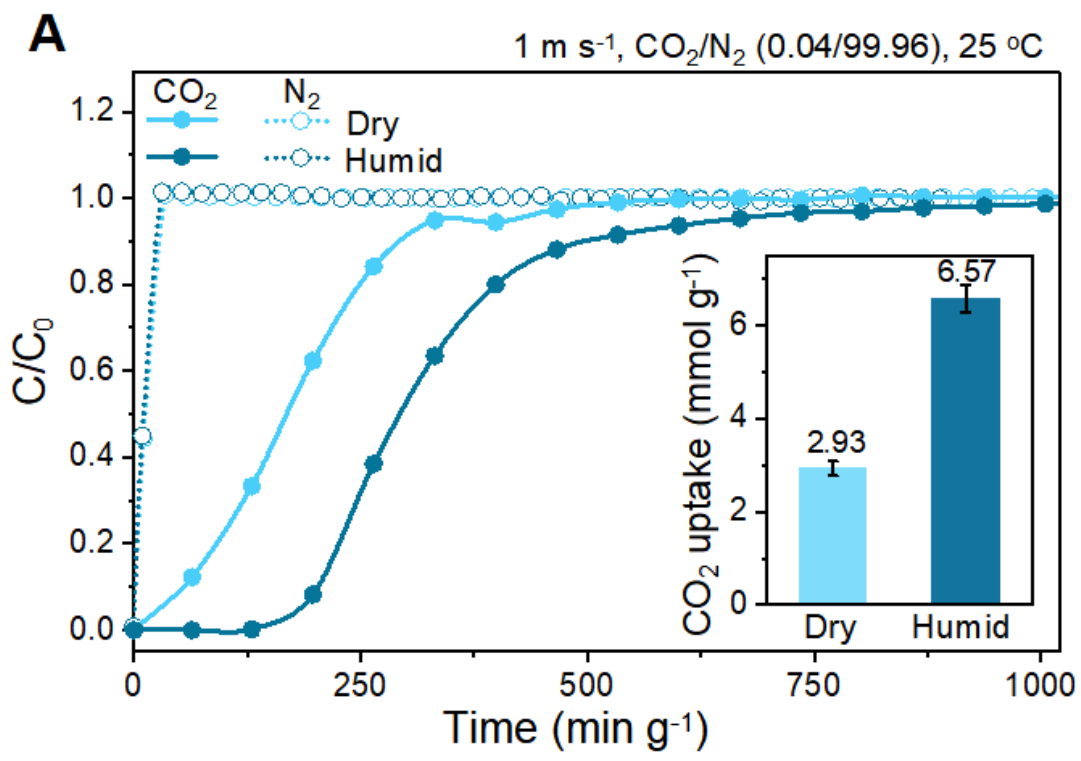

Figure 3A. Breakthrough curves and CO_2 uptakes adsorbed directly from the air of *N*5-*d*GA under dry and humid conditions.

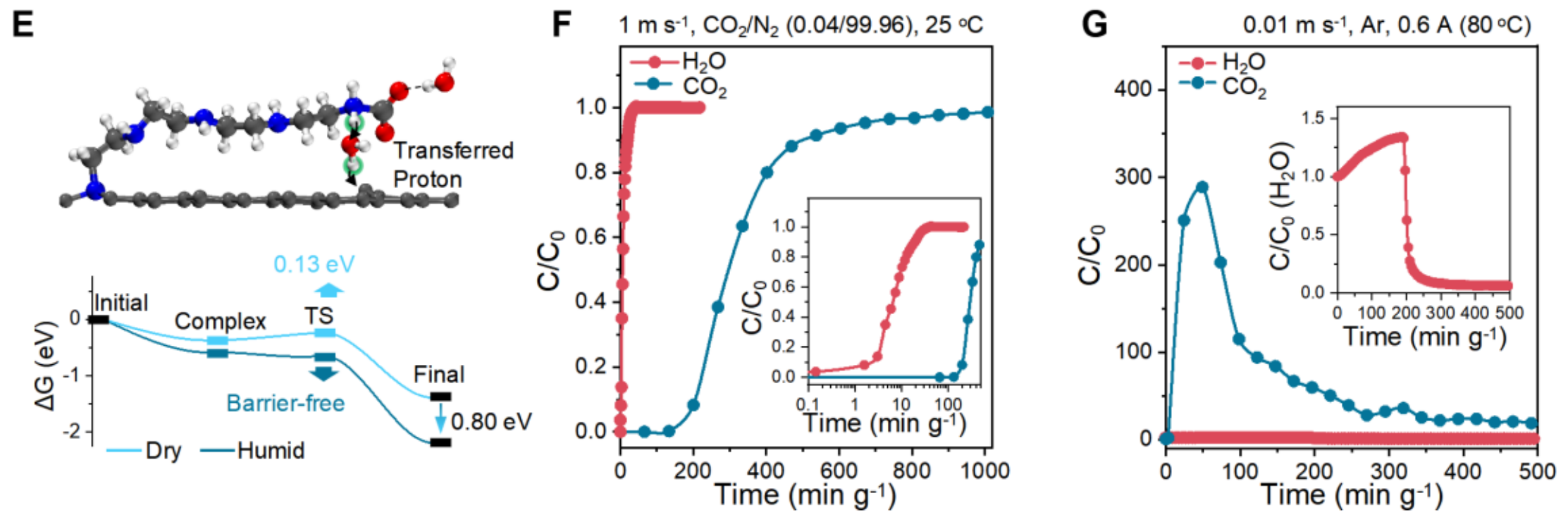

Extracted from Figure 3. (E) Schematic diagram and the ΔG plot of *N*5-*d*GA for CO_2 adsorption under humid conditions. (F) Breakthrough adsorption and (G) electrothermal desorption curves of CO_2 and water.

3. No cost-benefit analysis was done to show the merits of using renewable electricity.

Response: We sincerely thank the reviewer for raising this important point. In fact, a comprehensive cost-benefit analysis comparing ESA-based DAC powered by renewable electricity against conventional TSA systems has been conducted and is presented in **Figures** 5D-5F. The packing materials used in TSA- and ESA-DAC systems are Lewatit® VP OC 1065 and *N*5-*d*GA, respectively. The derivation of each term used in techno-economic analysis can be found in **Table** S8.

As shown in Figure 5D, at a deployment scale of 4000 t a^{-1}, ESA-DAC driven by renewable energy achieves a net CO_2 removal cost of \$ 948-1012 per tonne captured CO_2, which is 19-21% lower than TSA-DAC (\$1198-1253 per tonne) under identical conditions (Sievert, K., Schmidt, T. S. & Steffen, B. Considering technology

characteristics to project future costs of direct air capture. Joule 8, 979-999, (2024)). Specifically, there is a pronounced decrease in the proportion of variable operational expenditure (OPEX) for CO_2 capture, dropping from 23-24% in TSA-DAC to just 5% in ESA-DAC. In comparison, capital expenditures (CAPEX) show a slight decrease.

It is noted that the reduction in net removal costs of ESA-DAC system mainly comes from the decrease in variable OPEX for CO_2 capture. This is a direct result of the ESA system's ability to operate without steam handling, which significantly lowers energy consumption and associated maintenance costs. Under the solar PV scenario, the variable OPEX for ESA-DAC CO_2 capture follows a U-shaped trend with increasing desorption capacity, reaching a minimum of \$17 t^{-1} CO_2 at the desorption amount of 40 wt% (**Figure** 5E). However, at this point, the initial sorbent cost and net removal cost are \$79 t^{-1} CO_2 and \$2006 t^{-1} CO_2, respectively. Therefore, considering both CO_2 uptake and heat duty, setting the CO_2 desorption amount to 95 wt% is more favorable for achieving an optimal balance between efficiency and cost for ESA-DAC. As shown in **Figure** 5F and S20, the variable OPEX for CO_2 capture using ESA-DAC system in optimal conditions is \$48-62 t^{-1} CO_2 using various renewable electricity sources, representing a maximum reduction of 78% compared to TSA-DAC system. Furthermore, the record CO_2 uptake capacity of *N*5-*d*GA combined with its low ESA heat duty, as well as its outstanding cycling stability under humidity conditions ranging from 0 to 80% and at industrial gas flow velocities, further strengthens the economic viability of the ESA-DAC system.

The relevant content has been updated on pages 11-12 and **Figure** 5 of the revised manuscript, as well as **Figure** S18 and **Table** S8 of the Supplementary Information.

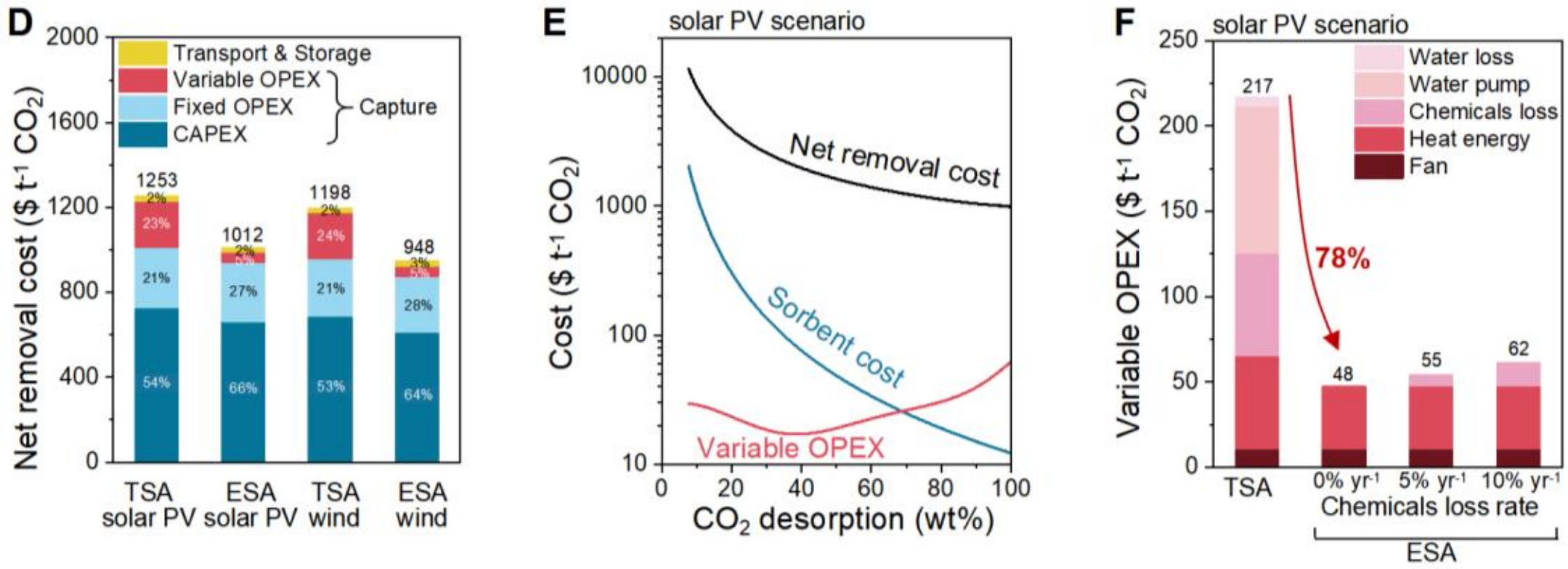

Extracted from Figure 5. (D) Cost for TSA- and ESA-DAC systems categorized by their electricity sources of a 4000 t a^{-1} scale. The stacked bars represent the CO_2 net removal cost. The amount of CO_2 desorbed is 95 wt%. (E) Relationship between DAC cost by solar PV and amount of CO_2 desorbed in the ESA-DAC system. (F) Detailed variable OPEX for CO_2 capture using TSA- and ESA-DAC systems by solar PV of a 4000 t a^{-1} scale. The amount of CO_2 desorbed is 95 wt%. The data of TSA-DAC system was reported by Sievert et al. (Sievert, K., Schmidt, T. S. & Steffen, B. Considering technology characteristics to project future costs of direct air capture. Joule 8, 979-999, (2024))

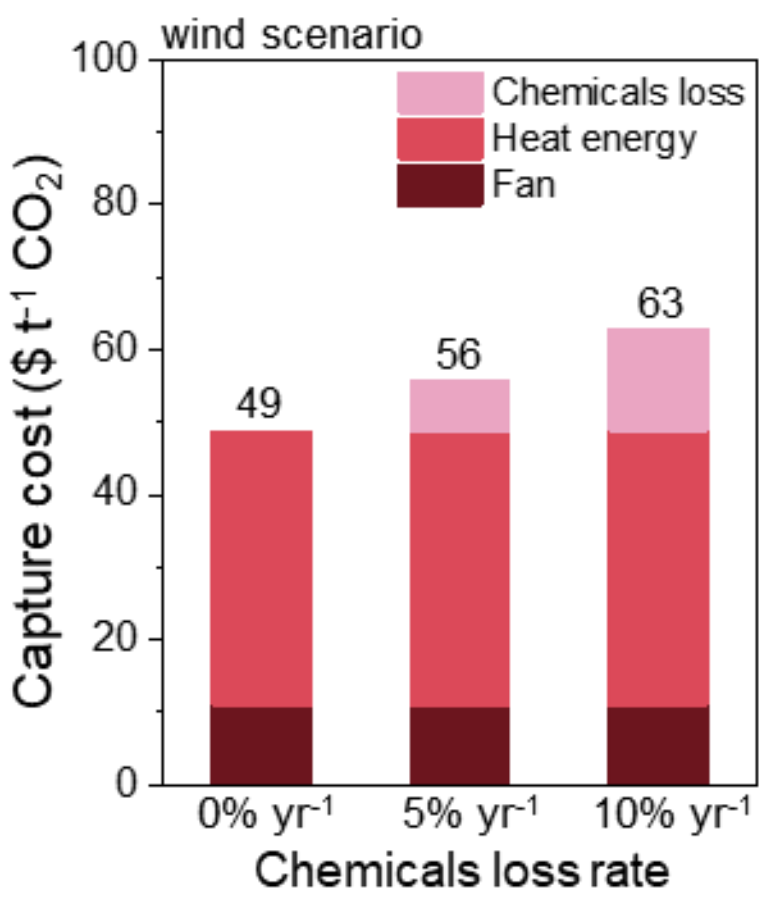

Figure S18: Detailed capture cost for ESA-DAC systems by wind of a 4000 t a^{-1} scale. The amount of CO_2 desorbed is 95 wt%.

Table S8: Process specific parameters of ESA-DAC.

Parameters	Value	Unit
Initial plant capacity	4000	t CO_2/a
adsorption capacity	6.57	mmol/g
adsorption time	6.00	h
desorption time	2.00	h
cooling time	4.00	h

Capacity factor	0.98	
Regenerative energy consumption	3.40	GJ/tCO_2
Cost of graphene aerogel	0.16	M$/t
Cost of *penta*-amine	0.004148	M$/t
Cost of Synthesis and post-processing (e.g. plasma, washing, drying)	0.003	M$/t
Regeneration temperature	80.00	℃
Specific gravity of *penta*-amine	66.7	%
Graphene aeroge specific gravity	33.3	%
Initial sorbent	9.48	t
Initial sorbent costs	0.56	M$
Capacity factor	95	%
Electricity cost of solar PV	39	$//MWh
Electricity cost of wind	37	$//MWh
Carbon intensity (x)	0.0176	
Additional electrical energy (like air blower, etc).	0.99	GJ/t CO_2

4. Multiple adsorption-desorption cycles were not investigated to demonstrate the regeneration of the material and the retention of the adsorption performance with no loss of the integrity of the material.

Response: To directly address concerns regarding long-term cycling stability and material integrity, we have now included comprehensive data from 100 consecutive adsorption–electrothermal desorption cycles.

As shown in **Figure** 4B, cyclic adsorption–desorption experiments were conducted to verify the cyclic stability of *N*5-*d*GA, which were repeated with adsorption at 25 °C and 80% RH, and electrothermal desorption at 0.6 A and 0% RH. Small fluctuations of CO_2 uptake over 10 cycles indicated a stable adsorption behavior. The results of 100 adsorption–desorption cycles are shown in **Figure** 4C. The CO_2 uptake of the as-prepared sorbent marginally decreases by 3.7% after the 100th cycle compared to the initial uptake, accompanied by virtually unchanged amine loading. Post-cycling characterization further supports the material's robustness. TGA analysis shows negligible change in amine loading, while XPS N1s spectra and Raman spectroscopy confirm that the covalent grafting between amine groups and *d*GA

remains intact, indicating no significant degradation during repeated electrothermal regeneration (**Figures** S15-S17).

The relevant content has been updated on pages 9-10 and **Figure** 4 of the revised manuscript, as well as **Figures** S15-S17 of the Supplementary Information.

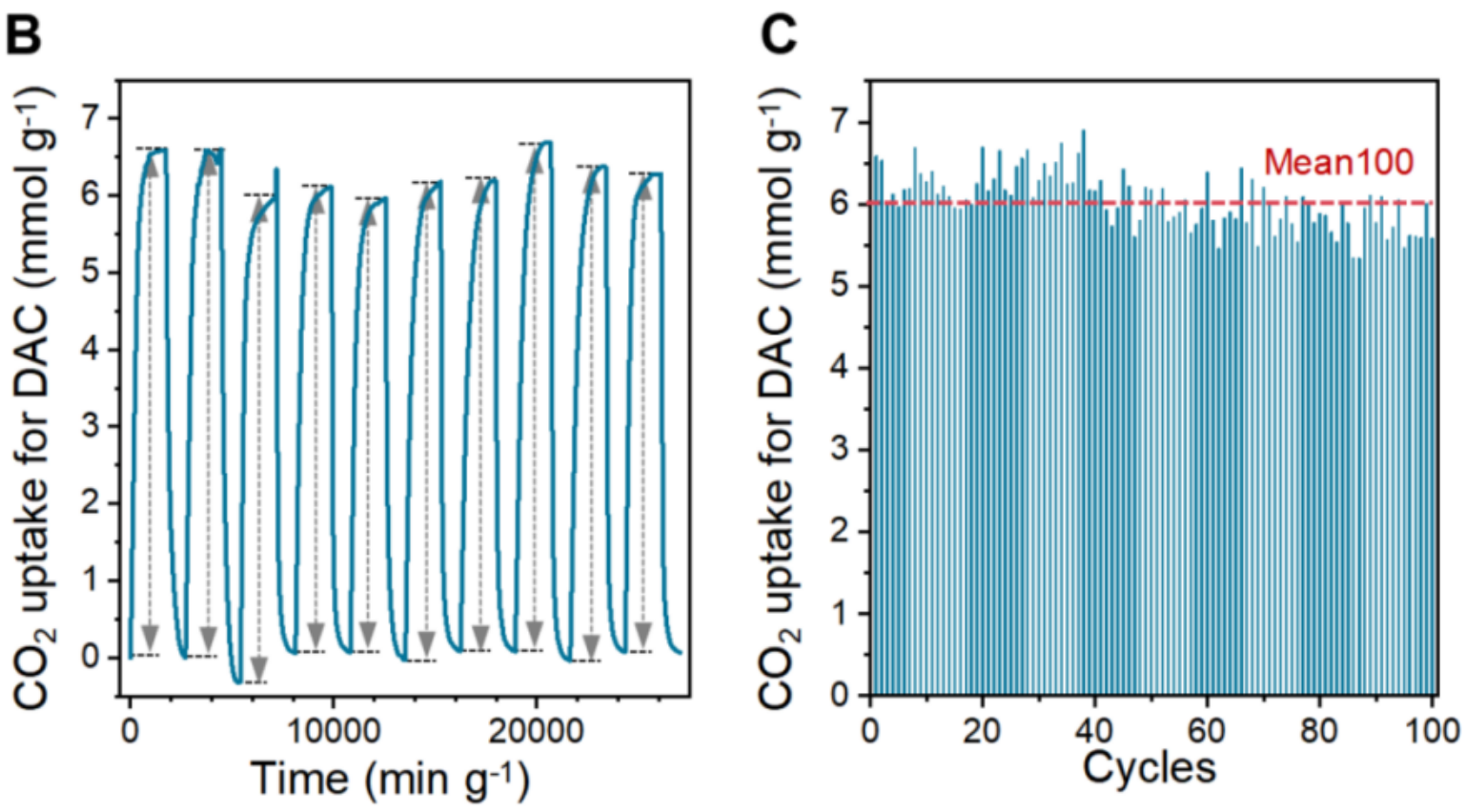

Extracted from Figure 4. (B) 10 cycles of CO_2 adsorption–electrothermal desorption. The gray arrows indicate the cyclic capacity for each cycle. (C) Bar graph of 100 cycles of CO_2 adsorption–electrothermal desorption. The horizontal red dashed lines represent the average CO_2 uptake over the 100th cycle.

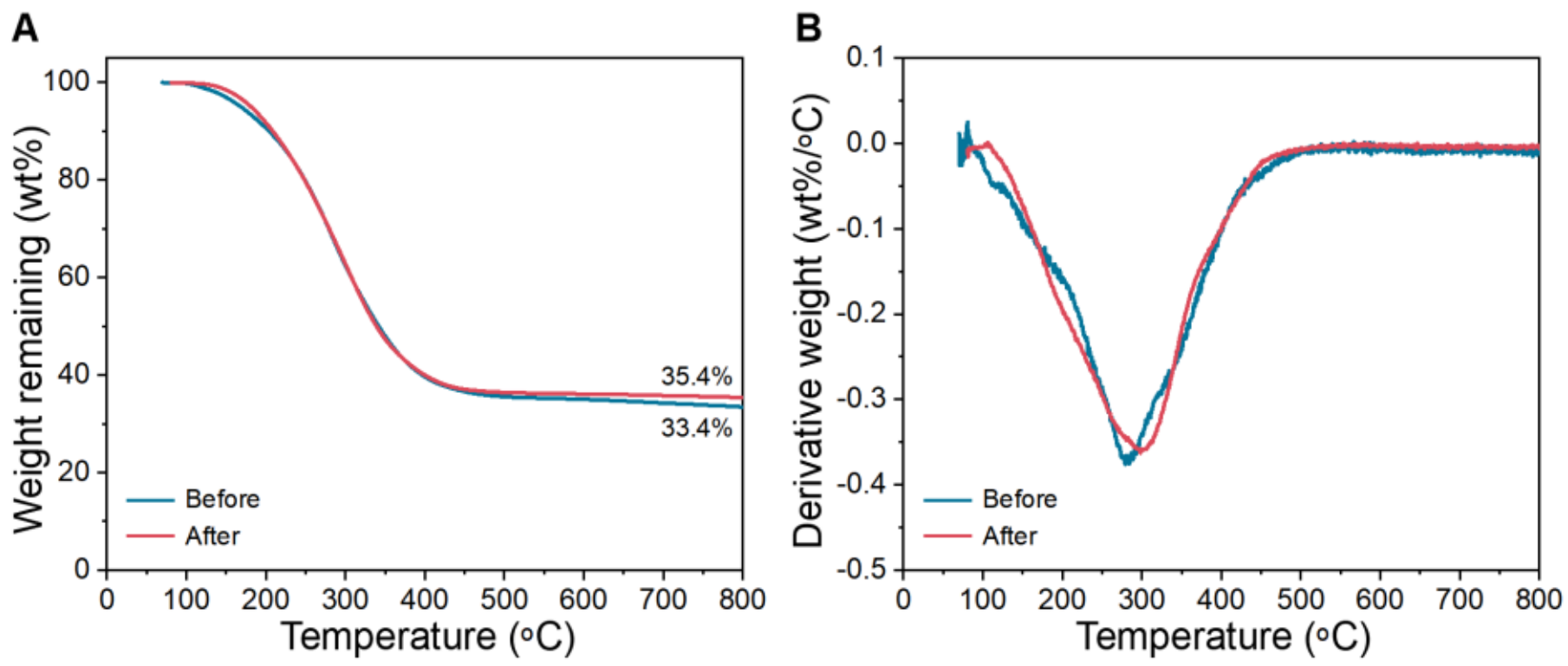

Figure S15: (A) TG curves and (B) derivative of thermal gravity curves of *N5*-*d*GA before and after 100 adsorption-desorption cycles.

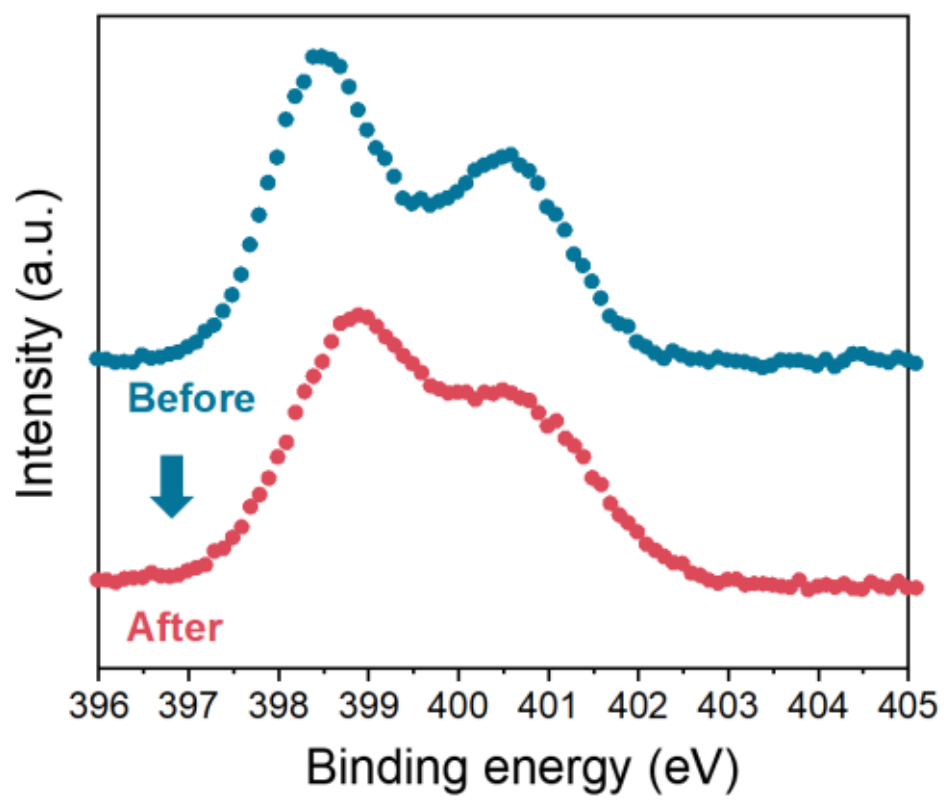

Figure S16: High-resolution XPS N1s spectra of *N*5-*d*GA before and after 100 adsorption-desorption cycles.

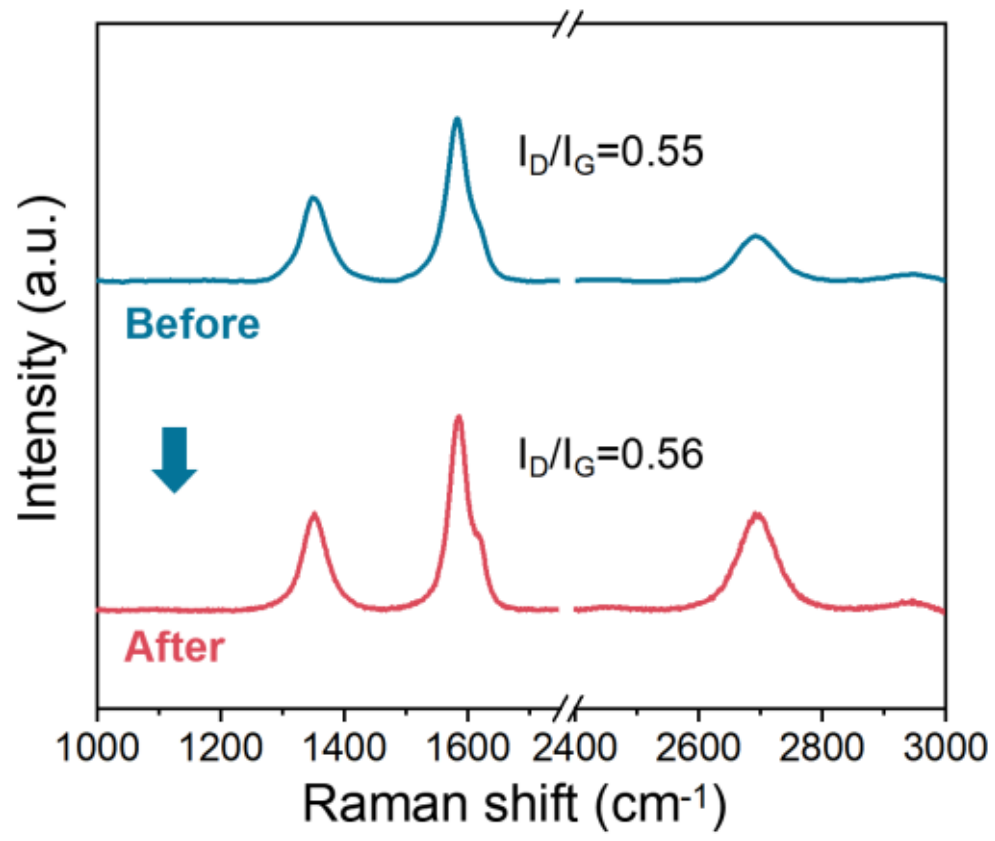

Figure S17: Raman spectroscopy of *N*5-*d*GA before and after 100 adsorption- desorption cycles.

5. I do not find any kinetic studies i.e. the rate of uptake of CO2 under different operating conditions typically encountered in industrial flue gases.

Response: To analyze the kinetics based on breakthrough adsorption curves, we fitted the experimental data using the Thomas model, which accounts for both external mass transfer and intraparticle diffusion in fixed-bed column (Chu, K. H. Fixed bed sorption: Setting the record straight on the Bohart-Adams and Thomas models. *J. Hazard. Mater.* **177**, 1006-1012, (2010)). Breakthrough tests were conducted at a gas velocity of 1 m s^{-1}, simulating high-flow industrial applications. Tests were performed with CO_2/N_2 mixtures (0.04/99.96) at 25°C under either dry (0% RH) or humid conditions (80% RH).

The kinetic studies can be found in **Figure** S8, and the high correlation coefficients ($R^2 > 0.99$) confirm an excellent fit of the model. the results demonstrate that the CO_2 adsorption rate constant is 12% lower in humid air than in dry air. This behavior is consistent with the mass transfer process of water-mediated CO_2 adsorption on amine-functionalized sorbents. As reported by Hahn et al. (Hahn, M. W., Steib, M., Jentys, A. & Lercher, J. A. Mechanism and kinetics of CO_2 adsorption on surface bonded amines. *J. Phys. Chem. C* **119**, 4126-4135, (2015)), a thin water film forms on the sorbent surface at high relative humidity, limiting the interaction of CO_2 with the active amine sites and constraining the rates of adsorption.

The relevant content has been updated on page 7 of the manuscript, and **Figure** S8 of the Supplementary Information.

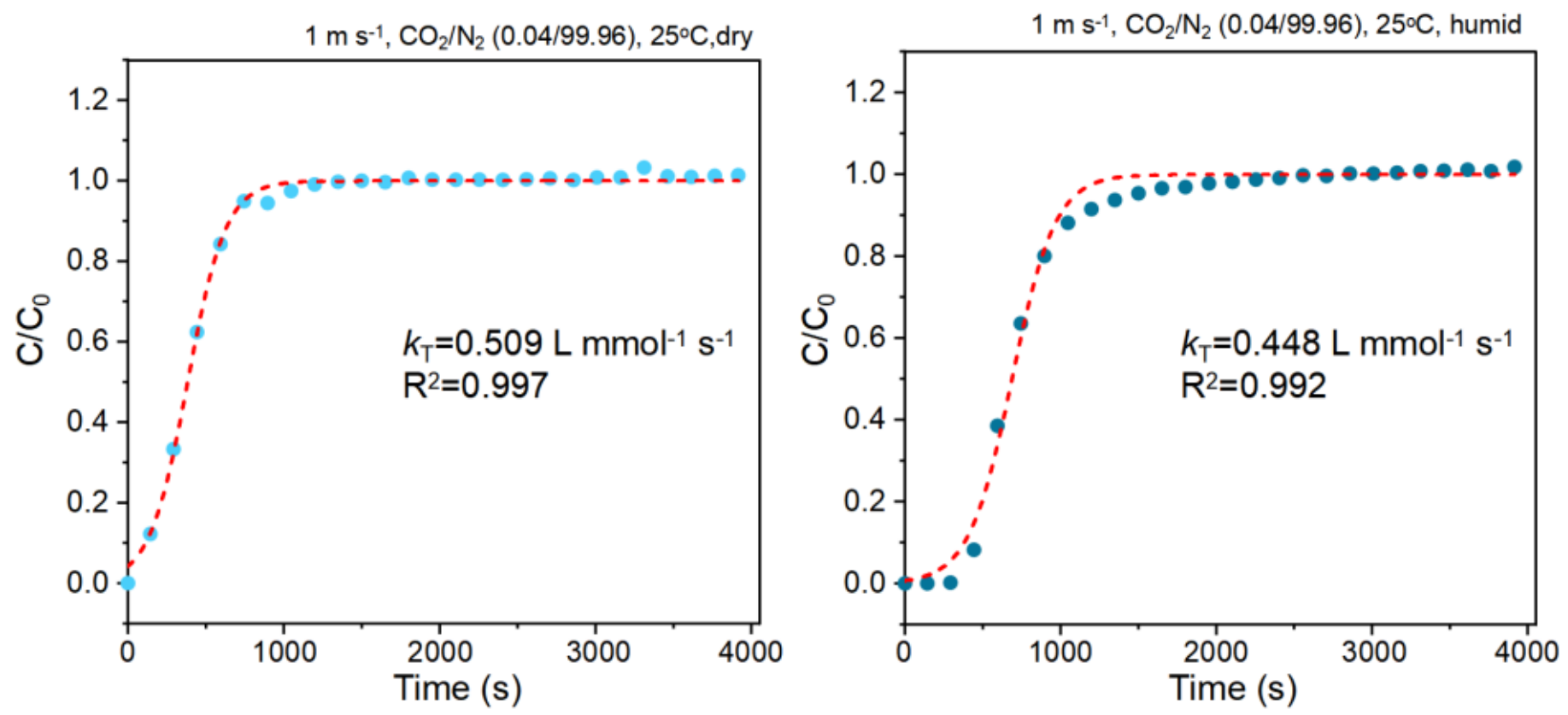

Figure S8: CO_2 adsorption rate constant of *N*5-*d*GA under dry and humid conditions. Dashed lines represent the breakthrough curves fitted using the Thomas model.

6. Statistical Analysis and Presentation of Results: The paper could benefit from a more detailed explanation of the statistical methods used, including the rationale for choosing particular models and statistical tests. Additionally, providing a clearer interpretation of the statistical significance and practical implications of the findings would be beneficial.

Response: Thank you for your kind suggestion. All experimental data presented in the manuscript are based on 3 independent replicates, and values reported in both the main text and the Supplementary Information represent the mean ± standard deviation. The consistency across replicates confirms the reliability of our results. Kinetic analysis was calculated using the typical Thomas model for breakthrough curves. The cost of DAC represents the average net cost of removing one ton of CO_2 for a given DAC technology including transport and storage, accounting for energy-related greenhouse gas emissions and the capacity factor. This approach ensures a comprehensive assessment of the true cost of CO_2 removed, considering only energy-related emissions as they dominate in life cycle analysis of DAC. A clear interpretation has been provided in the "EXPERIMENTAL DETAILS" chapter of the Supplementary Information.

7. Discussion of Limitations and Future Research: While the paper acknowledges

certain limitations, a more comprehensive discussion of these, including potential biases and the generalizability of the results, would be beneficial. Additionally, suggesting specific avenues for future research based on the study's findings and limitations could provide valuable guidance for the field.

Response: We understand your concerns. The primary objective of this work is to verify the advantages of proton-trap adsorption and electrothermal desorption mechanisms for ESA-DAC. However, we have also evaluated the performance of the materials under a range of real-world conditions. Specifically, DAC experiments were conducted in both dry air andhumid air to mimic ambient environments. The sorbent demonstrated stable performance over 100 consecutive electric-swing adsorption cycles and withstood 1,000 high-stress compression tests, showcasing its robust cyclability and mechanical integrity. Additionally, dynamic breakthrough experiments were conducted at a gas velocity of 1 m s^{-1}, which is representative of industrial-scale contactors. A preliminary techno-economic analysis was conducted at a 4000 t a^{-1} scale to evaluate the cost-benefit analysis of the ESA-DAC process using renewable electricity. These results highlight the material' s readiness for further engineering development and could provide valuable guidance for the field. In the future, we will pay more attention to the stability of *N*-*d*GA in the presence of oxygen.

8. Practical Implications and Policy Recommendations: It would be helpful to discuss the key findings in more depth, particularly how they can be realistically implemented in urban environments for direct CO2 capture from ambient air or in specific power plants or industrial environments. This could include a discussion of the feasibility, potential challenges, and ways to overcome these challenges.

Response: We appreciate your interest. In this work, the necessary experiments and discussions on the feasibility of directly capturing carbon dioxide from ambient air have been carried out. This includes operating at high gas velocity, stability over 100 cycles, and a preliminary techno-economic assessment for a 4000 t a^{-1} DAC system. This work's discussion is also based on the aforementioned feasibility verification.

9. Enhancing the Literature Review: The literature review could be expanded to provide a more comprehensive background, particularly in terms of comparing and contrasting this study's approach and findings with previous research. This would help in positioning the paper within the broader context of the field.

Response: Thanks for your suggestion. In response, we focused on recent advances in electric swing adsorption and Joule heating-driven DAC systems to provide a more comprehensive context for this work. The revised manuscript provides a detailed comparison of CO_2 capture capacity, regeneration heat and operating conditions as reported in relevant research over the past 5 years (**Figure** 5A and **Table** S1). Among the reported Joule heating-enabled sorbents (Lee, W. H. et al. Sorbent-coated carbon fibers for direct air capture using electrically driven temperature swing adsorption. Joule 7, 1241-1259, (2023); Lee, Y. H. *et al.* Design of electrified fiber sorbents for direct air capture with electrically-driven temperature vacuum swing adsorption. *Adv. Mater.*, e04542, (2025); Li, H. *et al.* Capturing carbon dioxide from air with charged-sorbents. *Nature* **630**, 654-659, (2024); Zhao, S. *et al.* Electrically conductive amine-functionalized adsorbents driven by joule heating for direct air capture. *ACS Sustainable Chemistry & Engineering* **13**, 14949-14960, (2025); Wu, R. *et al.* Distributed direct air capture by carbon nanofiber air filters. *Sci. Adv.* **11**, eadv6846, (2025)), *N*5-*d*GA exhibits the highest CO_2 uptake and the lowest heat duty. Additionally, we have carefully reviewed and updated all cited references to include the most relevant and recent contributions to the field.

The relevant content has been updated on **Figure** 5 of the revised manuscript, and **Table** S1 of the Supplementary Information.

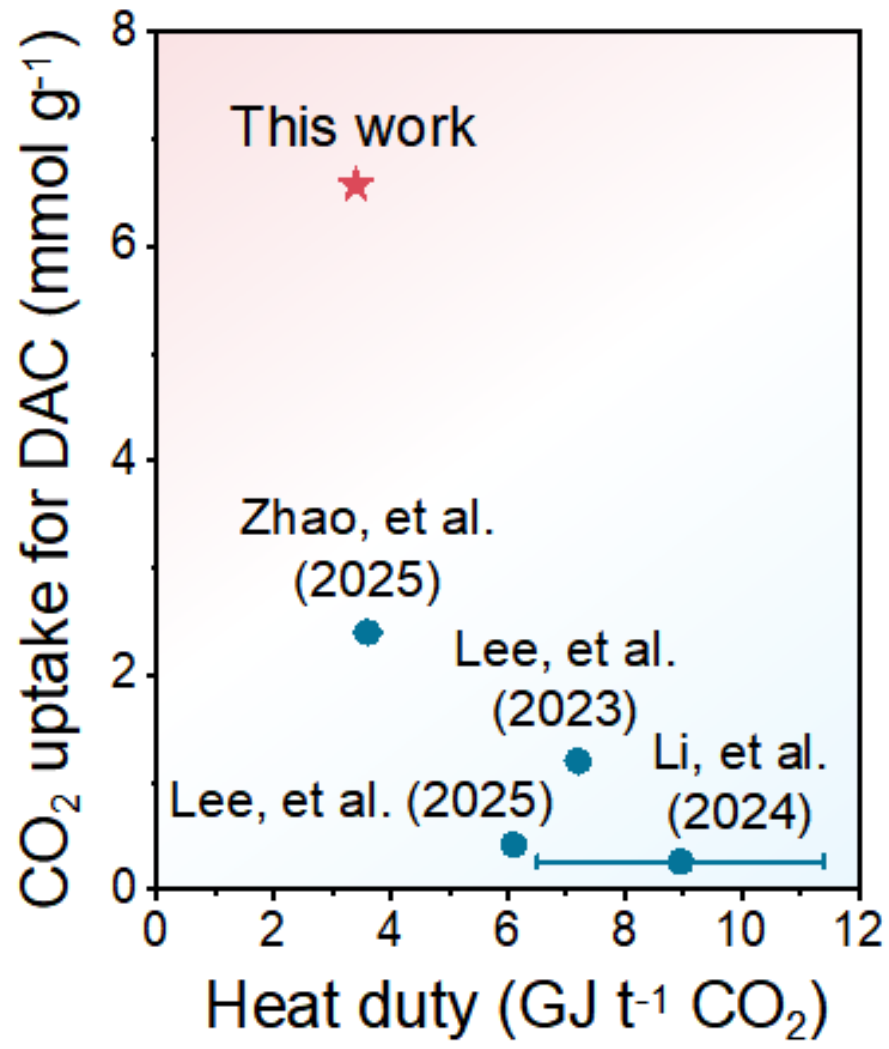

Figure 5A. A comparison of CO_2 uptake versus regeneration energy for *N*5-*d*GA and reported Joule heating-enabled sorbents, all DAC tested under ambient conditions (400 ppm CO_2, 25 °C, 1 atm).

Extracted from Table S1: CO_2 uptake for DAC of various reported sorbents.

Name	Gas flow (mL min^{-1})	Gas velocity (m s^{-1})	q (mmol g^{-1}, dry)	q (mmol g^{-1}, humid)	Heat duty for regeneration (GJ t^{-1})	Ref.
PEI-carbon fiber	100	5.89×10^{-4}	0.39	1.2	7.2	Lee, et al. (2023)
Ag-NC coated fiber	30	4.19×10^{-2}	0.42	-	6.1	Lee, et al. (2025)
PCS-OH	-	-	-	0.26	6.5~11.4	Li, et al. (2024)
Ad-0.48PE/0.32HP/0.20CB	400	0.13	-	2.4	3.5~3.7	Zhao, et al. (2025)
PEI-CNF	8(dry) 20(humid)	0.20(dry) 0.60(humid)	1.5	4.0	-	Wu, et al. (2025)
*N*5-*d*GA	1000	1.00	2.93	6.57	3.4	This work

Reviewer #3:

General comment: This paper is difficult to follow. The title discusses direct air capture, but the majority of the work concerns higher concentrations of CO2. Additionally, many different samples are discussed by their composition and synthesis are not described in all cases. This makes the reader have a very difficult time understanding the paper, and as a reader, I have doubts about the work that cannot be

easily reconciled due to missing information. It appears that work that could easily fill 1-2 full papers was crammed into a short manuscript length for a specific journal. This is unwise, and I cannot recommend publication.

Response: Thank you for your comments. We have removed the data related to other CO_2 concentrations (CO_2/N_2=15/85 and 1/99) from the main text in order to focus on DAC (CO_2/N_2=0.04/99.96). To avoid experimental redundancy, we first used computational screening to identify *penta*-amine as the optimal amine for graphene functionalization, then focused all subsequent characterization and testing on this single, high-performance formulation, rather than described in all cases.

Electric swing adsorption (ESA) offers a promising steam-free DAC pathway that has attracted attention (Lee, W. H. et al. Sorbent-coated carbon fibers for direct air capture using electrically driven temperature swing adsorption. Joule 7, 1241-1259, (2023); Lee, Y. H. et al. Design of electrified fiber sorbents for direct air capture with electrically-driven temperature vacuum swing adsorption. Adv. Mater., e04542, (2025); Wu, R. et al. Distributed direct air capture by carbon nanofiber air filters. Sci. Adv. 11, eadv6846, (2025)), but its efficiency has been historically capped by the 2:1 amine:CO_2 binding ratio inherent to conventional mechanisms. Herein, this work introduces a paradigm shift. We have engineered a point defect-mediated proton trapping network within ESA sorbents. This innovation fundamentally redefines the amine-CO_2 coordination, enabling a highly efficient 1:1 stoichiometric capture. A comprehensive techno-economic analysis projects operating costs for DAC of \$48-62 t^{-1} CO_2 for a 4000 t a^{-1} facility using renewable electricity. This represents a maximum cost reduction of 78% compared to traditional TSA-DAC and significantly surpasses the \$100 t^{-1} CO_2 target, establishing a clear pathway to gigaton-scale deployment. We believe these findings bridge fundamental science and scalable process engineering, representing a significant leap forward in DAC technology with broad appeal to the chemical engineering community.

1. The authors make many important claims. A new mechanism for CO2 capture. Unprecedented CO2 uptakes. Important impacts on DAC. However, there is no

experimental evidence for the claimed mechanism that the readers will believe (in situ IR or NMR spectroscopy). Also, much of the sorption studies are done with 100%, 15% or 1% CO2, not 0.04% CO2 for DAC. I am not convinced by much of the presented work and discussion.

Response： We sincerely thanks for your suggesting.To robustly probe the binding mechanism, we employed alternative techniques that circumvent these limitations and provide complementary insight. DFT calculation results in **Figures** 2A and 2B revealed a 79% lower energy barrier for the proton-trap pathway compared to the conventional zwitterion mechanism. The proton traps exhibit rapid regeneration under the influence of an electric field, demonstrating a high suitability for the ESA process. **Figure** 2C shows that the presence of an electric field can effectively reduce the energy barrier of the dissociation of protons attached to point defects. The energy barrier of CO_2 desorption is reduced by 28.9% under an electric field intensity of 0.01 V nm^{-1} (**Figure** 2D). **Figure** 2E shows the Confocal Raman spectroscopy during CO_2 adsorption–desorption. The new peaks at 1570 cm^{-1} and 2348 cm^{-1} are assigned to CO_2-amine interaction products (Hadjiivanov, K. I., et al. Power of infrared and Raman spectroscopies to characterize metalorganic frameworks and investigate their interaction with guest molecules. *Chemical Reviews*, 121, 1286-1424, (2021)). Additionally, a distinct C-H stretching peak develops at 2920 cm^{-1}, indicating protonation of the point defect site (Cançado, L. G., et al. Quantifying defects in graphene via raman spectroscopy at different excitation energies. *Nano Letters*, 11, 3190-3196, (2011); Elias, D. C., et al. Control of graphene's properties by reversible hydrogenation. *Science*, 323, 610-613, (2009)). These observations align closely with the DFT-predicted reaction pathway (**Figure** 2A). Notably, the CO_2-derived species and the C-H bond exhibit concerted emergence during adsorption and synchronous disappearance during regeneration, respectively.

Additionally, all CO_2 uptake values reported in the manuscript exclusively reflect 400 ppm CO_2 breakthrough experiments. The inlet gas was simulated air containing CO_2/N_2 (0.04/99.96) at a gas flow rate of 1000 mL min^{-1} under dry (0% RH) and humid (80% RH) conditions, with an outlet flow rate of 999 mL min^{-1}. To ensure

methodological clarity and alignment with the DAC focus of this work, we have removed all high-concentration CO_2 adsorption data (CO_2/N_2=15/85 and 1/99) from the main text—these data were not representative of realistic DAC conditions and created unnecessary ambiguity.

The relevant content has been updated on pages 5-6, **Figure** 2 of the revised manuscript, as well as the "CO_2 uptake for DAC" section of the Supplementary Information.

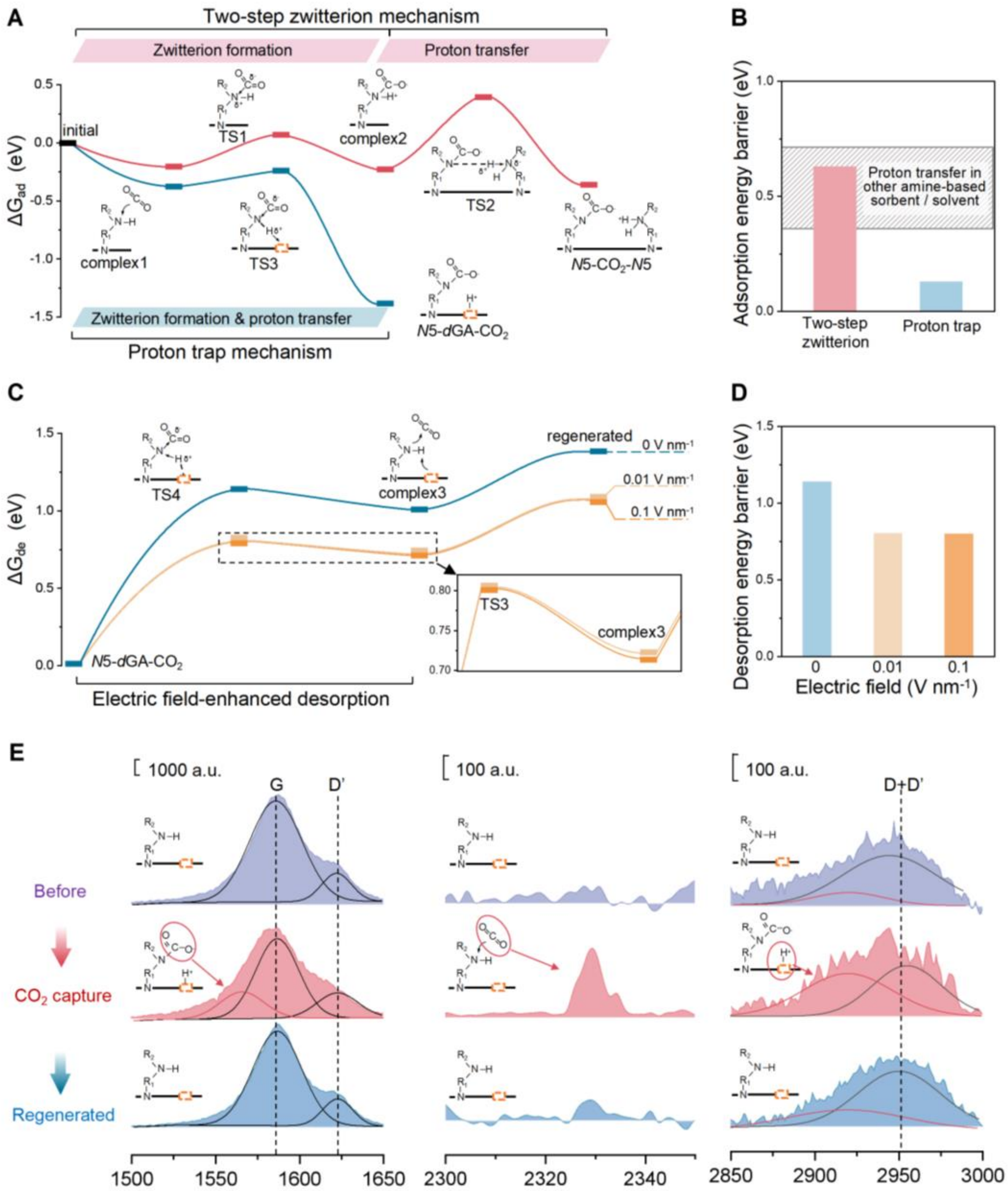

Figure 2. CO_2 adsorption and desorption pathways of *N*5-*d*GA.

(A) The CO_2 adsorption pathway and (B) adsorption energy barrier of *N*5-*d*GA via two-step zwitterion mechanism and proton strap mechanism. The orange dashed box represents a point defect

on *d*GA. The gray solid box represents the reported value of proton transfer barriers for amine-based solvent or sorbent. (C) The CO_2 desorption pathway and (D) desorption energy barrier of *N*5-*d*GA under different electric field strengths. (E) Amine-defect synergistic enhancement of proton transfer on *N*5-*d*GA revealed using the Raman spectra.

2. Given the low density, the sorbents here will have terrible volumetric productivity. Both gravimetric (you pay per kg sorbent) and volumetric (reactor and process size determined by volumetric productivity) productivity are important. The authors should compare the size of the contactor needed to obtain a fixed CO2 sorption amount with other state-of-the art sorbents.

Response: Thank you for your suggestion. As requested, we have estimated the approximate volumetric capacity of *N*5-*d*GA based on its gravimetric capacity and measured bulk density, and compared it with state-of-the art sorbents under related conditions. As shown below, the ultrahigh porosity of the graphene aerogel (>98%) results in a reduced CO_2 capacity per unit volume for *N*5-*d*GA. To obtain a fixed CO_2 sorption amount, the contactor size for *N*5-*d*GA would be larger compared to these sorbents. However, the high CO_2 uptake (6.73 mmol/g), exceptional cyclic stability (>100 cycles with <5% decay), high mechanical robustness, low heat duty (3.4 GJ t^{-1} CO_2), and low operating cost ($48-62 t^{-1} CO_2) present a compelling case for practical DAC deployment of *N*5-*d*GA.

The relevant content has been updated in **Table** S1 of the Supplementary Information.

**Name**	**CO_2 uptake ($mmol \cdot g^{-1}$)**	**Bulk density ($mg \cdot cm^{-3}$)**	**Volumetric capacity ($mol \cdot m^{-3}$)**
*N*5-*d*GA (this work)	6.57	35	52.6
MIL-101(Cr)@TEPA	0.95	430	408.5
Lewatit® VP OC 1065	1.16	670	777.2
MOR-type zeolites	1.05	950	997.5

3. Why are there references in the abstract? No journal does this?

Response: We have removed all references in the abstract.

4. The authors discuss poor conversion of renewable electricity to heat. But nobody would do that?! People would use other sources of heat, like geothermal or nuclear, with a very low or no carbon footprint. This appears to be a false motivation.

Response: We agree that low-carbon heat sources, like geothermal and nuclear energy, are highly valuable for DAC. However, our focus on ESA-DAC is not a dismissal of these alternatives, but a strategic design choice. Currently, the global energy structure is accelerating the transformation towards wind and solar renewable energy, but according to International Renewable Energy Agency (World Energy Transitions Outlook 2024), the total global wind and solar abandonment in 2024 will exceed 100 TWh (China alone has 67.4TWh as reported by National Energy Administration). Furthermore, the International Energy Agency predicts that the total amount of wind and solar curtailment may reach 2000 TWh in 2030 (Integrating Solar and Wind: Global experience and emerging challenges. 2024). These curtailments often occur in areas such as deserts and plateaus, which are rich in wind and solar energy but lack infrastructure. ESA technology offers several advantages for DAC, including the ability to rapidly and precisely control temperature, a compact module design, the elimination of the need for steam or hot water infrastructure, and the capacity to dynamically respond to intermittent renewable power. These features enable ESA-DAC to achieve distributed deployment and to flexibly offset green power fluctuations.

We have revised the manuscript to clarify this content in the "Discussion" section, emphasizing the feasibility of ESA-DAC to mitigate of wind and solar curtailments and the high cost of DAC.

5. Authors report only one reference for ESA and DAC (ref 1). There are others – a better literature search should be conducted: https://doi.org/10.1016/j.joule.2023.05.016 This might be ref 3.

Response: We focused on recent advances in electric swing adsorption and Joule heating-driven DAC systems to provide a more comprehensive context for this work. The revised manuscript provides a detailed comparison of CO_2 capture capacity, regeneration heat and operating conditions as reported in relevant research over the past 5 years (**Figure** 5A and **Table** S1). Among the reported Joule heating-enabled sorbents (Lee, W. H. et al. Sorbent-coated carbon fibers for direct air capture using electrically driven temperature swing adsorption. Joule 7, 1241-1259, (2023); Lee, Y. H. *et al.* Design of electrified fiber sorbents for direct air capture with electrically-driven temperature vacuum swing adsorption. *Adv. Mater.*, e04542, (2025); Li, H. *et al.* Capturing carbon dioxide from air with charged-sorbents. *Nature* **630**, 654-659, (2024); Zhao, S. *et al.* Electrically conductive amine-functionalized adsorbents driven by joule heating for direct air capture. *ACS Sustainable Chemistry & Engineering* **13**, 14949-14960, (2025); Wu, R. *et al.* Distributed direct air capture by carbon nanofiber air filters. *Sci. Adv.* **11**, eadv6846, (2025)), *N*5-*d*GA exhibits the highest CO_2 uptake and the lowest heat duty. Additionally, we have carefully reviewed and updated all cited references to include the most relevant and recent contributions to the field.

The relevant content has been updated on **Figure** 5 of the revised manuscript, and **Table** S1 of the Supplementary Information.

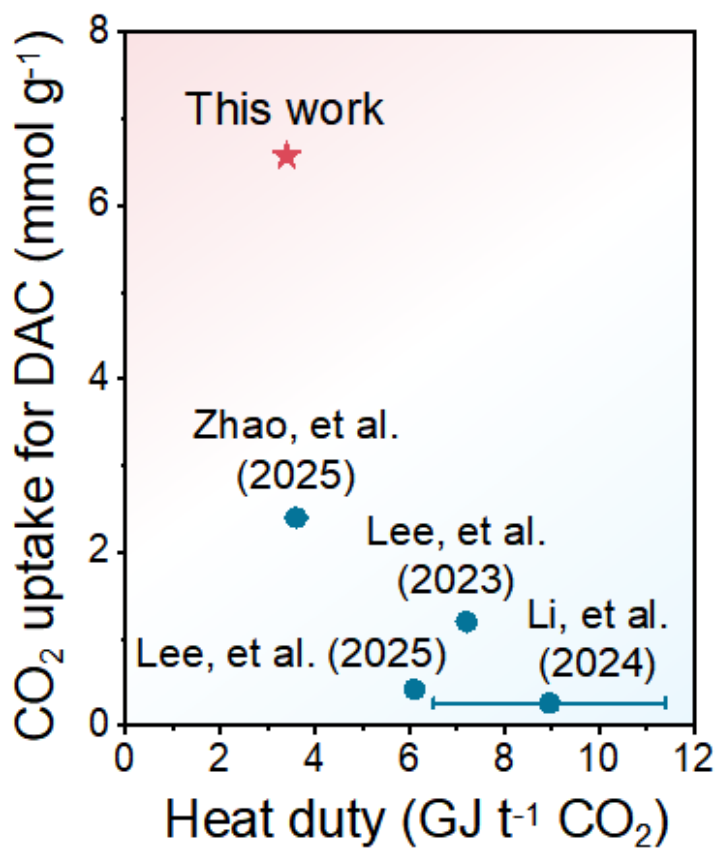

Figure 5A. A comparison of CO_2 uptake versus regeneration energy for *N*5-*d*GA and reported Joule heating-enabled sorbents, all DAC tested under ambient conditions (25 °C, 1 atm).

Extracted from Table S1

Name	Gas flow (mL min^{-1})	Gas velocity (m s^{-1})	q (mmol g^{-1}, dry)	q (mmol g^{-1}, humid)	Heat duty for regeneration (GJ t^{-1})	Ref.
PEI-carbon fiber	100	5.89×10^{-4}	0.39	1.2	7.2	Lee, et al. (2023)
Ag-NC coated fiber	30	4.19×10^{-2}	0.42	-	6.1	Lee, et al. (2025)
PCS-OH	-	-	-	0.26	6.5~11.4	Li, et al. (2024)
Ad-0.48PE/0.32HP/0.20CB	400	0.13	-	2.4	3.5~3.7	Zhao, et al. (2025)
PEI-CNF	8(dry) 20(humid)	0.20(dry) 0.60(humid)	1.5	4.0	-	Wu, et al. (2025)
*N*5-*d*GA	1000	1.00	2.93	6.57	3.4	This work

6. Page 2, lines 67-68, 0.4 to 0.65 is NOT a wide range of ratios, despite what is claimed.

Response: We thank the reviewer for this careful observation. While the ID/IG ratio from 0.4 to 0.65 is not a wide range, it represents a precisely tunable window that enables continuous control over amine loading, and final adjustment of CO_2 capture performance (**Figures** S23 and S4). The manuscript has been revised to emphasize more accurately the tunability of the defect density achieved through our plasma treatment process, rather than the absolute breadth of the range.

The relevant content has been updated in page 16, "Characterization" section of the revised manuscript.

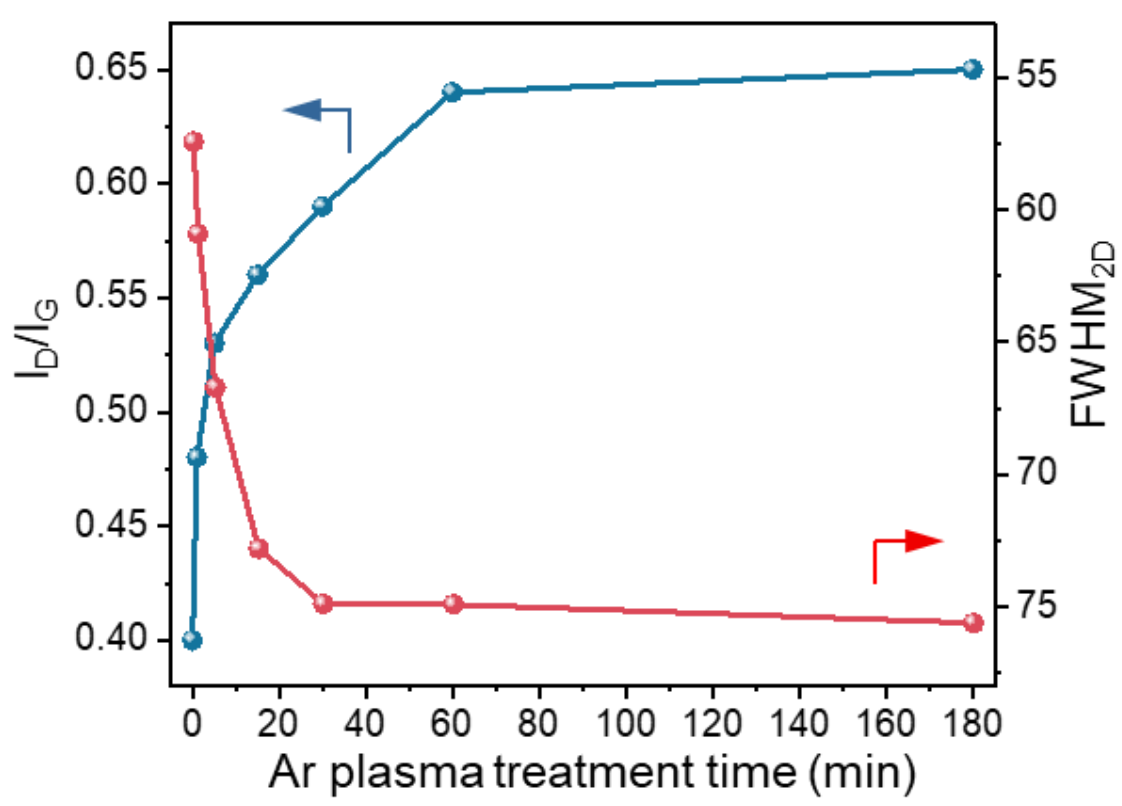

Figure S23: I_D/I_G and $FWHM_{2D}$ of *d*GA after different durations of Ar plasma treatment.

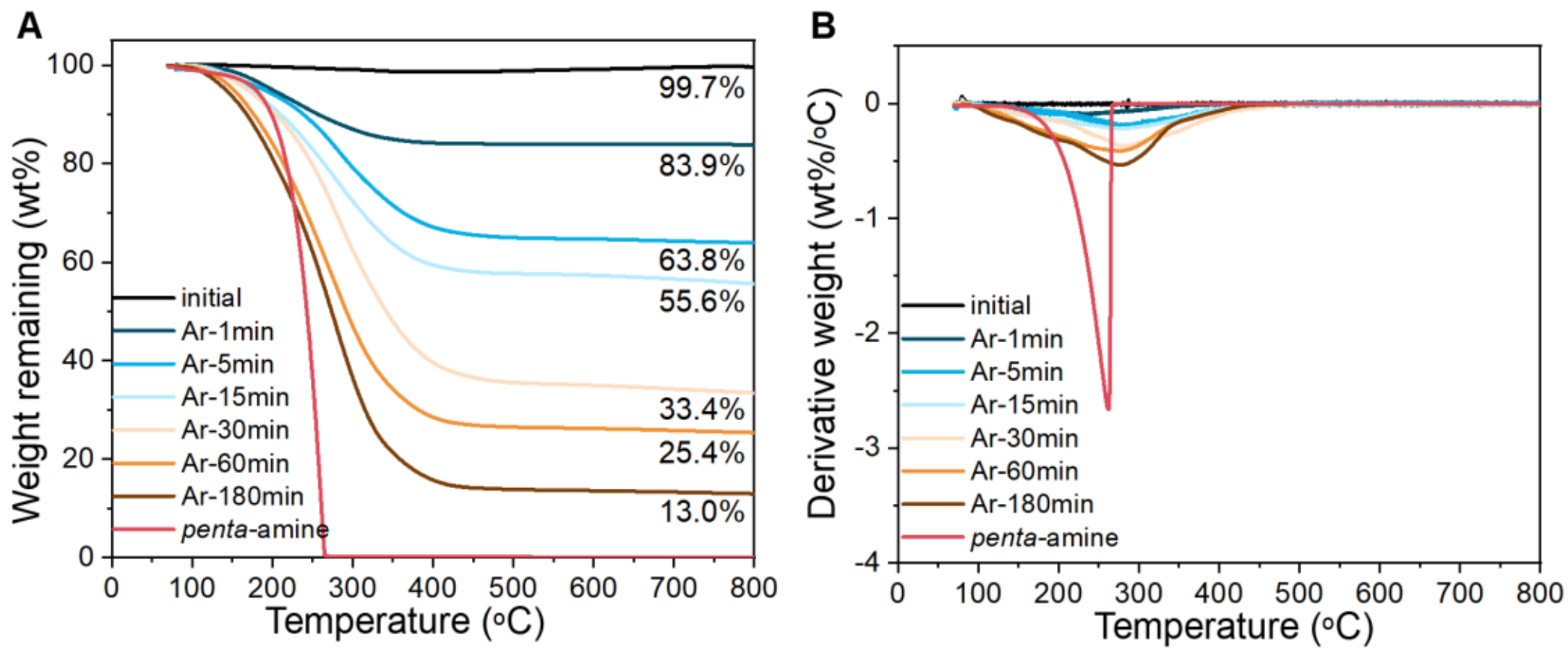

Figure S4: (A) TG curves and (B) derivative of thermal gravity curves of *N*5-*d*GA after different durations of Ar plasma treatment.

7. It is claimed that XPS proves covalent attachment of amines to dGA. I do not understand this and it is not explained. The authors should be aware that technical grade TEPA (penta) has amine macrocycles – see https://papers.ssrn.com/sol3/papers.cfm?abstract_id=3814668

Response: To clarify the mechanism of covalent attachment, we performed a control experiment using pristine graphene aerogel functionalized under identical conditions with industrial-grade TEPA, referred to as *penta*-amine-loaded GA. As shown in **Figure** S27, the N1s spectrum of *penta*-amine-loaded GA exhibits only pyridinic nitrogen (~398.4 eV, assigned to -NH-) and pyrrolic nitrogen (~399.8 eV, assigned to $-NH_2$). In contrast, *N*5-*d*GA displays a new peak at ~400.7 eV, characteristic of graphitic nitrogen, accompanied by a marked decrease in its pyrrolic component. This result indicates that the terminal $-NH_2$ groups of TEPA undergo covalent bonding with point defects in *d*GA to form stable C-N linkages and generate graphitic nitrogen moieties. The control experiment clearly demonstrates that defect sites are essential for the covalent grafting of TEPA.

The relevant content has been updated in page 16, "Characterization" section of the revised manuscript, as well as **Figure** S27 of the Supplementary Information.

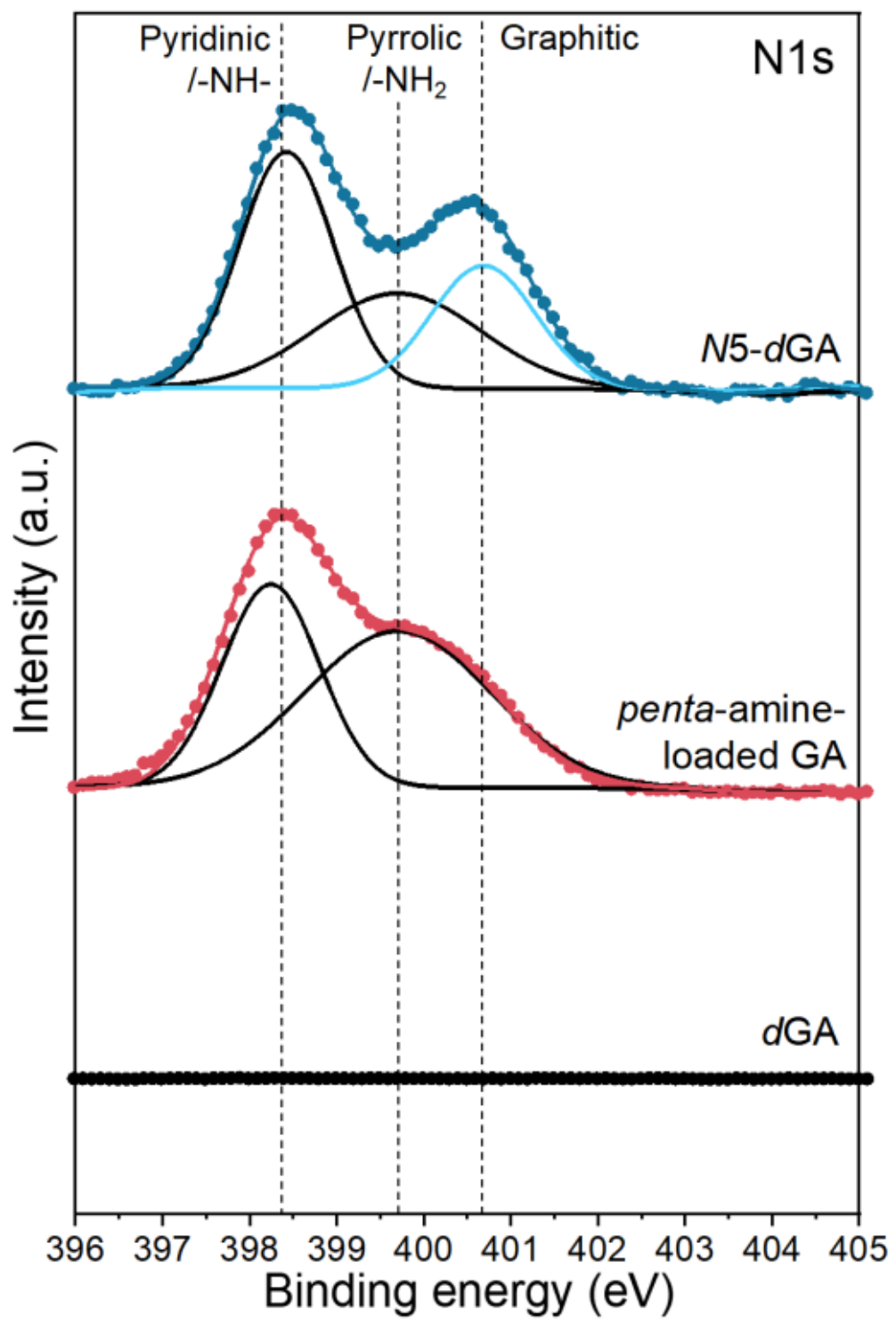

Figure S27: High-resolution XPS N1s spectra of *N5*-*d*GA, *penta*-amine-loaded GA and *d*GA.

8. NdGA is not defined in the paper but is extensively discussed. How can a reader assess the paper is key samples are not described in the paper or the SI?

Response Thank you for the suggestion. The first mention of *N*-*d*GA in the Introduction has been defined as an amine-functionalized point defect-consisting graphene aerogel. In response to this definition, a schematic illustration of *N*-*d*GA has been added to the Introduction (**Figure** 1), clearly depicting its structure. All subsequent references to *N*-*d*GA are consistent with this definition, ensuring clarity for readers. The relevant content has been updated in page 4, "Introduction" section and **Figure** 1 of the revised manuscript.

"Here, we report a paradigm shift in ESA sorbent design by engineering a point defect-mediated proton trapping network. This innovation effectively redirects proton management during CO_2 capture, enabling all amine functional groups to directly bind CO_2 in a 1:1 stoichiometric ratio (**Figure** 1A, Reaction 3). Point defects introduced into carbon frameworks can create three-dimensional unpaired electrons via sp^3 hybridization, according to recent reports (Ramprasad, R., Zhu, H., Rinke, P. & Scheffler, M. New perspective on formation energies and energy levels of point defects

in nonmetals. *Phys. Rev. Lett.* **108**, 066404, (2012); Xu, M., Li, A., Pennycook, S. J., Gao, S.-P. & Zhou, W. Probing a defect-site-specific electronic orbital in graphene with single-atom sensitivity. *Phys. Rev. Lett.* **131**, 186202, (2023)). This enhances the ability of the materials to anchor protons vertically through electrostatic attraction (An, Y., Oliveira, A. F., Brumme, T., Kuc, A. & Heine, T. Stone-wales defects cause high proton permeability and isotope selectivity of single-layer graphene. *Adv. Mater.* **32**, 2002442, (2020)). Thus, point defects can serve as effective proton traps theoretically. Based on this, we designed and fabricated an amine-functionalized point defect-consisting graphene aerogel, named *N-d*GA (**Figure** 1B)."

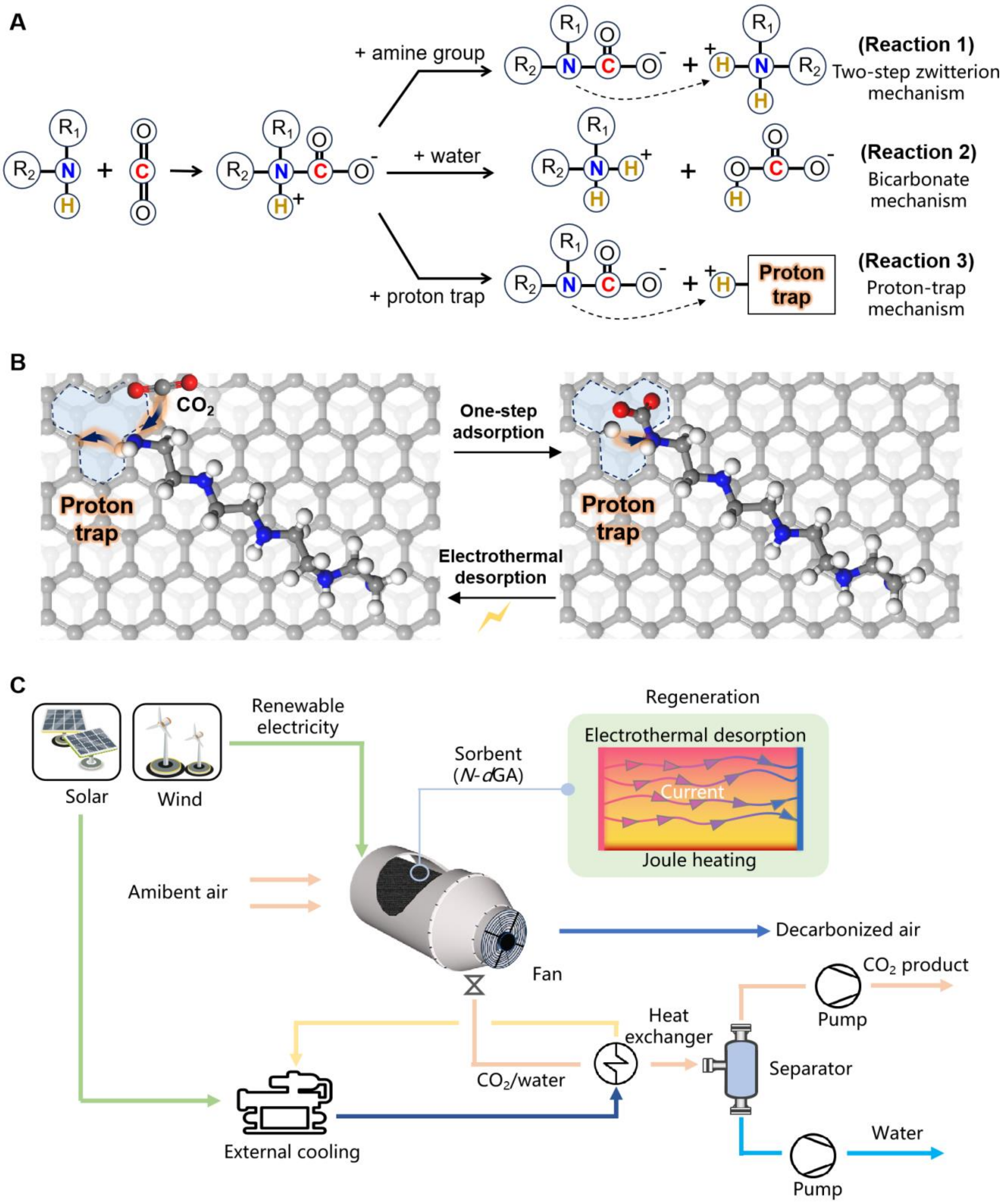

Figure 1. Schematic illustration of the proton trap-based process for atmospheric CO_2. (A) Two-step zwitterion, bicarbonate and proton-trap mechanisms. (B) CO_2 capture and release of *N-d*GA via a proton-trap mechanism. (C) Complete process for stable and efficient atmospheric CO_2 capture.

9. Fig 2, many cells in this figure use different gas compositions but that is not apparent from the caption. This makes the paper misleading and very hard to interpret.

Response: Sorry for your condusion. Now all CO_2 uptake values in the manuscript exclusively reflect 400 ppm CO_2 breakthrough experiments to ensure that the paper is

not misleading. The relevant content has been updated in **Figure** 3 of the revised manuscript.

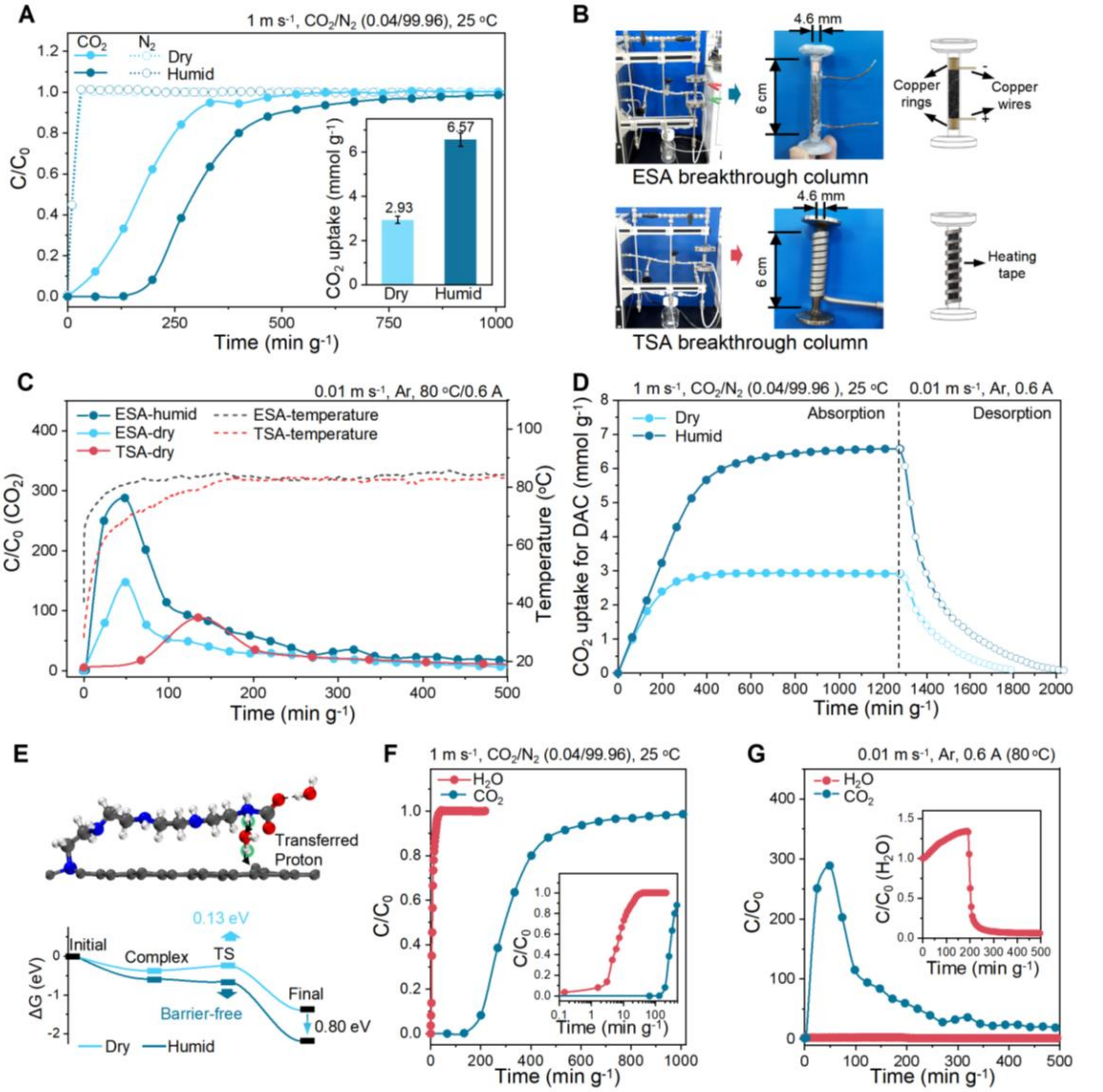

Figure 3. ESA performance of *N-d*GA for DAC.

(A) Breakthrough curves and CO_2 uptakes adsorbed directly from the air of *N*5-*d*GA under dry and humid conditions. (B) The breakthrough columns for ESA and TSA. (C) CO_2 desorption and temperature curves of *N*5-*d*GA via ESA and TSA. (D) CO_2 adsorption and desorption process of *N*5-*d*GA under dry and humid conditions. (E) Schematic diagram and the ΔG plot of *N*5-*d*GA for CO_2 adsorption under humid conditions. (F) Breakthrough adsorption and (G) electrothermal desorption curves of CO_2 and water.

10. p5, amines to not "attract" CO2. Rather, CO2 has random collisions with different sites, and when it encounters an amine the amine can nucleophilicly attack the CO2.

Response: We agree that CO_2 isn't attracted by amines, but reacts via nucleophilic

attack upon random collision with amine sites. We have carefully reviewed the entire manuscript to ensure consistent and accurate use of terminology throughout. The following content has been updated on page 5 of the revised manuscript.

"To illustrate the enhancing effect of proton traps on the adsorption reaction, the the process in which CO_2 undergoes nucleophilic attack by amine groups of *N*5-*d*GA was simulated using DFT calculations."

11. Figure 4, very large currents are required to desorb CO2, much higher than the Joule reference above (ref 3).

Response: Upon careful re-examination of original Ref. 3 (Lee, W. H. et al. Sorbent-coated carbon fibers for direct air capture using electrically driven temperature swing adsorption. Joule 7, 1241-1259 (2023)), we note that the applied current for electrothermal CO_2 desorption reaches up to 0.8～1.0 A for the 22.5 cm × 6 EA sample (see Figure 1 of this Joule reference), which is higher than our operating current of 0.6 A. Moreover, a more recent study in Advanced Material (Lee, Y. H. et al. Design of electrified fiber sorbents for direct air capture with electrically-driven temperature vacuum swing adsorption. Adv. Mater., e04542, (2025)) reports an even higher current of 1.9 A for CO_2 desorption (see Figure 4e of this Advanced Material reference). It is important to note that those studies target a desorption temperature of 110 °C, whereas our *N*5-*d*GA achieves complete CO_2 release at a significantly lower temperature of 80 °C (**Table** R1 and **Figure** 3D). This demonstrates that the 0.6 A current in our system is lower than those reported in state-of-the-art electrified sorbents.

Table R1: A comparison of current intensity for *N*5-*d*GA and reported Joule heating-enabled sorbents to desorb CO_2.

Name	Desorption temperature (℃)	Current intensity (A)	Heat duty for regeneration (GJ t^{-1})	Ref.
PEI-carbon fiber	90~110	0.8~1.0	7.2	Lee, et al. (2023)
Ag-NC coated fiber	110	1.9	6.1	Lee, et al. (2025)
*N*5-*d*GA	80	0.6	3.4	This work

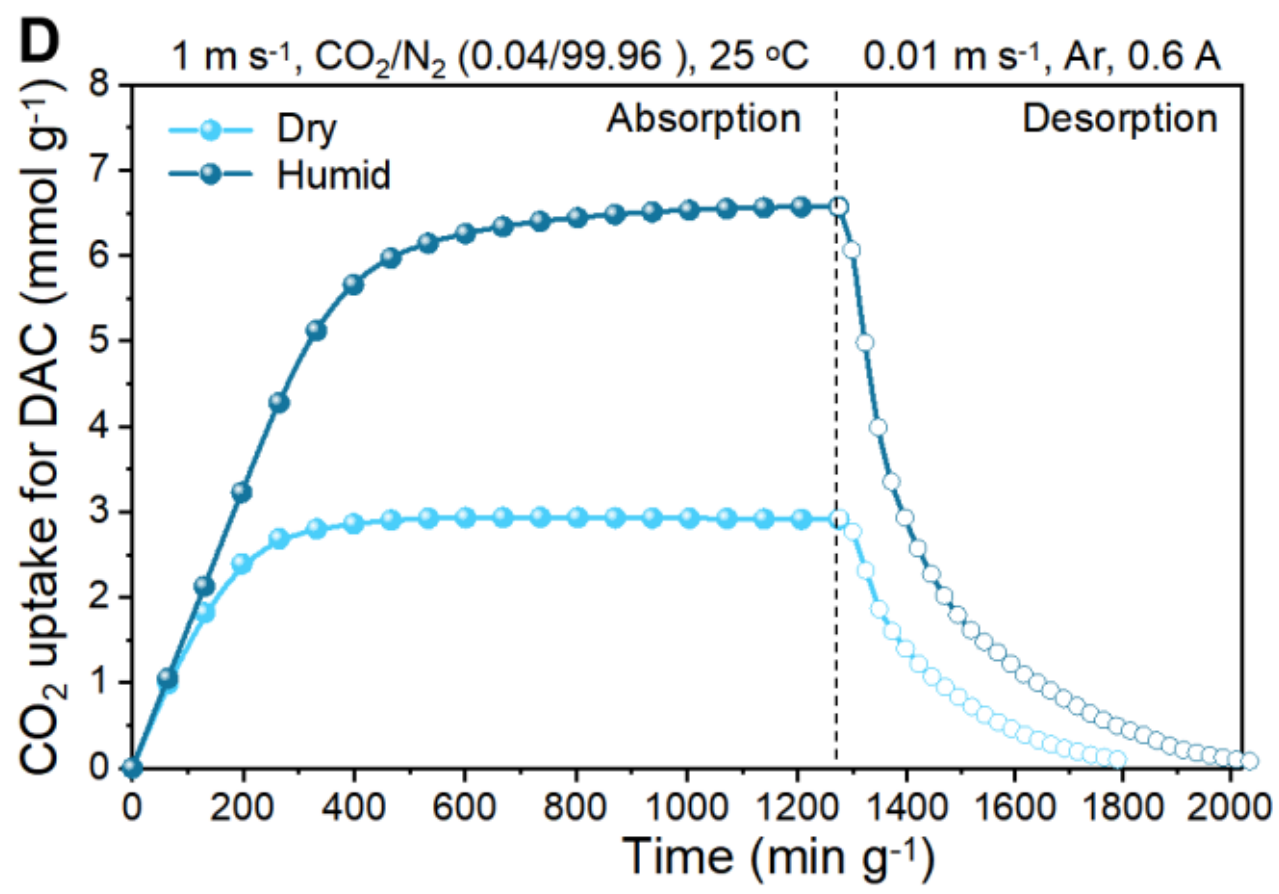

Figure 3D. CO_2 adsorption and desorption process of *N*5-*d*GA under dry and humid conditions.

12. There is little or no discussion of water. How much water is adsorbed by these materials? What is the energy penalty for water adsorption and desorption.

Response: We have conducted comprehensive competitive adsorption studies under simulated ambient air conditions (400 ppm CO_2, 80% RH, balance N_2). CO_2 and water vapor were simultaneously introduced into a dry sorbent at 80% RH. The breakthrough adsorption curves for water and CO_2 are shown in **Figure** 3F, where water exhibits a significantly shorter breakthrough time than CO_2. Complete electrothermal desorption of water was achieved within 200 min g^{-1} (**Figure** 3G), confirming *N*5-*d*GA's weak retention capacity for adsorbed water. The water-to-CO_2 mass ratio of 0.13 was determined from competitive adsorption data (**Table** S2).

As shown in **Figure** 4A, the ranges of variation for The depletion heat and sensible heat of water at different current intensities are 0.3-0.66 GJ t^{-1} and 0.03-0.06 GJ t^{-1}, respectively. While increasing current from 0.6 A to 0.8 A sharply raises sensible heat, reducing it to 0.5 A increases both sorbent sensible heat and water desorption heat. This arises from a 50% reduction in CO_2 desorption at 0.5 A, requiring larger sorbent mass to maintain equivalent CO_2 cyclic capacity. Notably, the minimum ESA heat duty of 3.4 GJ t^{-1} CO_2 occurs at 0.6 A, with excellent agreement between experimental and calculated values.

The relevant content has been updated on pages 8-9 and **Figures** 3 and 4 of the revised manuscript, and **Table** S2 of the Supplementary Information.

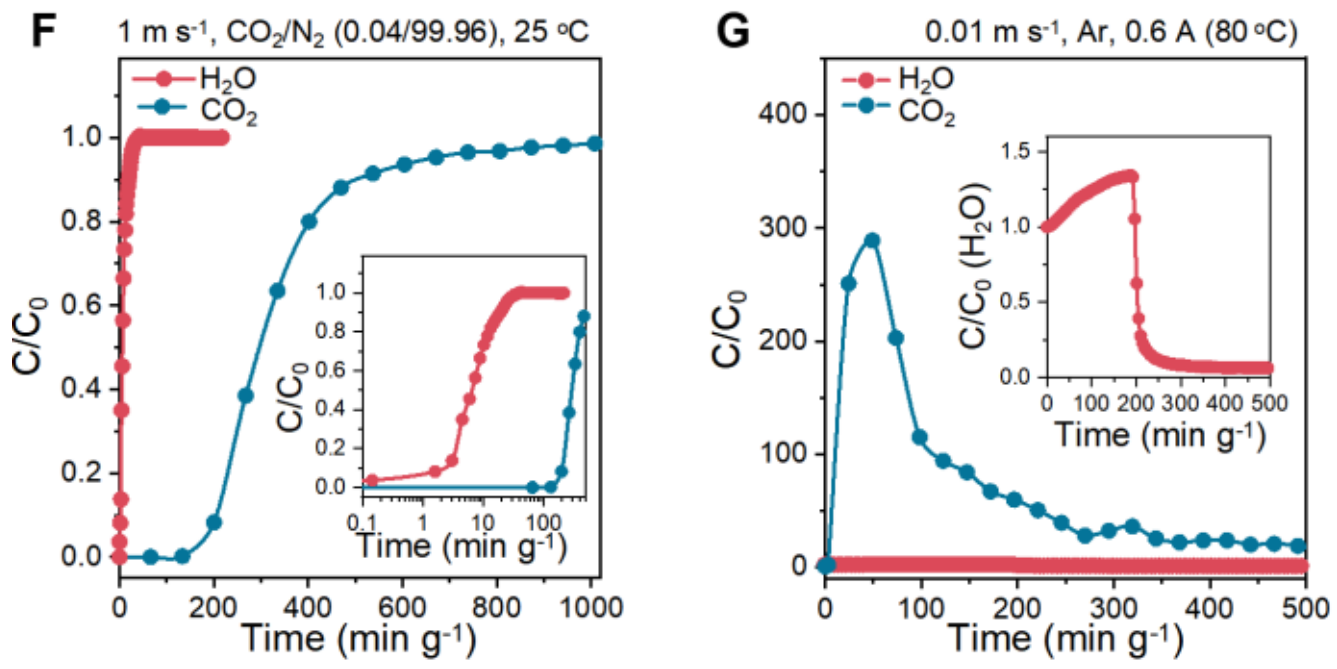

Extracted from Figure 3. (F) Breakthrough adsorption and (G) electrothermal desorption curves of CO_2 and water.

Table S2: Parameters used for calculated ESA heat duty.

Parameters	Symbol	Value				Unit
CO_2 adsorption enthalpy changes	ΔH_{ads}	60.7				kJ mol^{-1}
Water adsorption enthalpy changes	ΔH_{H2O}	42				kJ mol^{-1}
Mass ratio of adsorbed water to CO_2	$r_{H2O/CO2}$	0.13				-
Mass ratio of column to sample	$r_{column/sample}$	10.42				-
Heat capacity of CO_2	$C_{p,CO2}$	0.85				kJ kg^{-1} K^{-1}
Heat capacity of H_2O	$C_{p,H2O}$	4.18				kJ kg^{-1} K^{-1}
Heat capacity of graphene	$C_{p,graphene}$	0.75				kJ kg^{-1} K^{-1}
Heat capacity of *penta*-amine	$C_{p,amine}$	2.0				kJ kg^{-1} K^{-1}
Heat capacity of quartz	$C_{p,quartz}$	0.8				kJ kg^{-1} K^{-1}
Current intensity of electrothermal desorption		**0.5**	**0.6**	**0.7**	**0.8**	A
Electrothermal temperature		60	80	105	129	°C
The amount of CO_2 desorbed		45	95	95	95	wt%
Temperature rise of sample	ΔT_{sample}	35	55	80	104	K
Temperature rise of column	ΔT_{column}	28.7	45	56.7	68	K

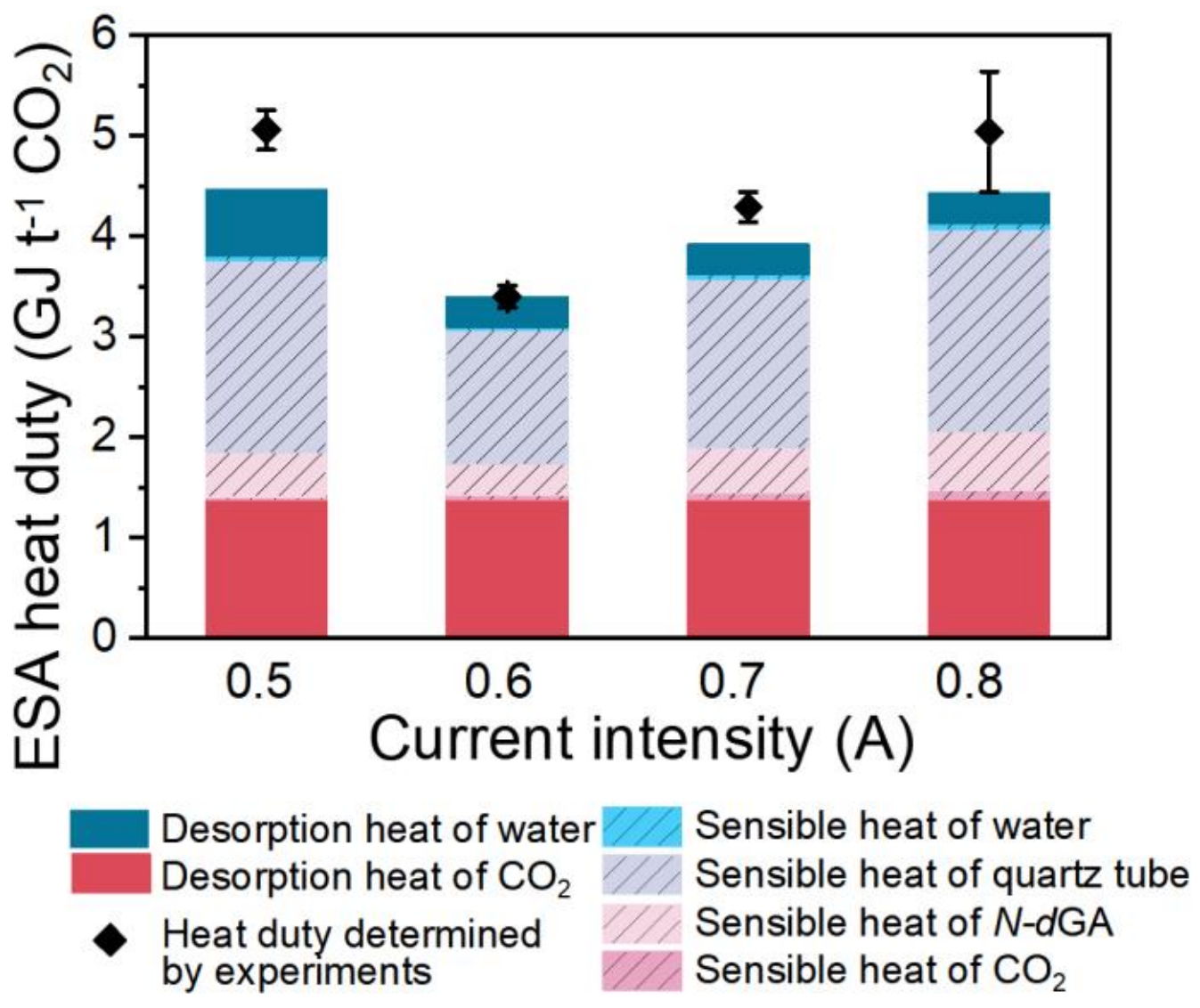

Figure 4A: Comparison of calculated and experimental ESA heat duties for CO_2 desorption using *N*5-*d*GA. The *N*5-*d*GA adsorbs CO_2 to saturation in humid air.

13. There are many steps to the synthesis of the sorbent. For DAC, we will need millions of tons of sorbents. Can this sorbent be synthesized at scale at low cost?

Response: Scalability and cost of sorbents are critical considerations that cannot be overlooked for any emerging technology. In fact, a comprehensive cost-benefit analysis comparing ESA-based DAC powered by renewable electricity against conventional TSA systems has been conducted in the revised manuscript. The packing materials used in TSA- and ESA-DAC systems are Lewatit® VP OC 1065 and *N*5-*d*GA, respectively. Our techno-economic analysis shows that the cost of synthesis and post-processing (including plasma treatment, ethanol washing, and drying) accounts for only 5.3% of the total sorbent cost (**Table** S8). Furthermore, at a deployment scale of 4000 t CO_2 per year, the material cost of *N*5-*d*GA in the ESA-DAC process is just 12% higher than that of the commercial sorbents Lewatit® VP OC 1065 used in conventional TSA-DAC. As shown in **Figure** 5, when powered by renewable electricity, ESA-DAC achieves up to 78% lower operating costs compared to TSA-DAC and delivers a capture cost well below the \$100 t^{-1} CO_2 target, establishing a clear pathway to gigaton-scale deployment.

We have added a new section "Pathway to economic viability" to discuss scalability considerations and cost trade-offs in the revised manuscript. The relevant

content has been found at pages 11-12 and **Figure** 5 of the revised manuscript, as well as **Table** S8 of the Supplementary Information.

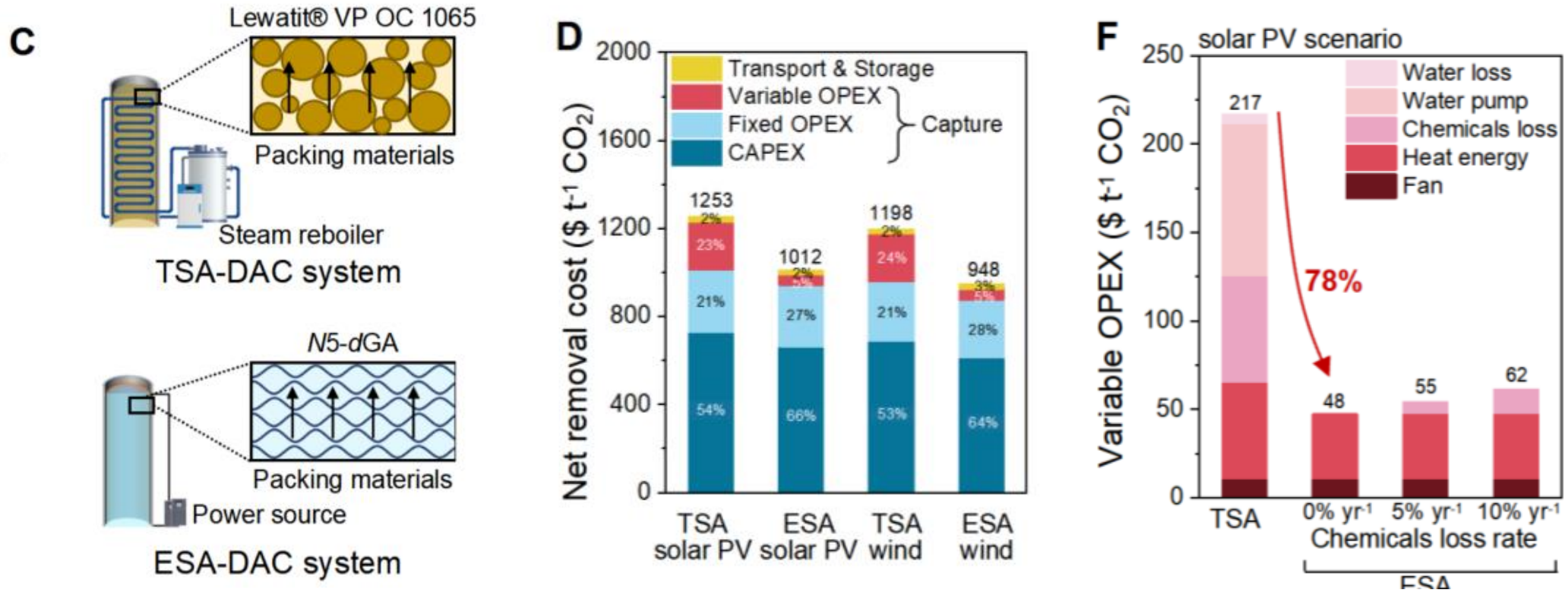

Extracted from Figure 5. (C) The schematic diagram of TSA- and ESA-DAC systems. (D) Cost for TSA- and ESA-DAC systems categorized by their electricity sources of a 4000 t a^{-1} scale. The stacked bars represent the CO_2 net removal cost. The amount of CO_2 desorbed is 95 wt%. (F) Detailed variable OPEX for CO_2 capture using TSA- and ESA-DAC systems by solar PV of a 4000 t a^{-1} scale. The amount of CO_2 desorbed is 95 wt%. The data of TSA-DAC system was reported by Sievert et al. (Sievert, K., Schmidt, T. S. & Steffen, B. Considering technology characteristics to project future costs of direct air capture. Joule 8, 979－999, (2024))

Table S8: Process specific parameters of ESA-DAC.

Parameters	Value	Unit
Initial plant capacity	4000	t CO_2/a
adsorption capacity	6.57	mmol/g
adsorption time	6.00	h
desorption time	2.00	h
cooling time	4.00	h
Capacity factor	0.98	
Regenerative energy consumption	3.40	GJ/tCO_2
Cost of graphene aerogel	0.16	M$/t
Cost of *penta*-amine	0.004148	M$/t
Cost of Synthesis and post-processing (e.g. plasma, washing, drying)	0.003	M$/t
Regeneration temperature	80.00	℃
Specific gravity of *penta*-amine	66.7	%
Graphene aeroge specific gravity	33.3	%
Initial sorbent	9.48	t
Initial sorbent costs	0.56	M$
Capacity factor	95	%

Electricity cost of solar PV	39	$//MWh
Electricity cost of wind	37	$//MWh
Carbon intensity (x)	0.0176	
Additional electrical energy (like air blower, etc).	0.99	GJ/t CO_2

14. How are TGA experiments used to assess amine loading in dGA materials?

Response: TGA experiments and amine loading quantification were performed on an SDT Q600 instrument. The sample temperature was ramped from 25 °C to 800 °C at 10 °C min^{-1} under a continuous N_2 flow of 100 mL min^{-1}. Figure S4 is these TG curves. As control, the unmodified *d*GA carrier (initial) showed negligible mass loss (0.3%) at 800 °C, while all amine-functionalized samples exhibited a single major weight loss peak (**Figure** S4B). This peak (200–350 °C) was near to the weight loss peak of pure penta-amine, confirming that the decomposition corresponds to amines. Therefore, amine loading (wt%) can be calculated from the weight loss, which consistent with other reported amine-functionalized sorbents (Mohammadi, A. & Pour, A. N. Triethylenetetramine-impregnated ZIF-8 nanoparticles for CO_2 adsorption. *J. CO_2 Util.* 69, 102424, (2023); Shen, Y. et al. A pore matching amine-functionalized strategy for efficient CO_2 physisorption with low energy penalty. *Chem. Eng. J.* 432, 134403, (2022)).

The relevant content has been updated on **Figure** S4 of the Supplementary Information.

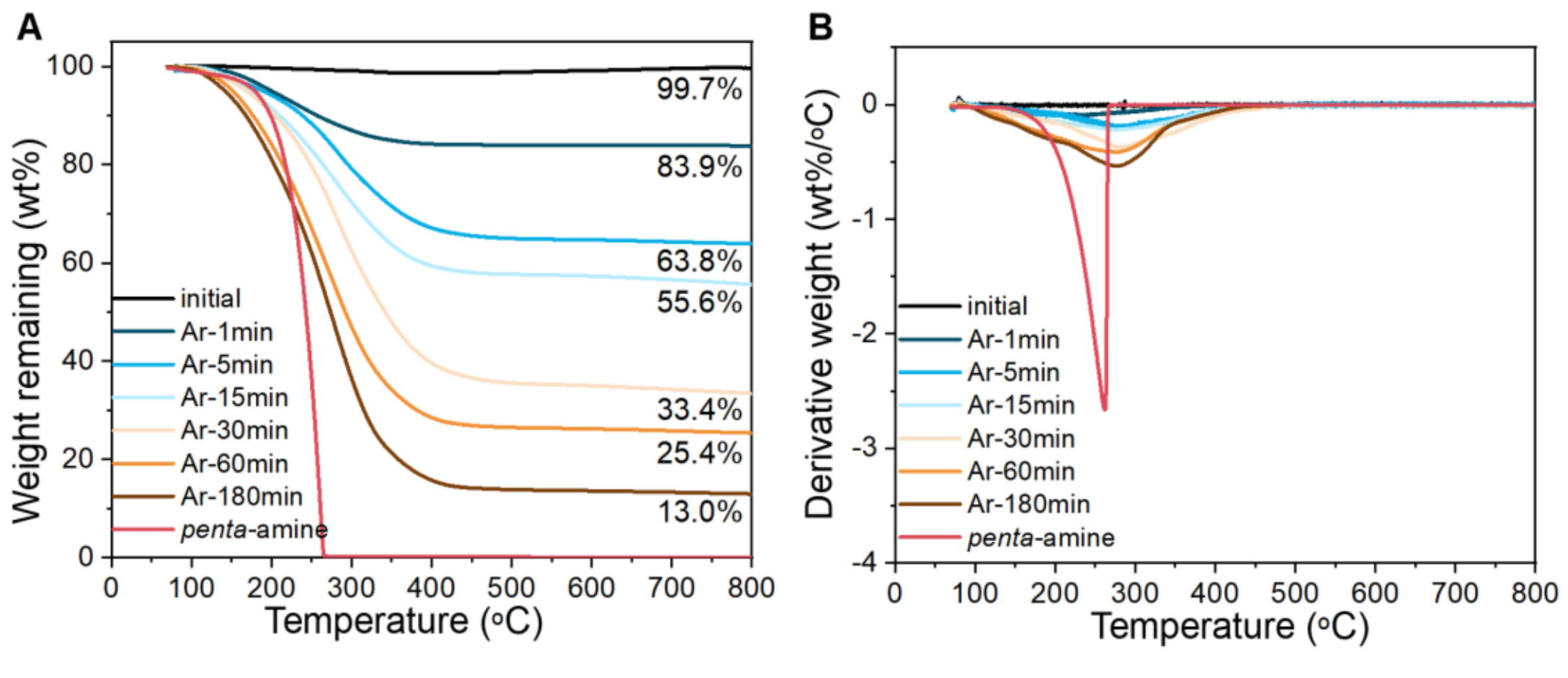

Figure S4: (A) TG curves and (B) derivative of thermal gravity curves of *N*5-*d*GA after different durations of Ar plasma treatment.

15. Reproducibility of the Dawson method of measuring CO2 uptake should be presented.

Response: We used the static gravimetric method reported by Dawson et al. (Dawson, R. et al. 'Dry bases': Carbon dioxide capture using alkaline dry water. Energy Environ. Sci. 7, 1786-1791, (2014)) to measure CO_2 uptake under pure CO_2 (1 bar) in an Ar glovebox (<0.1 ppm H_2O), as conventional TGA is unreliable for ultralow-density aerogels. This protocol enabled accurate determination of the theoretical maximum adsorption capacity during material screening. To confirm reproducibility, each sample was independently tested three times using this method and the average value was reported. The results in **Figures** S3 and S5 show excellent consistency.

Notably, based on those results, *N*5-*d*GA with 66.6 wt% amine loading was selected for subsequent DAC tests under realistic conditions (CO_2/N_2=0.04/99.96, gas velocity=1 m s^{-1}), which were evaluated by dynamic breakthrough experiments.

The relevant content has been updated in "CO_2 adsorption measurements" section of the Supplementary Information.

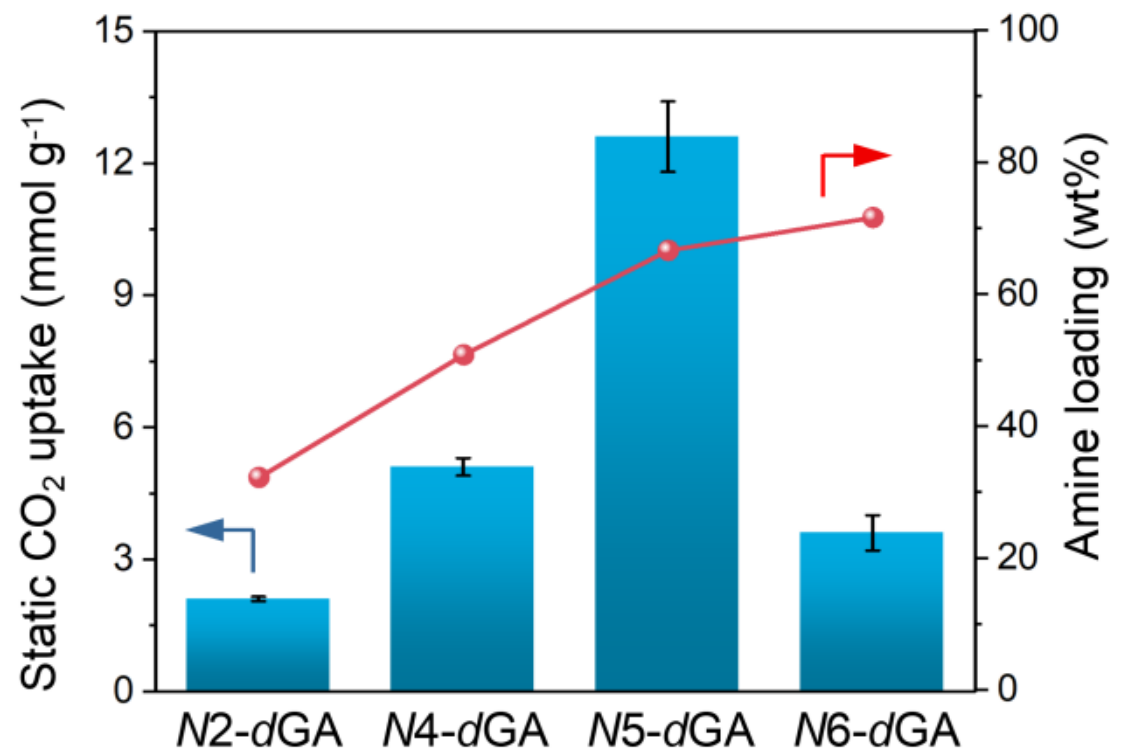

Figure S3: CO_2 uptakes and amine loadings of *N*-*d*GA under a pure CO_2 atmosphere. (25 °C, 1.0 bar)

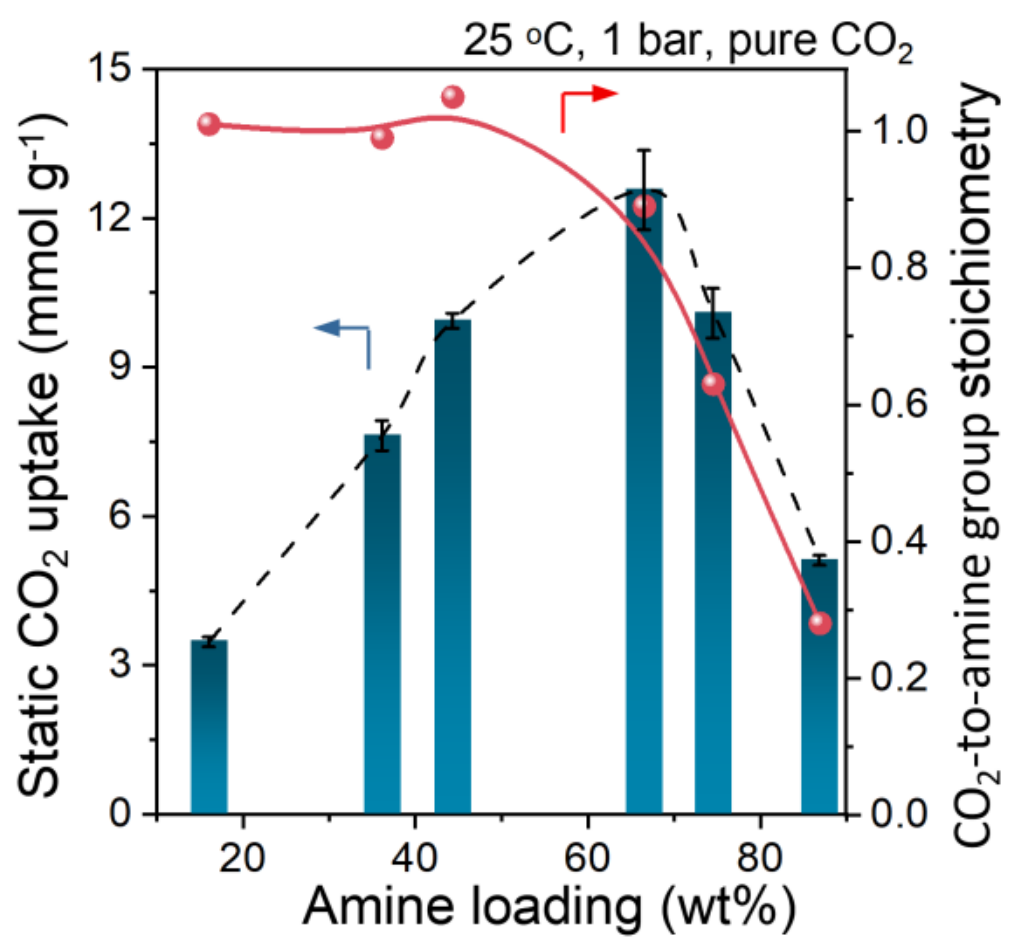

Figure S5: CO_2 uptakes and CO_2-to-amine group stoichiometry of *N*5-*d*GA with different amine loadings.

16. Does the use of water bubblers change the CO2 concentration in the gas phase?

Response: In our experiments, the CO_2 concentration in the gas phase is directly measured before every DAC test using a GC9790II gas chromatograph with a calibrated CO_2 standard curve. The CO_2 standard curve is updated quarterly. The gas stream is adjusted and verified to contain exactly 400 ppm CO_2 prior to exposure to the sorbent whether dry or humidified via water bubbler.

17. What is dual-amine, tetra-amine?

Response: The terms "dual-amine," "tetra-amine," etc., are shorthand notations to denote linear polyethyleneimines with increasing numbers of amine groups. Similar abbreviations are widely used in literature(Eugene J. K. et al. Cooperative carbon capture and steam regeneration with tetraamine-appended metal–organic frameworks. *Science* 369, 392-396 (2020); Yang, S. et al. Mesh-like structure integrated core-shell-shell nanocomposites for enhanced stability and performance in carbon capture. *Nature Communications* 16, 10526, (2025); Waheed, A. & Baig, U., Exploiting phase inversion for penta-amine impregnation of ultrafiltration support matrix for rapid fabrication of a hyper-cross-linked polyamide membrane for organic solvent nanofiltration. *Process Safety and Environmental Protection* 169, 24-33, (2023)). All organic amines used in this work are determined as follows: *dual*-amine,

ethylenediamine; *tetra*-amine, triethylenetetramine; *penta*-amine, tetraethylenepentamine; *hex*-amine, pentaethylenehexamine. The relevant content has been updated in page 16, "Preparation of samples" section of the revised manuscript.

18. There is no useful discussion of kinetic analysis.

Response: Thank you for your suggestion. To analyze the kinetics based on breakthrough adsorption curves, we fitted the experimental data using the Thomas model, which accounts for both external mass transfer and intraparticle diffusion in fixed-bed column (Chu, K. H. Fixed bed sorption: Setting the record straight on the Bohart-Adams and Thomas models. *J. Hazard. Mater.* **177**, 1006-1012, (2010)). Breakthrough tests were conducted at a gas velocity of 1 m s^{-1}, simulating high-flow industrial applications. Tests were performed with CO_2/N_2 mixtures (0.04/99.96) at 25°C under either dry (0% RH) or humid conditions (80% RH).

The kinetic studies can be found in **Figure** S8, and the high correlation coefficients ($R^2 > 0.99$) confirm an excellent fit of the model. the results demonstrate that the CO_2 adsorption rate constant is 12% lower in humid air than in dry air. This behavior is consistent with the mass transfer process of water-mediated CO_2 adsorption on amine-functionalized sorbents. As reported by Hahn et al. (Hahn, M. W., Steib, M., Jentys, A. & Lercher, J. A. Mechanism and kinetics of CO_2 adsorption on surface bonded amines. *J. Phys. Chem. C* **119**, 4126-4135, (2015)), a thin water film forms on the sorbent surface at high relative humidity, limiting the interaction of CO_2 with the active amine sites and constraining the rates of adsorption.

The relevant content has been updated on page 7 of the revised manuscript, and **Figure** S8 of the Supplementary Information.

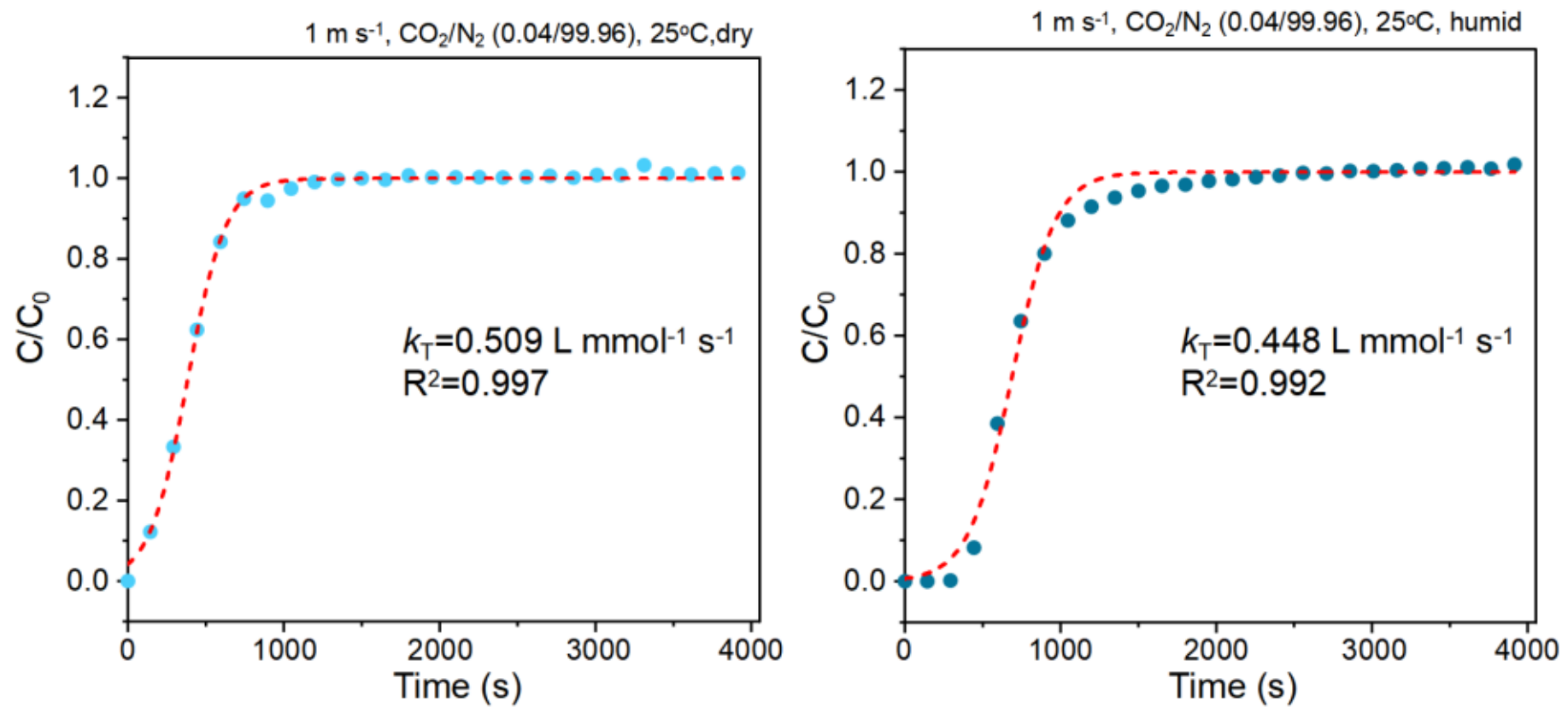

Figure S8: CO_2 adsorption rate constant of *N*5-*d*GA under dry and humid conditions. Dashed lines represent the breakthrough curves fitted using the Thomas model.

19. The diameter of GO-aerogel shown in Figure 2 is 5 mm. In this case, what is the penetration depth of Ar plasma into GO structure? What is the defect concentration distribution along the radial direction?

Response: In general, for typical macroporous materials with a porosity greater than 90%, the penetration depth of low-pressure capacitively coupled plasma ranges roughly from 1.6 to 4 mm (Marxen, M. L., et al. On the plasma permeability of highly porous ceramic framework materials using polymers as marker materials. *Plasma Processes and Polymers* 20, 2200118, (2022)). The ultra-high porosity of graphene aerogel (>99%) enables deep diffusion of Ar plasma species. To assess plasma penetration depth and radial defect distribution, we performed Raman spectroscopy on both the surface and core of the 5 mm-diameter *d*GA (**Figure** R2). The I_D/I_G ratios are 0.59 (surface) and 0.61 (core), indicating that the defect density of the surface and core is almost identical. This suggests that Ar plasma effectively penetrates throughout the entire sample, with no significant gradient in structural modification.

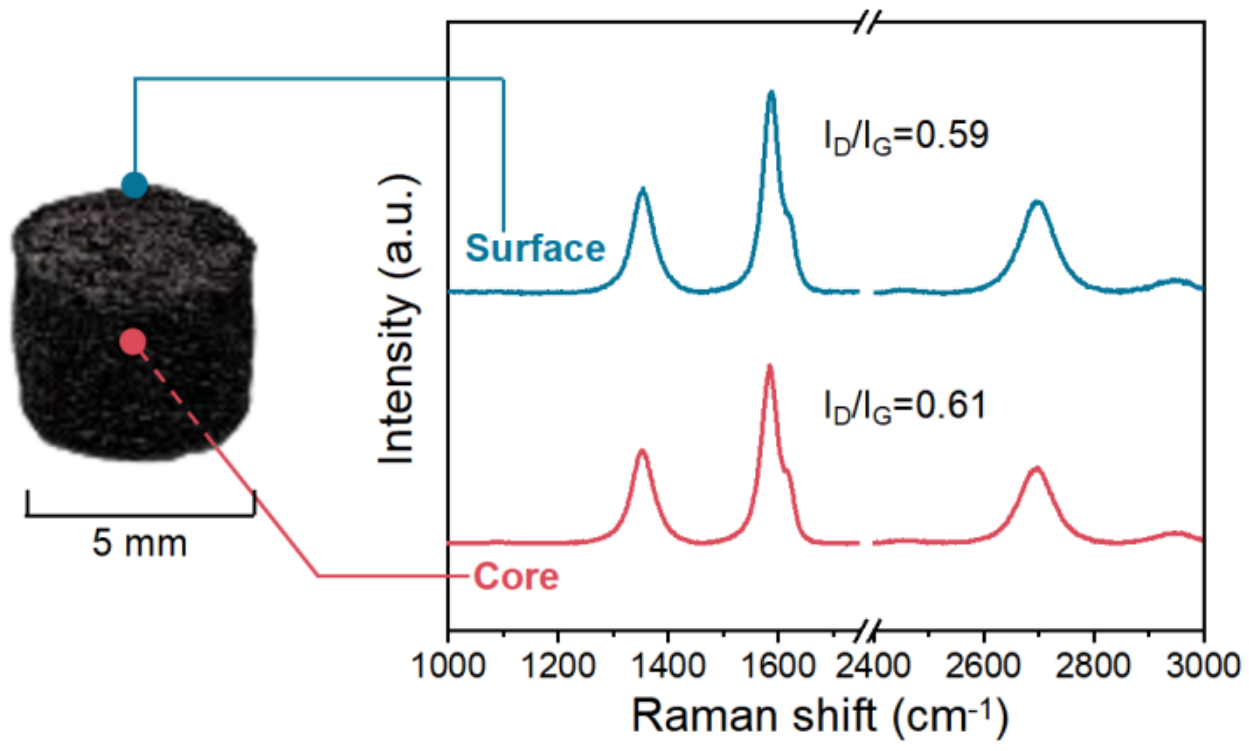

Figure R2: Raman spectroscopy of the surface and core *d*GA after 30 min Ar plasma treatment.

20. Introducing a defect into a graphene structure will increase the resistance of the graphene composite. What were the resistances of GO gel before and after defect/amine functionalization? How does the author rationalize the conductivity of the defective graphene? (see comment 11)

Response: We supplemented the electrical conductivity measurements of the various materials packed into the breakthrough column (inner diameter: 4.6 mm; length: 60 mm)—the same configuration used for adsorption and electrothermal desorption—with additional measurements. The results show that introducing point defects reduces the conductivity of the graphene aerogel from 969 S m^{-1} to 649 S m^{-1}, which is a decrease of around 33%. Subsequent amine functionalisation lowered the conductivity by a further 37%, yielding a value of 409 S m^{-1} for *N*5-*d*GA. The electrical conductivity of the *N*5-*d*GA remained stable at 409-443 S m^{-1} for currents ranging from 0.5 to 0.7 A. Although this conductivity is lower than that of carbon fibers (Lee, W. H. et al. Sorbent-coated carbon fibers for direct air capture using electrically driven temperature swing adsorption. Joule 7, 1241-1259 (2023); Lee, Y. H. et al. Design of electrified fiber sorbents for direct air capture with electrically-driven temperature vacuum swing adsorption. Adv. Mater., e04542, (2025)), it enables efficient electrothermal regeneration at a significantly lower temperature (80 °C) compared to typical systems requiring >100 °C. Consequently, the lower operating temperature more than compensates for the conductivity, resulting in a reduced current demand and lower overall energy consumption (As shown in **Table** R1 in comment 11). The relevant

content has been updated on page 7 of the revised manuscript, as well as **Figure** S9 of the Supplementary Information.

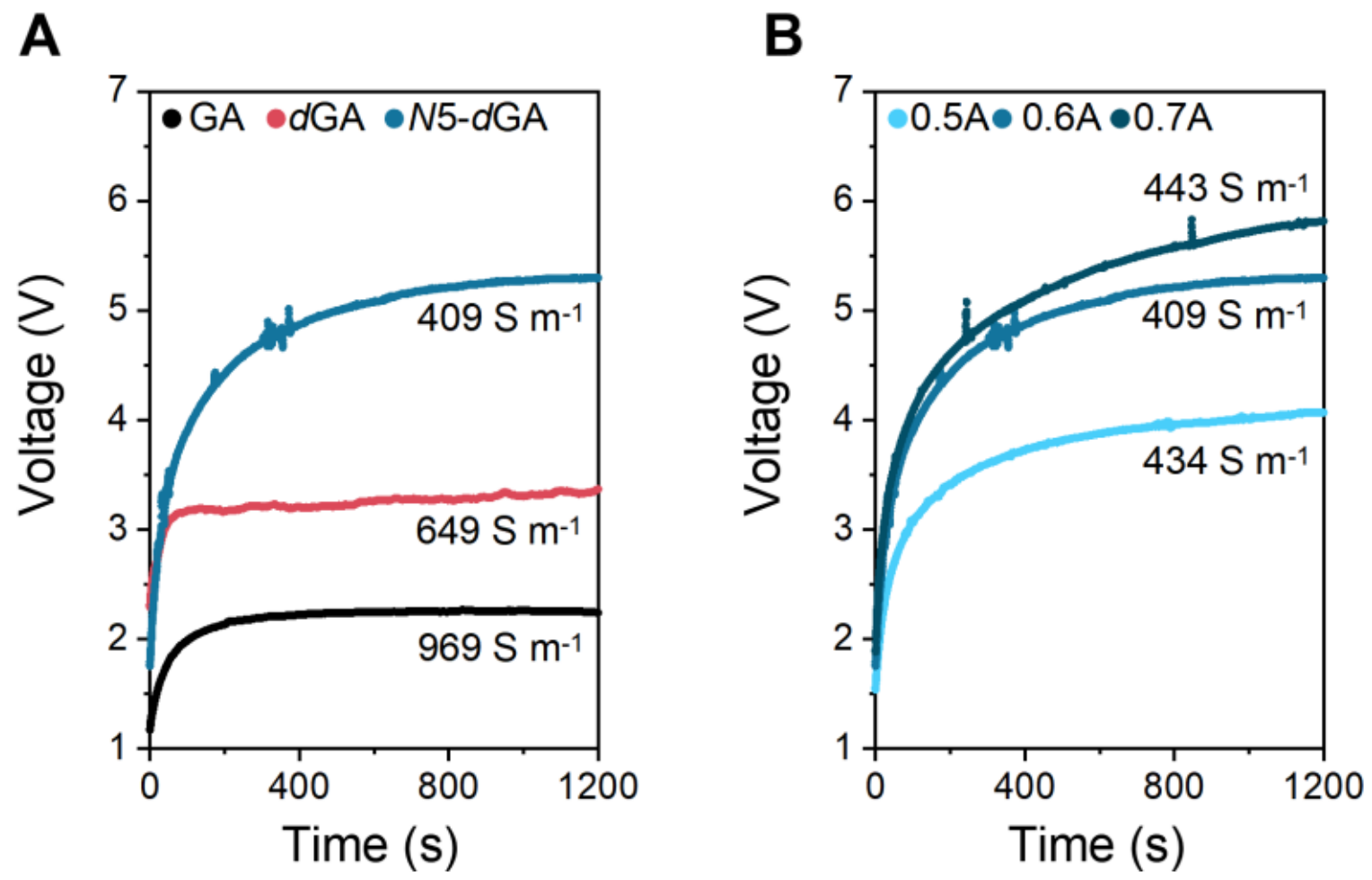

Figure S9: (A) The electrical conductivity of GA, *d*GA and *N*5-*d*GA packed into a quartz breakthrough column. I=0.6 A. (B) The electrical conductivity of *N*5-*d*GA packed into a quartz breakthrough column at different current intensities.

Table R1: A comparison of current intensity for *N*5-*d*GA and reported Joule heating-enabled sorbents to desorb CO_2.

Name	Desorption temperature (℃)	Current intensity (A)	Heat duty for regeneration (GJ t^{-1})	Ref.
PEI-carbon fiber	90~110	0.8~1.0	7.2	Lee, et al. (2023)
Ag-NC coated fiber	110	1.9	6.1	Lee, et al. (2025)
*N*5-*d*GA	80	0.6	3.4	This work

21. Lines 142-3: How did you measure porosity? What kind of porosity does it mean? Mesopore porosity or Macropore porosity?

Response: The porosity of graphene aerogels is conventionally calculated from bulk and skeletal densities using the formula (Sun, H., Xu, Z., & Gao, C. Multifunctional, ultra-flyweight, synergistically assembled carbon aerogels. *Advanced Materials*, 25, 2554-2560, (2013); Xu, X., et al. Double-negative-index ceramic aerogels for thermal superinsulation. *Science*, 363, 723-727, (2019)):

$$porosity = (1 - \frac{\rho_{\text{bulk}}}{\rho_{\text{skeletal}}}) \times 100\%$$

In our work, the ρ_{bulk} of *N*5-*d*GA was measured to be 27 mg cm^{-3}. The skeletal density, accounting for the composite density of graphene and *penta*-amine, was estimated as 1420 mg cm^{-3} based on its mass ratio. Therefore, the porosity of *N*5-*d*GA is 98.1%. Scanning electron microscopy (Figure S24) reveals an hyperboloid macroporous network with pore sizes of approximately 50 μm, consistent with gas-bubble templating during hydroplastic foaming. It matches that of the pristine graphene aerogel reported in our earlier work (Pang, K. et al. Hydroplastic foaming of graphene aerogels and artificially intelligent tactile sensors. *Science Advances* 6, eabd4045, (2020)), confirming that amine functionalization does not significantly alter either the overall porosity or the macroporous structure.

The relevant content has been updated on page 17, "Characterization" section of the revised manuscript.

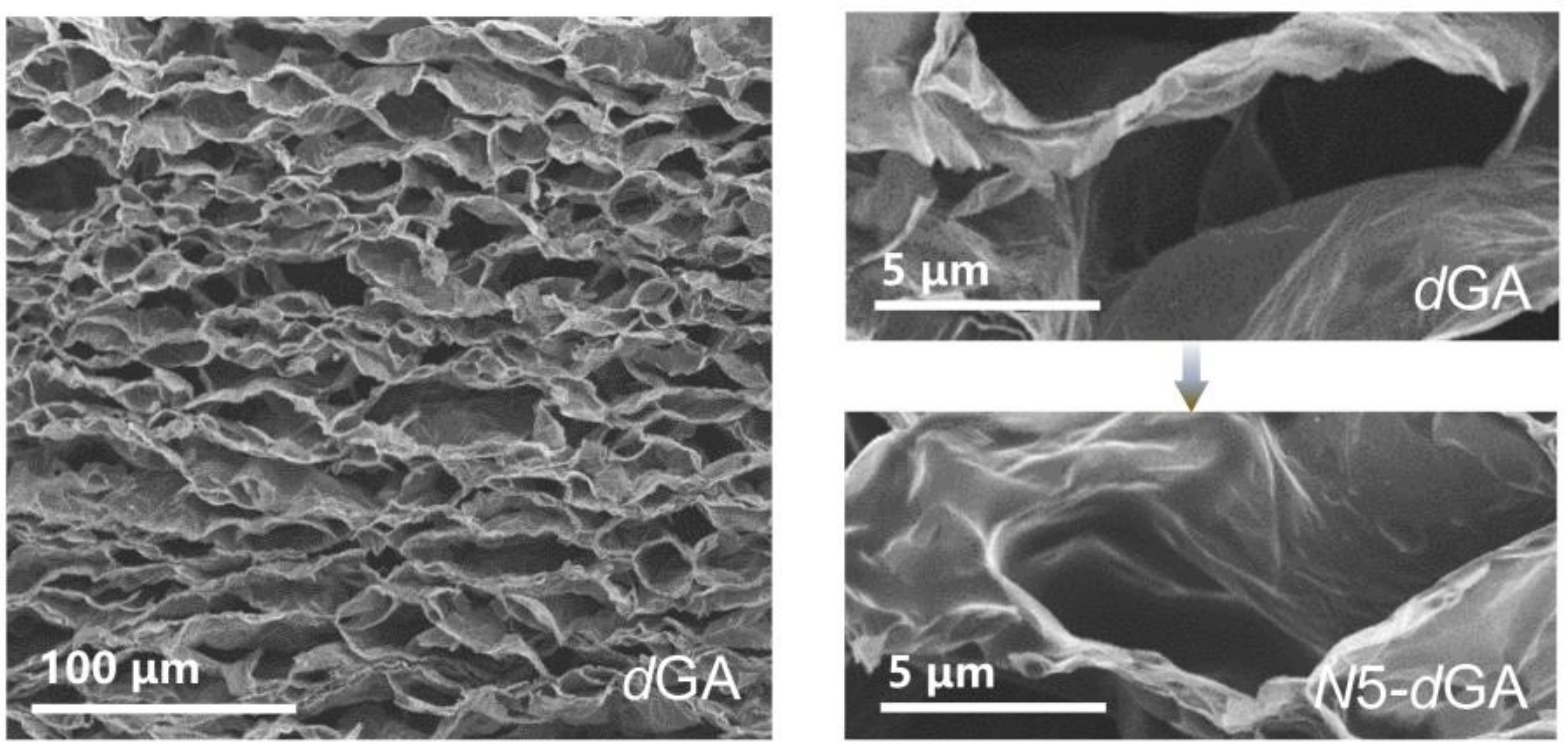

Figure S24: FE-SEM images showing the high porosity and hyperboloid structure of *N*5-*d*GA after *penta*-amine grafting.

22. If amines form layers in between graphene layers, then most of the defects will not interact with amines. What is the other evidence for one-step Zwitterion formation by defect-proton transfer to amine?

Response: The result of Raman mapping in **Figure** S30 shows that the I_D/I_G ratio increases from ~0.45 (pristine GA) to ~0.60 following point defect engineering. It then decreases to ~0.55 upon *penta*-amine grafting, yet remains significantly higher than pristine GA. This confirms that a substantial population of defects remains accessible for interaction with amines. Moreover, DFT calculations demonstrate that the proton traps can be rapidly regenerated under the influence of an electric field, indicating their

high suitability for the ESA process. **Figure** 2C shows that the presence of an electric field can effectively reduce the energy barrier of the dissociation of protons attached to point defects. The energy barrier of CO_2 desorption is reduced by 28.9% under an electric field intensity of 0.01 V nm^{-1} (**Figure** 2D).

The relevant content has been updated in **Figure** S29 of the Supplementary Information, and **Figures** 2C-2D of the revised manuscript.

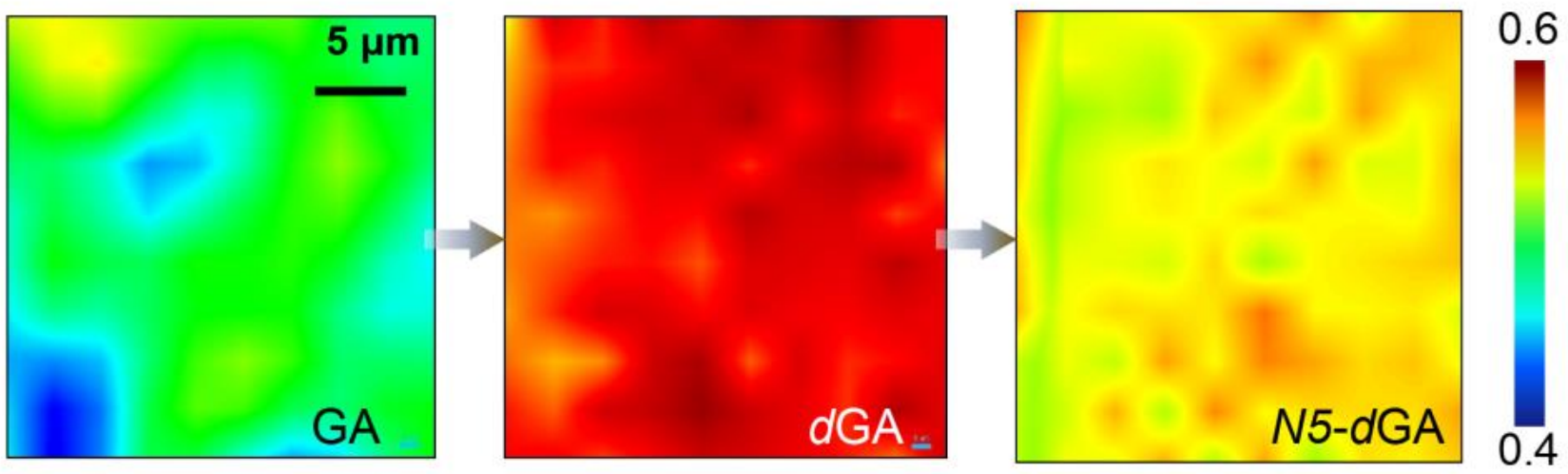

Figure S29: Raman mappings of overall I_D/I_G for samples (scanning area: 20 μm×20 μm).

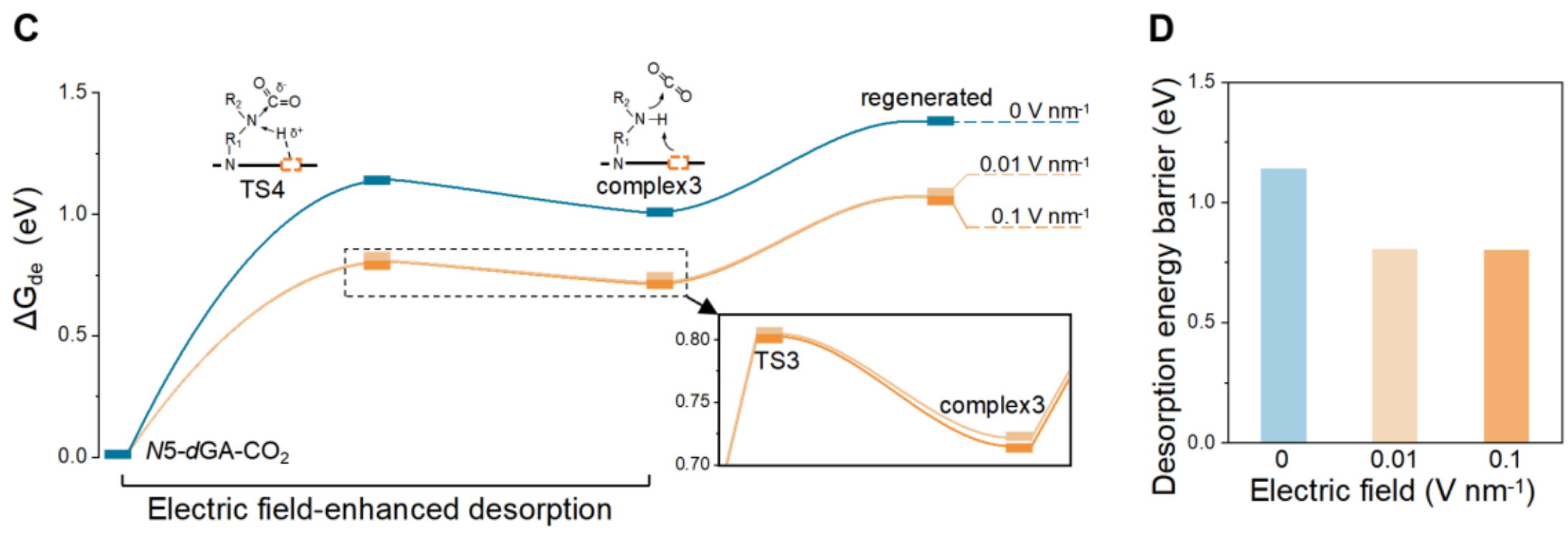

Extracted from Figure 2. (C) The CO_2 desorption pathway and (D) desorption energy barrier of *N*5-*d*GA under different electric field strengths.

23. Is there any evidence supporting that the transient Raman peak at 2348 cm-1 indicates defect-proton interaction such as calculation from the first principles? Could it be just CO2-amine interaction or by-products from the CO2-amine reactions?

Response: Thank you for your kind suggestion. To clarify the proton-trap mechanism, we have performed a detailed analysis of the Raman spectra (**Figure** 2E). Although the symmetric stretching vibration of COO^- (～1380 cm^{-1}) is obscured by the D band of graphene (1270~1450 cm^{-1}), several diagnostic spectral features are observed upon CO_2 adsorption. Specifically, the new peaks at 1570 cm^{-1} and 2348 cm^{-1} are assigned to CO_2-

amine interaction products (Hadjiivanov, K. I., et al. Power of infrared and Raman spectroscopies to characterize metalorganic frameworks and investigate their interaction with guest molecules. *Chemical Reviews*, 121, 1286-1424, (2021)). Additionally, a distinct C-H stretching peak develops at 2920 cm^{-1}, indicating protonation of the point defect site (Cançado, L. G., et al. Quantifying defects in graphene via raman spectroscopy at different excitation energies. *Nano Letters*, 11, 3190-3196, (2011); Elias, D. C., et al. Control of graphene's properties by reversible hydrogenation. *Science*, 323, 610-613, (2009)). These observations align closely with the DFT-predicted reaction pathway (**Figure** 2A). Notably, the CO_2-derived species and the C-H bond exhibit concerted emergence during adsorption and synchronous disappearance during regeneration, respectively. This coupled phenomenon confirms the reversible activation of point defect-based proton traps, providing the direct experimental evidence for the one-step mechanism in *N*5-*d*GA.

The relevant content has been updated on page 7 and **Figure** 2 of the revised manuscript.

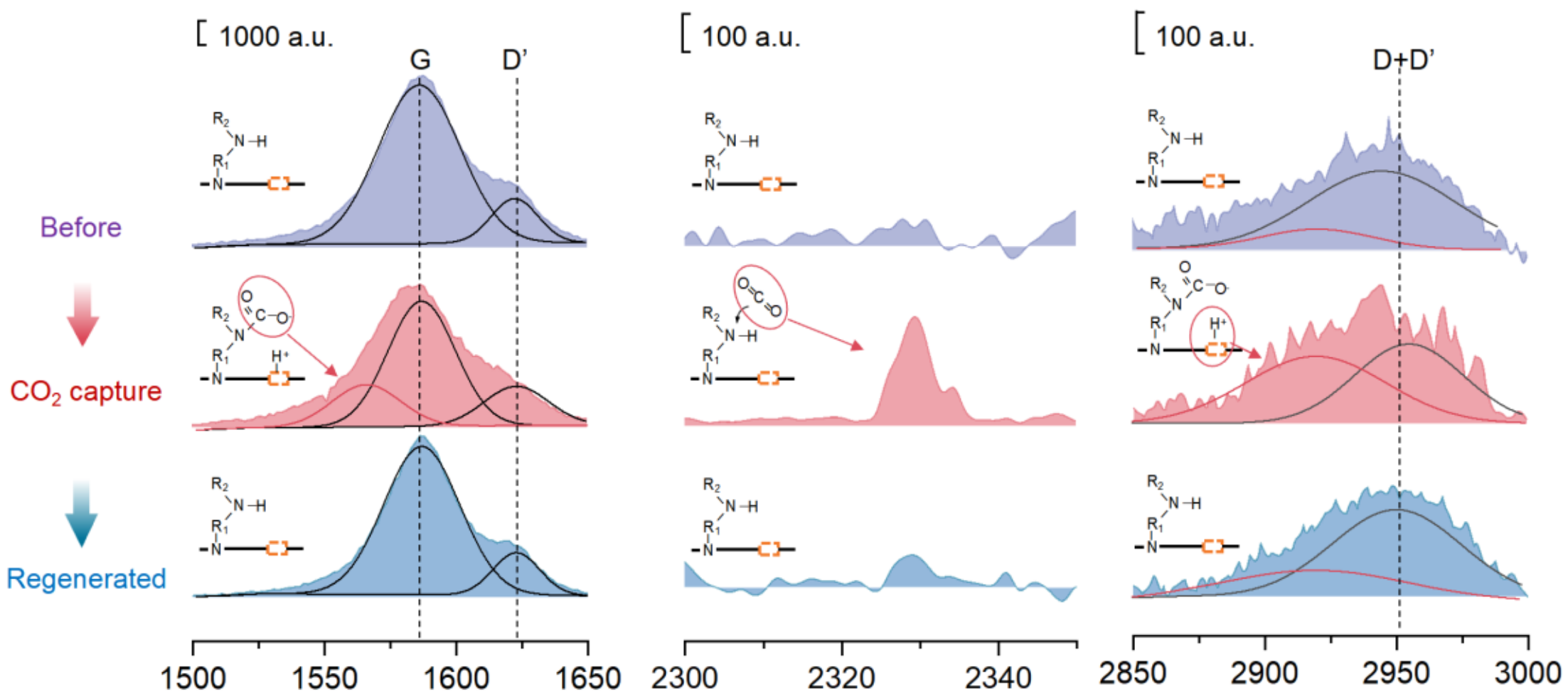

Figure 2E. Amine-defect synergistic enhancement of proton transfer on *N*5-*d*GA revealed using the Raman spectra.

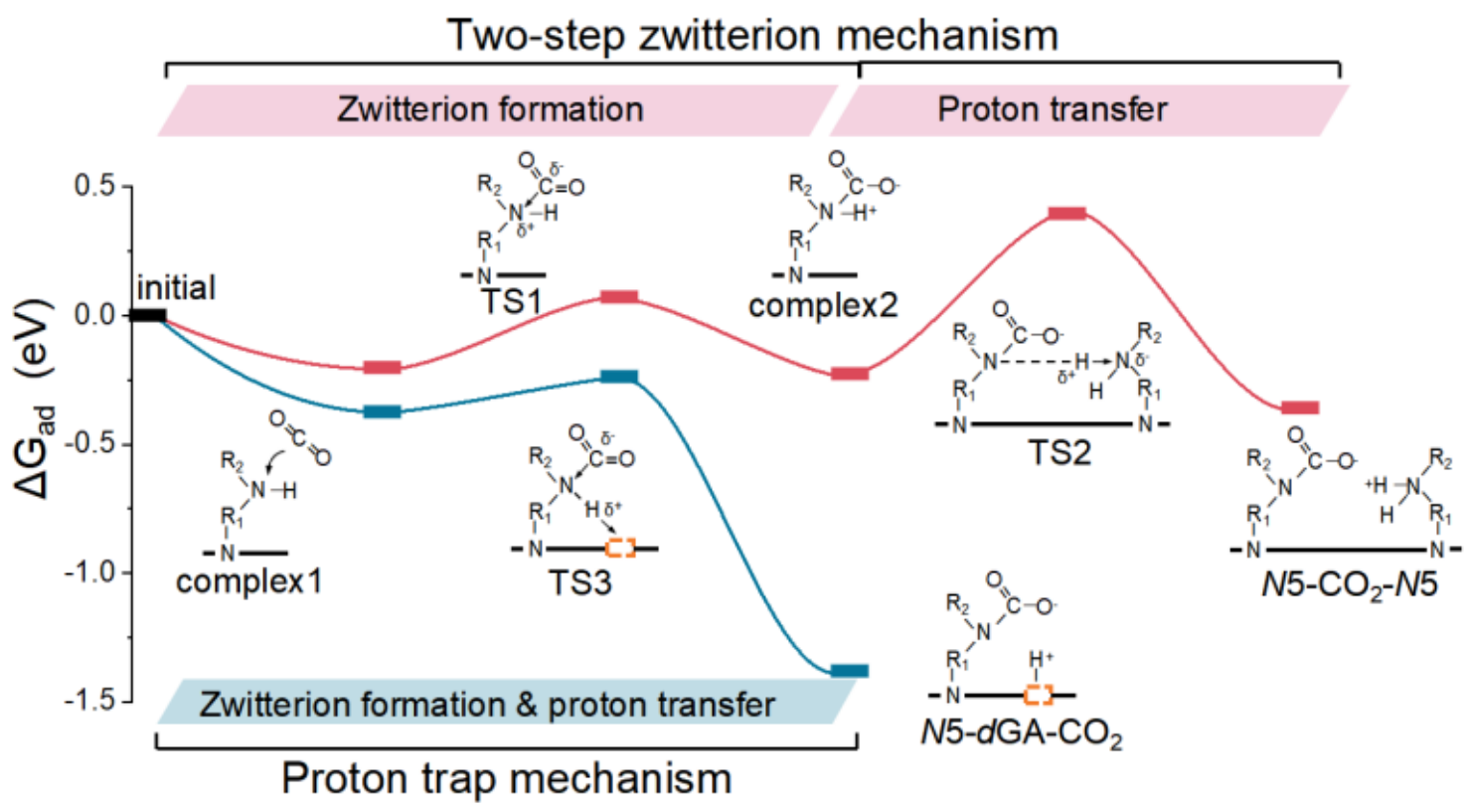

Figure 2A. The CO_2 adsorption pathway of *N*5-*d*GA via two-step zwitterion mechanism and proton strap mechanism. The orange dashed box represents a point defect on *d*GA.

24. Overall, experimental information and support for ESA are very, very weak. It also seems like the sorbent for the high-flux system, and the ESA module look different. Why is that?

Response: The design of an ESA module is fundamentally dictated by the morphology of the material. Current reported ESA modules rely on carbon fiber-based sorbent (Lee, W. H. et al. Sorbent-coated carbon fibers for direct air capture using electrically driven temperature swing adsorption. Joule 7, 1241-1259, (2023); Lee, Y. H. et al. Design of electrified fiber sorbents for direct air capture with electrically-driven temperature vacuum swing adsorption. Adv. Mater., e04542, (2025)), which cannot be processed into particulate form or packed as conventional adsorbent beds. In contrast, our *N*5-*d*GA is a mechanically robust bulk material that can be readily fragmented and packed directly into a standard fixed-bed column. Consequently, our ESA module closely resembles conventional adsorption beds in both appearance and configuration, with no essential structural distinction (**Figure** 3B).

To further strengthen the experimental foundation for ESA application, we have added competitive adsorption analysis (**Figure** 3E-G), regeneration energy evaluation (**Figure** 4A), long-term cycling stability over multiple adsorption-desorption cycles (**Figure** 4B-C), kinetics analysis (**Figure** S8), electrical conductivity characterization (**Figure** S9-S10), electrothermal heating profiles (**Figure** S11), and a dedicated section

on the “Pathway to Economic Viability”, which includes a detailed cost-benefit analysis of *N*5-*d*GA for practical ESA-DAC deployment. We believe that these additions comprehensively address the engineering feasibility and performance robustness of our system.

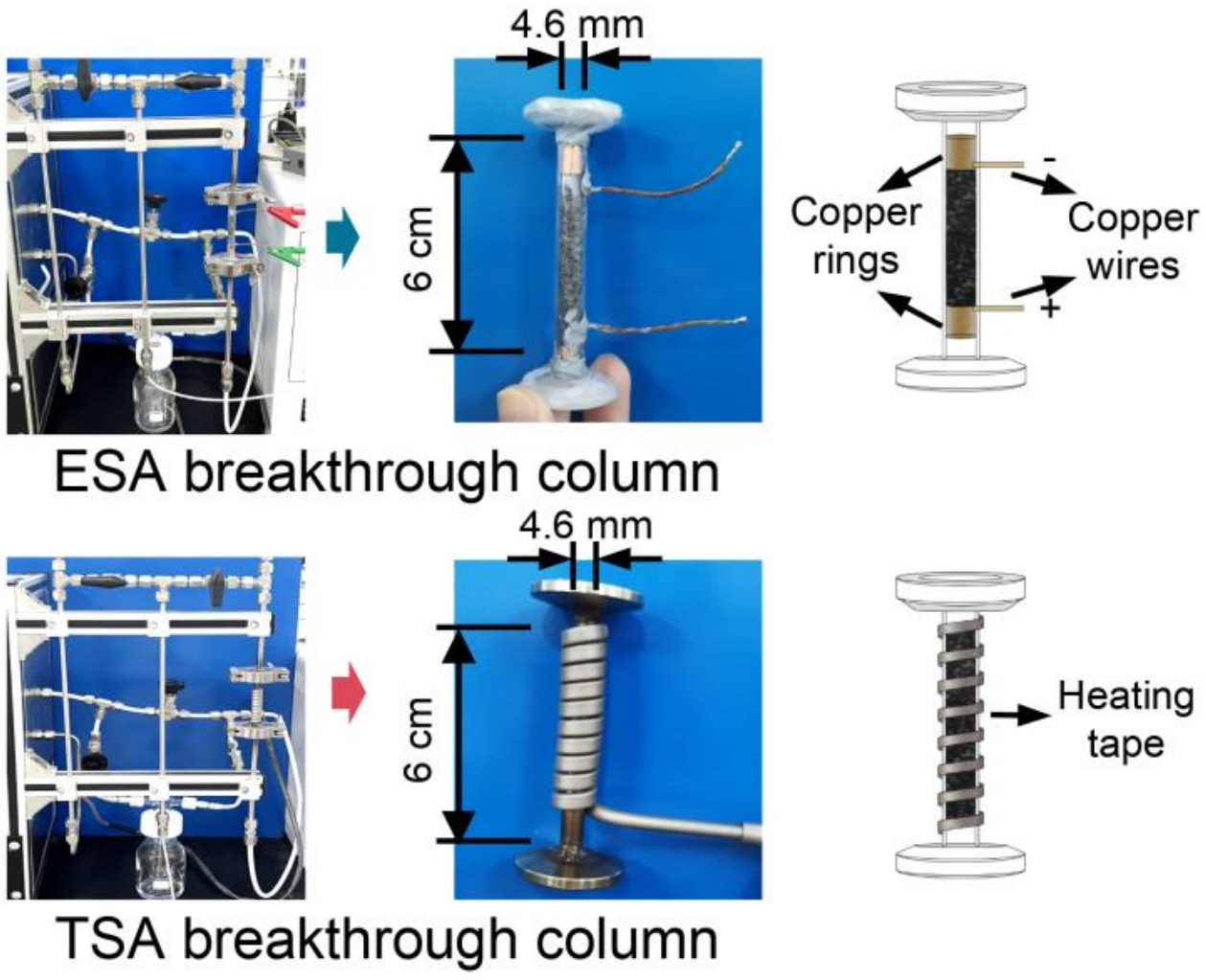

Figure 3B. The breakthrough columns for ESA and TSA.

25. In the ESA part, the applied voltage is another important parameter, but the information cannot be found in the manuscript.

Response: We agree with your suggestion. Figure S9 shows the voltage and related electrical properties applied to the *N*5-*d*GA packed into the breakthrough column with an inner diameter of 4.6 mm and a length of 6 cm during electrothermal desorption. In our experiments, a constant current of 0.6 A was applied for electrothermal desorption of *N*5-*d*GA, resulting in a voltage of 5.3 V. The electrical conductivity of the *N*5-*d*GA remained stable at 409-443 S m^{-1} for currents ranging from 0.5 to 0.7 A.

The relevant content has been updated in **Figure** S9 of the Supplementary Information.

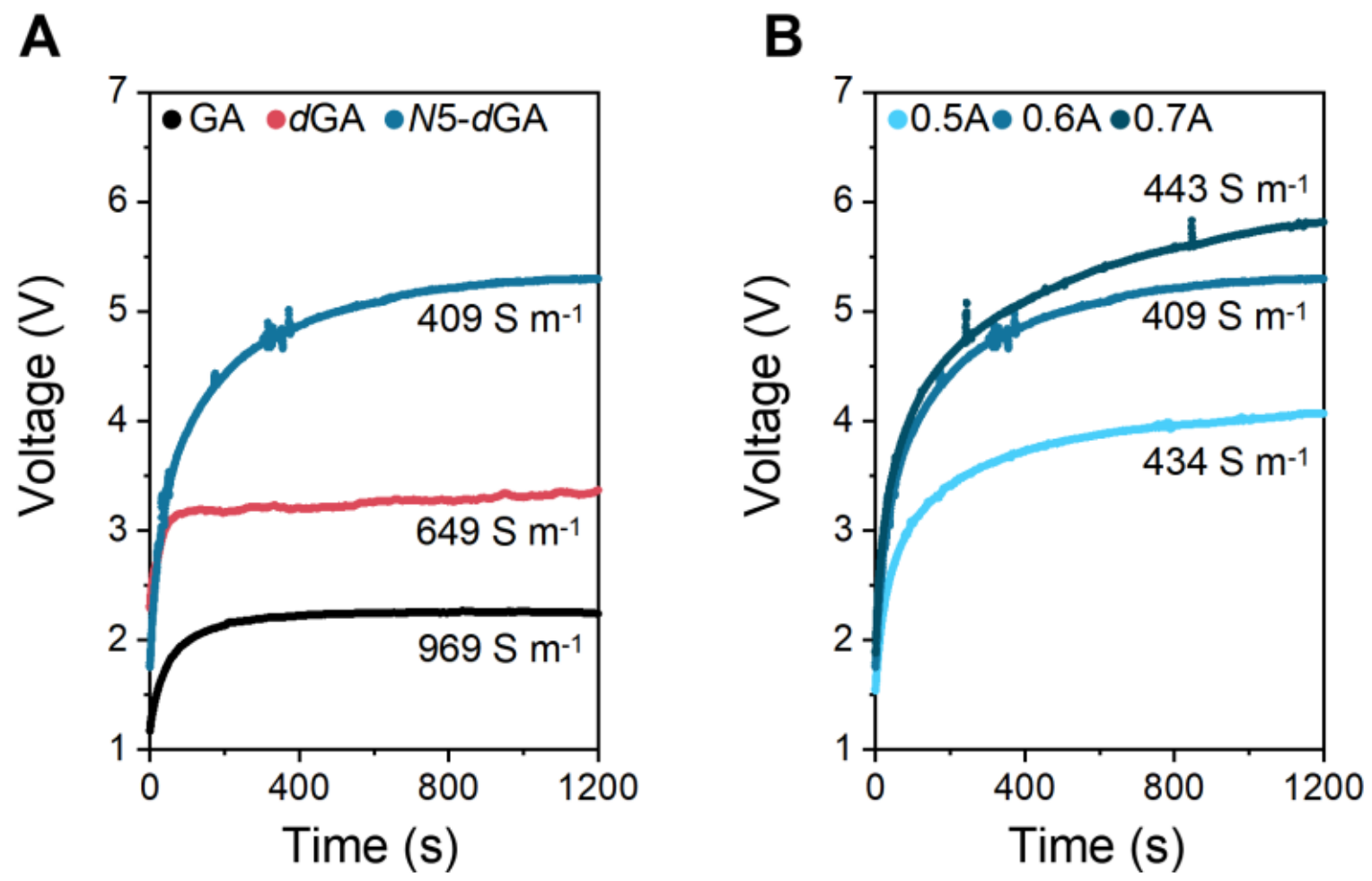

Figure S9: (A) The electrical conductivity of GA, *d*GA and *N*5-*d*GA packed into a quartz breakthrough column. I=0.6 A. (B) The electrical conductivity of *N*5-*d*GA packed into a quartz breakthrough column at different current intensities.

26. Supporting Figure 44 is too small to examine. Also, it seems like the author used a pile of rectangular-shaped sorbents for ESA. With this set up, ohmic contact can be poor and increase the resistance to very high values. The authors need to show the resistance of the single ESA sorbent and a pile of ESA sorbents along with the temperature profile of the whole ESA module during desorption.

Response: In fact, it has been reported in the literature that the electrical conductivity of graphene aerogels increases significantly upon compression due to the formation of additional contact points between fragmented sheets (Yang M., et al. Biomimetic architectured graphene aerogel with exceptional strength and resilience. *ACS Nano* 11, 6817-6824, (2017); Marinho, B., et al. Electrical conductivity of compacts of graphene, multi-wall carbon nanotubes, carbon black, and graphite powder. *Powder Technology*, 221, 351-358, (2012); Holm, R., Electric Contacts, Springer, Verlag Berlin, (1967)). As illustrated in the enlarged schematic of the packed bed (**Figure** S10), the electrical conductivity of a single bulk *N*5-*d*GA under a constant current of 0.6 A is 217 S m^{-1}, whereas the *N*5-*d*GA packed into the breakthrough column exhibits a electrical conductivity of 409 S m^{-1}—nearly double that of the single bulk sorbent.

In addition, the uniform Joule heating behavior of the single bulk *N*5-*d*GA is shown in **Figures** S12-S14. Moreover, temperature profiles during electrothermal

desorption demonstrate that the heating temperatures of the packed bed are nearly identical to those of the bulk sorbent (**Figure** S11). The above results fully demonstrate the resistance of the single ESA sorbent and a pile of ESA sorbents along with the temperature profile of the whole ESA module during desorption.

The relevant content has been updated in **Figures** S10-S14 of the Supplementary Information.

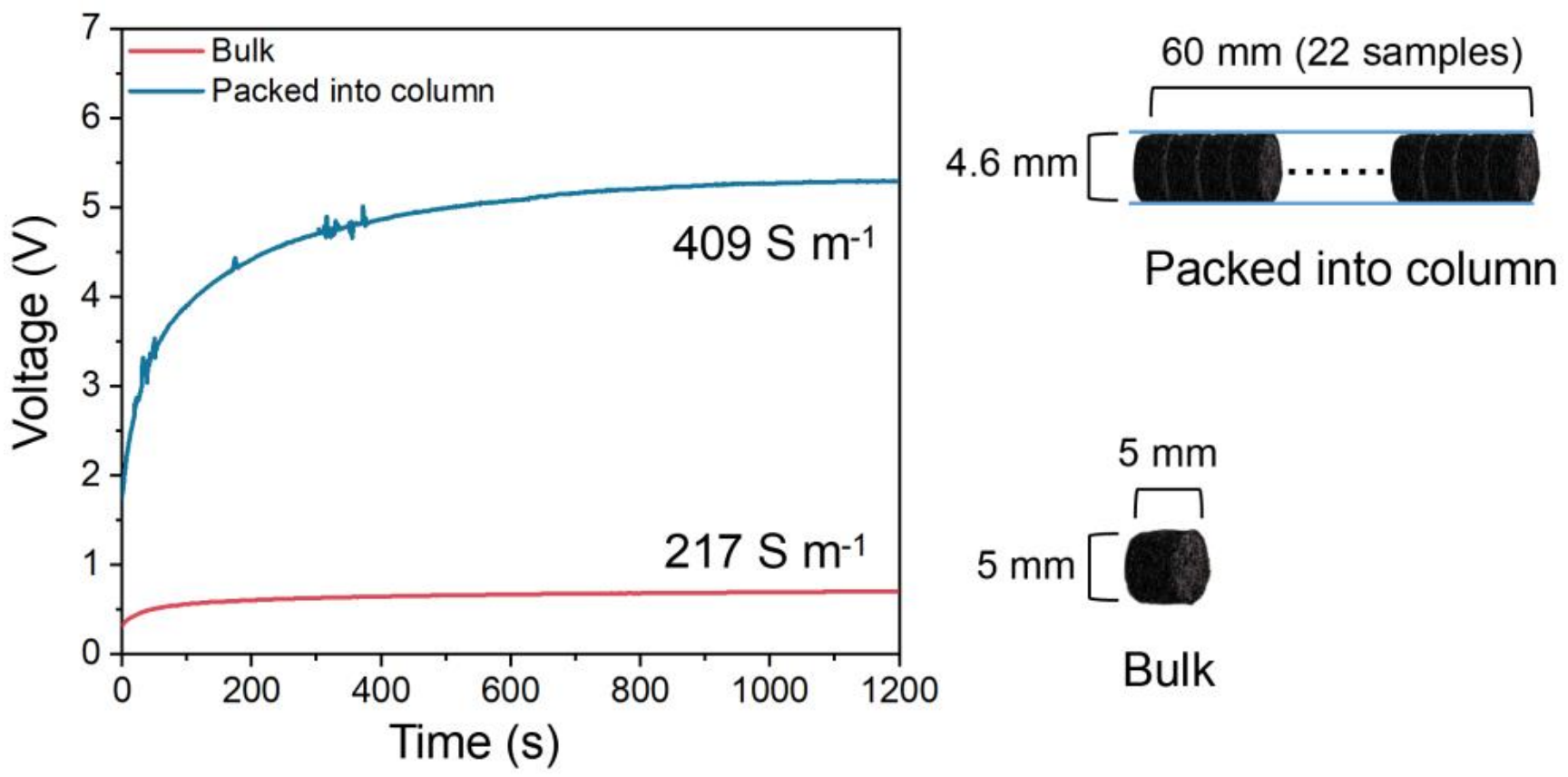

Figure S10. The electrical conductivity of single bulk *N*5-*d*GA and *N*5-*d*GA packed into a quartz breakthrough column. I=0.6 A.

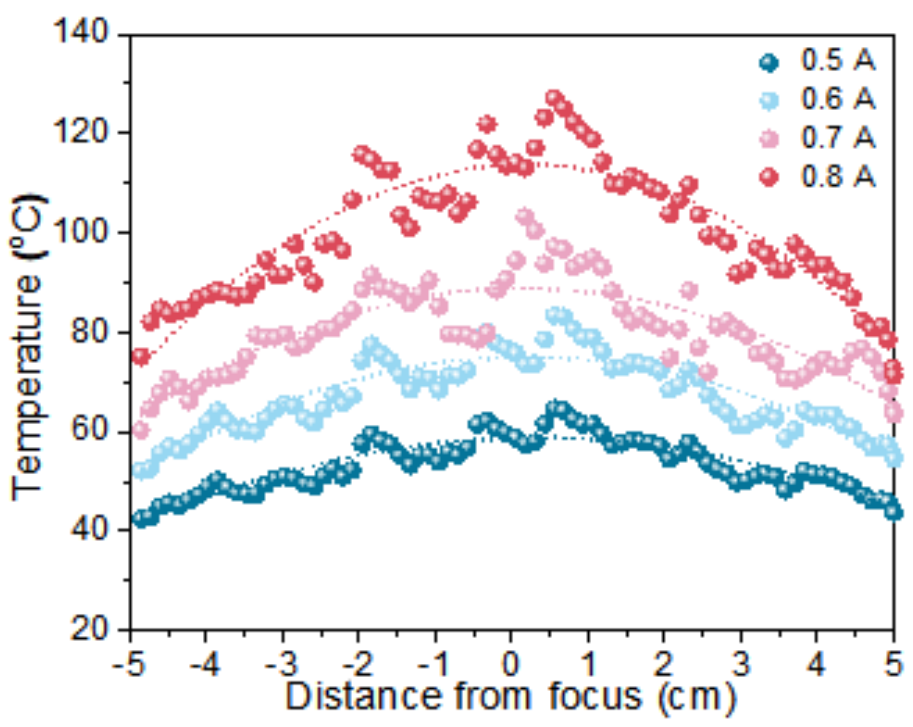

Figure S12: Distance-dependence temperature of focus point on bulk *N*5-*d*GA surface at different current intensities.

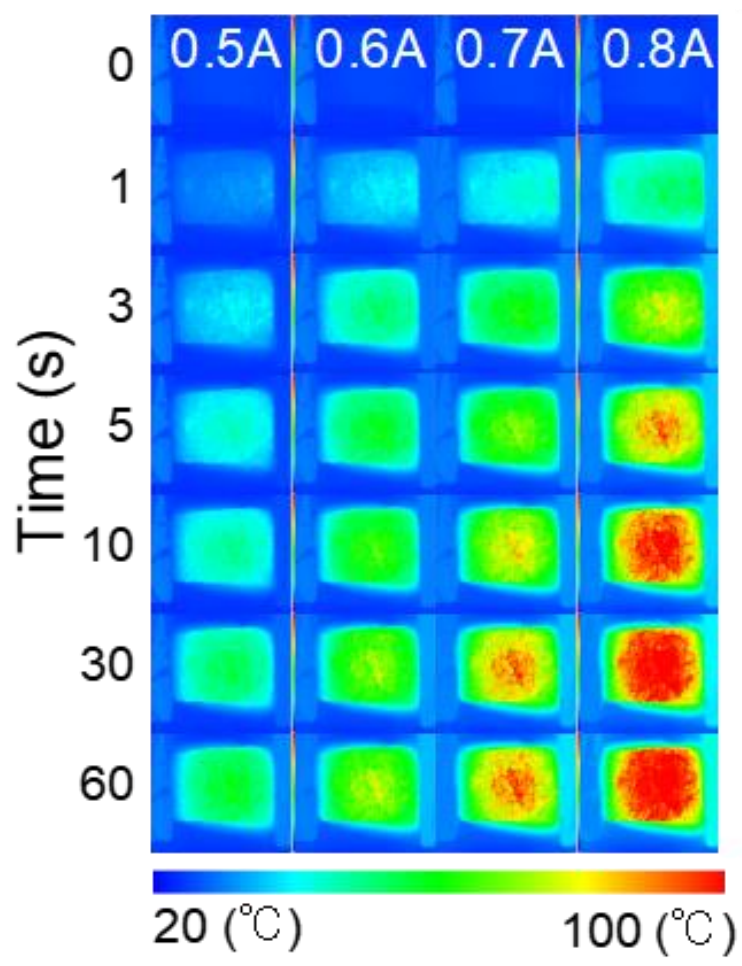

Figure S13: The thermal images of bulk *N*5-*d*GA at different current intensities.

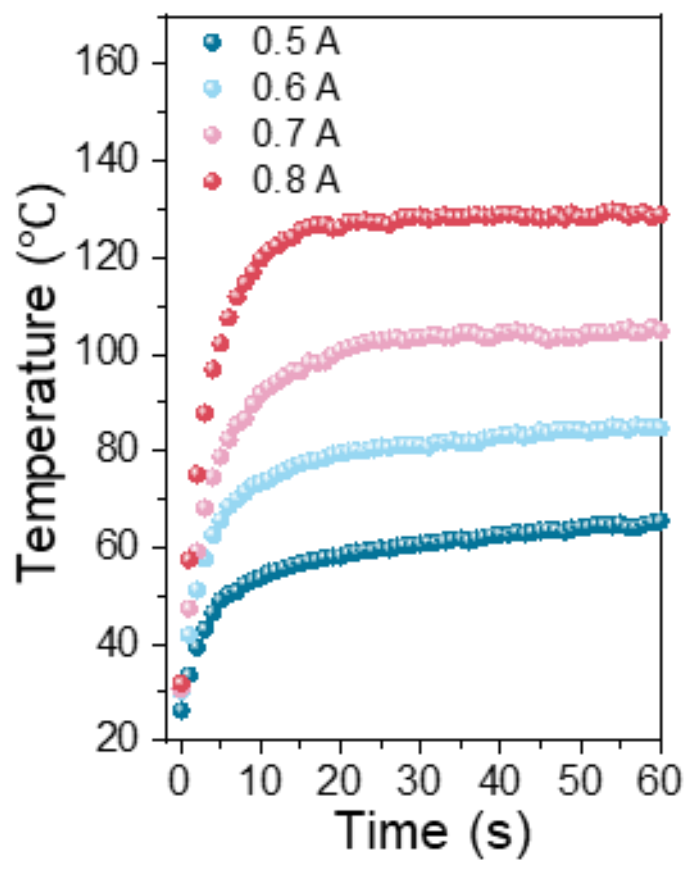

Figure S14: Time-dependence temperature curves of the bulk *N*5-*d*GA at different current intensities.

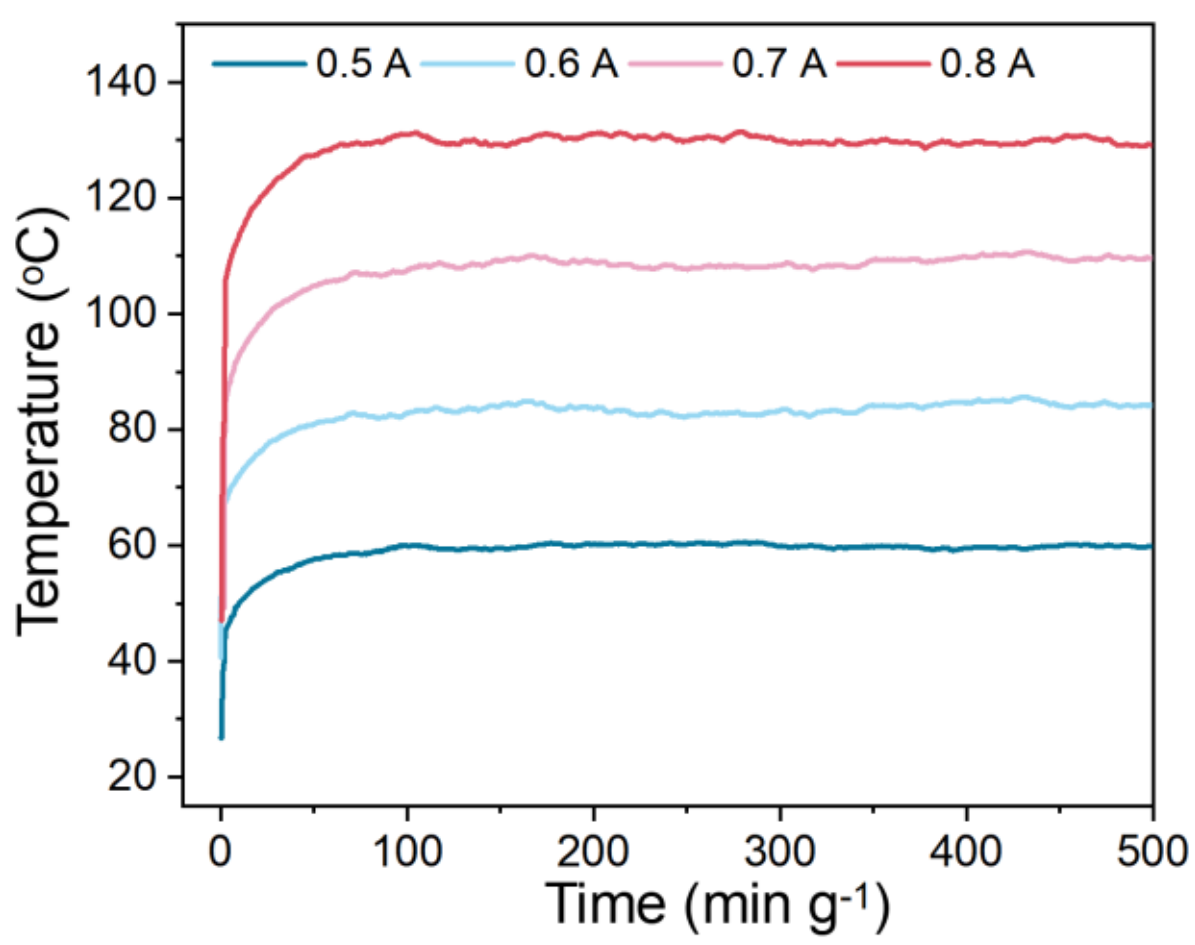

Figure S11: Time-dependence temperature curves of *N*5-*d*GA packed into a quartz breakthrough column at different current intensities.

27. With the rectangular shape sorbent, the gas flow rate of the outlet can be much lower than that of the inlet due to the significant pressure drop. The author needs to show the flow rates of the inlet and outlet.

Response: Thank you for the suggestion. We measured both the inlet and outlet flow rates during the breakthrough adsorption experiment and determined the pressure drop across the column using an OMEGA PX409-USBH pressure sensor. At a gas velocity of 1 m s^{-1}, the bed pressure drop is only 13 kPa m^{-1}. This value is significantly lower than that of conventional packed adsorption beds (~50 kPa m^{-1}) and is comparable to structured adsorption beds (Joannis, P., et al. Techno-economic analysis of packed bed and structured adsorbent for direct air capture. *Carbon Capture Science & Technology*, 17, 100518, (2025)). For our 6 cm-long bed, the total pressure drop is therefore only 0.78 kPa, which is negligible compared to the pressure head of the flow meter. Consequently, the gas velocity remains essentially unchanged after passing through the breakthrough column.

Typos: p1, line 36, "low" CO_2 concentration.

Response: We carefully checked the entire text to ensure that there were no typos.

Point-by-Point Response to Referees and Editors

Reviewer #4:

General comment: Overall, the authors have addressed most of Reviewer 1's concerns. They have undertaken substantial additional work and provided a useful set of new data. However, several of Reviewer 1's questions (notably Q1, Q6, and Q9) remain insufficiently addressed and would benefit from further clarification.

Response: Thank you for recognizing the substantial additional work and new data provided in the previous revision. We also appreciate the constructive comments regarding the remaining issues. In this revision, we have focused specifically on these points by adding targeted supplementary experiments, refining the relevant discussions, and incorporating additional analytical content where appropriate. Detailed point-by-point responses are provided below.

1. The nature of the defect sites and the interactions between amine groups, CO_2, and defects -which are central to the proposed proton trap mechanism-remain insufficiently clear and are not strongly supported by experimental evidence. At present, the only experimental indication of defect structure appears to be a single AC TEM image, while evidence for proton transfer relies on noisy and largely inconclusive Raman data around 2900 cm^{-1} (Figure 2e, right panel). Additional clarification and experimental support would substantially strengthen the manuscript. While the challenges associated with solid state NMR are understandably outlined, some experimental evidence beyond simulations and the limited experimental data currently presented would be desirable.

Response: We agree that additional evidence is needed to clarify the role of defect sites and the proton-transfer process in the proton-trap mechanism. To clarify the origin of the high-wavenumber Raman feature, we conducted DFT-based Raman simulations for *N*5-*d*GA before and after CO_2 adsorption. Raman spectra were simulated using the PAW-PBE method with Tkatchenko-Scheffler dispersion correction. As shown in Figure S8, CO_2 adsorption leads to an enhanced C-H stretching feature in the

2800~2900 cm^{-1} region. This result is consistent with the experimentally observed Raman change at 2920 cm^{-1} in Figure 2E, supporting the assignment of this feature to protonation of the point defect sites during CO_2 adsorption.

In addition, the result of electron paramagnetic resonance (EPR) spectroscopy further supports the involvement of point defects in the proton-transfer process. The EPR measurements were carried out at room temperature using a Bruker EMXplus-6/1 spectrometer. CO_2 adsorption and Joule heating regeneration were performed ex situ on the same sample. After CO_2 capture, the EPR signal at g≈2.003 is attenuated (Figure S9), indicating a decrease in point defect-related spin density (Just, S., et al. Preferential antiferromagnetic coupling of vacancies in graphene on SiO_2: Electron spin resonance and scanning tunneling spectroscopy. Phys. Rev. B, 90, 125449(2014)). After Joule heating regeneration, the EPR signal recovers to nearly its initial intensity. This attenuation and subsequent recovery are consistent with the reversible activation of point defect-based proton traps.

Together, these additional theoretical and experimental results strengthen the mechanistic basis of the proton-trap pathway and provide more direct support for the role of point defects in reversible proton transfer during CO_2 adsorption and regeneration. The relevant content has been updated on page 7, line 158-167, page 17, lines 472-473, and page 18, lines 503-506 of the revised manuscript, as well as Figures S8-S9 of the Supplementary Information.

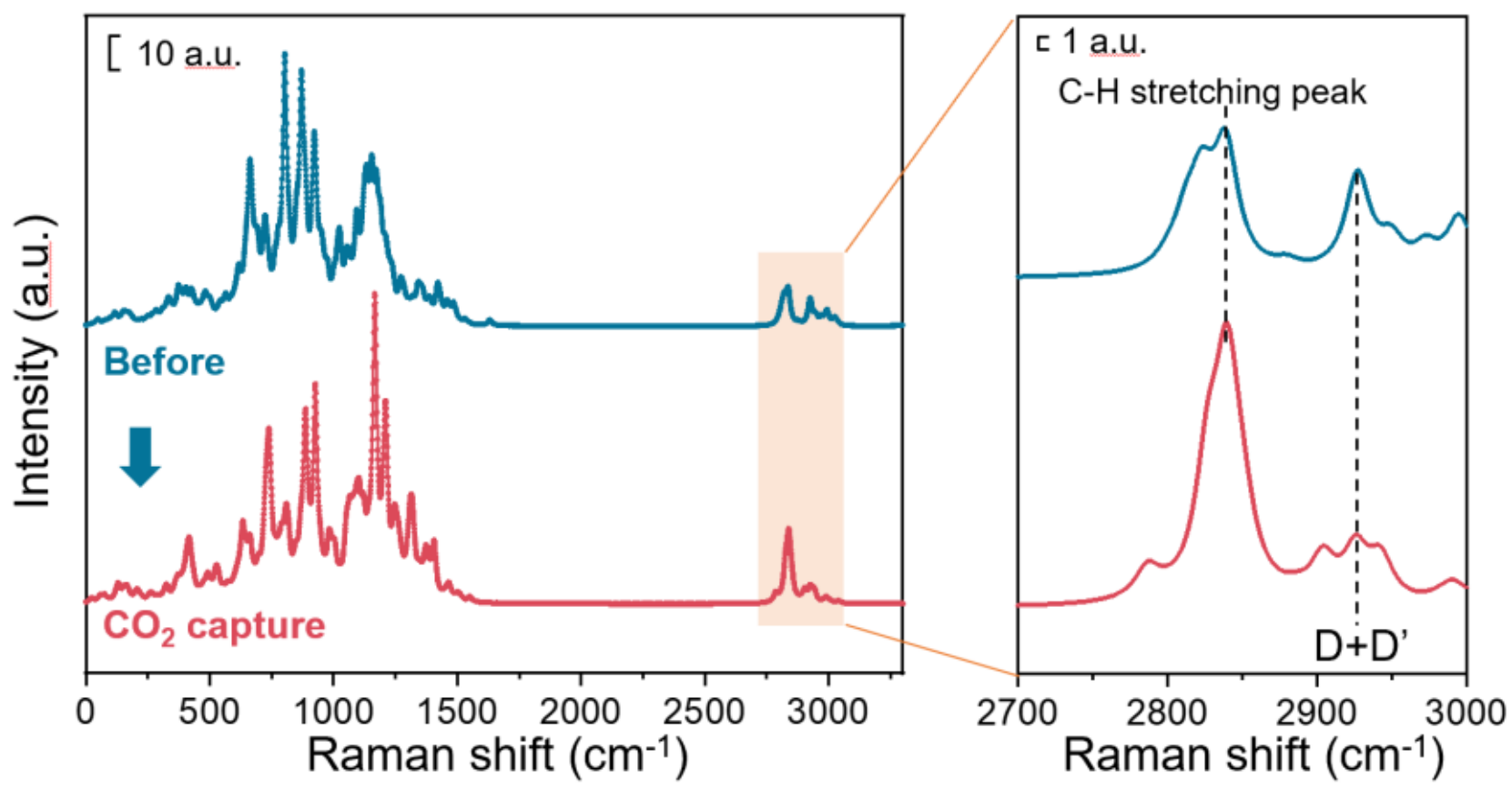

Figure S8: DFT-simulated Raman spectra of *N*5-*d*GA before and after CO_2 capture.

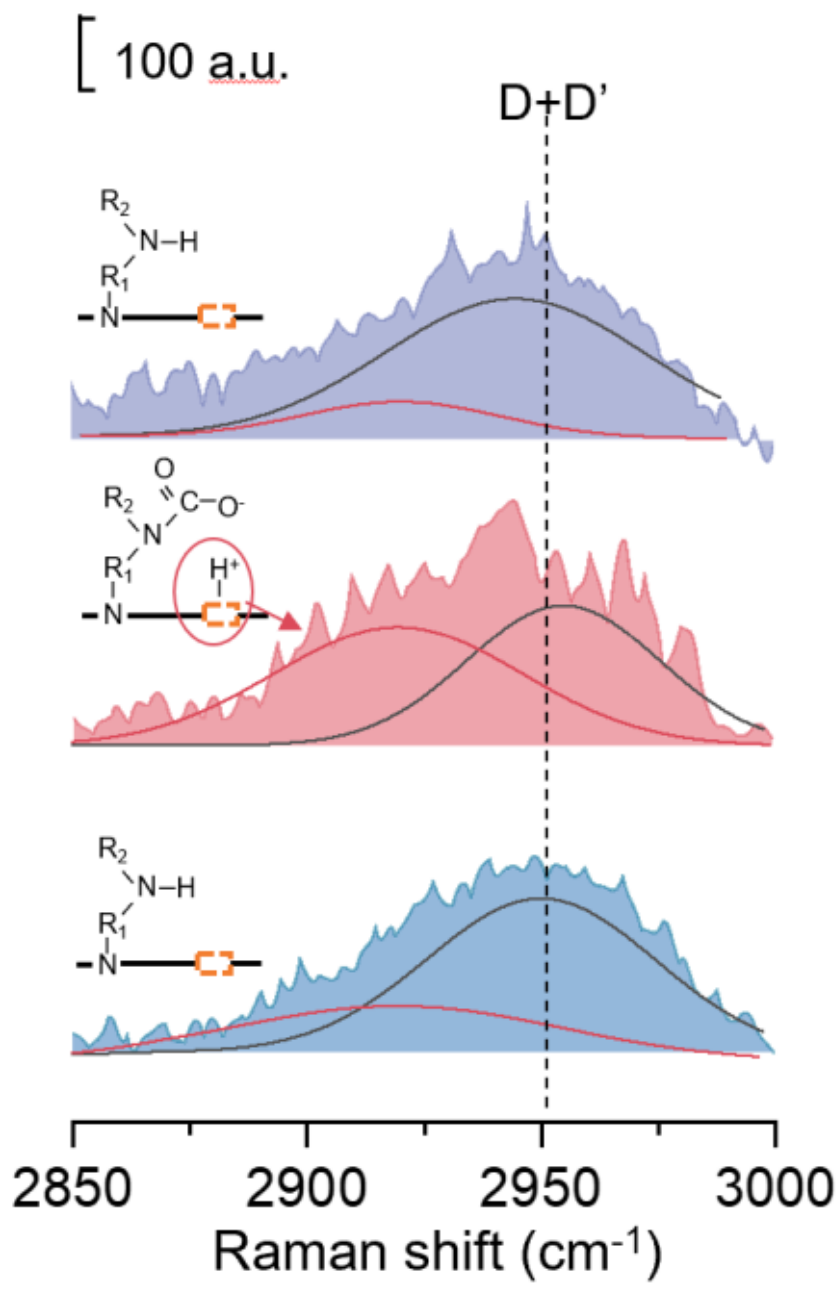

Extracted from Figure 3E: Amine-defect synergistic enhancement of proton transfer on *N*5-*d*GA revealed using the Raman spectra from 2850 cm^{-1} to 3000 cm^{-1}.

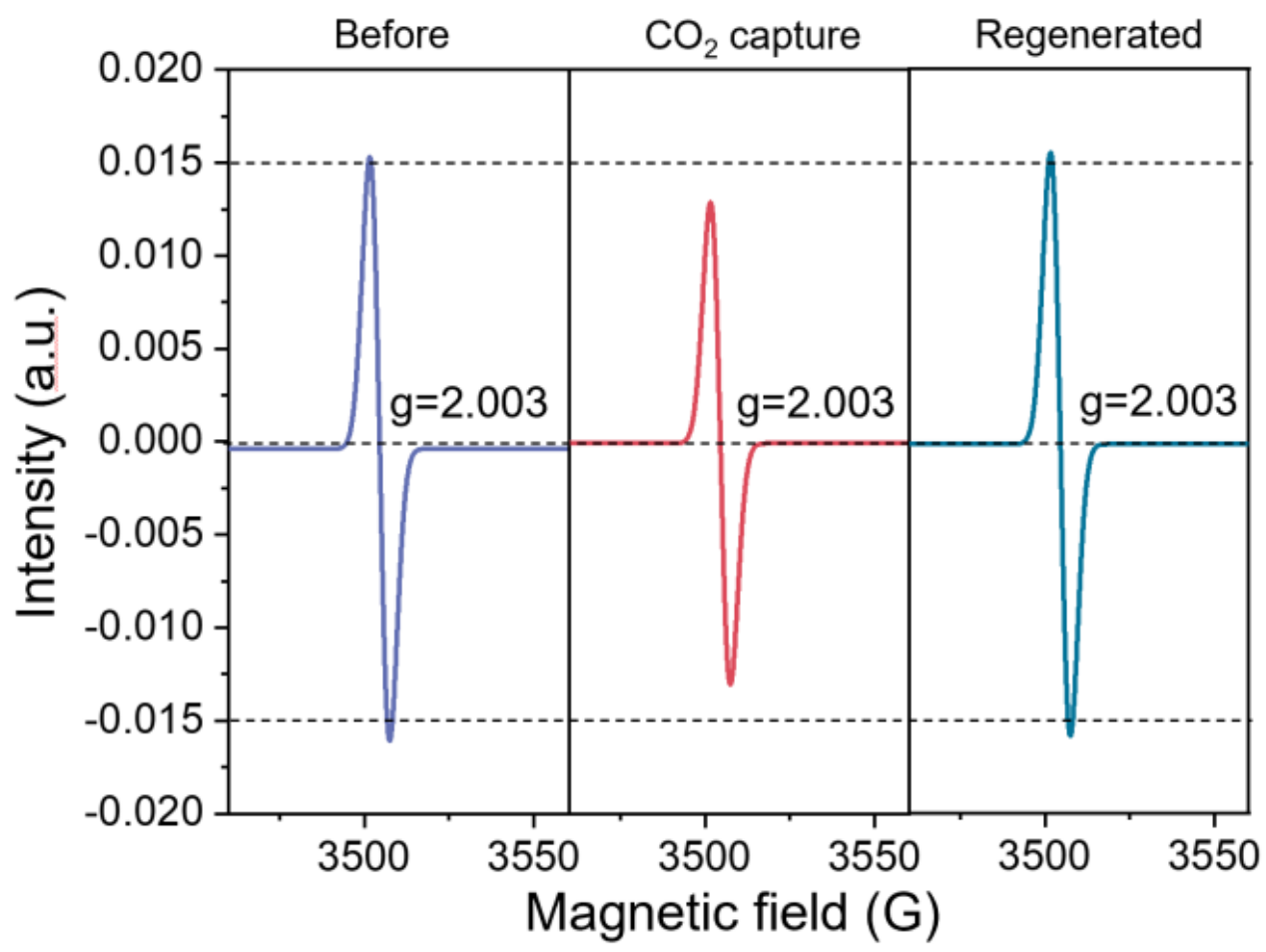

Figure S9: EPR spectra of *N*5-*d*GA before and after CO_2 capture, and after Joule heating regeneration.

2. The added details describing the experimental functionalisation procedures are generally adequate but remain somewhat sparse and could be further expanded to improve clarity and reproducibility. For example, it is unclear whether the plasma treated samples needed to be functionalized immediately or whether they could be stored (e.g., under a protective atmosphere) prior to amine functionalisation. In addition, the rationale for selecting the functionalisation conditions should be clarified, particularly given their implications for the TEA: a 10 hour treatment at 90°C represents

a relatively long and potentially energy intensive process.

Response: Thank you for your kind suggestion. Ar-induced point defects in graphene are highly stable at room temperature. (Just, S., et al. Preferential antiferromagnetic coupling of vacancies in graphene on SiO_2: Electron spin resonance and scanning tunneling spectroscopy. Phys. Rev. B, 90, 125449(2014)). Accordingly, the samples were stored in a sealed vessel under an Ar-protective atmosphere at room temperature after Ar-plasma treatment. Subsequent amine functionalization requires sufficient thermal activation at appropriate temperatures to promote amine grafting onto the graphene supports. The TG analysis results show that pentamine can maintain long-term stability at 90ºC (Figure R1), while significant thermal weight loss occurs above 150ºC (Figure S4). Therefore, 90ºC was selected as the temperature for amine functionalization to provide adequate yet mild thermal activation. Similar amine-functionalization conditions in terms of temperature and duration have been reported in the literature. (Sun, X., et al. Atom-level interaction design between amines and support for achieving efficient and stable CO_2 capture. Nat. Commun. 15, 5068 (2024); Surkatti, R., et al. Comparative analysis of amine-functionalized silica for direct air capture (DAC): Material characterization, performance, and thermodynamic efficiency. Sep. Purif. Technol. 354, 128641 (2025))

In addition, as shown in Table S8, the amine functionalization step has been included in the synthesis and post-processing cost. This cost was allocated to thermal reduction (26%), plasma treatment (22%), amine functionalization (12%), washing (20%), drying (10%), and auxiliary handling (10%). We agree that more precise cost control is important for large-scale material production, and further optimization of the synthesis and post-processing procedures will be pursued in future work.

The relevant content has been updated on page 16, lines 426-427 of the revised manuscript and Table S8 of the Supplementary Information.

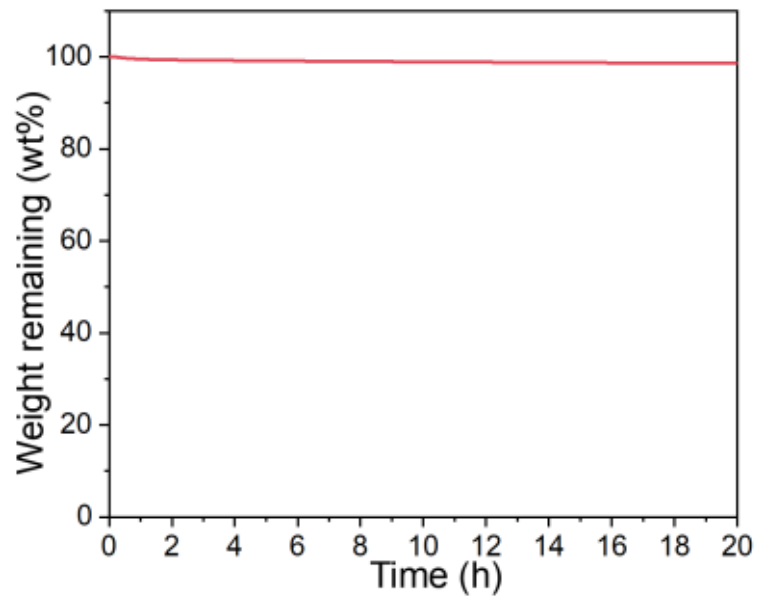

Figure R1: TG curves of thermal gravity curves of *penta*-amine at a constant temperature of 90°C.

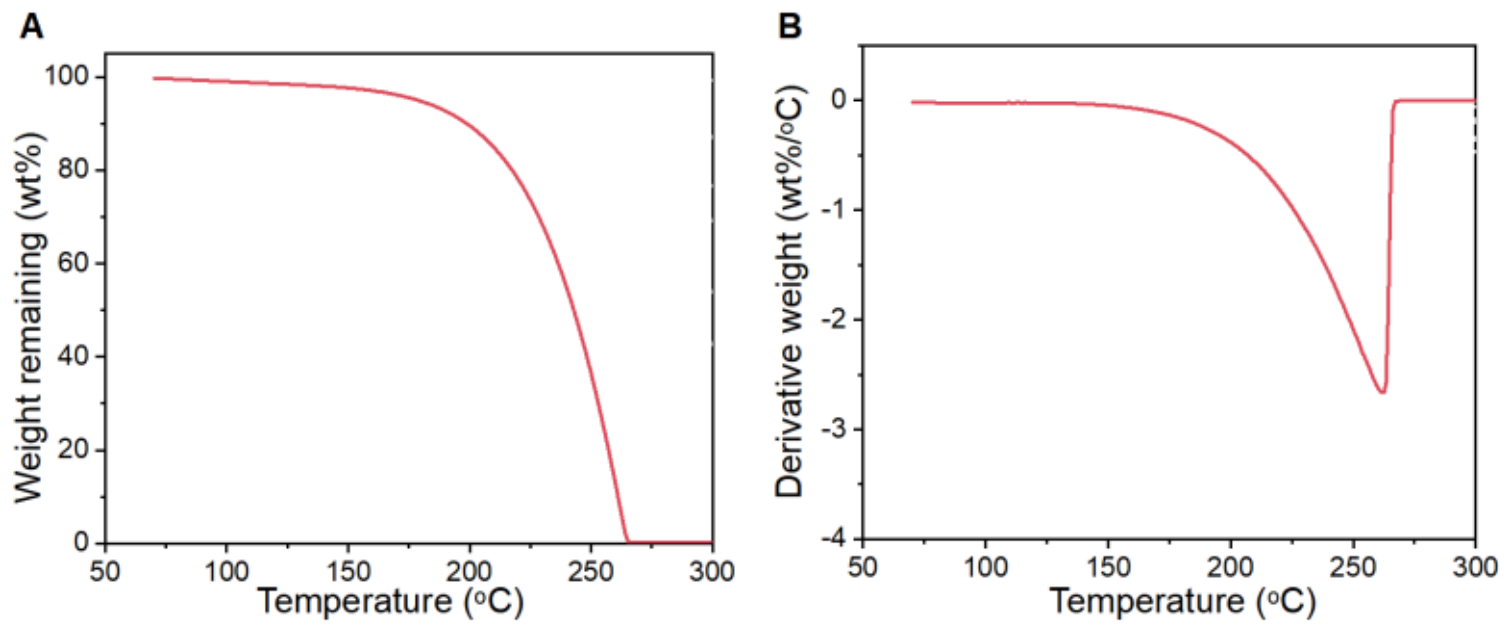

Extracted from Figure S4: (A) TG curves and (B) derivative of thermal gravity curves of *penta*-amine at a heating rate of 10°C min^{-1}.

Table S8: Process specific parameters of ESA-DAC.

Parameters	Value	Unit
Initial plant capacity	4000	t CO_2/a
adsorption capacity	6.57	mmol/g
adsorption time	6.00	h
desorption time	2.00	h
cooling time	4.00	h
Capacity factor	0.98	
Regenerative energy consumption	3.40	GJ/tCO_2
Cost of graphene aerogel	0.16	M$/t
Cost of *penta*-amine	0.004148	M$/t
Cost of synthesis and post-processing[a]	0.003	M$/t
Regeneration temperature	80.00	°C
Mass fraction of *penta*-amine	66.7	%
Mass fraction of graphene aeroge	33.3	%
Mass of initial sorbents	9.48	t
Cost of initial sorbents[b]	0.56	M$
CO_2 release ratio	95	%
Electricity cost of solar PV	39	$//MWh
Electricity cost of wind	37	$//MWh
Carbon intensity (x)	0.0176	
Additional electrical energy (like air blower, etc).	0.99	GJ/t CO_2

[a]The synthesis and post-processing cost was attributed to thermal reduction (26%), plasma treatment (22%), amine functionalization (12%), washing (20%), drying (10%), and auxiliary handling (10%).

[b]The resulting sorbent cost ($C_{sorbent}$) was calculated to be 0.059 M$ t^{-1}: $C_{sorbent}$=33.3 wt%×Cost of graphene aerogel+66.7 wt%×Cost of *penta*-amine+Cost of synthesis and post-processing. For an initial sorbent mass of 9.48 t, this gives an initial sorbent cost of approximately 0.56 M$.

3. I am not an expert in techno economic analysis or Qst calculations. Nevertheless, the work appears to be reasonably executed and appropriately integrated into the manuscript. It would, however, be important to more clearly specify how the TEA parameters were sourced and, in particular, to explicitly state the assumptions underlying the estimated costs of the graphene aerogel and the amine functionalisation process.

Response: In this techno-economic analysis, the cost parameters for a large quantity of chemicals were obtained from supplier quotations, with *penta*-amine quoted from Aladdin and aqueous GO dispersions quoted from Hangzhou Gaoxi Technology Co., Ltd. The process-specific parameters, including synthesis conditions, adsorption capacity, adsorption time, desorption time, regeneration energy consumption, regeneration temperature, CO_2 release ratio, were all taken from the experimental results of this work. Moreover general techno-economic assumptions, such as device cost, installation cost, engineering and procurement cost, EPC factors, owners' costs, electricity cost, carbon intensity, were adopted from Refs. 23-30 as shown in the Supplementary Information. These solid-sorbent DAC studies provide relevant benchmark values for the present techno-economic analysis. The above parameters were used in this work to calculate levelized capital expenditure (CAPEX), annual fixed operational expenditure (fixed OPEX), annual variable OPEX and the cost of CO_2 transport & storage for DAC.

We have also clarified the assumptions used to estimate the *N*5-*d*GA sorbent cost. The sorbent was assumed to contain 33.3 wt% graphene aerogel and 66.7 wt% penta-amine, with unit costs of 0.16 M$ t^{-1} and 0.004148 M$ t^{-1}, respectively. An additional 0.003 M$ t^{-1} was assigned to synthesis and post-processing, including Ar-plasma treatment, amine functionalization, washing, drying, etc. The resulting sorbent cost ($C_{sorbent}$) was calculated as 0.059 M$ t^{-1}: $C_{sorbent}$=33.3 wt% × Cost of graphene aerogel+66.7 wt%×Cost of *penta*-amine+Cost of synthesis and post-processing. For

an initial sorbent mass of 9.48 t, this gives an initial sorbent cost of approximately 0.56 M$, which is included in the Table S8.

These assumptions and the corresponding formula have now been added in Table S8 of the Supplementary Information.

Table S8: Process specific parameters of ESA-DAC.

Parameters	Value	Unit
Initial plant capacity	4000	t CO_2/a
adsorption capacity	6.57	mmol/g
adsorption time	6.00	h
desorption time	2.00	h
cooling time	4.00	h
Capacity factor	0.98	
Regenerative energy consumption	3.40	GJ/tCO_2
Cost of graphene aerogel	0.16	M$/t
Cost of *penta*-amine	0.004148	M$/t
Cost of synthesis and post-processing[a]	0.003	M$/t
Regeneration temperature	80.00	°C
Mass fraction of *penta*-amine	66.7	%
Mass fraction of graphene aeroge	33.3	%
Mass of initial sorbents	9.48	t
Cost of initial sorbents[b]	0.56	M$
CO_2 release ratio	95	%
Electricity cost of solar PV	39	$//MWh
Electricity cost of wind	37	$//MWh
Carbon intensity (x)	0.0176	
Additional electrical energy (like air blower, etc).	0.99	GJ/t CO_2

[a]The synthesis and post-processing cost was attributed to thermal reduction (26%), plasma treatment (22%), amine functionalization (12%), washing (20%), drying (10%), and auxiliary handling (10%).

[b]The resulting sorbent cost ($C_{sorbent}$) was calculated as 0.059 M$ t^{-1}: $C_{sorbent}$=33.3 wt%×Cost of graphene aerogel+66.7 wt%×Cost of *penta*-amine+Cost of synthesis and post-processing. For an initial sorbent mass of 9.48 t, this gives an initial sorbent cost of approximately 0.56 M$.

4. The inclusion of volumetric capacity data is an important improvement, as is the comparison with competing materials. The reported volumetric capacity is relatively low and may limit commercial feasibility; however, I do not consider this a barrier to publication in Nature Communications. For transparency, the volumetric capacity value should also be explicitly stated in the main text.

Response: We fully agree that volumetric capacity is an important parameter with strong engineering relevance. In future work, we will continue to devote efforts to the development of ESA materials with promising volumetric capacity. As suggusted, the volumetric capacity has been explicitly stated on page 7, lines 178-180 of the revised manuscript.

"Consequently, CO_2 adsorbed directly from the air by *N*5-*d*GA under humid conditions increased to 6.57 mmol g^{-1}, which was higher than that of the current state-of-the-art sorbents in Table S1, and 28.8% higher than the current record (Chen, H., Dong, H., Shi, Z. & SenGupta, A. K. Direct air capture (DAC) and sequestration of CO_2: Dramatic effect of coordinated Cu(II) onto a chelating weak base ion exchanger. Sci. Adv. 9, eadg1956, (2023)). It should be noted that the ultrahigh porosity of the graphene aerogel (>98%) leads to a CO_2 volumetric capacity of 52.6 mol m^{-3} for *N*5-*d*GA."

5. The recycling experiments are a valuable addition and demonstrate reasonably good chemical stability. While many more cycles would be required in a practical application, the 100 cycles presented here appear appropriate in the context of a primarily fundamental scientific investigation.

Response: We agree with you that more cycles would be required in a practical application. Therefore, the cycling test has been extended to 500 adsorption-desorption cycles under the identical DAC conditions. As shown in the revised Figure 4C, CO_2 uptake was recorded for every cycle during the first 100 cycles and then at 10-cycle intervals from 100 to 500 cycles. The CO_2 uptake of the as-prepared sorbent marginally decreases by 3.7% after the 100th cycle compared to the initial uptake, accompanied by the virtually unchanged amine loading. Notably, the CO_2 uptake remains essentially stable from 100 to 500 cycles, with no remarkable decline during the cyecling experiment. This indicates that the sorbent reaches a stable cyclic working state after the initial conditioning period.

The relevant content has been updated on page 10, lines 249-254 and Figure 4 of the revised manuscript.

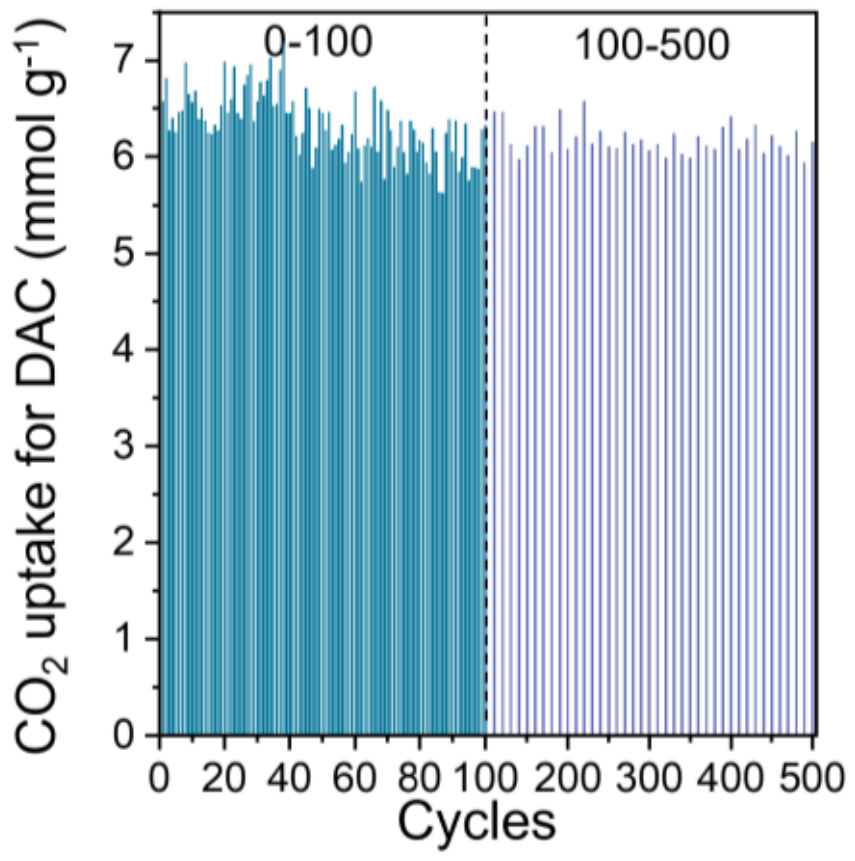

Figure 4C: CO_2 uptake of *N*5-*d*GA during 500 adsorption-electrothermal desorption cycles. CO_2 uptake values were recorded for each cycle from 0 to 100 cycles and at 10-cycle intervals from 100 to 500 cycles.

6. In my view, the authors have partially misunderstood this comment. Their response focuses on reaffirming the novelty of the proton trap process, whereas the original comment concerned the broader applicability of electrically swing adsorption (ESA) to defective material systems, including those with limited conductivity (e.g., defective MOFs combined with carbon to induce electrical heatability). That said, this is a relatively minor point intended to broaden the implications of the work and is not critical for publication in my view.

Response: Thank you for this constructive clarification. Owing to the advantages of high energy efficiency, power-input flexibility, and modular deployability, ESA process is well matched to renewable electricity sources such as wind and solar power. These features suggest that the ESA is a promising strategy for reducing the cost of direct air capture. In addition, ESA is not limited to carbon sorbents that are intrinsically conductive. Insulating or poorly conductive porous sorbents can also be adapted to ESA by introducing electrically conductive additives. For example, Zhao et al. demonstrated a Joule-heated DAC system that incorporated poorly conductive, amine-functionalized resins and carbon black (Zhao, S., et al. Electrically conductive amine-functionalized adsorbents driven by joule heating for direct air capture, ACS Sustain. Chem. Eng. 13, 14949-14960 (2025)). The conductive additive in this system enhanced the sorbent's

electrical heatability, enabling rapid CO_2 release and low-energy regeneration by Joule heating. More broadly, this strategy provides a useful reference for expanding proton-trap engineering to poorly conductive, amine-functionalized sorbents within ESA-DAC systems. We have added a perspective in the Discussion section to highlight the broader implications of this work.

"More broadly, the implications of the proton-trap strategy are not restricted to intrinsically conductive carbon sorbents. Defect-engineered porous materials with limited electrical conductivity could also be used into the ESA process by coupling them with conductive additives. In such hybrid sorbents, point defects may serve as proton-acceptors to promote assist amine-based CO_2 chemisorption, while the conductive component provides electrical heatability for Joule heating regeneration. This approach opens a broader materials-design space for next-generation amine-functionalized sorbents."

The relevant content has been updated on page 14, lines 352-359 of the revised manuscript.

7. This point has been fully addressed. Revising the main text to present data exclusively under ambient conditions (400 ppm CO_2) is a significant and necessary improvement and avoids potential misunderstandings, as previously noted by another reviewer.

Response: We are pleased that the previous revision has fully addressed this point and substantially improved the clarity and rigor of the manuscript. Thank you again for your positive assessment and constructive suggestions throughout the review process.

Reviewer #5:

General comment: The comments given to authors are well addressed.

Response: Many thanks for this kind feedback, we appreciate your input.

Reviewer #6:

General comment: The revised manuscript has been substantially improved compared

to the previous version. The overall structure is now significantly clearer, allowing the central concept and mechanistic insights to be more readily appreciated. The newly incorporated operando/in situ characterizations and the quantitative assessment of Joule-heating-driven regeneration further strengthen the key scientific claims of the work. The manuscript is suitable for publication after the following concerns are clarified.

Response: Thank you very much for your positive evaluation and for recognizing the improvements in the revised manuscript. We have carefully addressed all remaining comments in the point-by-point responses below.

1. From lines 174-177, the authors report that the kinetic rate constant decreases in the presence of water, indicating slower adsorption kinetics. In contrast, the calculations in lines 198-204 suggest that water enhances CO2 adsorption via hydrogen bonding. These observations appear to be inconsistent. The authors are encouraged to provide a more detailed discussion to explain the apparent discrepancy between kinetic and thermodynamic effects of water.

Response: The kinetic rate constant discussed in lines 174-177 of the origin manuscript is the Thomas-model rate constant fitted from fixed-bed breakthrough curves. This parameter is a typical apparent kinetic rate constant. It reflects the overall breakthrough behavior of the packed bed and includes contributions from external mass transfer and intrinsic reaction.

$$\ln\left(\frac{C_0}{C}-1\right)=\frac{k_{\mathrm{T}}q_0m_{\mathrm{sample}}}{Q}-k_{\mathrm{T}}C_0t_{\mathrm{r}} \qquad \text{(Equation S13)}$$

By contrast, the calculations in lines 198-204 of the origin manuscript only describe the intrinsic reaction effect of water at the adsorption site. Water molecules capable of forming hydrogen bonds with the adsorbed CO_2 reduce the potential energy of adsorption product by 0.80 eV, resulting in a more favorable CO_2 adsorption state. This is consistent with the increased CO_2 uptake under humid conditions.

However, under high humidity, adsorbed water or hydrated amine domains can increase the mass-transfer resistance for CO_2 within the fixed bed. As reported by Hahn

et al. (Hahn, M. W., Steib, M., Jentys, A. & Lercher, J. A. Mechanism and kinetics of CO_2 adsorption on surface bonded amines. J. Phys. Chem. C 119, 4126-4135, (2015)), a thin water film forms on the sorbent surface at high RH, creating a physical diffusion barrier for CO_2. This results in a 12% lower apparent kinetic rate constant, despite the more favorable intrinsic CO_2 adsorption reaction. Therefore, the higher CO_2 uptake and the lower apparent kinetic rate constant under humid conditions are not contradictory.

The relevant content has been updated on page 8, lines 212-214 of the revised manuscript.

2. Figure 4c shows a slight but noticeable decline in CO2 uptake over cycling. The authors are encouraged to clarify the underlying cause.

Response: Thank you for your careful observation. To clarify the slight decrease during early cycling, we extended the cycling test to 500 adsorption-desorption cycles under identical DAC conditions. As shown in the revised Figure 4C, the CO_2 uptake of *N*5-*d*GA remains essentially stable from 100 to 500 cycles, with no further measurable decline beyond experimental fluctuation. TG analysis of cycled *N*5-*d*GA shows negligible change in amine loading, while XPS N1s spectra and Raman spectroscopy confirm that the covalent grafting between amine groups and dGA remains intact (Figures S17-S19), indicating no significant degradation during repeated electrothermal regeneration (Min, K., et al. Oxidation-stable amine-containing adsorbents for carbon dioxide capture. Nat. Commun. 9, 726 (2018)).

We therefore attribute the initial decrease mainly to bed-level conditioning during the early adsorption-desorption cycles. For example, Al-Janabi et al. showed that velocity variations can affect CO_2 breakthrough curves in fixed-bed adsorption tests (Al-Janabi, N., et al., Velocity variation effect in fixed bed columns: A case study of CO_2 capture using porous solid adsorbents. AIChE J., 64, 2189-2197 (2018)). Chahbani and Tondeur further demonstrated that pressure drop can affect the dynamic behavior of fixed-bed adsorbers during adsorption and desorption (Chahbani, M. H., Tondeur, D. Pressure drop in fixed-bed adsorbers. Chem. Eng. J. 81, 23-34 (2001)). Under repeated gas flow and Joule heating regeneration, the packed bed may undergo minor

redistribution before reaching a stable configuration, leading to a small change in the apparent breakthrough uptake. After this initial conditioning period, the bed reaches a stable cyclic working state, as reflected by the nearly unchanged CO_2 uptake from 100 to 500 cycles.

The relevant content has been updated on page 10, lines 249-254 and Figure 4 of the revised manuscript.

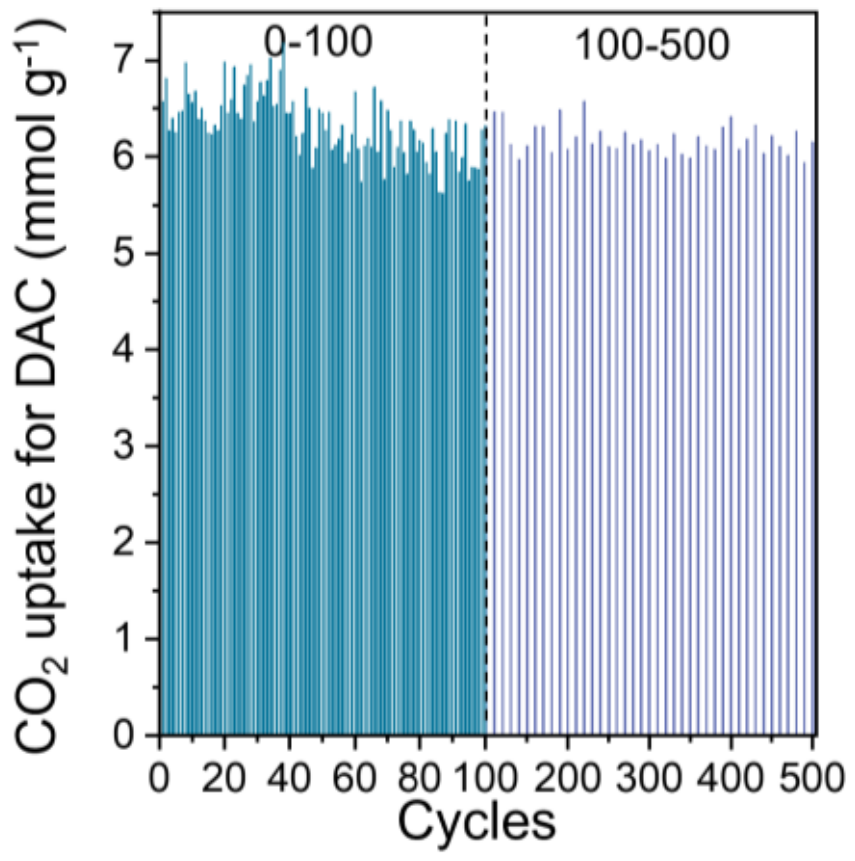

Figure 4C: Bar graph of 500 cycles of CO_2 adsorption-electrothermal desorption. CO_2 uptake values were recorded for each cycle from 0 to 100 cycles and at 10-cycle intervals from 100 to 500 cycles.

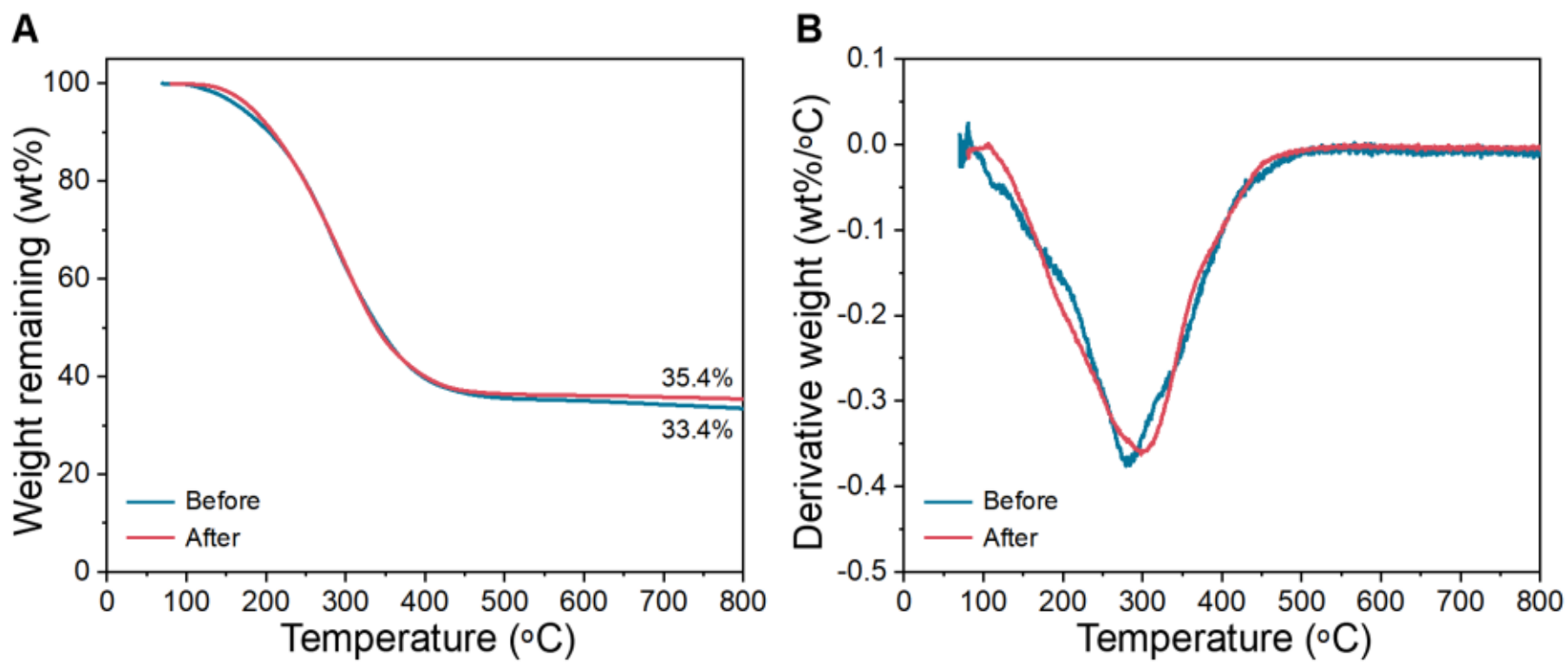

Figure S17: (A) TG curves and (B) derivative of thermal gravity curves of *N5-d*GA before and after 100 adsorption-desorption cycles.

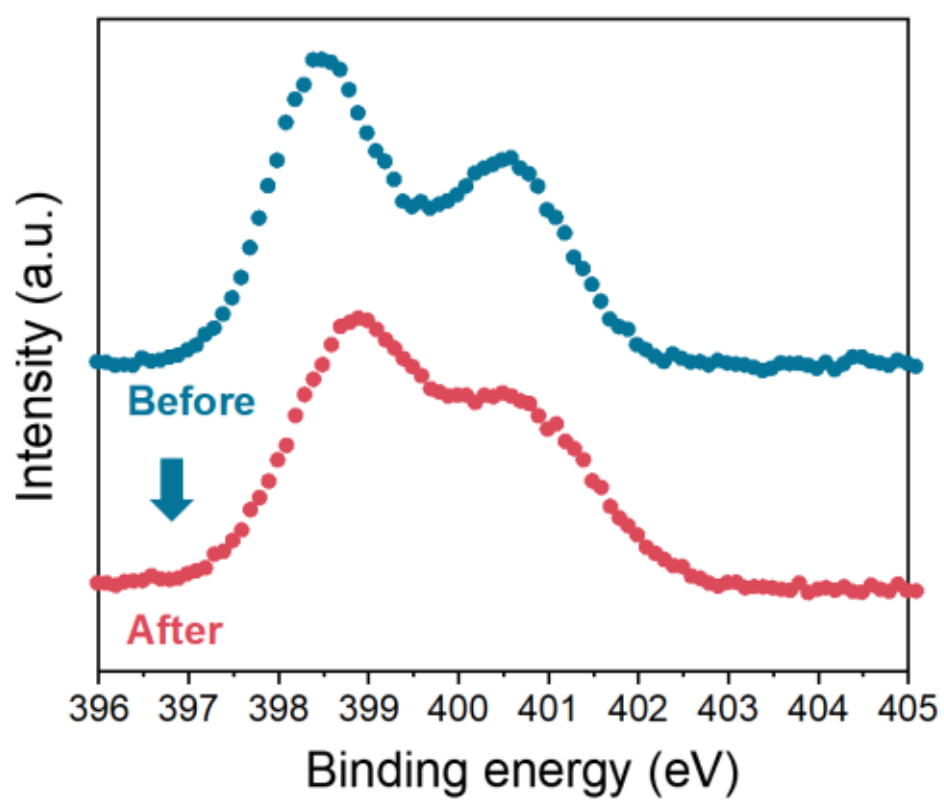

Figure S18: High-resolution XPS N1s spectra of *N*5-*d*GA before and after 100 adsorption-desorption cycles.

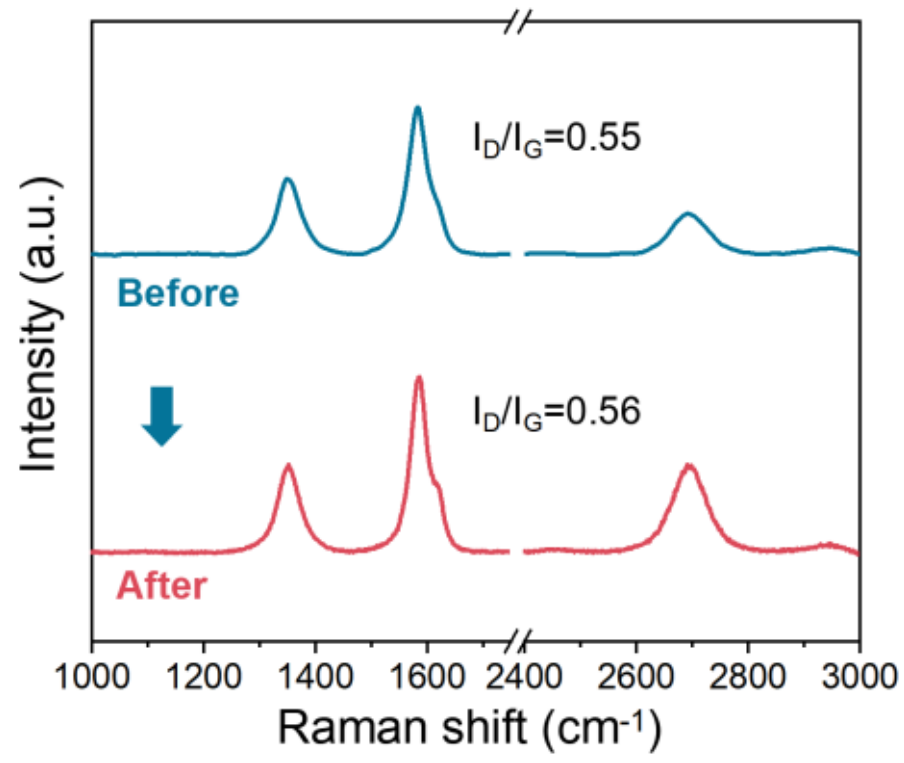

Figure S19: Raman spectroscopy of *N*5-*d*GA before and after 100 adsorption- desorption cycles.

3. The authors refer to a 95% CO_2 release in both the experimental demonstration (line 192) and the techno-economic analysis (line 295). Do these two values have the same meaning, that 95% of the sorbent was regenerated? If regeneration is incomplete, does the remaining fraction of adsorbed CO_2 accumulate over cycles, potentially leading to performance degradation? Further clarification on the definition and implications of this release ratio would be helpful.

Response: Sorry for the lack of clarity. The 95% CO_2 release ratio has the same meaning in both the experimental demonstration and in the techno-economic analysis. It refers to the fraction of the initially adsorbed CO_2 that is released during each Joule heating regeneration step. As shown in Figure 5E, increasing the release ratio beyond 95% provides only a small amount of additional working capacity, but requires higher operational expenditure. Therefore, 95% was selected as a practical compromise between CO_2 capture efficiency and cost for ESA-DAC. This value was selected for energy and cost analysis, and does not indicate that *N*5-*d*GA cannot be fully regenerated or that residual CO_2 would necessarily accumulate during cycling.

We also clarify that the cycling test in this work was not performed under this 95% CO_2 release condition. Each cycle involved saturated adsorption followed by complete regeneration. As shown in Figure 4B, the CO_2 desorption signal returned to the baseline after electrothermal regeneration, indicating that CO_2 was completely released under the experimental protocol. In addition, the cyclic CO_2 uptake remains essentially stable, demonstrating that progressive CO_2 accumulation does not occur over repeated cycles.

The relevant statements have been further clarified in page 10, lines 244-245 of the revised manuscript.

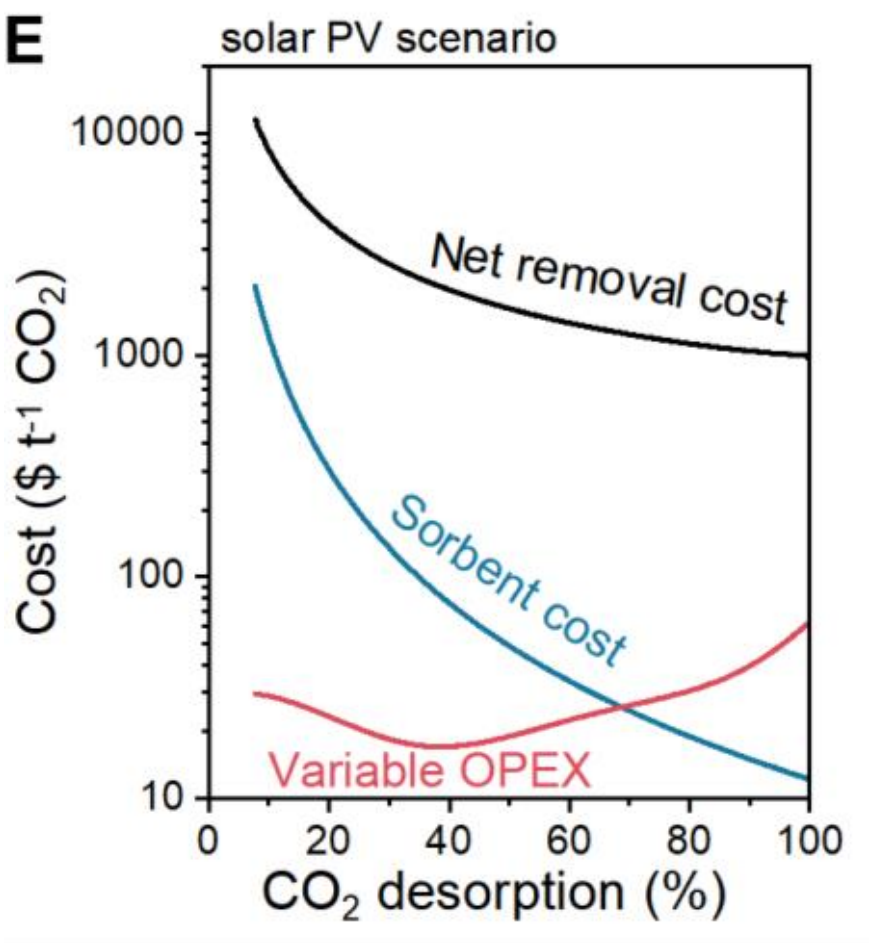

Figure 5E: Relationship between DAC cost by solar PV and amount of CO_2 desorbed in the ESA-DAC system.

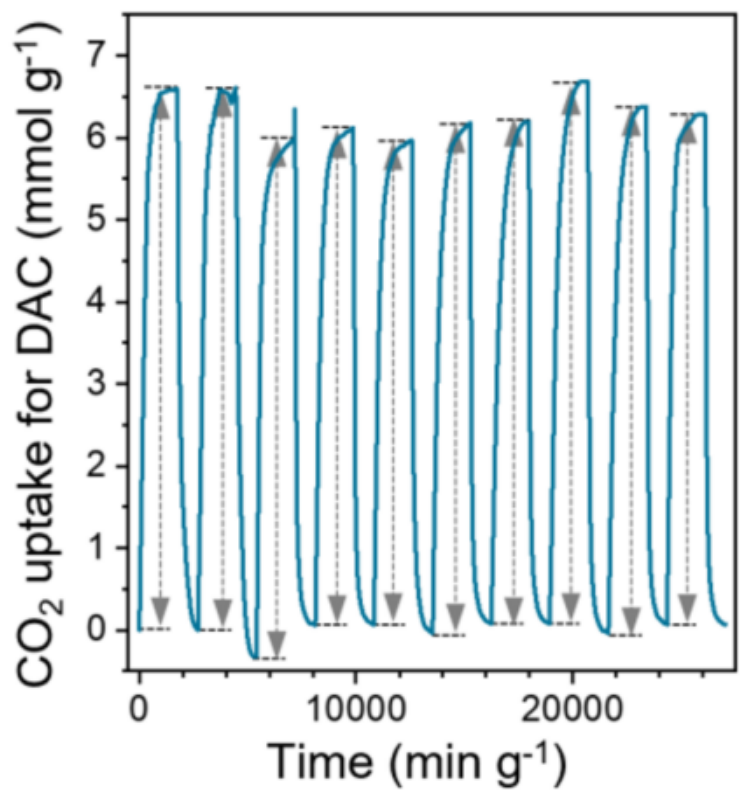

Figure 4B: 10 cycles of CO_2 adsorption–electrothermal desorption. The gray arrows indicate the cyclic capacity for each cycle.

4. In response to Question 11, the authors state that the regeneration energy and temperature are lower than those reported in prior studies. However, a key claim of this work is the enhanced CO_2 binding under dilute conditions. From a thermodynamic perspective, stronger binding is typically associated with higher desorption energy barriers. Therefore, it is not clear how both enhanced capture and facile regeneration are simultaneously achieved.

Response: Thank you for raising this important comment. We agree that stronger CO_2 binding under dilute conditions is generally associated with higher desorption energy barriers. In our case, *N*5-*d*GA shows clear chemisorption characteristics, with an adsorption isosteric heat of 60.7 kJ mol^{-1} (Table S1). This strong CO_2 affinity contributes to its high capture capacity under dilute CO_2 conditions. The facile

regeneration is mainly enabled by the ESA process. Unlike conventional thermal regeneration, Joule heating generates heat directly within the conductive adsorbent bed, thereby minimizing inefficient external heat transfer and enabling CO_2 release at moderate temperatures. In addition, the electric field further facilitates the regeneration process. As shown by the DFT calculations, at an electric-field intensity of 0.01 V nm^{-1}, the regeneration potential energy decreases by 22.6% (Figure 2C), and the CO_2 desorption energy barrier is reduced by 28.9% compared with that in the absence of an electric field (Figure 2D). Therefore, in this work, *N*5-*d*GA simultaneously achieves enhanced CO_2 capture and facile regeneration.

The relevant content has been further clarified in page 10, lines 240-242 of the revised manuscript.

Extracted from Table S1: CO_2 uptake for DAC of various reported sorbents.

**Type**	**Name**	**q (mmol g^{-1}, dry)**	**q (mmol g^{-1}, humid)**	**Desorption temperature (°C)**	**Heat duty for regeneration (GJ t^{-1})**	**Ref.**
TSA/ TVSA	MIL-101(Cr) _50 wt % TEPA	0.71[a]	0.95[a]	90	110 (kJ mol^{-1})[c]	12
	Developed by Oy Hydrocell Ltd.	0.54	0.89	90	67.9 (kJ mol^{-1})[c]	13
	2-UIO-EDA	0.04	0.44	140	58.13 (kJ mol^{-1})[c]	14
	MOR-type zeolites	1.05	1.02	100	-	
	13X-type zeolites	0.39	0.49	200~300	30 (kJ mol^{-1})[c]	15
	SAPO-34+MOR	0.9	0.94	100	-	
	PEI-ePTFE/silica	1.08[a]	1.5[a]	110	67 (kJ mol^{-1})[c]	16
	Polyam-N-Cu^{2+}	-	5.1	80	51.1 (kJ mol^{-1})[c]	17
	COF-999	0.96	2.05	60	53 (kJ mol^{-1})[c]	18
	Lewatit® VP OC 1065	0.63	1.16		54.9 (kJ mol^{-1})[c]	19
ESA/ Joule heating	PEI-carbon fiber	0.39[a]	1.2[a]	90~110<br>0.8~1.0 (A)[b]	7.2	8
	Ag-NC coated fiber	0.42	-	110<br>1.9 (A)[b]	6.1	9
	PCS-OH	-	0.26	100	6.5~11.4	20
	Ad-0.48PE/0.32HP/0.20CB	-	2.4	90	3.5~3.7	21
	PEI-CNF	1.5	4.0	80	-	22
	*N*5-*d*GA	2.93<br>2.91[a]	6.57<br>6.41[a]	80<br>0.6 (A)[b]	3.4<br>60.7 (kJ mol^{-1})[c]	This work

[a]The pseudo-equilibrium CO_2 uptake for DAC (C/C_0=0.95).
[b] The current intensity for CO_2 desorption, A.

[c] Isosteric adsorption heat or adsorption enthalpy change, kJ mol^{-1}.

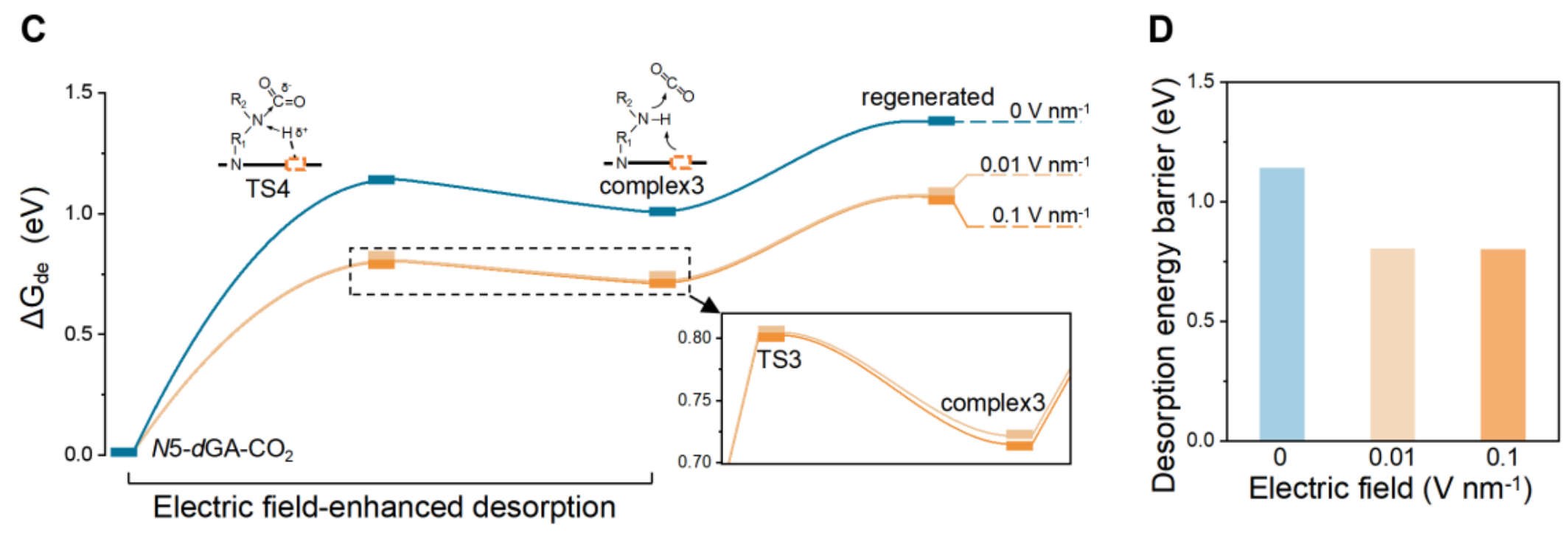

Extracted from Figure 2: (C) The CO_2 desorption pathway and (D) desorption energy barrier of *N*5-*d*GA under different electric field strengths.

Editors:

Additional editorial comments: Data presentation.

Response: We have revised the relevant bar graphs to better show the distribution of the underlying data. For plots with sample sizes below 10, like Figure 3A and S7, all individual data points are now shown.

The updated figures are shown in the revised manuscript. The numerical source data underlying these plots have also been provided in the Source Data file.

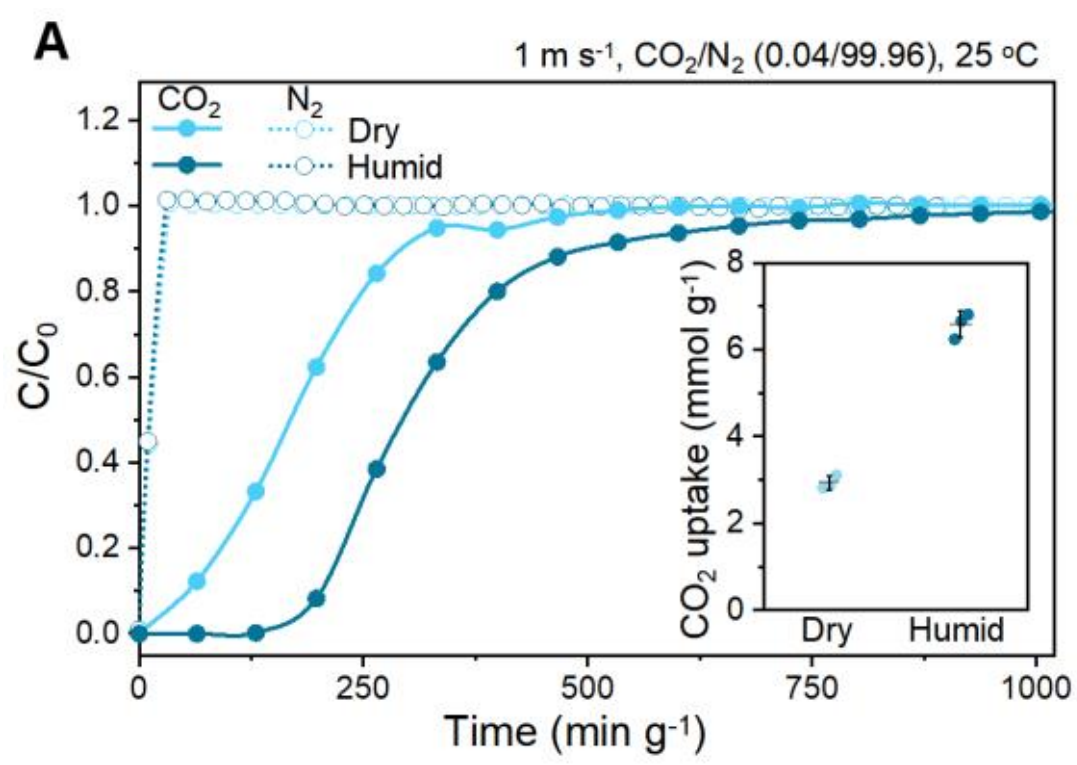

Figure 3A. Breakthrough curves and CO_2 uptakes adsorbed directly from the air of *N*5-*d*GA under dry and humid conditions.

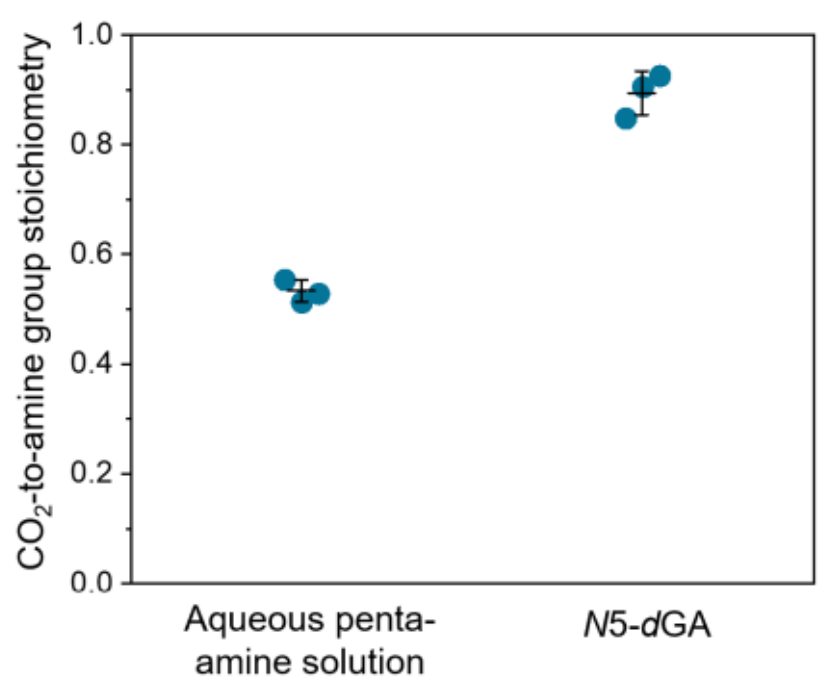

Figure S7: Comparison of the CO_2-to-amine group stoichiometry for the aqueous pentaamine solution and *N*5-*d*GA.

Point-by-Point Response to Referees

Reviewer #4:

General comment: The authors have fully addressed all my queries. I have no further comments.

Response: We sincerely thank the reviewer for their constructive comments throughout the review process. We are pleased that our revisions have addressed the reviewer's queries.

Reviewer #6:

General comment: The authors have satisfactorily clarified the concerns raised in my previous review. I appreciate the additional explanation and revisions provided in the manuscript. In my opinion, the manuscript is now suitable for publication in Nature Communications..

Response: We would like to thank the reviewer for their constructive feedback and positive assessment of our revised manuscript. We are glad that our additional explanations and revisions have addressed the reviewer's previous concerns.
